# Supplementary material for: Halogen–sodium exchange enables efficient access to organosodium compounds
Source: Commun Chem. 2021 May 24;4:76. doi: 10.1038/s42004-021-00513-2 (PMC9814623; doi:10.1038/s42004-021-00513-2)
Supplement: Supplementary file 1 — Supplementary Information [file 42004_2021_513_MOESM1_ESM.pdf]

Supplementary Information

Halogen–Sodium Exchange
Enables Efficient Access to Organosodium Compounds

Sobi Asako,*[1,2] Ikko Takahashi,[2] Hirotaka Nakajima,[1] Laurean Ilies,[2] and Kazuhiko Takai*[1]

[1] *Division of Applied Chemistry, Graduate School of Natural Science and Technology, Okayama University, 3-1-1 Tsushimanaka, Kita-ku, Okayama 700-8530, Japan*

[2] *RIKEN Center for Sustainable Resource Science, 2-1 Hirosawa, Wako, Saitama 351-0198, Japan*

*correspondence to: sobi.asako@riken.jp; ktakai@cc.okayama-u.ac.jp

Table of Contents

1. **Supplementary Methods**
2. **Supplementary References**
3. **Supplementary Figures**

1. Supplementary Methods

General. All reactions dealing with air- or moisture-sensitive compounds were carried out in a dry sealed reaction tube under an atmosphere of nitrogen or argon. Glass-coated stirring bars were used for the reactions using sodium dispersion, which showed slightly better efficiency than teflon-coated stirring bars. Analytical thin-layer chromatography was performed on glass plates coated with 0.25 mm silica gel containing a fluorescent indicator (Merck #1.05715 TLC Silica gel 60 F_{254}). Flash silica gel column chromatography was performed on silica gel 60N (Kanto, spherical and neutral, 40–50 µm) as described by Still et al.[1] ^{1}H NMR, ^{13}C NMR, and ^{19}F NMR spectra were recorded on a JEOL ECZ-400R and ECA-500 spectrometers and reported in parts per million using Me_4Si (^{1}H: 0.00 ppm), $CHCl_3$ (^{1}H: 7.26 ppm), $CDCl_3$ (^{13}C: 77.0 ppm) and C_6F_6 (^{19}F: –164.9 ppm) as an internal reference, respectively. The data is presented as follows: chemical shift, multiplicity (s = singlet, d = doublet, t = triplet, q = quartet, m = multiplet and/or multiplet resonaces, br = broad), coupling constant in Hertz (Hz), and integration. The melting points of solid materials were determined on a Yanaco MP-500 apparatus and were uncorrected. Gas chromatographic (GC) analyses were performed on Shimadzu GC-2025 equipped with an FID detector and a capillary column (HR-1, 0.25-mm i.d. x 25 m). Gel permeation chromatography (GPC) was performed on SHIMADZU LC-20A equipped with FP-2002 columns. Mass spectra (GC-MS) were taken at SHIMADZU QP2010SE equipped with a capillary column (Rxi-5Sil MS, 0.25-mm i.d. x 30 m). High resolution mass spectra were obtained on a Bruker microTOF-Q III (APCI).

Materials. Anhydrous hexane, THF, NMP, and benzene were purchased from Kanto Chemical Co., Inc. Anhydrous hexane was stored over activated molecular sieves 4A under nitrogen in a Schlenk flask. Methylcyclohexane (MCH) was purchased from Tokyo Chemical Industry Co., Ltd. and stored over activated molecular sieves 4A under nitrogen in a Schlenk flask. (*E*)-(2-Bromovinyl)benzene (**1t**) was prepared according to the literature procedure.[2] An *E*/*Z* mixture of (2-bromovinyl)benzene (**1t**) was purchased from BLDpharm and the ratio of its stereoisomers was determined by GC measurement. $ZnCl_2$•TMEDA was prepared according to the literature procedure.[3] 1-Chloro-2,2-dimethylpropane (neopentyl chloride) was purchased form Tokyo Chemical Industry Co., Ltd. and stored over activated molecular sieves 4A. Pd-PEPPSI-IPr and Pd-PEPPSI-IPent were purchased from Sigma–Aldrich Inc. Sodium dispersion (SD) was provided by KOBELCO ECO-Solutions Co., Ltd (the sodium dispersion is also commercially available from Tokyo Chemical Industry Co., Ltd. [code: D5792] and FUJIFILM Wako Chemicals [code: 638-46321]). The quality of sodium dispersion gradually deteriorates upon contact with air. Once exposed to air, it is recommended that sodium dispersion should be used within two weeks for high efficiency

and reproducibility. We typically subdivided the sodium dispersion into several dried vials and stored them under argon at –20 °C. If stored properly without exposure to air, the quality of sodium dispersion remains high for several months. The vials containing sodium dispersion was shaken vigorously before use. Unless otherwise noted, materials were obtained from commercial sources and used without further purification.

General Procedure for Halogen–Sodium Exchange

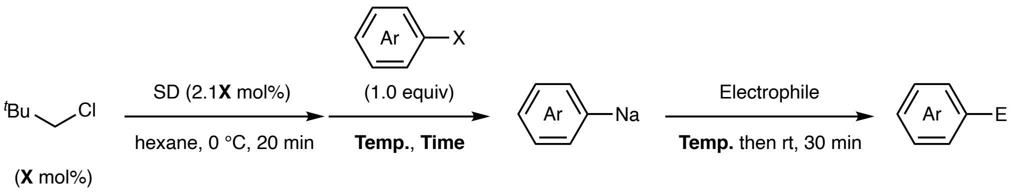

In a dry Schlenk tube equipped with a glass-coated stirring bar, neopentyl chloride (0.50 mmol, 200 mol%) was added to a mixture of hexane (2.0 mL) and sodium dispersion (ca. 26 wt%, 420 mol%) under nitrogen at 0 °C. After vigorously stirring at 0 °C for 20 min, the aryl halide (0.25 mmol, 1.0 equiv) was added at 0 °C and the reaction mixture was vigorously stirred for 30 min to form the corresponding arylsodium compound. The electrophile was added at the same temperature and the reaction mixture was vigorously stirred at room temperature for 30 min, and then it was quenched with H_2O. After extraction with ethyl acetate for three times, the combined organic layers were passed through a pad of silica gel and concentrated under reduced pressure. The crude product was purified by column chromatography on silica gel using the indicated eluent to afford the desired compound.

Caution: organosodium compounds are insoluble in hexane, and therefore efficient and vigorous stirring is very important.

Neopentylsodium was stable at least for 1 h at 0 °C in hexane after preparation and could be used for the halogen–sodium exchange reaction without decrease in efficiency (Supplementary Figure 1).

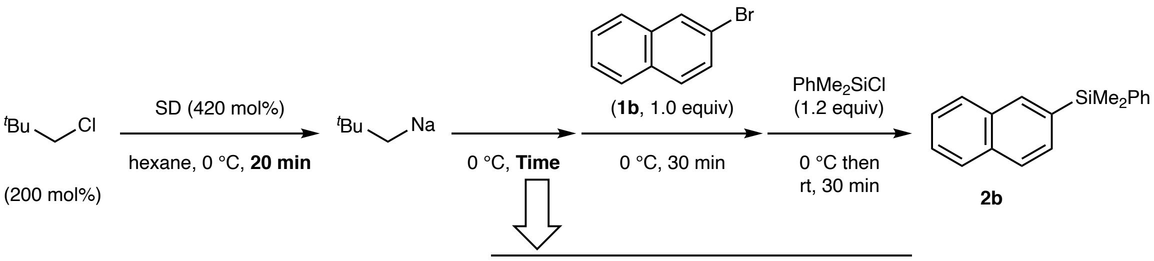

Time (min)	**2b** (%)[a]	color
0	>99	dark gray
30	>99	dark gray
60	>99	black
90	94	dark brown
120	93	dark brown

Supplementary Figure 1. Stability and reactivity of neopentylsodium for halogen–sodium exchange. [a]Yields were determined by ^{1}H NMR measurement using 1,1,2,2-tetrachloroethane as an internal standard.

Photographic Depiction of a Typical Reaction Setup: Handling of Sodium Dispersion, Preparation of Neopentylsodium, and Halogen–Sodium Exchange Reaction

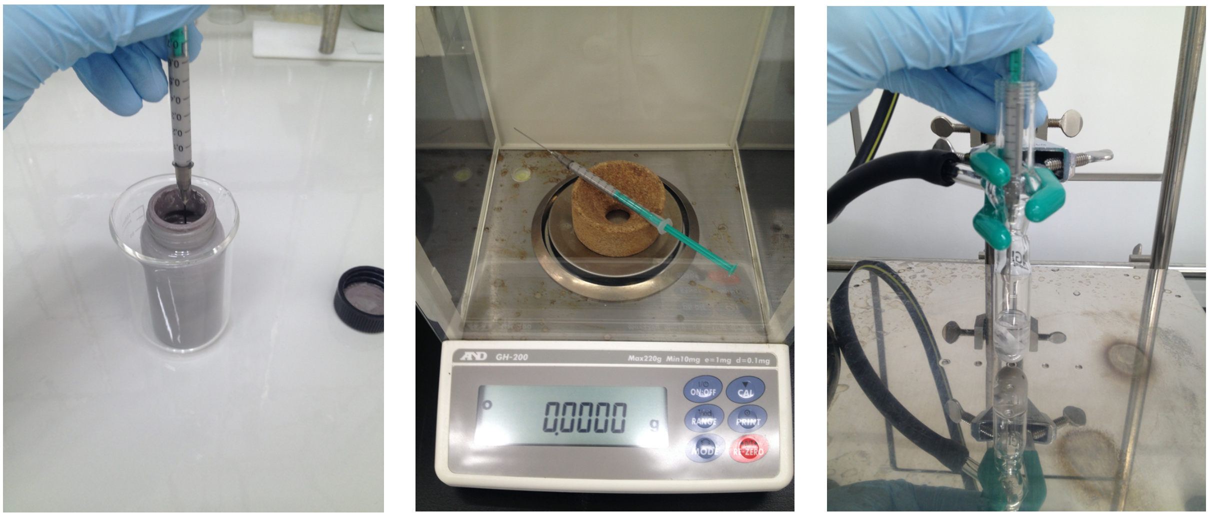

Supplementary Figure 2. Left: Sodium dispersion is taken up by syringe under air. **Middle**: Weighting the syringe containing sodium dispersion (calibrated to 0). **Right**: Sodium dispersion is added to hexane placed in a Schlenk tube under a N_2 flow.

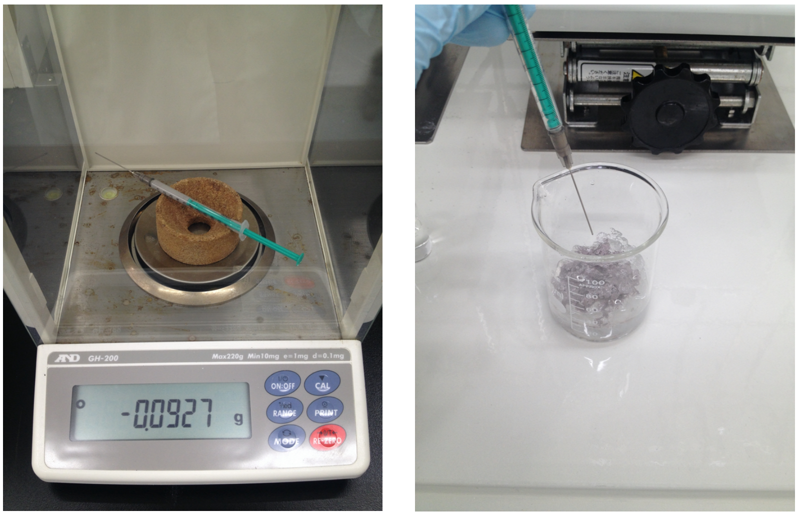

Supplementary Figure 3. Left: Weighting the syringe after adding the sodium dispersion to the reaction vessel. **Right**: The residual sodium dispersion is quenched by ice water.

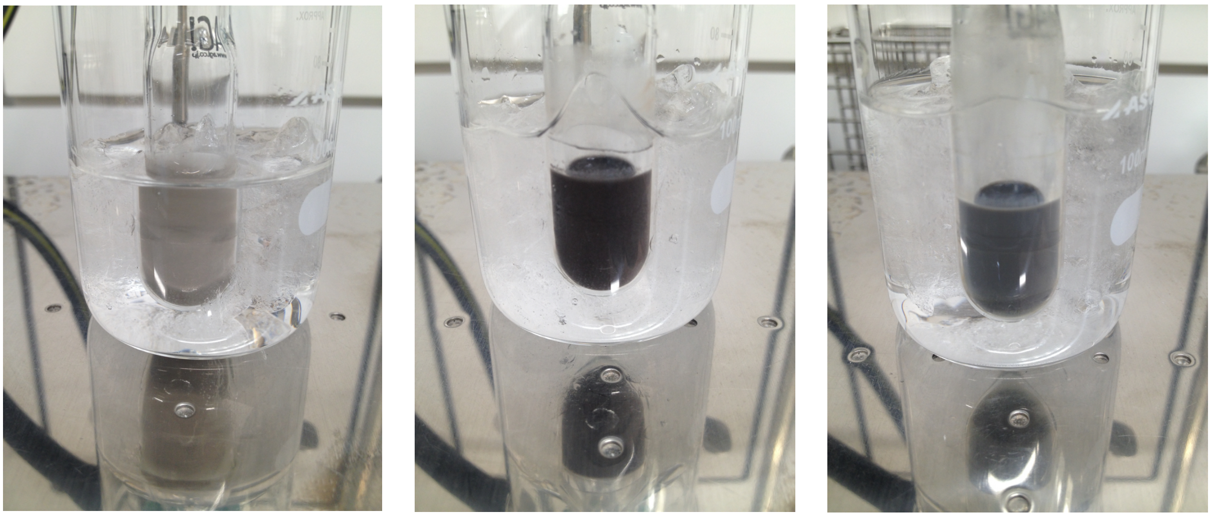

Supplementary Figure 4. Left: After addition of neopentyl chloride, the reaction mixture is stirred at 0 °C, cooled by an ice-water bath. **Middle**: After stirring for 20 min at 0 °C, the reaction mixture turns dark gray (almost black), which indicates the generation of neopentylsodium. **Right**: Halogen–sodium exchange of 9-bromophenanthrene (**1c**) with neopentylsodium is completed after 30 min to afford 9-phenanthrenylsodium. All reagents are added to the reaction mixture under a flow of N_2.

Dimethyl(naphthalen-1-yl)(phenyl)silane (2a)

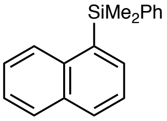

The general procedure was applied to neopentyl chloride (53.1 mg, 0.50 mmol), sodium dispersion (25.5 wt%, 96.8 mg, 1.07 mmol), 1-bromonaphthalene (**1a**, 50.9 mg, 0.25 mmol), and $PhMe_2SiCl$ (50 μL, 0.30 mmol). The crude product was purified by column chromatography on silica gel (hexane/chloroform = 100:3) to afford the title compound as a colorless oil (60.1 mg, 93%). The spectral data were in accordance with those reported in the literature.[4]

- Reaction using 1-iodonatphthalene (1a') as the substrate

The general procedure was applied to neopentyl chloride (53.1 mg, 0.50 mmol), sodium dispersion (26.2 wt%, 93.9 mg, 1.07 mmol), 1-iodonaphthalene (**1a'**, 64.0 mg, 0.25 mmol), and $PhMe_2SiCl$ (50 μL, 0.30 mmol). The crude product was purified by column chromatography on silica gel (hexane/chloroform = 100:3) to afford the title compound as a colorless oil (53.3 mg, 81%).

Dimethyl(naphthalen-2-yl)(phenyl)silane (2b)

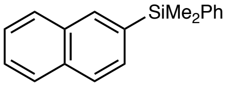

The general procedure was applied to neopentyl chloride (56.8 mg, 0.53 mmol), sodium dispersion (25.5 wt%, 100.6 mg, 1.11 mmol), 2-bromonaphthalene (**1b**, 55.4 mg, 0.27 mmol), and $PhMe_2SiCl$ (50 μL, 0.30 mmol). The crude product was purified by column chromatography on silica gel (hexane/chloroform = 100:3) to afford the title compound as a colorless oil (65.9 mg, 94%). The spectral data were in accordance with those reported in the literature.[4]

Dimethyl(phenanthren-9-yl)(phenyl)silane (2c)

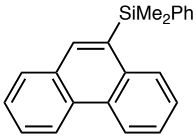

The general procedure was applied to neopentyl chloride (53.1 mg, 0.50 mmol), sodium dispersion (25.7 wt%, 95.3 mg, 1.07 mmol), 9-bromophenanthrene (**1c**, 63.2 mg, 0.25 mmol), and $PhMe_2SiCl$ (50 μL, 0.30 mmol). The crude product was purified by column chromatography on silica gel (hexane/chloroform = 100:3) to afford the title compound as a colorless solid (75.8 mg, 99%).

Melting point: 124–125 °C; ^{1}H NMR (500 MHz, $CDCl_3$): δ 8.79 (d, J = 8.0 Hz, 1H), 8.74 (d, J = 8.0 Hz, 1H), 8.12 (s, 1H), 8.07 (d, J = 8.6 Hz, 1H), 7.96 (d, J = 8.1 Hz, 1H), 7.73 (ddd, J = 8.0, 6.9, 1.2 Hz, 1H), 7.68–7.64 (m, 4H), 7.52 (ddd, J = 8.0, 6.9, 1.2 Hz, 1H), 7.46–7.34 (m, 3H), 0.84 (s, 6H); ^{13}C NMR (126 MHz, $CDCl_3$): δ 138.8, 136.9, 134.8, 134.3, 134.1, 131.2, 131.1, 130.1, 129.5, 129.1, 128.9, 127.9, 127.3, 126.6, 126.2, 126.0, 123.1, 122.4, –0.9; GC-MS (EI) *m/z* (relative intensity): 313 (16), 312 (M^+, 49), 298 (29), 297 (100), 207 (94), 44 (50); HRMS (APCI+): *m/z* Calcd. for $C_{22}H_{20}Si$ $[M]^+$: 312.1329; found: 312.1335.

Trimethyl(phenanthrene-9-yl)silane (2c')

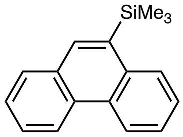

The general procedure was applied to neopentyl chloride (52.9 mg, 0.50 mmol), sodium dispersion (26.1 wt%, 92.7 mg, 1.05 mmol), 9-bromophenanthrene (**1c**, 64.3 mg, 0.25 mmol), and Me_3SiCl (38 μL, 0.30 mmol). The crude product was purified by column chromatography on silica gel (hexane/$CHCl_3$ = 100:3) to afford the title compound as a colorless oil (52.0 mg, 83%).

^{1}H NMR (500 MHz, $CDCl_3$): δ 8.80 (dd, *J* = 7.5, 1.7 Hz, 1H), 8.72 (d, *J* = 8.0 Hz, 1H), 8.23 (dd, *J* = 7.5, 1.7 Hz, 1H), 8.03 (s, 1H), 7.95 (d, *J*= 6.9 Hz, 1H), 7.72–7.63 (m, 4H), 0.60 (s, 9H); ^{13}C NMR (126 MHz, $CDCl_3$): δ 136.6, 135.3, 134.9, 131.12, 130.97, 130.1, 128.9, 128.8, 127.1, 126.6, 126.2, 126.0, 123.3, 122.4, 0.25; HRMS (APCI+): *m*/*z* Calcd. for $C_{17}H_{18}Si$ $[M]^{+}$: 250.1172; found: 250.1164.

Dodecyldimethyl(phenanthrene-9-yl)silane (2c")

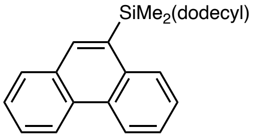

The general procedure was applied to neopentyl chloride (53.7 mg, 0.50 mmol), sodium dispersion (26.1 wt%, 94.0 mg, 1.07 mmol), 9-bromophenanthrene (**1c**, 64.2 mg, 0.25 mmol), and (dodecyl)Me_2SiCl (91 μL, 0.30 mmol). The crude product was purified by column chromatography on silica gel (hexane/$CHCl_3$ = 100:3) to afford the title compound as a colorless oil (85.7 mg, 85%).

^{1}H NMR (500 MHz, $CDCl_3$): δ 8.80 (dd, *J* = 8.0, 1.7 Hz, 1H) 8.72 (d, *J* = 8.6 Hz, 1H), 8.23 (dd, *J* = 7.4, 2.6 Hz, 1H), 8.03 (s, 1H), 7.95 (dd, *J* = 8.0, 1.4 Hz, 1H), 7.72–7.63 (m, 4H), 1.48–1.28 (m, 20H), 1.12–1.09 (m, 2H), 0.96 (t, *J* = 7.2 Hz, 3H), 0.58 (s, 6H); ^{13}C NMR (126 MHz, $CDCl_3$): δ 136.0, 135.6, 135.1, 131.1, 131.0, 130.0, 128.81, 128.79, 127.0, 126.5, 126.1, 125.9, 123.3, 122.4, 33.6, 31.9, 29.68, 29.68, 29.65, 19.6, 29.4, 29.3, 24.1, 22.7, 16.5, 14.1, –1.5; HRMS (APCI+): *m*/*z* Calcd. for $C_{28}H_{40}Si$ $[M]^{+}$: 404.2894; found: 404.2893.

Dimethyl(phenyl)(pyren-1-yl)silane (2d)

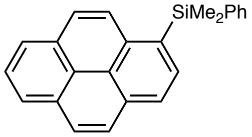

The general procedure was applied to neopentyl chloride (53.2 mg, 0.50 mmol), sodium dispersion (25.9 wt%, 94.0 mg, 1.06 mmol), 1-bromopyrene (**1d**, 70.2 mg, 0.25 mmol), and $PhMe_2SiCl$ (50 μL, 0.30 mmol). After quenching with H_2O, the organic materials were extracted with ethyl acetate for three times. The combined extracts were washed with brine and dried over Na_2SO_4. After the solvent was removed under reduced pressure, the crude product was purified by column chromatography on silica gel (hexane/chloroform = 10:1) to afford the title compound as a light yellow solid (77.1 mg, 92%).

Melting point: 157–158 °C; ^{1}H NMR (500 MHz, $CDCl_3$): δ 8.36 (d, *J* = 7.6 Hz, 1H), 8.33 (d, *J* = 9.2 Hz, 1H), 8.27–8.21 (m, 3H), 8.17–8.13 (m, 2H), 8.08–8.04 (m, 2H), 7.73–7.71 (m,

2H), 7.50–7.43 (m, 3H), 0.96 (s, 6H); ^{13}C NMR (126 MHz, $CDCl_3$): δ 139.1, 135.9, 134.2, 133.1, 132.9, 132.4, 131.2, 130.5, 129.1, 128.03, 128.01, 127.95, 127.5, 127.1, 125.8, 125.2, 125.1, 124.8, 124.7, 124.1, –0.5; GC-MS (EI) *m*/*z* (relative intensity): 338 (7), 337 (M^+, 19), 336 (75), 322 (30), 243 (29), 241 (23); HRMS (APCI+): *m*/*z* Calcd. for $C_{24}H_{19}Si$ $[M+H]^+$: 337.1407; found: 337.1402.

(4-Methoxyphenyl)dimethyl(phenyl)silane (2e)

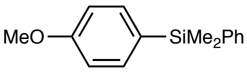

The general procedure was applied to neopentyl chloride (53.1 mg, 0.50 mmol), sodium dispersion (26.2 wt%, 92.2 mg, 1.05 mmol), 4-bromoanisole (**1e**, 49.1 mg, 0.26 mmol), and $PhMe_2SiCl$ (50 µL, 0.30 mmol). The exchange reaction was conducted at –40 °C for 10 min. The crude product was purified by column chromatography on silica gel (hexane/ethyl acetate = 50:1) to afford a mixture of the title compound and the disilylated product (**2e'**) as a colorless oil (59.8 mg, **2e**:**2e'** = 92:8, 90%). The spectral data were in accordance with those reported in the literature.[4] The minor product **2e'** was characterized by MS and ^{1}H NMR spectra.

(3-Methoxyphenyl)dimethyl(phenyl)silane (2f)

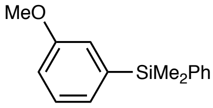

The general procedure was applied to neopentyl chloride (53.6 mg, 0.50 mmol), sodium dispersion (26.2 wt%, 91.2 mg, 1.04 mmol), 3-bromoanisole (**1f**, 45.4 mg, 0.24 mmol), and $PhMe_2SiCl$ (50 µL, 0.30 mmol). The exchange reaction was conducted at –40 °C for 10 min. The crude product was purified by column chromatography on silica gel (hexane/ethyl acetate = 100:1) to afford a mixture of the title compound and the disilylated product (**2f'**) as a colorless oil (56.1 mg, **2f**:**2f'** = 97:3, 94%). The spectral data were in accordance with those reported in the literature.[4] The minor product **2f'** was characterized by MS and ^{1}H NMR spectra.

(4-Chlorophenyl)dimethyl(phenyl)silane (2g)

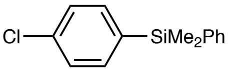

The general procedure was applied to neopentyl chloride (52.5 mg, 0.49 mmol), sodium

dispersion (26.2 wt%, 92.1 mg, 1.05 mmol), 4-bromochlorobenzene (**1g**, 49.6 mg, 0.26 mmol), and $PhMe_2SiCl$ (50 μL, 0.30 mmol). The exchange reaction was conducted at –40 °C for 10 min. The crude product was purified by column chromatography on silica gel (hexane/chloroform = 100:3) to afford the title compound as a colorless oil (54.6 mg, 85%). The spectral data were in accordance with those reported in the literature.[5]

(4-Fluorophenyl)dimethyl(phenyl)silane (2h)

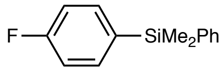

The general procedure was applied to neopentyl chloride (53.8 mg, 0.50 mmol), sodium dispersion (26.2 wt%, 92.3 mg, 1.05 mmol), 4-bromofluorobenzene (**1h**, 44.6 mg, 0.26 mmol) $PhMe_2SiCl$ (50 μL, 0.30 mmol). The exchange reaction was conducted at –40 °C for 10 min. The crude product was purified by column chromatography on silica gel (hexane/chloroform = 100:3) to afford the title compound as a light-yellow oil (50.5 mg, 86%). The spectral data were in accordance with those reported in the literature.[4]

Dimethyl(phenyl)(4-(trifluoromethyl)phenyl)silane (2i)

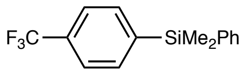

The general procedure was applied to neopentyl chloride (57.0 mg, 0.53 mmol), sodium dispersion (25.5 wt%, 101.0 mg, 1.12 mmol), 4-bromobenzotrifluoride (**1i**, 60.4 mg, 0.27 mmol), and $PhMe_2SiCl$ (54 μL, 0.32 mmol). The crude product was purified by column chromatography on silica gel (hexane) to afford the title compound as a colorless liquid (73.1 mg, 97%). The spectral data were in accordance with those reported in the literature.[5]

4-(Dimethyl(phenyl)silyl)phenol (2j)

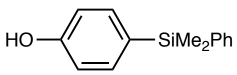

The general procedure was applied to hexane (2.5 mL), neopentyl chloride (79.2 mg, 0.74 mmol), sodium dispersion (25.7 wt%, 146.8 mg, 1.64 mmol), 4-bromophenol (**1j**, 43.5 mg, 0.25 mmol), and $PhMe_2SiCl$ (124 μL, 0.75 mmol). After reaction, quenching, extraction and filtration, the concentrated crude mixture was reacted with K_2CO_3 (175.2 mg, 1.27 mmol) in MeOH (3.0 mL) for 1 h. After diluting with H_2O, the organic materials were extracted with ethyl acetate for three times. The combined extracts were washed with brine and dried over Na_2SO_4. After the solvent was removed under reduced pressure, the crude product was

purified by column chromatography on silica gel (hexane/acetone = 10:1) to afford the title compound as a pale-yellow oil (52.2 mg, 91%). The spectral data were in accordance with those reported in the literature.[6]

(4-(Dimethyl(phenyl)silyl)phenyl)methanol (2k)

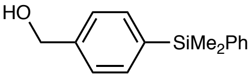

The general procedure was applied to hexane (2.5 mL), neopentyl chloride (79.0 mg, 0.75 mmol), sodium dispersion (26.0 wt%, 139.6 mg, 1.58 mmol), (4-bromophenyl)methanol (**1k**, 46.9 mg, 0.25 mmol), and $PhMe_2SiCl$ (124 μL, 0.75 mmol) After reaction, quenching, extraction and filtration, the concentrated crude mixture was reacted with K_2CO_3 (176.1 mg, 1.27 mmol) in MeOH (3.0 mL) for 1 h. After diluting with H_2O, the organic materials were extracted with ethyl acetate for three times. The combined extracts were washed with brine and dried over Na_2SO_4. After the solvent was removed under reduced pressure, the crude product was purified by column chromatography on silica gel (hexane/ethyl acetate = 7:1) to afford the title compound as a yellow oil (51.0 mg, 84%). The spectral data were in accordance with those reported in the literature.[7]

5-(Dimethyl(phenyl)silyl-1*H*-indole (2l)

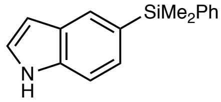

The general procedure was applied to hexane (2.5 mL), neopentyl chloride (79.1 mg, 0.74 mmol), sodium dispersion (25.7 wt%, 142.1 mg, 1.59 mmol), 5-bromoindole (**1l**, 49.1 mg, 0.25 mmol), and $PhMe_2SiCl$ (124 μL, 0.75 mmol). After reaction, quenching, extraction and filtration, the concentrated crude mixture was reacted with K_2CO_3 (176.1 mg, 1.27 mmol) in MeOH (3.0 mL) for 2 h. After diluting with H_2O, the organic materials were extracted with ethyl acetate for three times. The combined extracts were washed with brine and dried over Na_2SO_4. After the solvent was removed under reduced pressure, the crude product was purified by column chromatography on silica gel (hexane/ethyl acetate = 10:1), followed by GPC (chloroform) to afford the title compound as a colorless liquid (44.5 mg, 71%).

^{1}H NMR (500 MHz, $CDCl_3$): δ 7.97 (brs, 1H), 7.84 (s, 1H), 7.56–7.54 (m, 2H), 7.34–7.31 (m, 5H), 7.10 (t, J = 2.6 Hz, 1H), 6.52 (t, J = 2.6 Hz, 1H), 0.58 (s, 6H); ^{13}C NMR (126 MHz, $CDCl_3$): δ 139.3, 136.4, 134.2, 128.8, 127.73, 127.69, 127.6, 127.4, 127.3, 124.1, 110.8, 102.5, –1.9; GC-MS (EI) m/z (relative intensity): 251 (M^+, 27), 236 (100), 237 (20), 118 (15), 117

(10), 43 (9); HRMS (APCI+): *m/z* Calcd. for $C_{16}H_{17}NSi$ $[M+H]^+$: 252.1203; found: 252.1202.

2-(Dimethyl(phenyl)silyl)pyridine (2m)

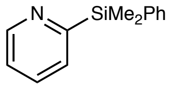

The general procedure was applied to neopentyl chloride (53.9 mg, 0.51 mmol), sodium dispersion (25.7 wt%, 96.6 mg, 1.08 mmol), 2-bromopyridine (**1m**, 41.5 mg, 0.26 mmol), and $PhMe_2SiCl$ (50 µL, 0.30 mmol). The exchange reaction was conducted at –78 °C for 30 min. The crude product was purified by column chromatography on silica gel (hexane/ethyl acetate = 15:1) to afford the title compound as a light-yellow liquid (30.8 mg, 55%). The spectral data were in accordance with those reported in the literature.[4]

8-(Dimethyl(phenyl)silyl)quinoline (2n)

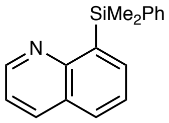

The general procedure was applied to neopentyl chloride (53.5 mg, 0.50 mmol), sodium dispersion (25.7 wt%, 93.3 mg, 1.04 mmol), 8-bromoquinoline (**1n**, 52.6 mg, 0.25 mmol), and $PhMe_2SiCl$ (50 µL, 0.30 mmol). After preparation of neopentylsodium, a THF (0.80 mL) solution of **1n** was added to reaction mixture at –78 °C. The exchange reaction was conducted at same temperature for 30 min. The crude product was purified by column chromatography (hexane/ethyl acetate = 50:1) to afford the title compound as a light yellow oil (50.1 mg, 75%).

^{1}H NMR (500 MHz, $CDCl_3$): δ 8.87 (dd, *J* = 4.2, 1.7 Hz, 1H), 8.07 (dd, *J* = 8.2, 1.7 Hz, 1H), 7.78 (dd, 7.6, 1.2 Hz, 1H), 7.70–7.68 (m, 3H), 7.43 (dd, *J* = 7.6, 7.6 Hz, 1H), 7.35–7.34 (m, 3H), 7.31 (dd, *J* = 8.2, 4.2 Hz, 1H), 0.76 (s, 6H); ^{13}C NMR (126 MHz, $CDCl_3$): δ 152.6, 149.0, 139.9, 139.5, 137.0, 135.9, 134.6, 129.3, 128.7, 127.7, 127.5, 125.9, 120.7, –1.4; GC-MS (EI) *m/z* (relative intensity): 263 (M^+, 5), 262 (12), 248 (100), 186 (56), 156 (53), 53 (41), 51 (58), 43 (38); HRMS (APCI+): *m/z* Calcd. for $C_{17}H_{17}NSi$ $[M+H]^+$: 264.1203; found: 264.1197.

Dimethyl(phenyl)(thiophen-2-yl)silane (2o)

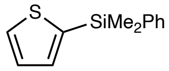

The general procedure was applied to neopentyl chloride (32.8 mg, 0.31 mmol), sodium dispersion (25.7 wt%, 55.0 mg, 0.61 mmol), 2-bromothiophene (**1o**, 41.1 mg, 0.25 mmol),

and $PhMe_2SiCl$ (58 µL, 0.35 mmol). The exchange reaction was conducted for 10 min. The crude product was purified by column chromatography on silica gel (hexane/chloroform = 100:3), followed by GPC (chloroform) to afford the title compound as a colorless liquid (40.3 mg, 73%). The spectral data were in accordance with those reported in the literature.[8]

1,4-Bis(dimethyl(phenyl)silyl)benzene (2p)

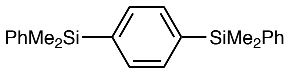

The general procedure was applied to neopentyl chloride (78.9 mg, 0.74 mmol), sodium dispersion (25.9 wt%, 138.5 mg, 1.56 mmol), 1,4-dibromobenzene (**1p**, 60.9 mg, 0.26 mmol), and $PhMe_2SiCl$ (124 µL, 0.75 mmol). The crude product was purified by column chromatography on silica gel (hexane/chloroform = 50:1) to afford the title compound as a colorless liquid (72.8 mg, 81%). The spectral data were in accordance with those reported in the literature.[9]

5,5-Dimethyl-5*H*-dibenzo[*b*,*d*]silole (2q)

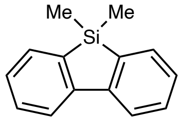

The general procedure was applied to neopentyl chloride (89.2 mg, 0.84 mmol), sodium dispersion (26.0 wt%, 157.6 mg, 1.78 mmol), 2,2'-dibromo-1,1'-biphenyl (**1q**, 79.0 mg, 0.25 mmol), and Me_2SiCl_2 (60 µL, 0.50 mmol). The crude product was purified by column chromatography on silica gel (hexane/chloroform = 100:3) to afford the title compound as a colorless oil (44.7 mg, 84%). The spectral data were in accordance with those reported in the literature.[10]

5,5-Diphenyl-5*H*-dibenzo[*b*,*d*]silole (2r)

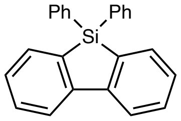

The general procedure was applied to neopentyl chloride (89.7 mg, 0.84 mmol), sodium dispersion (26.0 wt%, 158.7 mg, 1.79 mmol), 2,2'-dibromo-1,1'-biphenyl (**1q**, 79.1 mg, 0.25 mmol), and Ph_2SiCl_2 (104 µL, 0.50 mmol). The crude product was purified by column chromatography on silica gel (hexane/chloroform = 40:1) to afford the title compound as a colorless solid (73.4 mg, 87%). The spectral data were in accordance with those reported in the literature.[10]

- Reaction on 4.0 mmol scale

The general procedure was applied to hexane (35 mL), neopentyl chloride (1.67 mL, 13.6 mmol), sodium dispersion (26.0 wt%, 2.531 g, 28.6 mmol), 2,2'-dibromo-1,1'-biphenyl (**1q**, 1.25 g, 4.0 mmol), and Ph_2SiCl_2 (1.66 mL, 8.0 mmol). The preparation of neopentylsodium was conducted for 40 min. After quenching with H_2O, organic materials were extracted with ethyl acetate for three times. The combined extracts were washed with brine and dried over Na_2SO_4. After the solvent was removed under reduced pressure, the crude product was purified by column chromatography on silica gel (hexane/chloroform = 20:1) to afford the title compound as a colorless solid (1.18 g, 88%).

(2-Deuterioethene-1,1,2-triyl)tribenzene (2s)

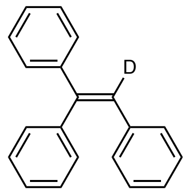

The general procedure was applied to neopentyl chloride (53.7 mg, 0.50 mmol), sodium dispersion (25.5 wt%, 92.3 mg, 1.02 mmol), 2-bromo-1,1,2-triphenylethylene (**1s**, 83.9 mg, 0.25 mmol), and D_2O (1.0 mL). The crude product was purified by column chromatography on silica gel (hexane/chloroform = 100:3) to afford the title compound as a colorless oil (58.2 mg, 90%). The deuterium content was determined to be 96% by ^{1}H NMR analysis (500 MHz, $CDCl_3$). The spectral data were in accordance with those reported in the literature.[11]

(*E*)-Dimethyl(phenyl)(styryl)silane (2t)

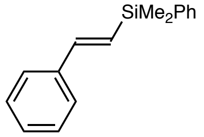

The general procedure was applied to neopentyl chloride (53.7 mg, 0.50 mmol), sodium dispersion (26.2 wt%, 95.4 mg, 1.09 mmol), (*E*)-(2-bromovinyl)benzene (**1t**, 46.5 mg, 0.25 mmol), and $PhMe_2SiCl$ (50 µL, 0.30 mmol). The exchange reaction was conducted for 10 min. The crude product was purified by column chromatography on silica gel (hexane) to afford the title compound as a colorless liquid (52.4 mg, 87%). The spectral data were in accordance with those reported in the literature.[12]

Dimethyl(phenyl)(styryl)silane (2t)

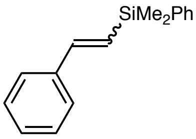

The general procedure was applied to neopentyl chloride (53.6 mg, 0.50 mmol), sodium dispersion (26.0 wt%, 91.6 mg, 1.04 mmol), (2-bromovinyl)benzene (**1t** (*E*/*Z* = 86:14), 45.0 mg, 0.25 mmol), and $PhMe_2SiCl$ (50 μL, 0.30 mmol). The exchange reaction was conducted for 10 min. The crude product was purified by column chromatography on silica gel (hexane) to afford the title compound as a colorless liquid (42.6 mg, 73%, (*E*/*Z* = 86:14)).[12]

1,3,5-Tris(4-(dimethyl(phenyl)silyl)phenyl)benzene (2u)

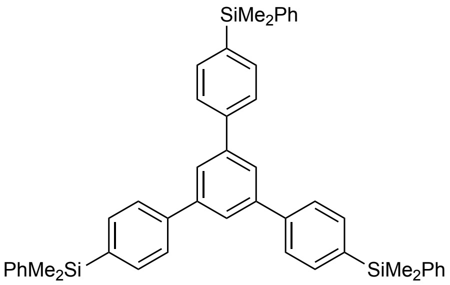

The general procedure was applied to hexane (3.0 mL), neopentyl chloride (119.9 mg, 1.12 mmol), sodium dispersion (26.0 wt%, 209.6 mg, 2.37 mmol), 1,3,5-tris(4-bromophenyl)benzene (**1u**, 135.9 mg, 0.25 mmol), and $PhMe_2SiCl$ (187 μL, 1.13 mmol). The electrophilic trapping reaction was conducted for 1 h. After quenching with H_2O, the organic materials were extracted with dichloromethane for three times. The combined extracts were dried over Na_2SO_4. After the solvent was removed under reduced pressure, the crude product was purified by column chromatography on silica gel (hexane/dichloromethane = 5:1), followed by GPC (chloroform) to afford the title compound as a colorless solid (113.3 mg, 64%).

Melting point: 128–129 °C; ^{1}H NMR (500 MHz, $CDCl_3$): δ 7.84 (s, 3H), 7.73–7.69 (m, 12H), 7.64–7.63 (m, 6H), 7.46–7.41 (m, 9H), 0.66 (s, 18H); ^{13}C NMR (126 MHz, $CDCl_3$): δ 142.2, 141.7, 138.1, 137.4, 134.8, 134.2, 129.2, 127.9, 126.7, 125.3, –2.3; HRMS (APCI+): *m*/*z* Calcd. for $C_{48}H_{48}Si_3$ $[M+H]^+$: 709.3137; found: 709.3140.

Tetrakis(4-(dimethyl(phenyl)silyl)phenyl)methane (2v)

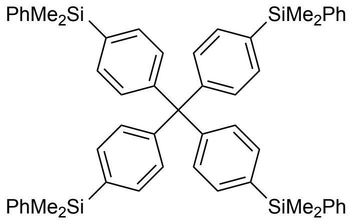

The general procedure was applied to hexane (3.0 mL), neopentyl chloride (126.8 mg, 1.19 mmol), sodium dispersion (26.0 wt%, 223.9 mg, 2.53 mmol), tetrakis(4-bromophenyl)methane (**1v**, 127.6 mg, 0.20 mmol), and $PhMe_2SiCl$ (199 μL, 1.20 mmol). The electrophilic trapping reaction was conducted for 1 h. After quenching with H_2O, the organic materials were extracted with dichloromethane for three times. The combined extracts were dried over Na_2SO_4. After the solvent was removed under reduced pressure, the crude product was purified by column chromatography on silica gel (hexane/dichloromethane = 5:1), followed by GPC (chloroform) to afford the title compound as a colorless solid (101.9 mg, 59%).

Melting point: 236–237 °C; ^{1}H NMR (500 MHz, $CDCl_3$): δ 7.60–7.59 (m, 8H), 7.44–7.38 (m, 20H), 7.22 (d, J = 8.0 Hz, 8H), 0.56 (s, 24H); ^{13}C NMR (126 MHz, $CDCl_3$): δ 147.2, 138.3, 135.4, 134.2, 133.2, 130.6, 129.0, 127.8, 64.9, –2.3; HRMS (APCI+): *m*/*z* Calcd. for $C_{57}H_{60}Si_4$ $[M]^+$: 856.3767; found: 856.3803.

General Procedure for the Pd-catalysed Negishi Cross-Coupling Reactions with Organosodium Compounds Prepared by Bromine–Sodium Exchange

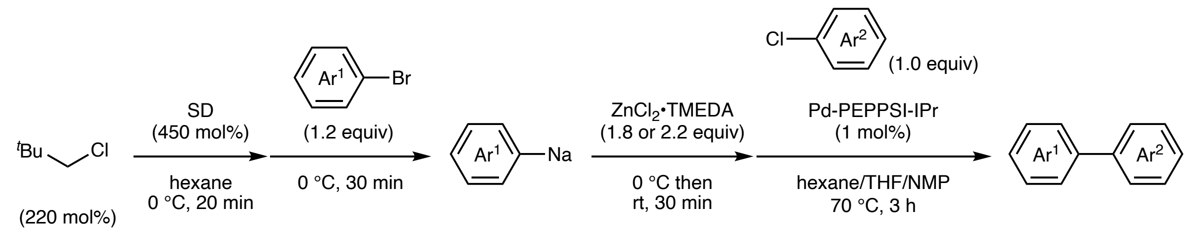

In a dry Schlenk tube equipped with a glass-coated stirring bar, neopentyl chloride (0.65 mmol, 220 mol%) was added to a mixture of hexane (2.0 mL) and sodium dispersion (ca. 26 wt%, 450 mol%) under nitrogen at 0 °C. After vigorously stirring at 0 °C for 20 min, the aryl bromide (0.36 mmol, 1.2 equiv) was added at 0 °C and the reaction mixture was vigorously stirred for 30 min to form the corresponding arylsodium compound. $ZnCl_2$•TMEDA (0.54 mmol, 1.8 equiv) was added at 0 °C and the reaction mixture was stirred at ambient temperature for 30 min to form the corresponding arylzinc compound. THF (0.80 mL), NMP (0.40 mL), Pd-PEPPSI-IPr (3.0 μmol, 1 mol%), and the aryl chloride (0.30 mmol, 1.0 equiv) were sequentially added and the reaction mixture was stirred at 70 °C for 3 h. The reaction was quenched with a saturated aqueous solution of NH_4Cl. After extraction with ethyl acetate for three times, the combined organic layers were passed through a pad of silica gel and concentrated under reduced pressure. The crude product was purified by column chromatography on silica gel using the indicated eluent to afford the desired compound.

Methyl 4-(naphthalen-1-yl)benzoate (4a)

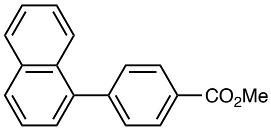

The general procedure was applied to neopentyl chloride (69.1 mg, 0.65 mmol), sodium dispersion (26.0 wt%, 120.6 mg, 1.36 mmol), 1-bromonaphthalene (**1a**, 74.6mg, 0.36 mmol), $ZnCl_2$•TMEDA (135.0 mg, 0.53 mmol), Pd-PEPPSI-IPr (2.1 mg, 3.1 μmol), and methyl 4-chlorobenzoate (51.9 mg, 0.30 mmol). The crude product was purified by column chromatography on silica gel (hexane/ethyl acetate = 15:1) to afford the title compound as a colorless viscous oil (72.7 mg, 91%). The spectral data were in accordance with those reported in the literature.[13]

***N*,*N*-Diphenyl-4’-(trifluoromethyl)-[1,1’-biphenyl]-4-amine (4b)**

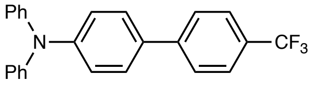

The general procedure was applied to neopentyl chloride (69.9 mg, 0.66 mmol), sodium dispersion (25.8 wt%, 123.1 mg, 1.38 mmol), 4-bromotriphenylamine (117.9 mg, 0.36 mmol), $ZnCl_2$•TMEDA (138.3 mg, 0.55 mmol), Pd-PEPPSI-IPr (2.1 mg, 3.1 μmol), and 4-chlorobenzotrifluoride (54.5 mg, 0.30 mmol). The crude product was purified by column chromatography on silica gel (hexane/ethyl acetate = 100:1) to afford the title compound as a pale-yellow solid (112.8 mg, 96%).

Melting point; 102–103 °C; ^{1}H NMR (500 MHz, $CDCl_3$): δ 7.69 (brm, 4H), 7.51 (d, J = 8.6 Hz, 2H), 7.32 (dd, J = 8.0, 8.0 Hz, 4H), 7.20–7.18 (m, 6H), 7.10 (dd, J = 7.5, 7.5 Hz, 2H); ^{13}C NMR (126 MHz, $CDCl_3$): δ 148.1, 147.4, 144.1, 133.1, 129.4, 129.1, 128.7 (q, J_{CF} = 32 Hz), 127.9, 126.7, 125.7 (q, J_{CF} = 4 Hz), 124.7, 123.4 (q, J_{CF} = 187 Hz), 123.3 (q, J_{CF} = 4 Hz); ^{19}F NMR (470 MHz, $CDCl_3$): δ –65.5 (s); GC-MS (EI) m/z (relative intensity): 390 (26), 389 (M^+, 100), 388 (18), 184 (12), 77 (9); HRMS (APCI+): m/z Calcd. for $C_{25}H_{18}F_3N$ $[M]^+$: 389.1386; found: 389.1374.

(3’,5’-Di-*tert*-butyl-[1,1’-biphenyl]-2-yl)(phenyl)methanone (4c)

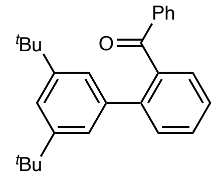

The general procedure was applied to neopentyl chloride (69.0 mg, 0.65 mmol), sodium dispersion (25.8 wt%, 122.5 mg, 1.37 mmol), 1-bromo-3,5-di-*tert*-butylbenzene (97.3 mg, 0.36 mmol), $ZnCl_2$•TMEDA (136.9 mg, 0.54 mmol), Pd-PEPPSI-IPr (2.3 mg, 3.4 μmol), and 2-chlorobenzophenone (65.5 mg, 0.30 mmol). The crude product was purified by column chromatography on silica gel (hexane/ethyl acetate = 80:1) to afford the title compound as a colorless viscous oil (107.8 mg, 96%).

^{1}H NMR (500 MHz, $CDCl_3$): δ 7.58–7.50 (m, 5H), 7.46 (ddd, J = 7.5, 7.5, 1.8 Hz, 1H), 7.28 (dd, J = 7.5, 7.5 Hz, 1H), 7.16–7.11 (m, 3H), 7.04 (d, J = 2.3 Hz, 2H), 1.18 (s, 18H); ^{13}C NMR (126 MHz, $CDCl_3$): δ 199.1, 150.4, 142.2, 139.34, 139.30, 137.2, 132.4, 130.2, 129.7, 129.5, 128.6, 127.6, 127.0, 123.6, 121.0, 34.7, 31.2; GC-MS (EI) m/z (relative intensity): 371 (M^+, 12), 370 (46), 300 (20), 299 (94), 105 (100), 77 (43), 355 (43), 57 (97), 41 (48); HRMS (APCI+): m/z Calcd. for $C_{27}H_{30}O$ $[M+H]^+$: 371.2369; found: 371.2376.

2-Methyl-5-(naphthalen-2-yl)thiophene (4d)

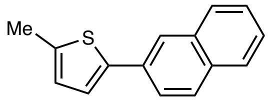

The general procedure was applied to neopentyl chloride (68.6 mg, 0.64 mmol), sodium dispersion (25.8 wt%, 124.0 mg, 1.39 mmol), 2-bromo-5-methylthiophene (64.4 mg, 0.36 mmol), $ZnCl_2$•TMEDA (138.9 mg, 0.55 mmol), Pd-PEPPSI-IPr (2.1 mg, 3.1 μmol), and 2-chloronaphthalene (48.9 mg, 0.30 mmol). The crude product was purified by column chromatography on silica gel (hexane/chloroform = 100:3) to afford the title compound as a colorless solid (64.4 mg, 95%). The spectral data were in accordance with those reported in the literature.[14]

2-(4-(Trifluoromethyl)phenyl)pyridine (4e)

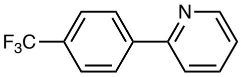

The general procedure was applied to neopentyl chloride (69.0 mg, 0.65 mmol), sodium dispersion (26.0 wt%, 120.2 mg, 1.36 mmol), 4-bromobenzotrifluoride (**1i**, 81.3 mg, 0.36 mmol), $ZnCl_2$•TMEDA (163.8 mg, 0.65 mmol), Pd-PEPPSI-IPr (2.0 mg, 2.9 μmol), and 2-chloropyridine (34.8 mg, 0.31 mmol). The crude product was purified by column chromatography on silica gel (hexane/ethyl acetate = 10:1) to afford the title compound as a colorless solid (60.3 mg, 88%). The spectral data were in accordance with those reported in the literature.[15]

2-(4-(Trifluoromethyl)phenyl)naphthalene (4f)

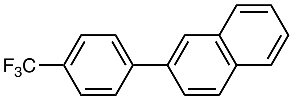

The general procedure was applied to neopentyl chloride (68.9 mg, 0.65 mmol), sodium dispersion (26.0 wt%, 121.0 mg, 1.37 mmol), 4-bromobenzotrifluoride (**1i**, 80.9 mg, 0.36 mmol), $ZnCl_2$•TMEDA (137.3 mg, 0.54 mmol), Pd-PEPPSI-IPr (2.0 mg, 2.9 μmol), and 2-chloronaphthalene (49.2 mg, 0.30 mmol). The crude product was purified by column chromatography on silica gel (hexane/chloroform = 100:3) to afford the title compound as a colorless solid (74.8 mg, 91%). The spectral data were in accordance with those reported in the literature.[16]

General Procedure for the Pd-Catalysed Suzuki–Miyaura Cross-Coupling Reactions with Organosodium Compounds Prepared by Bromine–Sodium Exchange

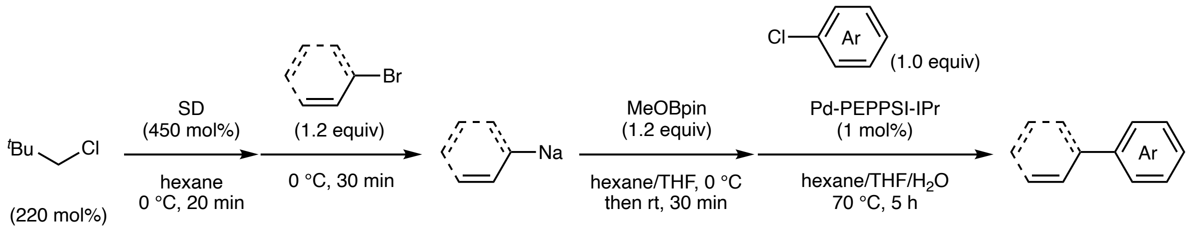

In a dry Schlenk tube equipped with a glass-coated stirring bar, neopentyl chloride (0.65 mmol, 220 mol%) was added to a mixture of hexane (2.0 mL) and sodium dispersion (ca. 26 wt%, 450 mol%) under nitrogen at 0 °C. After vigorously stirring at 0 °C for 20 min, the aryl bromide (0.36 mmol, 1.2 equiv) was added at 0 °C and the reaction mixture was vigorously stirred for 30 min to form the corresponding arylsodium compound. MeOBpin (0.36 mmol, 1.2 equiv) and THF (0.80 mL) were added at 0 °C and the reaction mixture was vigorously stirred at for 30 min to form the corresponding arylboron compound, followed by addition of H_2O (0.40 mL) at the same temperature. Pd-PEPPSI-IPr (3.0 μmol, 1 mol%) and the aryl halide (0.30 mmol, 1.0 equiv) were sequentially added and the reaction mixture was stirred at 70 °C for 5 h. The reaction was quenched with a saturated aqueous solution of NH_4Cl. After extraction with ethyl acetate for three times, the combined organic layers were passed through a pad of silica gel and concentrated under reduced pressure. The crude product was purified by column chromatography on silica gel using the indicated eluent to afford the desired compound.

4-(Naphthalen-2-yl)benzonitrile (5a)

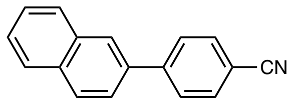

The general procedure was applied to neopentyl chloride (69.0 mg, 0.65 mmol), sodium dispersion (25.8 wt%,124.1 mg, 1.39 mmol), 2-bromonaphthalene (**1b**, 74.9 mg, 0.36 mmol), MeOBpin (58.2 mg, 0.37 mmol), Pd-PEPPI-IPr (2.0 mg, 2.9 μmol), and 4-chlorobenzonitrile (42.2 mg, 0.31 mmol). The crude product was purified by column chromatography on silica gel (hexane/chloroform/ethyl acetate = 20:2:1) to afford the title compound as a colorless solid (65.2 mg, 93%). The spectral data were in accordance with those reported in the literature.[17]

4-Fluoro-4'-methoxy-1,1'-biphenyl (5b)

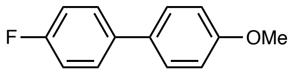

The general procedure was applied to neopentyl chloride (80.3 mg, 0.75 mmol), sodium dispersion (26.0 wt%, 139.8 mg, 1.58 mmol), 4-bromofluorobenzene (**1h**, 73.8 mg, 0.42 mmol), exchange reaction at –40 °C, MeOBpin (67.2 mg, 0.43 mmol), transmetalation reaction at –40 °C, Pd-PEPPSI-IPr (2.2 mg, 3.2 μmol), and 4-chloroanisole (42.7 mg, 0.30 mmol). The crude product was purified by column chromatography on silica gel (hexane/ethyl acetate = 30:1) to afford the title compound as a colorless solid (56.4 mg, 93%). The spectral data were in accordance with those reported in the literature.[18]

4'-(9*H*-Carbazol-9-yl)-[1,1'-biphenyl]-2-carbaldehyde (5c)

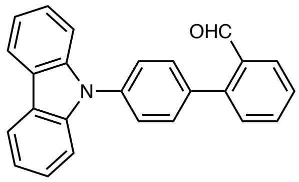

The general procedure was applied to neopentyl chloride (69.7 mg, 0.65 mmol), sodium dispersion (25.7 wt%, 124.0 mg, 1.39 mmol), 9-(4-bromophenyl)carbazole (116.5 mg, 0.36 mmol), MeOBpin (57.9 mg, 0.37 mmol), Pd-PEPPSI-IPr (2.2 mg, 3.2 μmol), and 2-chlorobenzaldehyde (42.1 mg, 0.30 mmol). The crude product was purified by column chromatography on silica gel (hexane/ethyl acetate = 20:1) to afford the title compound as a pale yellow solid (90.9 mg, 87%).

Melting point; 177–178 °C; ^{1}H NMR (500 MHz, $CDCl_3$): δ 10.13 (s, 1H), 8.13 (d, J = 7.5 Hz, 2H), 8.06 (d, J = 7.5 Hz, 1H), 7.67–7.62 (m, 3H), 7.56–7.47 (m, 6H), 7.41 (ddd, J = 7.7, 7.7, 1.2 Hz, 2H), 7.29 (dd, J = 7.5, 7.5 Hz, 2H); ^{13}C NMR (126 MHz, $CDCl_3$): δ 192.0, 144.8, 140.5, 137.7, 136.6, 133.67, 133.67, 131.4, 130.7, 128.1, 127.8, 126.7, 126.0, 123.5, 120.3, 120.2, 109.7; GC-MS (EI) *m*/*z* (relative intensity): 348 (31), 347 (M^+, 100), 319 (13), 318 (16), 167 (14), 153 (17), 152 (27); HRMS (APCI+): *m*/*z* Calcd. for $C_{25}H_{17}NO$ $[M]^+$: 347.1305; found: 347.1304.

2-(Quinolin-8-yl)-4-(trifluoromethoxy)aniline (5d)

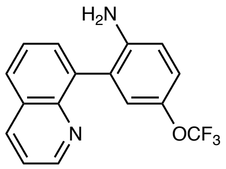

The general procedure was applied to neopentyl chloride (80.7 mg, 0.76 mmol) and sodium

dispersion (26.0 wt%, 140.5 mg, 1.59 mmol), THF (0.80 mL) solution of 8-bromoquinoline (**1n**, 87.9 mg, 0.42 mmol) was added at –78 °C and exchange reaction at same temperature, MeOBpin (67.0 mg, 0.42 mmol), H_2O (0.40 mL), Pd-PEPPSI-IPr (2.3 mg, 3.4 μmol), and 2-chloro-4-trifluoromethoxyaniline (44 μL, 0.30 mmol). The crude product was purified by column chromatography on silica gel (hexane/ethyl acetate = 2:1) to afford the title compound as a yellow oil (74.3 mg, 81%).

^{1}H NMR (500 MHz, $CDCl_3$): δ 8.94 (dd, J = 4.4, 1.7 Hz, 1H), 8.22 (dd, J = 8.4, 1.7 Hz, 1H), 7.87 (dd, J = 8.0, 1.6 Hz, 1H), 7.68 (dd, J = 7.0, 1.6 Hz, 1H), 7.61 (dd, J = 8.0, 7.0 Hz, 1H), 7.42 (dd, J = 8.4, 4.4 Hz, 1H), 7.12–7.08 (m, 2H), 6.78 (d, J = 8.6 Hz, 1H), 3.84 (s, 2H); ^{13}C NMR (126 MHz, $CDCl_3$): δ 150.7, 145.8, 143.7, 141.3, 137.9, 136.8, 131.8, 128.6, 128.4, 127.5, 126.7, 124.5, 121.6, 121.2, 120.7 (q, J_{CF} = 256 Hz) 116.7; ^{19}F NMR (470 MHz, $CDCl_3$): δ –61.3 (s); GC-MS (EI) m/z (relative intensity): 304 (M^+, 63), 303 (100), 302 (14), 288 (69), 191 (47), 69 (74), 44 (50); HRMS (APCI+): m/z Calcd. for $C_{16}H_{11}F_3N_2O$ $[M+H]^+$: 305.0896; found: 305.0899.

(*E*)-1-Methoxy-4-nitro-2-styrylbenzene (5e)

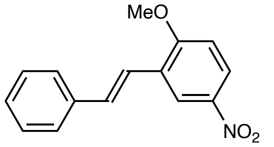

The general procedure was applied to neopentyl chloride (68.4 mg, 0.64 mmol), sodium dispersion (25.7 wt%, 120.1 mg, 1.34 mmol), (*E*)-(2-bromovinyl)benzene (**1t**, 67.4 mg, 0.37 mmol), MeOBpin (57.9 mg, 0.37 mmol), Pd-PEPPSI-IPr (4.2 mg, 6.2 μmol), and 2-chloro-4-nitroanisole (56.4 mg, 0.30 mmol). The crude product was purified by column chromatography on silica gel (hexane/chloroform/ethyl acetate = 50:10:1) to afford the title compound as a yellow solid (54.0 mg, 70%). The spectral data were in accordance with those reported in the literature.[19]

2,2'-(9,9-Dioctyl-9*H*-fluorene-2,7-diyl)dithiophene (5f)

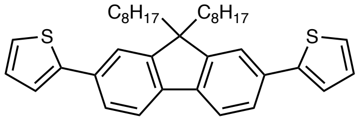

The general procedure was applied to hexane (2.5 mL), neopentyl chloride (96.2 mg, 0.90 mmol), sodium dispersion (25.7 wt%, 171.6 mg, 1.92 mmol), 2-bromothiophene (**1o**, 129.8 mg, 0.80 mmol), exchange reaction for 10 min, MeOBpin (136.6 mg, 0.86 mmol), THF (1.0

mL), H_2O (0.50 mL), Pd-PEPPSI-IPr (4.2 mg, 6.2 μmol), and 9,9-dioctyl-2,7-dibromofluorene (165.0 mg, 0.30 mmol). The crude product was purified by column chromatography on silica gel (hexane/chloroform = 100:3) to afford the title compound as a yellow oil (136.8 mg, 82%). The spectral data were in accordance with those reported in the literature.[20]

General Procedure for the Pd-Catalysed Direct Cross-Coupling Reaction with Organosodium Compounds Prepared by Bromine–Sodium Exchange

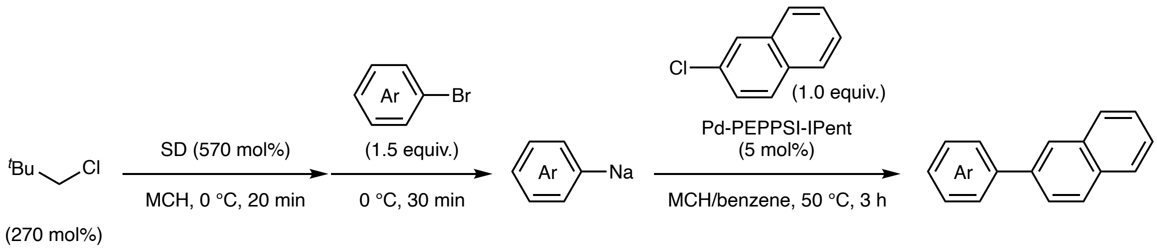

In a dry Schlenk tube equipped with a glass-coated stirring bar, neopentyl chloride (0.81 mmol, 270 mol%) was added to a mixture of methylcyclohexane (MCH, 2.0 mL) and sodium dispersion (ca. 26 wt%, 1.70 mmol, 570 mol%) under nitrogen at 0 °C. After vigorously stirring at 0 °C for 20 min, the aryl bromide (0.45 mmol, 1.5 equiv) was added at 0 °C and the reaction mixture was vigorously stirred for 30 min to form the corresponding arylsodium compound. Pd-PEPPIS-IPent (15 μmol, 5 mol%), 2-chloronaphthalene (0.30 mmol, 1.0 equiv), and benzene (1.0 mL) were sequentially added at 0 °C and the reaction mixture was stirred at 50 °C for 3 h. The reaction was quenched with a saturated aqueous solution of NH_4Cl. After extraction with ethyl acetate for three times, the combined organic layers were passed through a pad of silica gel and concentrated under reduced pressure. The crude product was purified by column chromatography on silica gel using the indicated eluent to afford the desired compound.

9-(Naphthalen-2-yl)phenanthrene (6a)

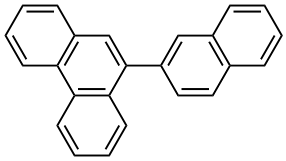

The general procedure was applied to neopentyl chloride (86.4 mg, 0.81 mmol), sodium dispersion (26.1 wt%, 149.6 mg, 1.70 mmol), 9-bromophenanthrene (**1c**, 116.0 mg, 0.45 mmol), Pd-PEPPSI-IPent (12.1 mg, 15.3 μmol), and 2-chloronapthalene (48.9 mg, 0.30 mmol). The crude product was purified by column chromatography (hexane/chloroform = 100:3 to 25:1) and GPC (chloroform) to afford the title compound as a colorless solid (70.1 mg, 77%). The spectral data were in accordance with those reported in literature.[21]

- Control Experiment –

The direct cross-coupling of organosodium compound in the absence of the Pd catalyst still afforded coupled product **6a** but in a much lower yield (Supplementary Figure 5, entry 1).

This reaction seems to proceed via *ortho*-deprotonation of the aryl chloride and the subsequent benzyne generation. While Pd[P(^{t}Bu)$_3$]$_2$ did not work (entry 2), Pd-PEPPSI-IPent clearly promoted the cross-coupling reaction (entry 3).

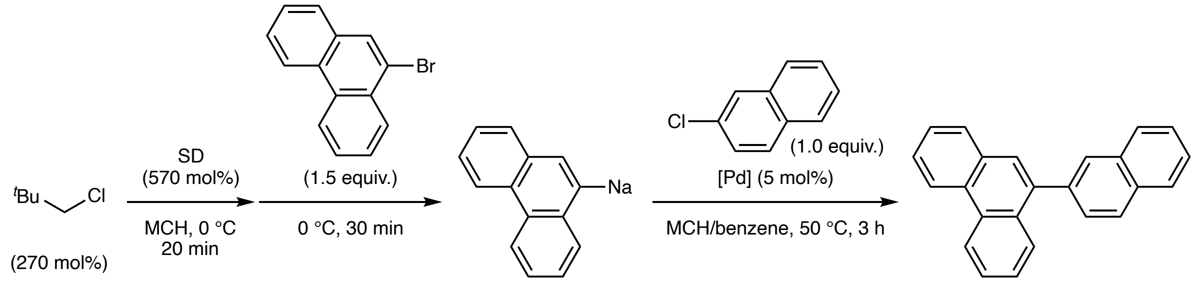

6a

entry	[Pd]	**6a** (%)[a]
1	none	18
2	Pd[(^{t}Bu)$_3$P]$_2$	17
3	Pd-PEPPSI-IPent	79

Supplementary Figure 5. Control experiments of Pd-catalysed direct cross-coupling of arylsodium. [a]Yields were determined by GC measurement using tridecane as an internal standard.

1-(Naphthalen-2-yl)pyrene (6b)

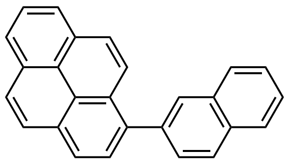

The general procedure was applied to neopentyl chloride (86.0 mg, 0.81 mmol), sodium dispersion (26.1 wt%, 152.6 mg, 1.73 mmol), 1-bromopyrene (**1d**, 127.0 mg, 0.45 mmol), Pd-PEPPSI-IPent (11.9 mg, 15.0 μmol), and 2-chloronapthalene (48.9 mg, 0.30 mmol). The crude product was purified by column chromatography (hexane/chloroform = 20:1) to afford the title compound as a pale yellow solid (62.1 mg, 63%). The spectral data were in accordance with those reported in literature.[22]

2-(4-(*tert*-Butyl)phenyl)naphthalene (6c)

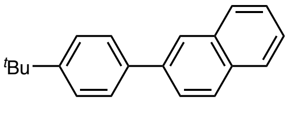

The general procedure was applied to neopentyl chloride (85.6 mg, 0.80 mmol), sodium dispersion (26.1 wt%, 151.0 mg, 1.71 mmol), 1-bromo-4-*tert*-butylbenzene (98.3 mg, 0.46 mmol), Pd-PEPPSI-IPent (11.8 mg, 14.9 μmol), and 2-chloronapthalene (48.9 mg, 0.30

mmol). The crude product was purified by column chromatography (hexane/chloroform = 100:3) to afford the title compound as a colorless solid (52.6 mg, 67%). The spectral data were in accordance with those reported in literature.[23]

2-(2-(Trifluoromethyl)phenyl)naphthalene (6d)

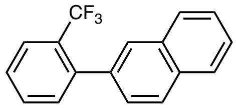

The general procedure was applied to neopentyl chloride (86.6 mg, 0.81 mmol), sodium dispersion (26.1 wt%, 148.5 mg, 1.69 mmol), 2-bromobenzotrifluoride (103.4 mg, 0.46 mmol), Pd-PEPPSI-IPent (11.9 mg, 15.0 μmol), and 2-chloronapthalene (48.9 mg, 0.30 mmol). The crude product was purified by column chromatography (hexane/chloroform = 100:3) to afford the title compound as a light yellow oil (60.9 mg, 74%). The spectral data were in accordance with those reported in literature.[24]

2-Methyl-5-(naphthalen-2-yl)thiophene (6e)

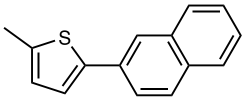

The general procedure was applied to neopentyl chloride (87.8 mg, 0.82 mmol), sodium dispersion (26.1 wt%, 152.3 mg, 1.73 mmol), 2-bromo-5-methylthiophene (81.3 mg, 0.46 mmol), Pd-PEPPSI-IPent (12.1 mg, 15.3 μmol), and 2-chloronapthalene (49.0 mg, 0.30 mmol). The coupling reaction was conducted for 1 h. The crude product was purified by column chromatography (hexane/chloroform = 100:3) to afford the title compound as a colorless solid (35.7 mg, 53%). The spectral data were in accordance with those reported in literature.[14]

2. Supplementary References

(1) Still, W. C.; Kahn, M.; Mitra, A. Rapid chromatographic technique for preparative separations with moderate resolution. *J. Org. Chem.* **1978**, *43*, 2923–2925.

(2) Bull, J. A.; Mousseau, J. J.; Charette, A. B. Convenient one-pot synthesis of (*E*)-*β*-aryl vinyl halides from benzyl bromides and dihalomethanes. *Org. Lett.* **2008**, *10*, 5485–5488.

(3) Isobe, M.; Kondo, S.; Nagasawa, N.; Goto, T. Trialkylzinclithium [R_3ZnLi] a new reagent for conjugate addition to *α,β*-unsaturated ketones. *Chem. Lett.* **1997**, *6*, 679–682.

(4) Kong, Y.-Y.; Wang, Z.-X. Nickel-catalyzed reaction of aryl 2-pyridyl ethers with silylzinc chlorides: Silylation of aryl 2-pyridyl ethers via cleavage of the carbon–oxygen bond. *Adv. Synth. Catal.* **2019**, *361*, 5440–5448.

(5) Jang, J.; Byun, S.; Kim, B. M.; Lee, S. Arylsilylation of aryl halides using the magnetically recyclable bimetallic Pd–Pt–Fe_3O_4 catalyst. *Chem. Commun.* **2018**, *54*, 3492–3495.

(6) Fujii, S.; Miyajima, Y.; Masuno, H.; Kagechika, H. Increased hydrophobicity and estrogenic activity of simple phenols with silicon and germanium-containing substituents. *J. Med. Chem.* **2013**, *56*, 160–166.

(7) Guo, H.; Chen, X.; Zhao, C.; He, W. Suzuki-type cross coupling between aryl halides and silylboranes for the syntheses of aryl silanes. *Chem. Commun.* **2015**, *51*, 17410–17412.

(8) Hu, S.-S.; Weber, W. P. Photolysis of 1,1,2,2-tetramethyl-1,2-bis-(2’-thienyl)disilane. *J. Organomet. Chem.* **1989**, *369*, 155–163.

(9) Mallick, S.; Xu, P.; Würthwein, E.-U.; Studer, A. Silyldefluorination of fluoroarenes by concerted nucleophilic aromatic substitution. *Angew. Chem., Int. Ed.* **2019**, *58*, 283–287.

(10) Omann, L.; Oestreich, M. A catalytic S_EAr approach to dibenzosiloles functionalized at both benzene cores. *Angew. Chem., Int. Ed.* **2015**, *54*, 10276–10279.

(11) Xue, F.; Zhao, J.; Hor, T. S. A. Ambient arylmagnesiation of alkynes catalysed by ligandless nickle(II). *Chem. Commun.* **2013**, *49*, 10121–10123.

(12) Itami, K.; Nokami, T.; Yoshida, J. [Bis(2-pyridyldimethylsilyl)methyl]lithium. New reagent for the stereoselective synthesis of vinylsilanes. *Org. Lett.* **2000**, *2*, 1299–1302.

(13) Klein, P.; Lechner, V. D.; Schimmel, T.; Hintermann, L. Generation of organozinc reagents by nickel diazadiene complex catalyzed zinc insertion into aryl sulfonates. *Chem. Eur. J.* **2020**, *26*, 176–180.

(14) Zhang, Y.; Zhao, H.; Zhang, M.; Su, W. Carboxylic acids as traceless directing groups for the rhodium(III)-catalyzed decarboxylative C–H arylation of thiophenes. *Angew. Chem., Int. Ed.* **2015**, *54*, 3817–3821.

(15) Martinez-Solorio, D.; Melillo, B.; Sanchez, L.; Liang, Y.; Lam, E.; Houk, K. N.; Smith, A. B., III Design, synthesis, and validation of an effective, reusable silicon-based transfer agent for room-temperature Pd-catalyzed cross-coupling reactions of aryl and heteroaryl chlorides with readily available aryl lithium reagents. *J. Am. Chem. Soc.* **2016**, *138*, 1836–1839.

(16) Schwarzer, M. C.; Konno, R.; Hojo, T.; Ohtsuki, A.; Nakamura, K.; Yasutome, A.; Takahashi, H.; Shimasaki, T.; Tobisu, M.; Chatani, N.; Mori, S. Combined theoretical and experimental studies of nickel-catalyzed cross-coupling of methoxyarenes with arylboronic esters via C–O bond cleavage. *J. Am. Chem. Soc.* **2017**, *139*, 10347–10358.

(17) Hua, X.; Masson-Makdissi, J.; Sullivan, R. J.; Newman, S. G. Inherent vs apparent chemoselectivity in the Kumada–Corriu cross-coupling reaction. *Org. Lett.* **2016**, *18*, 5312–5315.

(18) Asghar, S.; Tailor, S. B.; Elorriaga, D.; Bedford, R. B. Cobalt-catalyzed Suzuki biaryl coupling of aryl halides. *Angew. Chem., Int. Ed.* **2017**, *56*, 16367–16370.

(19) Joncour, R.; Susperregui, N.; Pinaud, N.; Miqueu K.; Fouquet, E.; Sotiropoulos, J.-M.; Felpin, F.-X. Chelation-assisted cross-coupling of anilines through in situ activation as diazonium salts with boronic acids under ligand-, base-, and salt-free conditions. *Chem. Eur. J.* **2013**, *19*, 9291–9296.

(20) Kim, J.-H.; Kim, H. U.; Mi, D.; Jin, S.-H.; Shin, W. S.; Yoon, S. C.; Kang, I.-N.; Hwang, D.-H. Introduction of perylene units for enhanced interchain interaction in conjugated polymers for organic photovoltaic devices. *Macromolecules* **2012**, *45*, 2367–2376.

(21) Morioka, T.; Nishizawa, A.; Furukawa, T.; Tobisu, M.; Chatani, N. Nickel-mediated decarbonylation of simple unstrained ketones through the cleavage of carbon–carbon bonds. *J. Am. Chem. Soc.* **2017**, *139*, 1416–1419.

(22) Lee, W.; Kang, Y.; Lee, P. H. Synthesis and characterization of polyaromatic compounds using tri(napththyl)indium. *J. Org. Chem.* **2008**, *73*, 4326–4329.

(23) Yang, Z.-K.; Wang, D.-Y.; Minami, H.; Ogawa, H.; Ozaki, T.; Saito, T.; Miyamoto, K.; Wang, C.; Uchiyama, M. Cross-coupling of organolithium with ethers or aryl ammounium salts by C–O or C–N bond cleavage. *Chem. Eur. J.* **2016**, *22*, 15693–15699.

(24) Vila, C.; Cembellín, S.; Hornillos, V.; Giannerini, M.; Fañanás-Mastral, M.; Feringa, B. L. *t*BuLi-mediated one-pot direct highly selective cross-coupling of two distinct aryl bromides. *Chem. Eur. J.* **2015**, *21*, 15520–15524.

3. Supplementary Figures

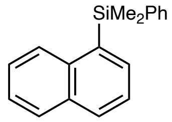

2a (^{1}H NMR, 500 MHz, $CDCl_3$)

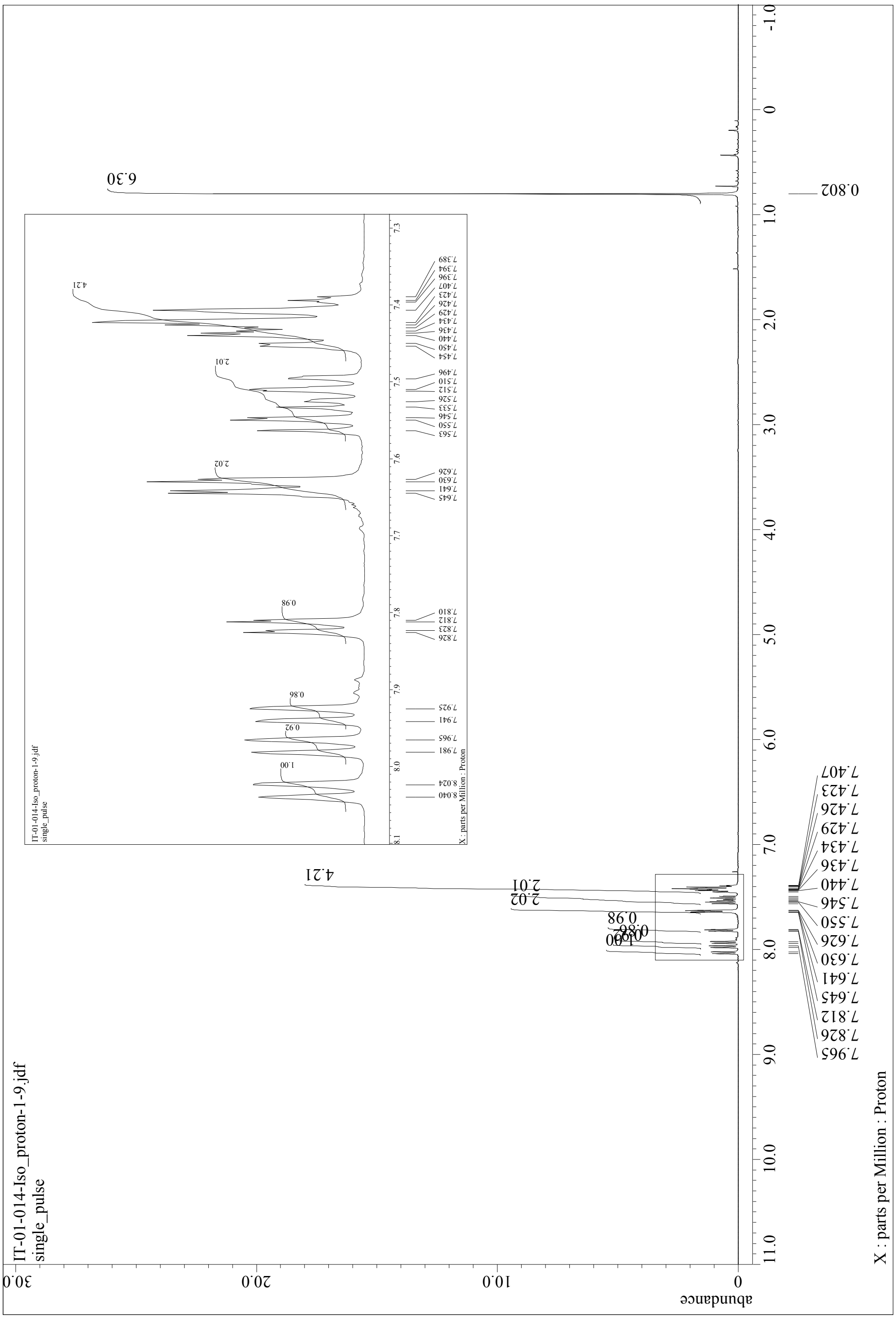

Supplementary Figure 6. ^{1}H NMR spectrum of **2a**

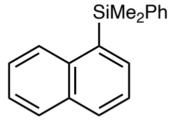

2a (^{13}C NMR, 126 MHz, $CDCl_3$)

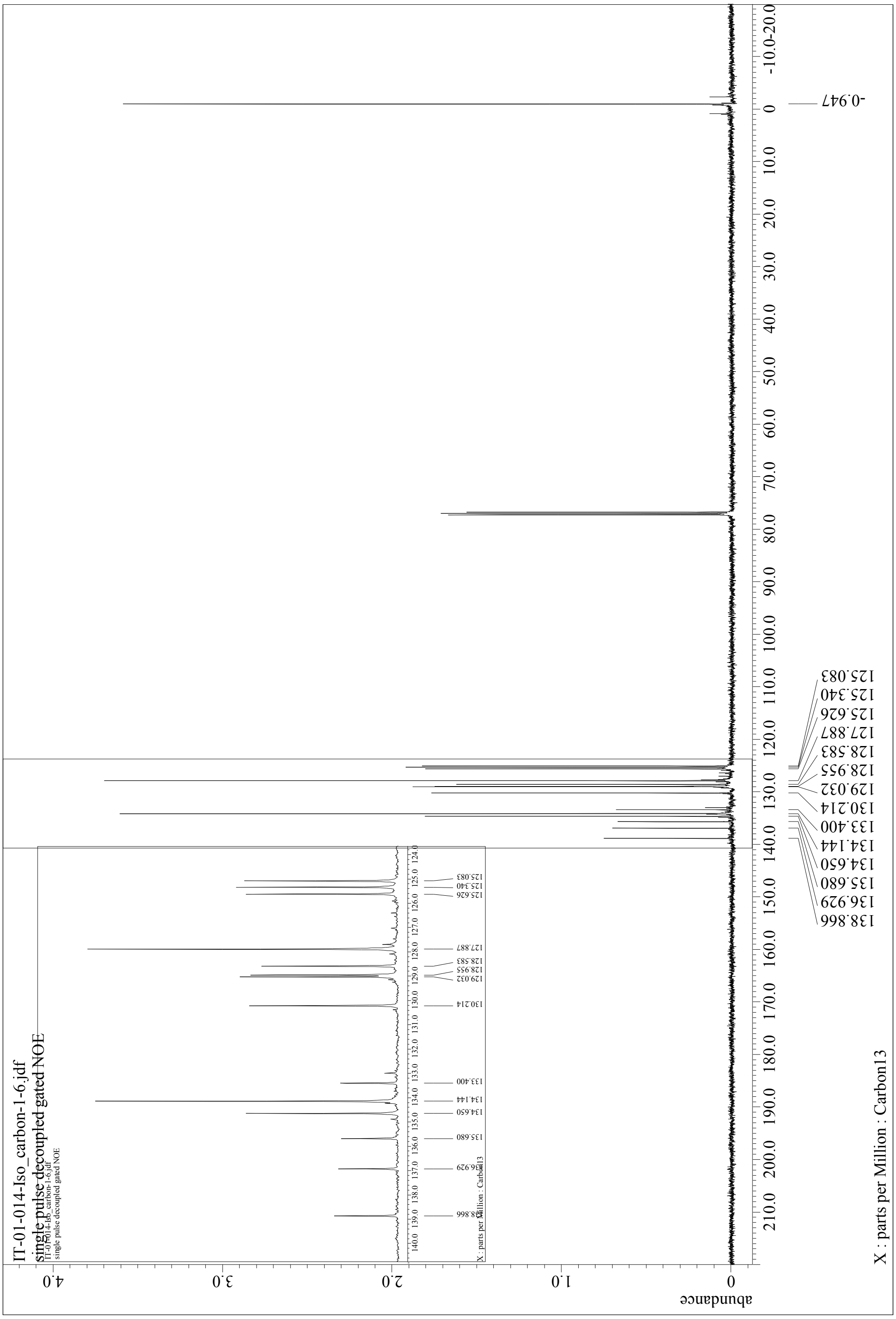

Supplementary Figure 7. ^{13}C NMR spectrum of **2a**

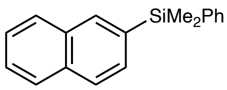

2b (^{1}H NMR, 500 MHz, $CDCl_3$)

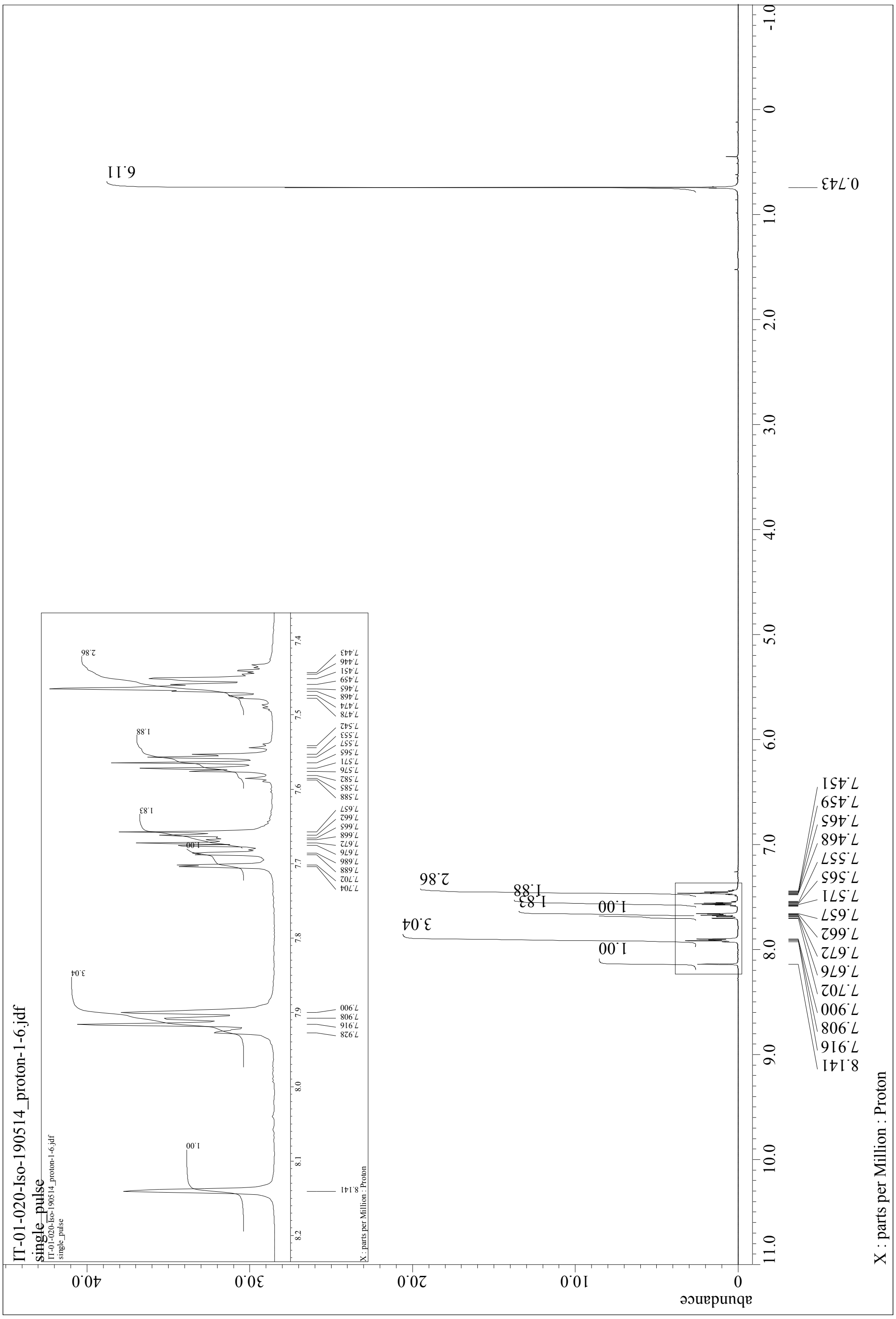

Supplementary Figure 8. ^{1}H NMR spectrum of **2b**

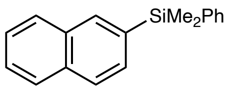

2b (^{13}C NMR, 126 MHz, $CDCl_3$)

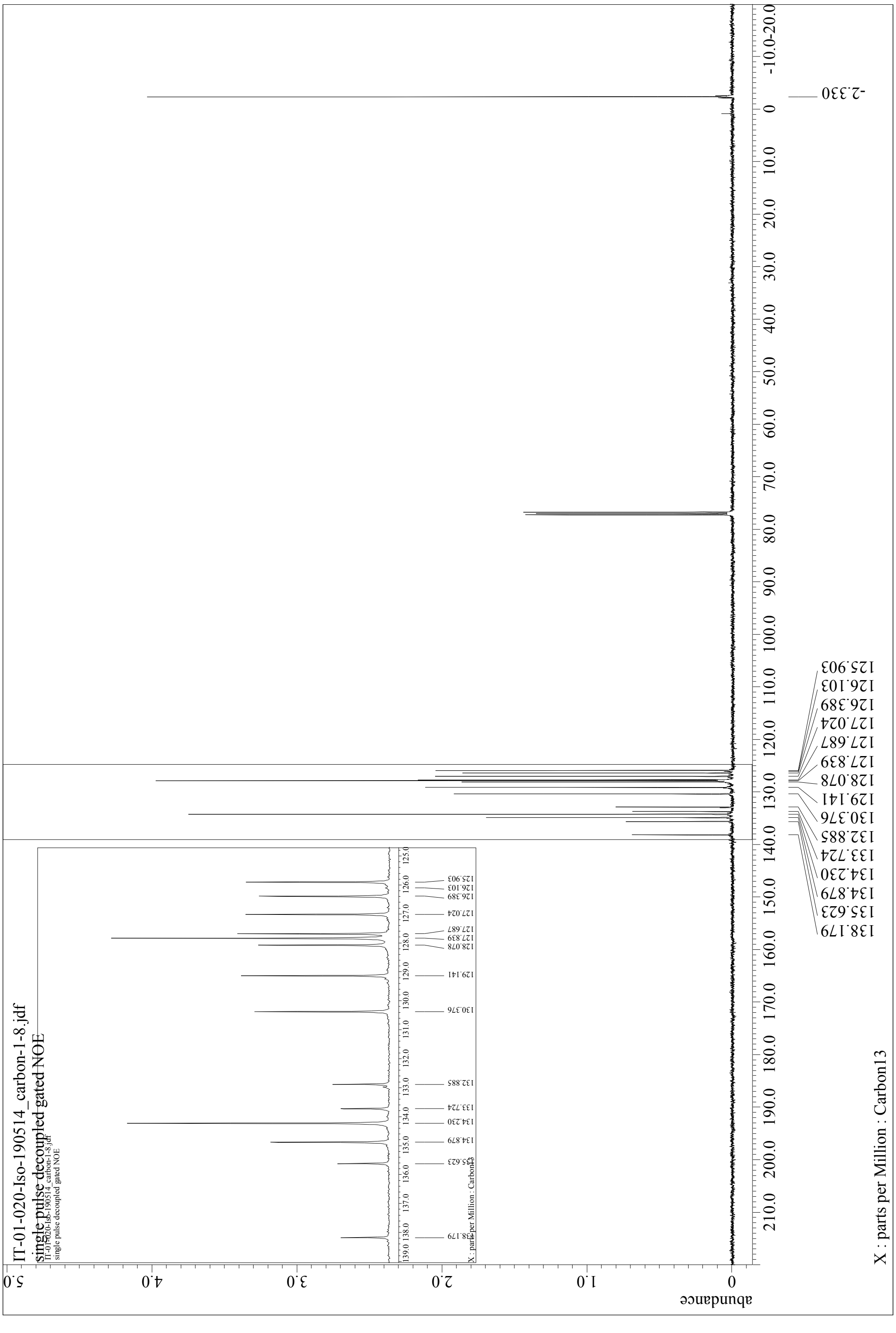

Supplementary Figure 9. ^{13}C NMR spectrum of **2b**

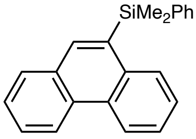

2c (^{1}H NMR, 500 MHz, $CDCl_3$)

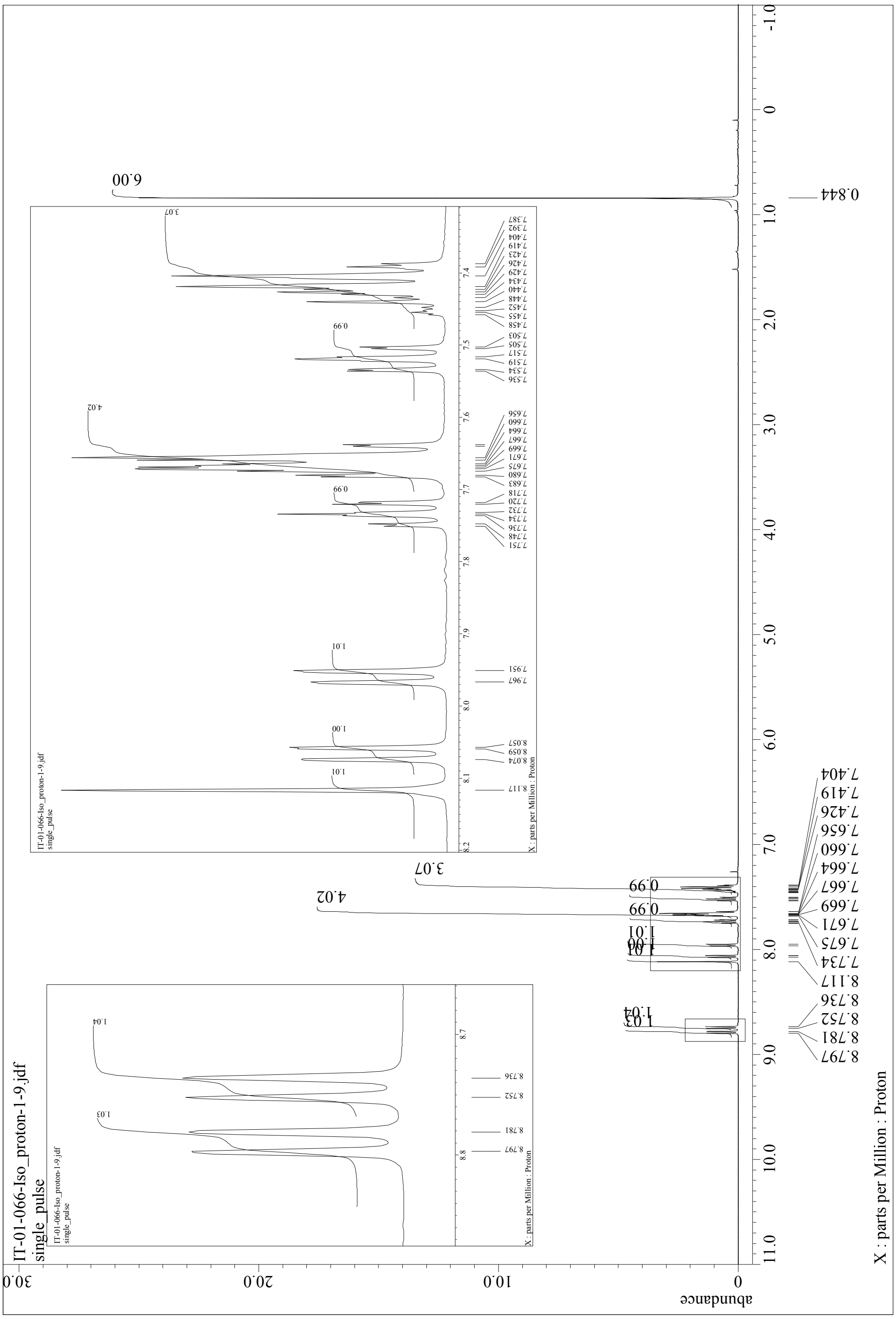

Supplementary Figure 10. ^{1}H NMR spectrum of **2c**

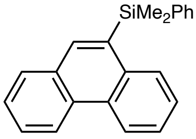

2c (^{13}C NMR, 126 MHz, $CDCl_3$)

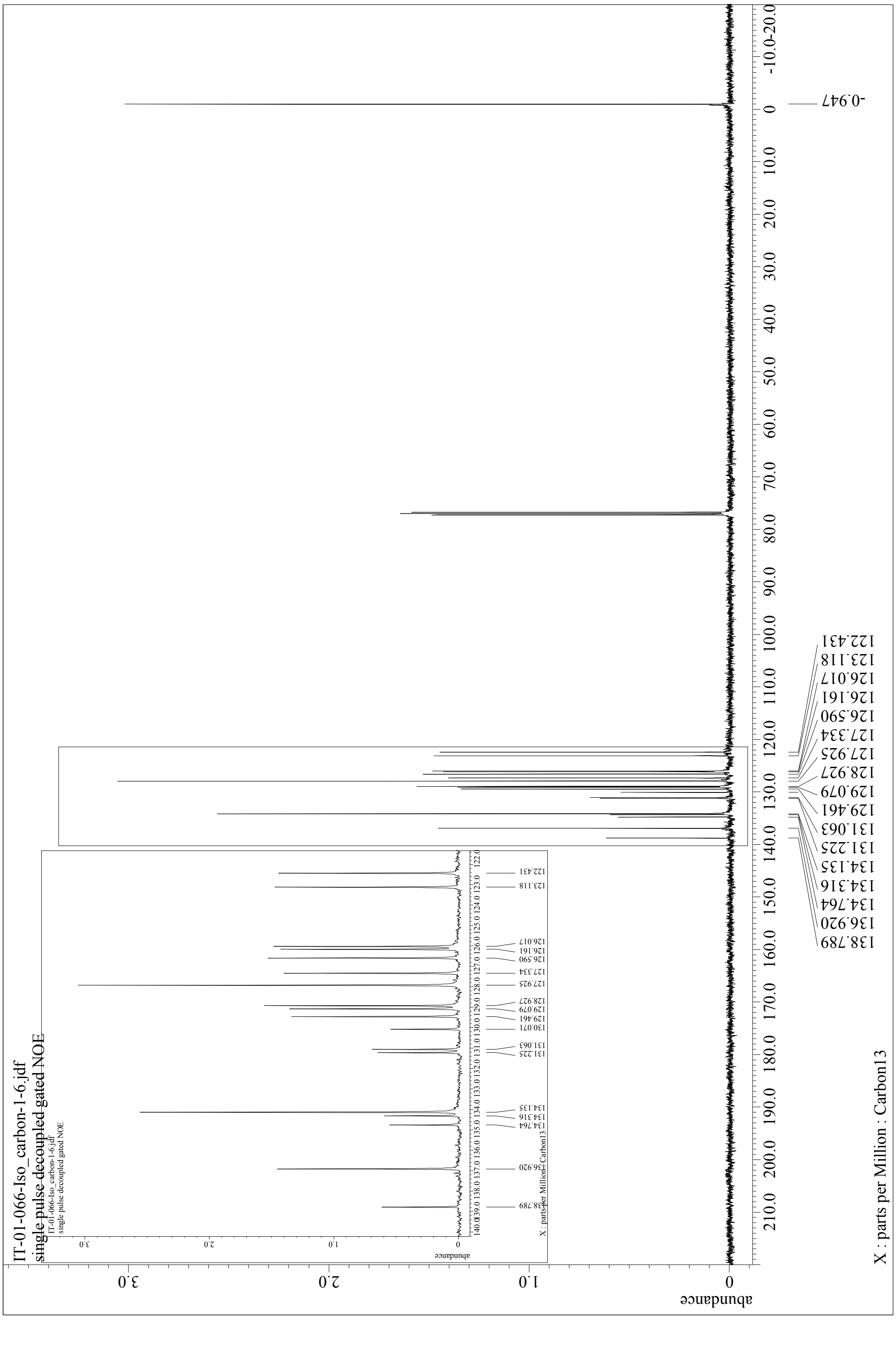

Supplementary Figure 11. ^{13}C NMR spectrum of **2c**

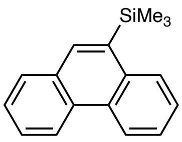

2c’ (^{1}H NMR, 500 MHz, $CDCl_3$)

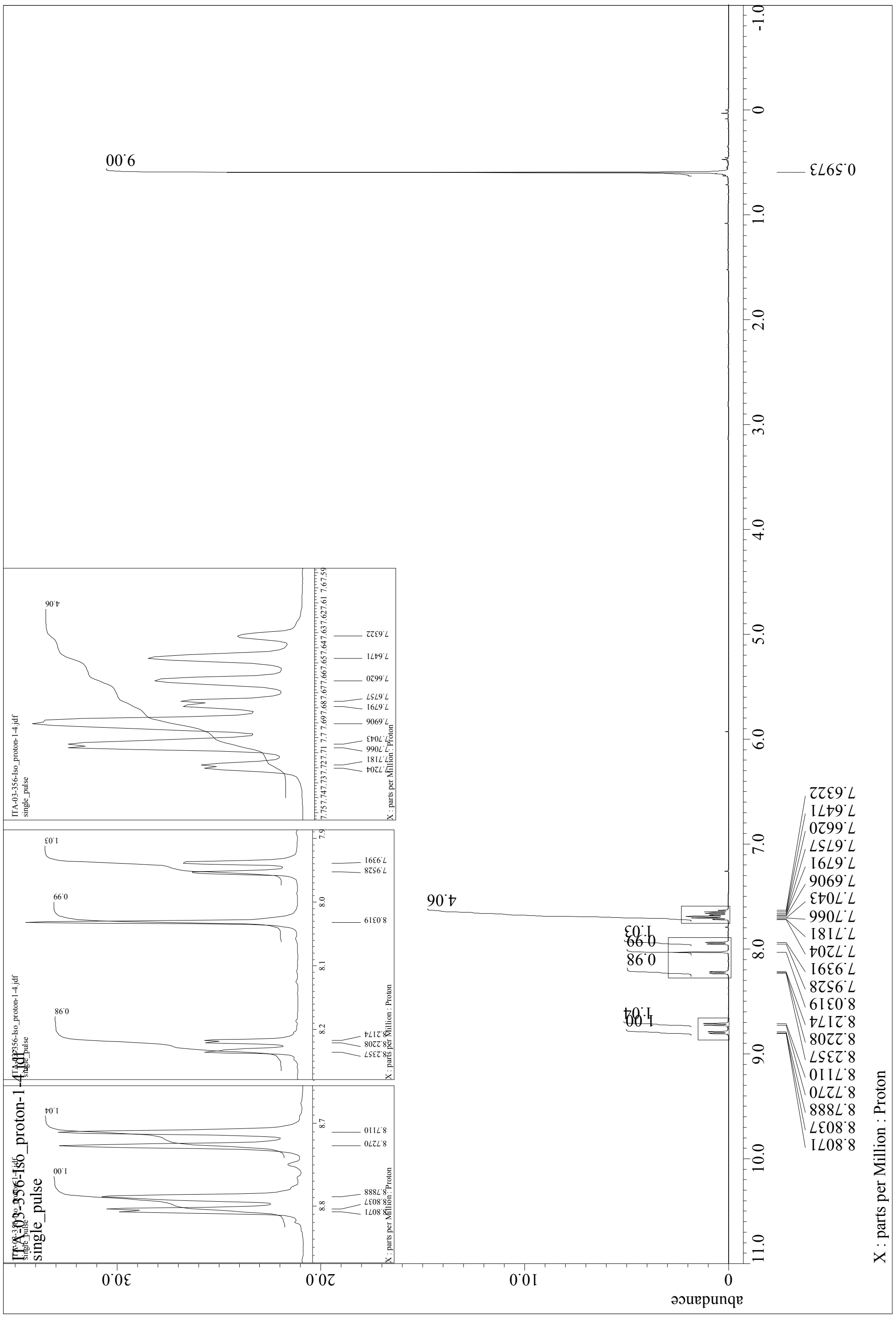

Supplementary Figure 12. ^{1}H NMR spectrum of **2c’**

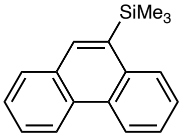

2c’ (^{13}C NMR, 126 MHz, $CDCl_3$)

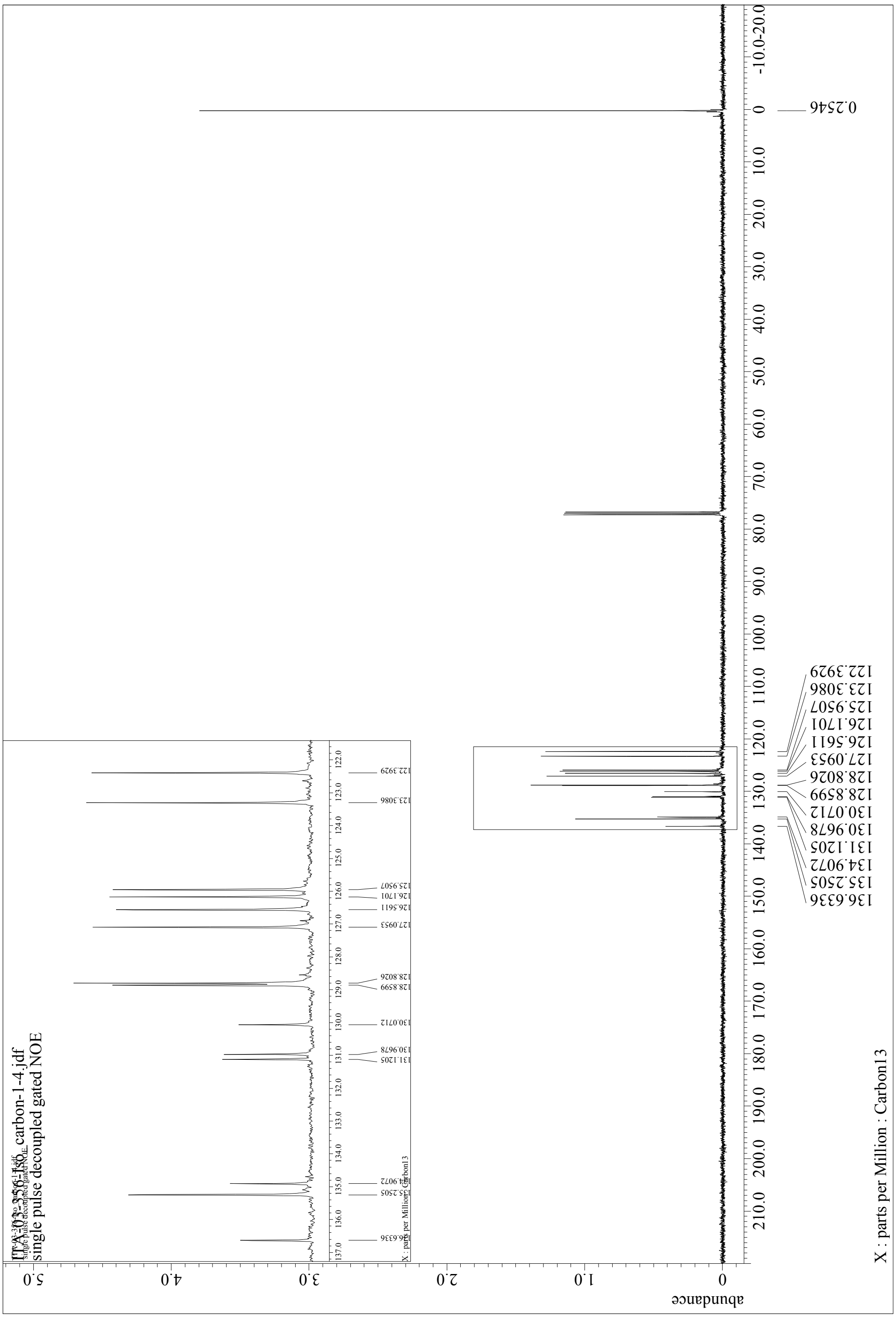

Supplementary Figure 13. ^{13}C NMR spectrum of **2c’**

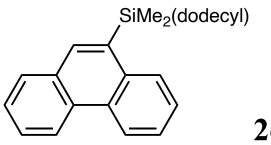

2c” (^{1}H NMR, 500 MHz, CDCl$_3$)

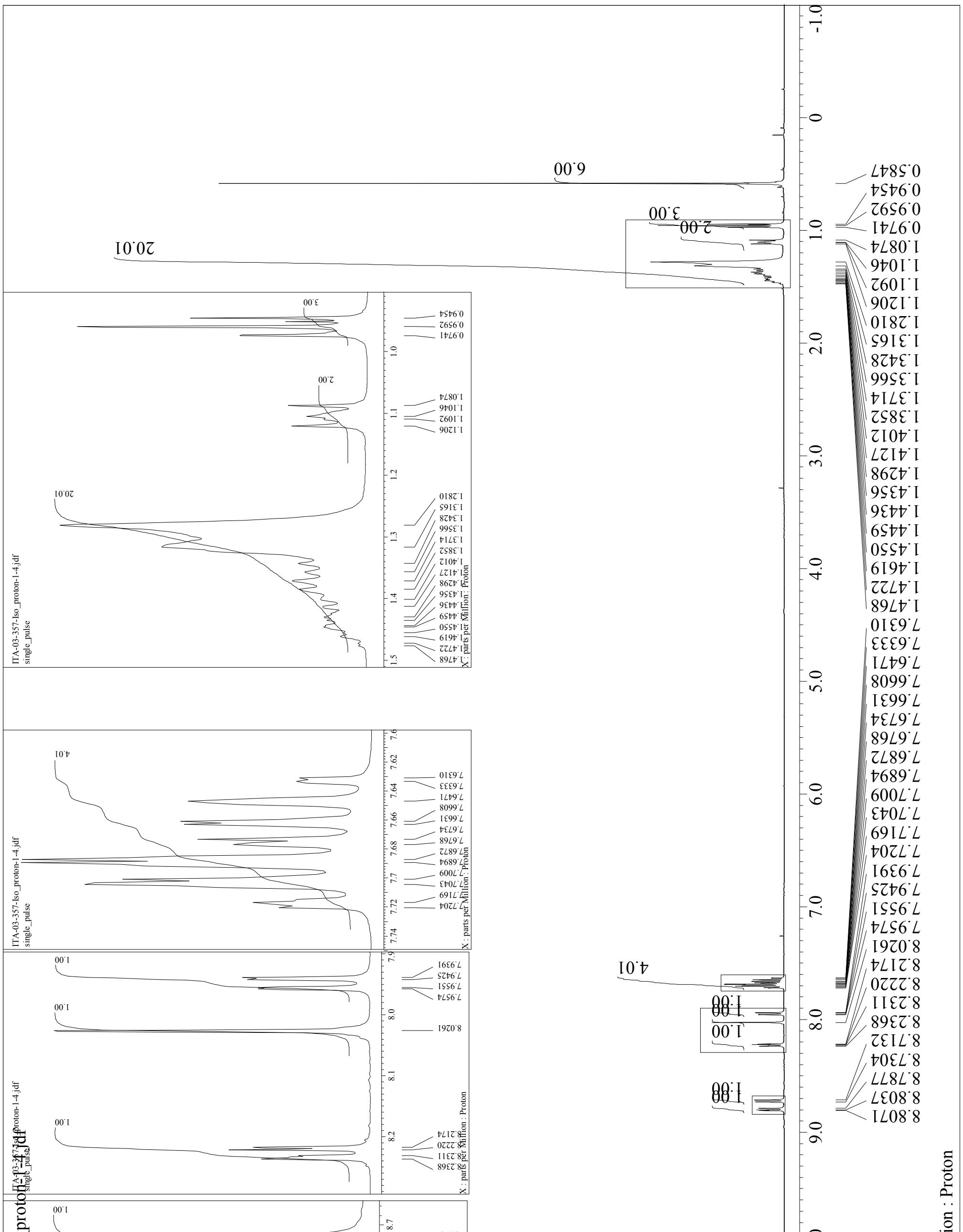

Supplementary Figure 14. ^{1}H NMR spectrum of **2c”**

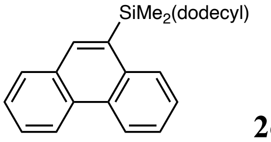

2c” (^{13}C NMR, 500 MHz, $CDCl_3$)

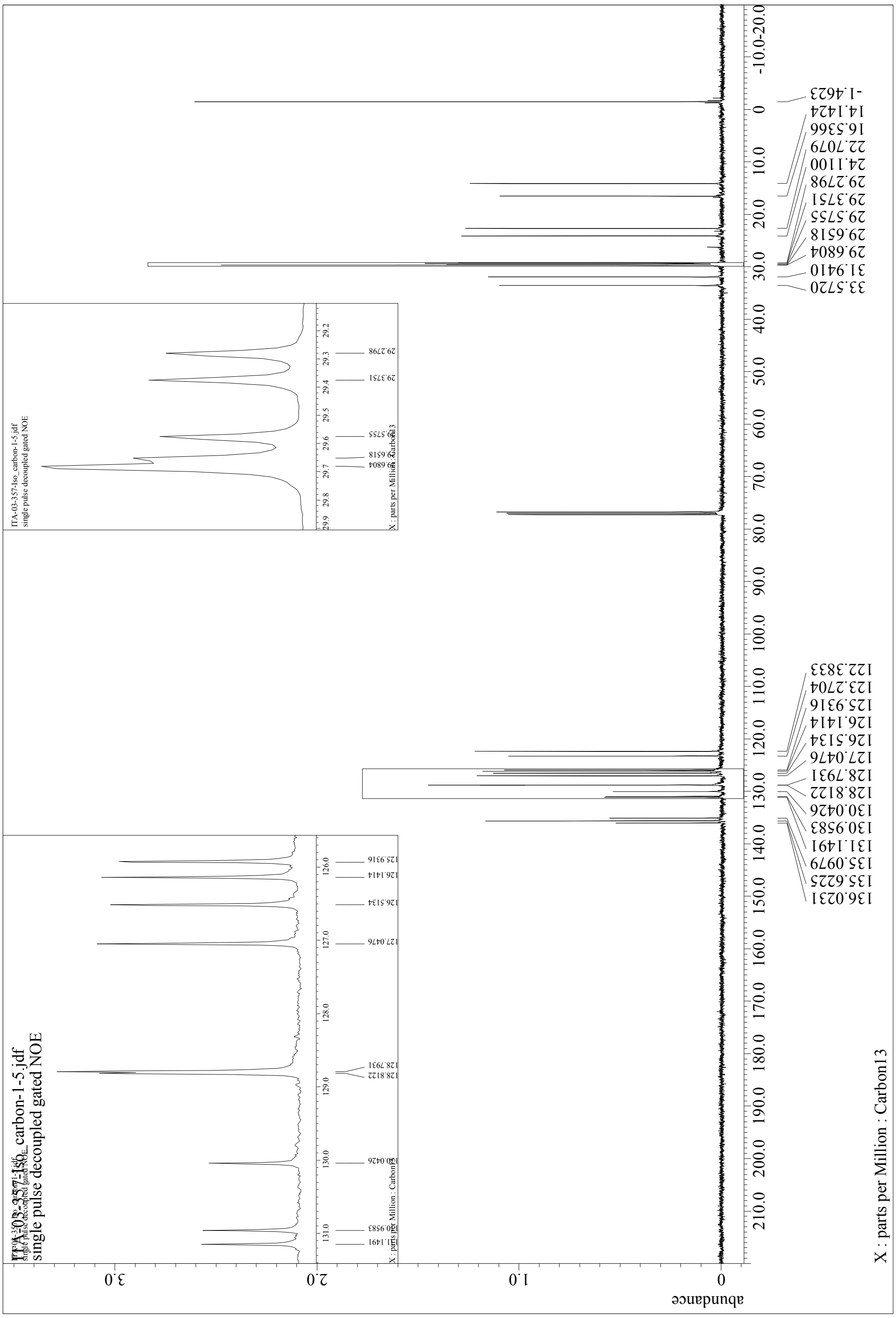

Supplementary Figure 15. ^{13}C NMR spectrum of **2c”**

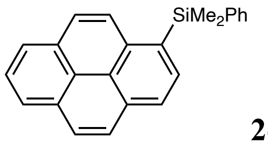

2d (^{1}H NMR, 500 MHz, $CDCl_3$)

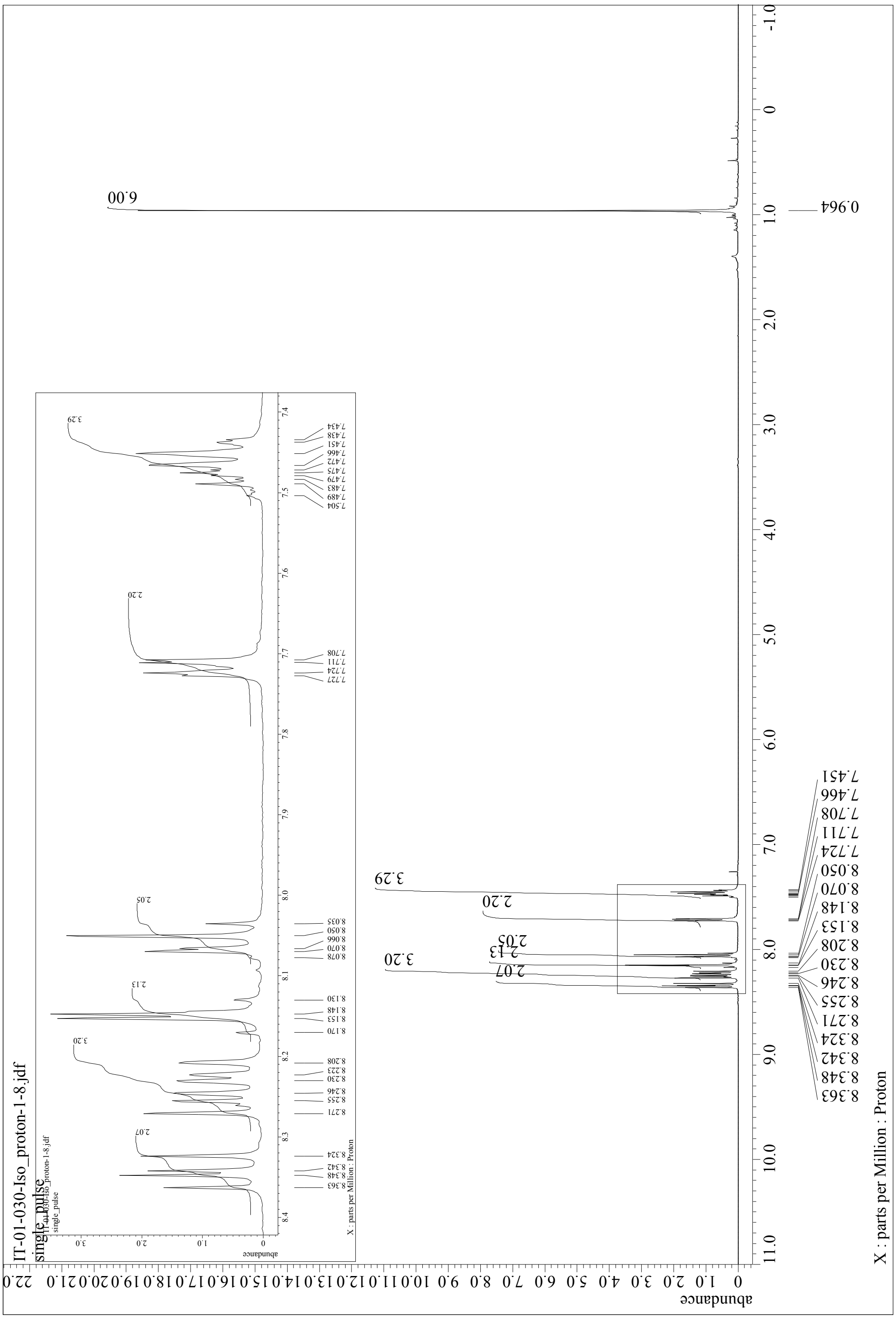

Supplementary Figure 16. ^{1}H NMR spectrum of **2d**

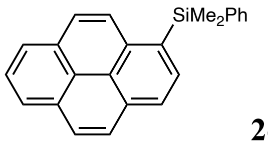

2d ([13] NMR, 126 MHz, $CDCl_3$)

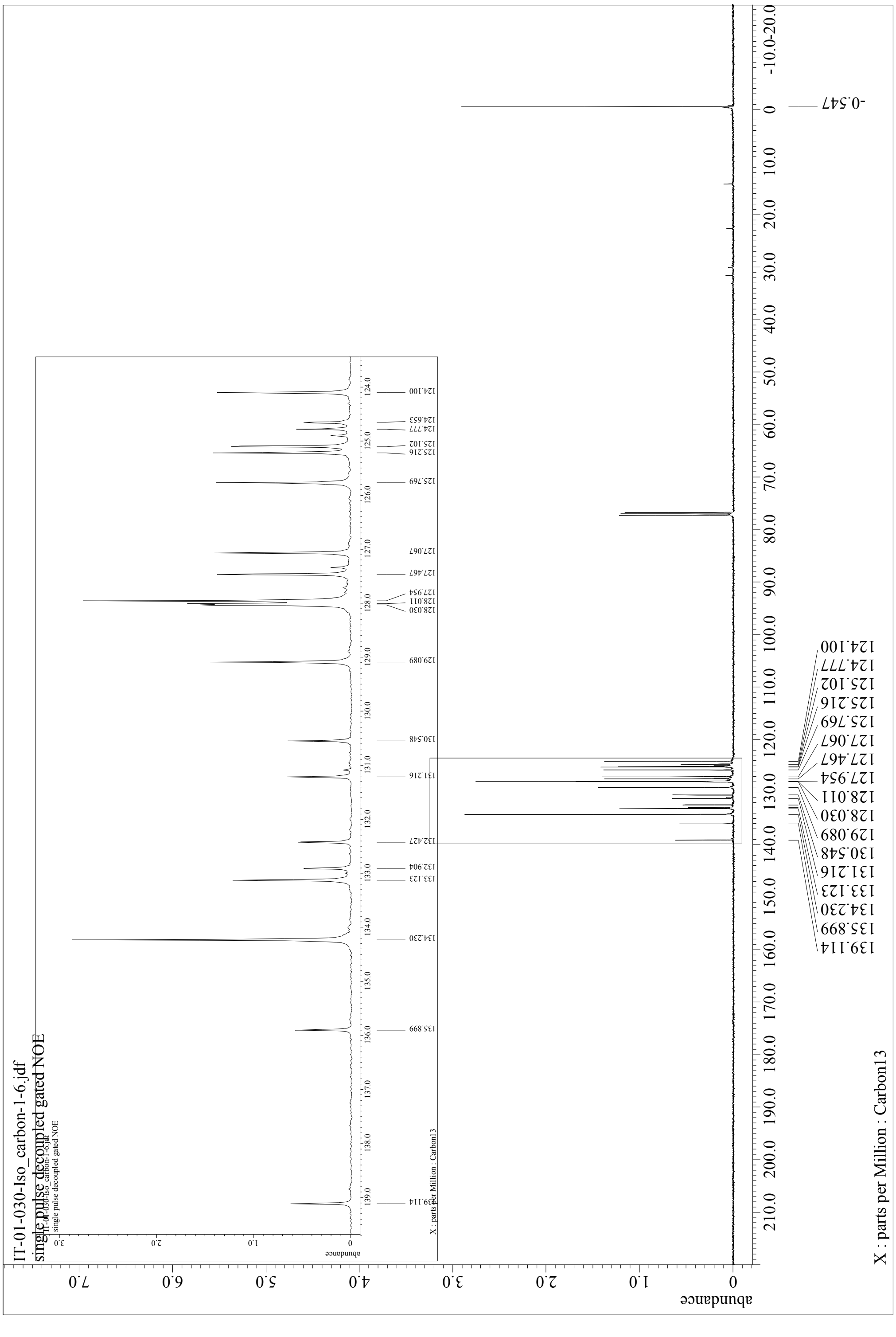

Supplementary Figure 17. ^{13}C NMR spectrum of **2d**

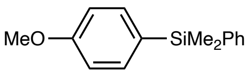

2e (^{1}H NMR, 500 MHz, $CDCl_3$)

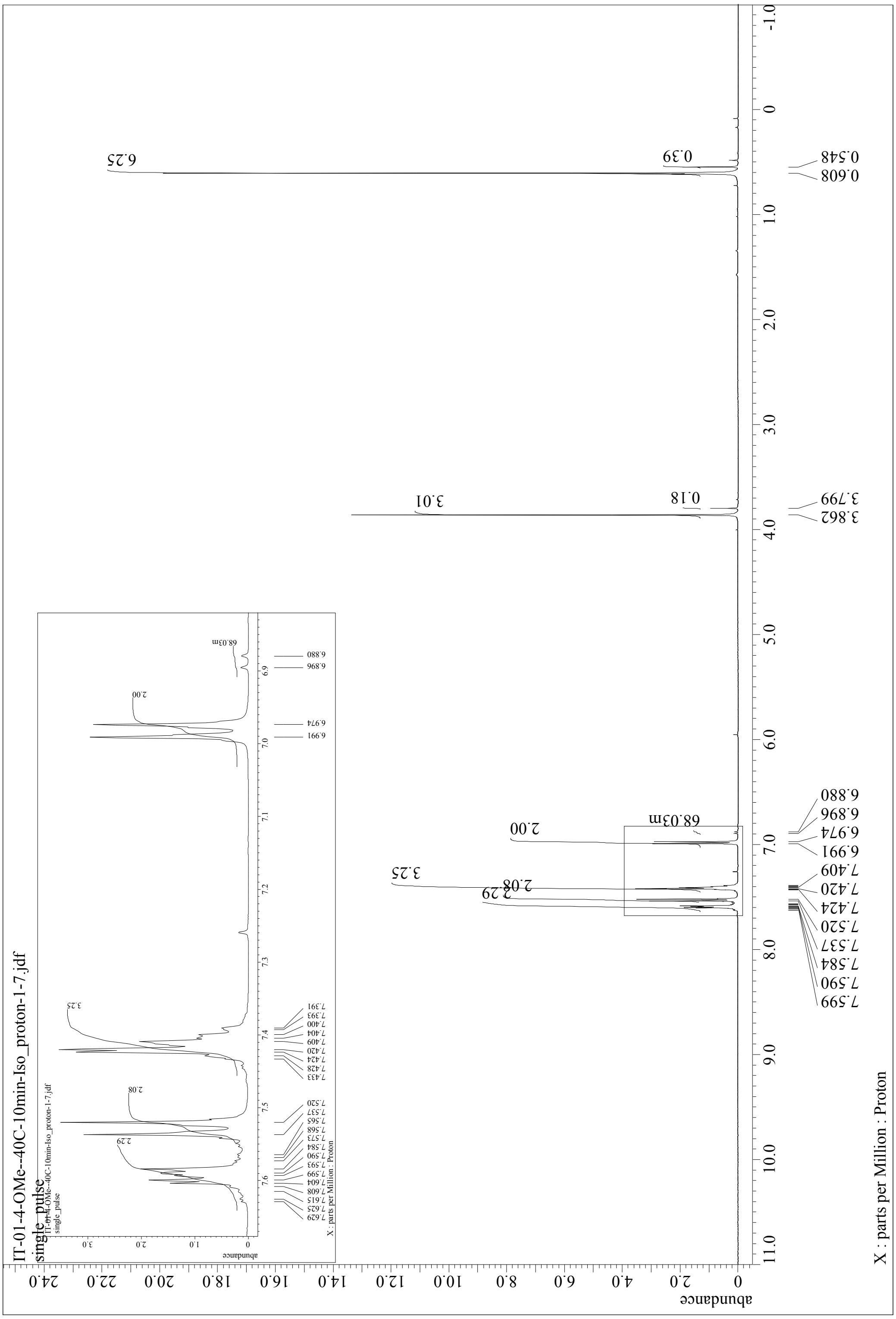

Supplementary Figure 18. ^{1}H NMR spectrum of **2e**

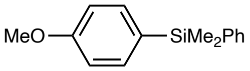

2e (^{13}C NMR, 126 MHz, $CDCl_3$)

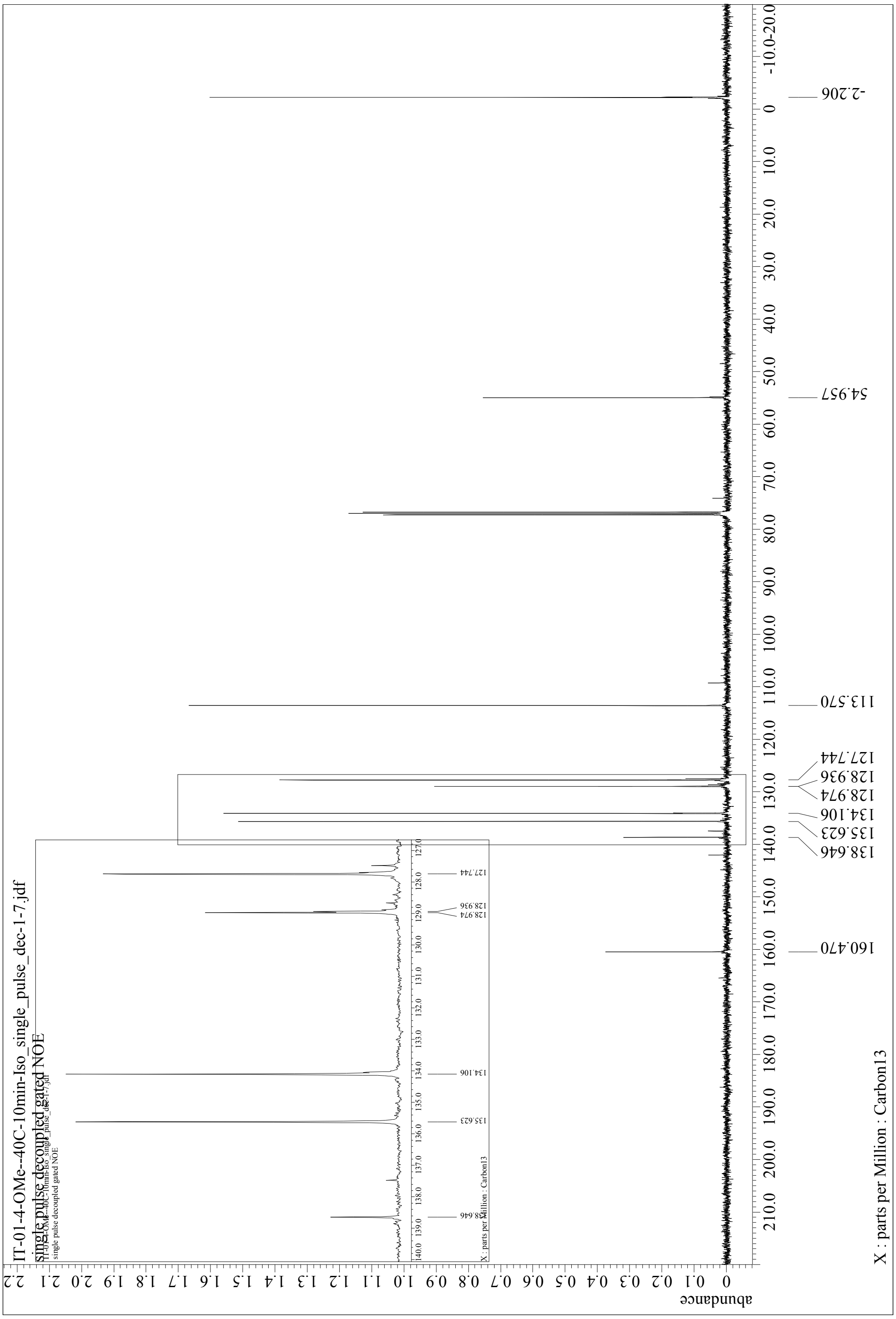

Supplementary Figure 19. ^{13}C NMR spectrum of **2e**

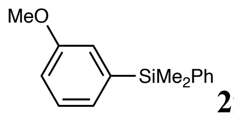

2f (^{1}H NMR, 500 MHz, $CDCl_3$)

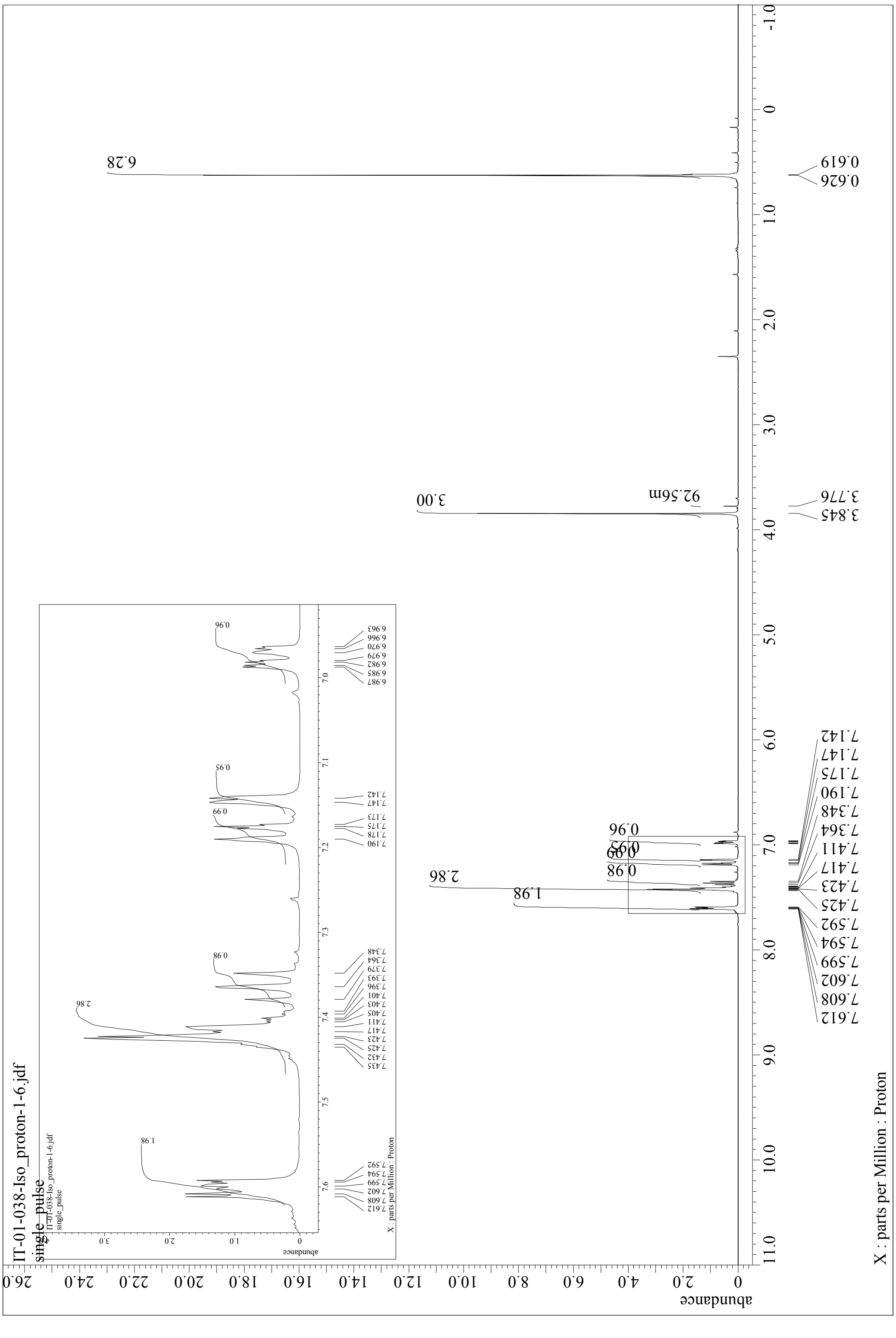

Supplementary Figure 20. ^{1}H NMR spectrum of **2f**

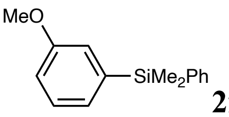

2f (^{13}C NMR, 126 MHz, $CDCl_3$)

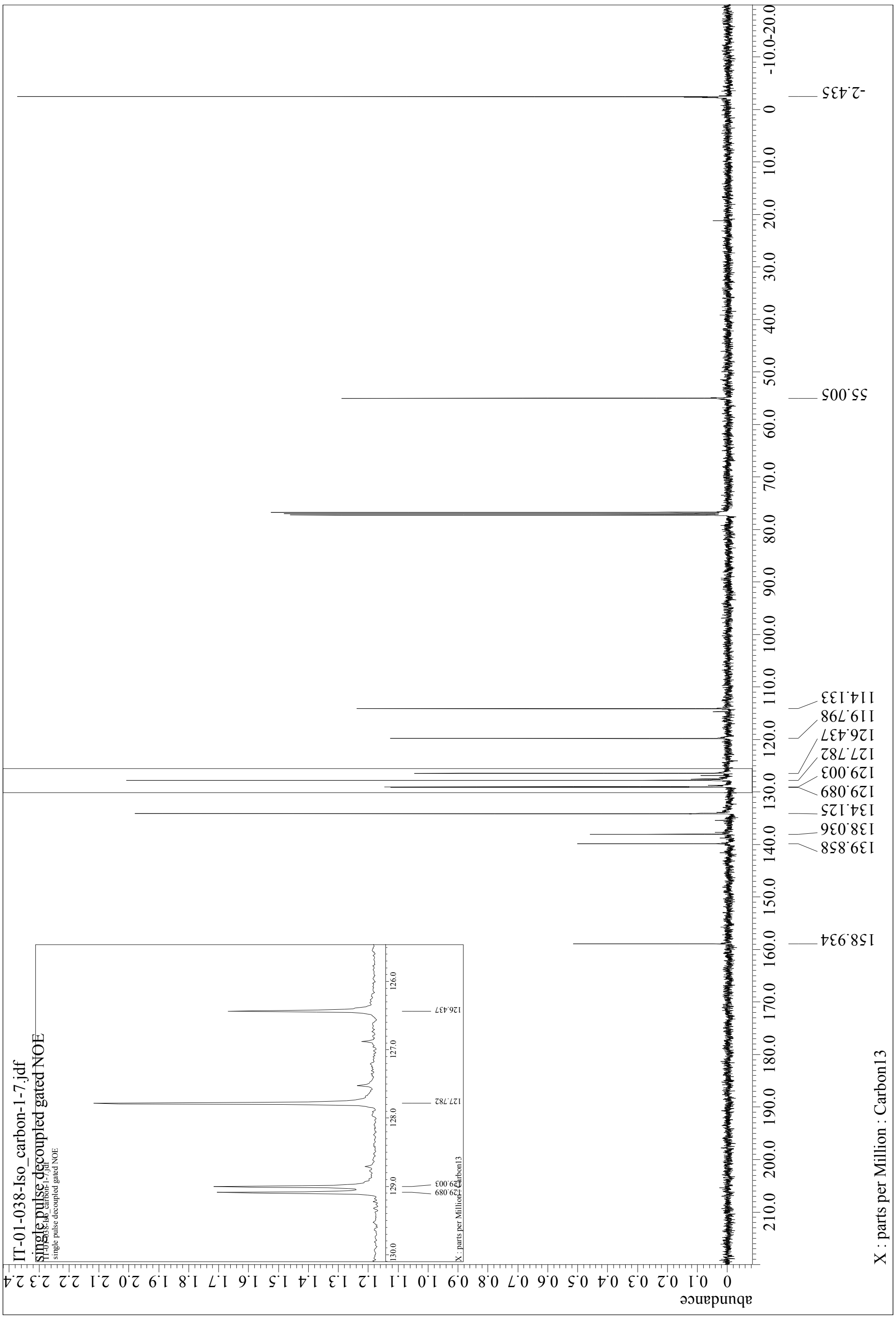

Supplementary Figure 21. ^{13}C NMR spectrum of **2f**

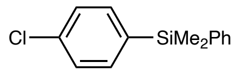

2g (^{1}H NMR, 500 MHz, $CDCl_3$)

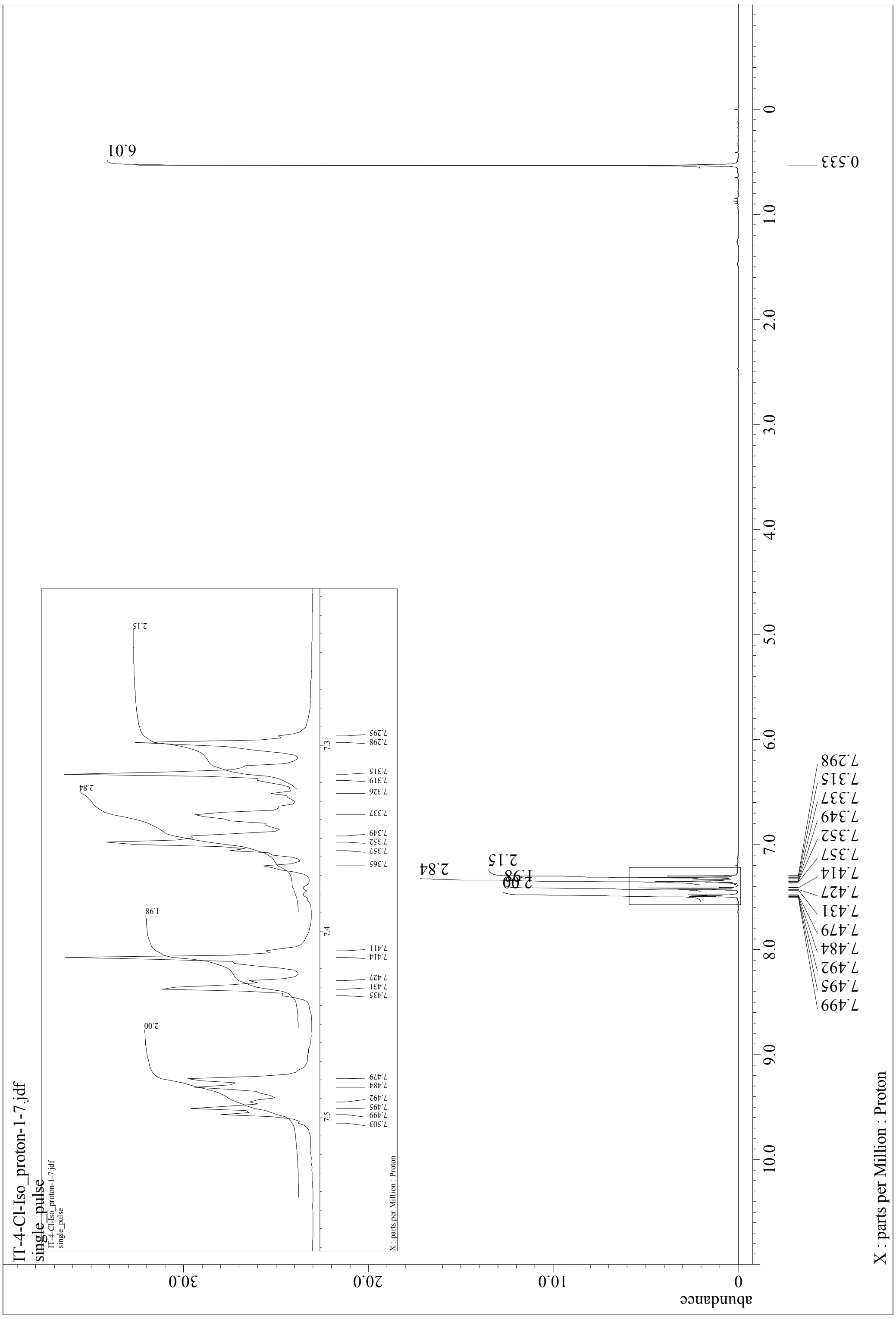

Supplementary Figure 22. ^{1}H NMR spectrum of **2g**

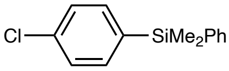

2g (^{13}C NMR, 126 MHz, $CDCl_3$)

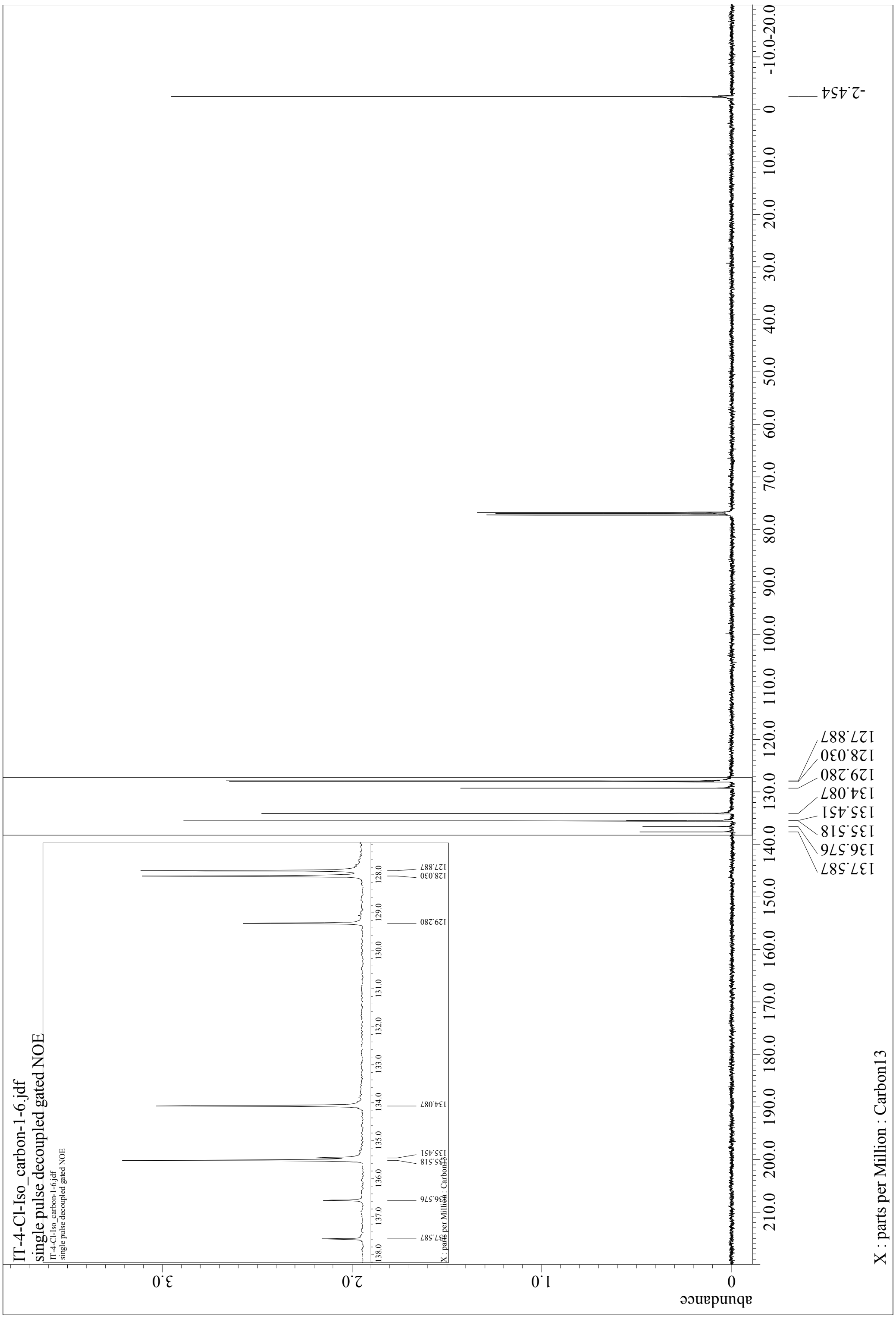

Supplementary Figure 23. ^{13}C NMR spectrum of **2g**

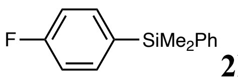

2h (^{1}H NMR, 500 MHz, $CDCl_3$)

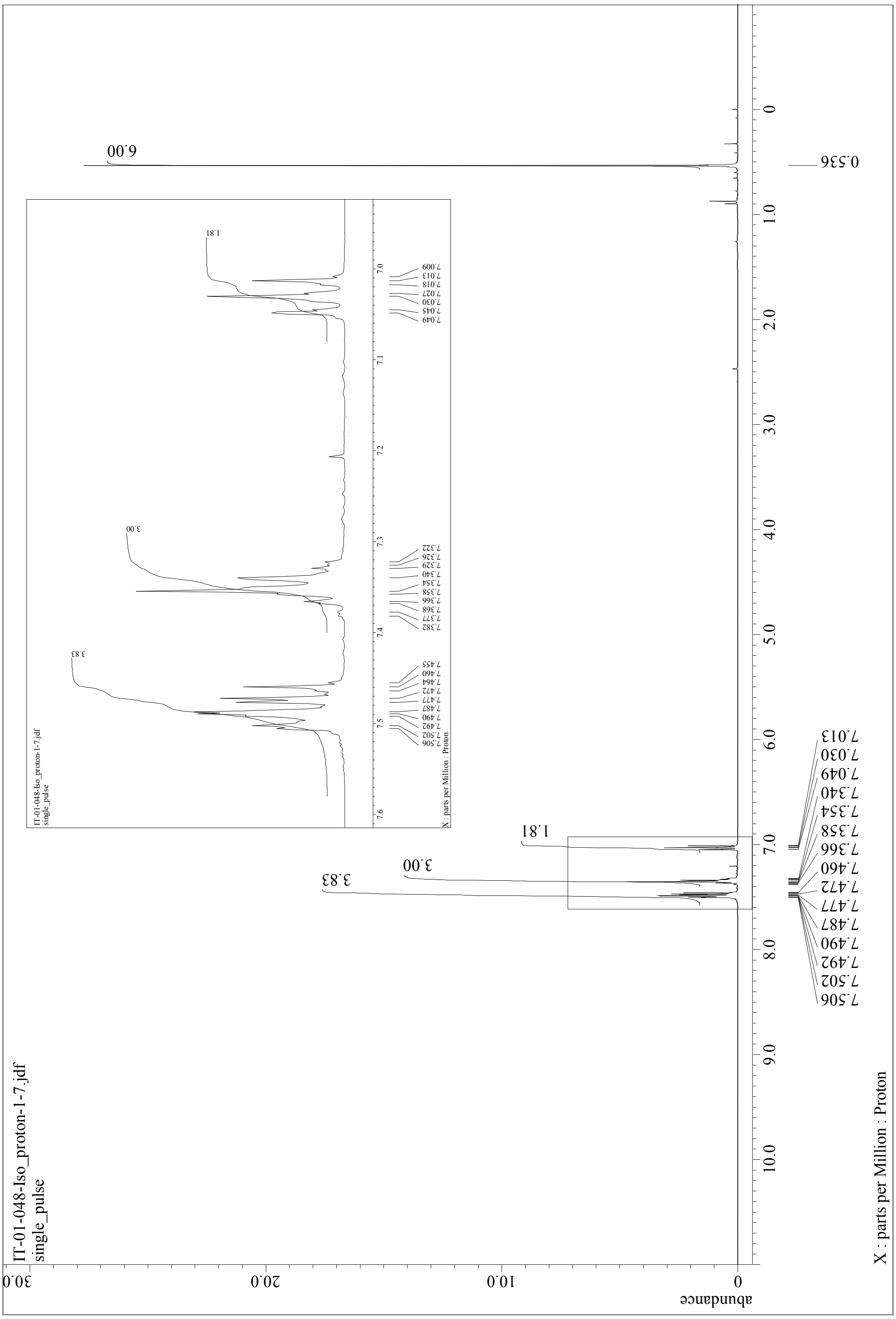

Supplementary Figure 24. ^{1}H NMR spectrum of **2h**

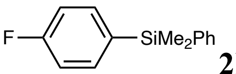

2h (^{13}C NMR, 126 MHz, $CDCl_3$)

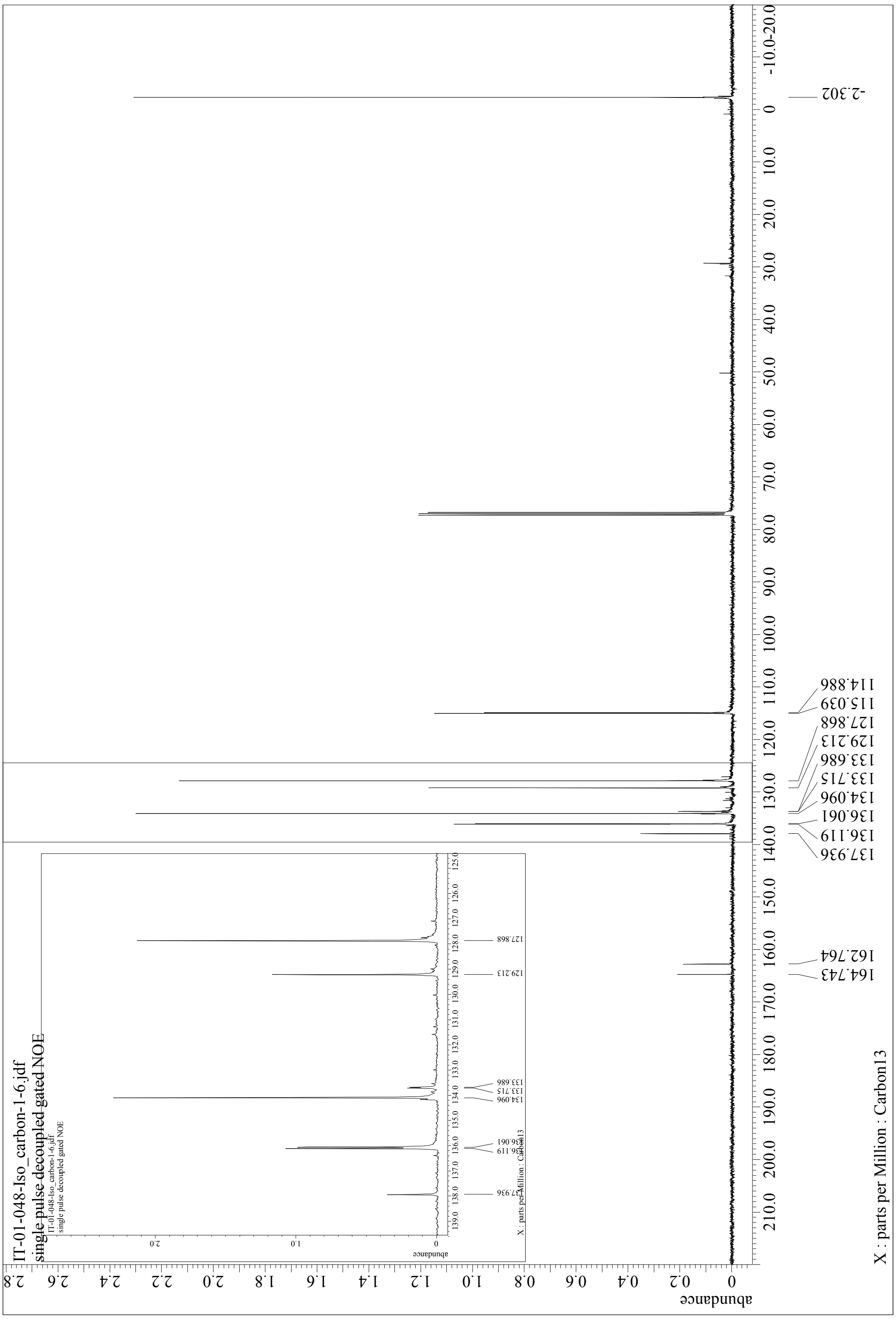

Supplementary Figure 25. ^{13}C NMR spectrum of **2h**

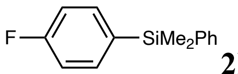

2h (^{19}F NMR, 470 MHz, $CDCl_3$)

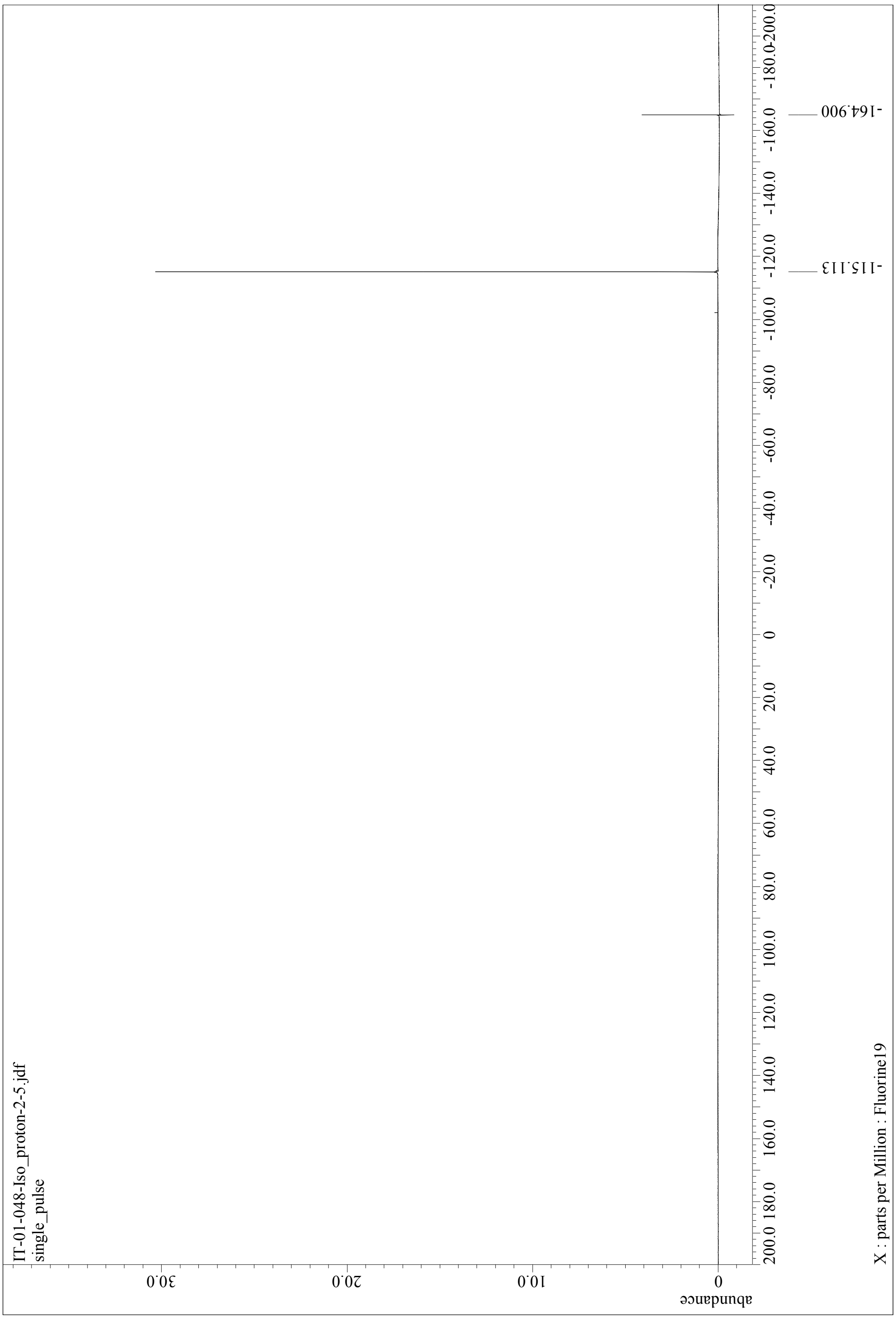

Supplementary Figure 26. ^{19}F NMR spectrum of **2h**

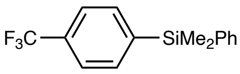

2i (^{1}H NMR, 500 MHz, $CDCl_3$)

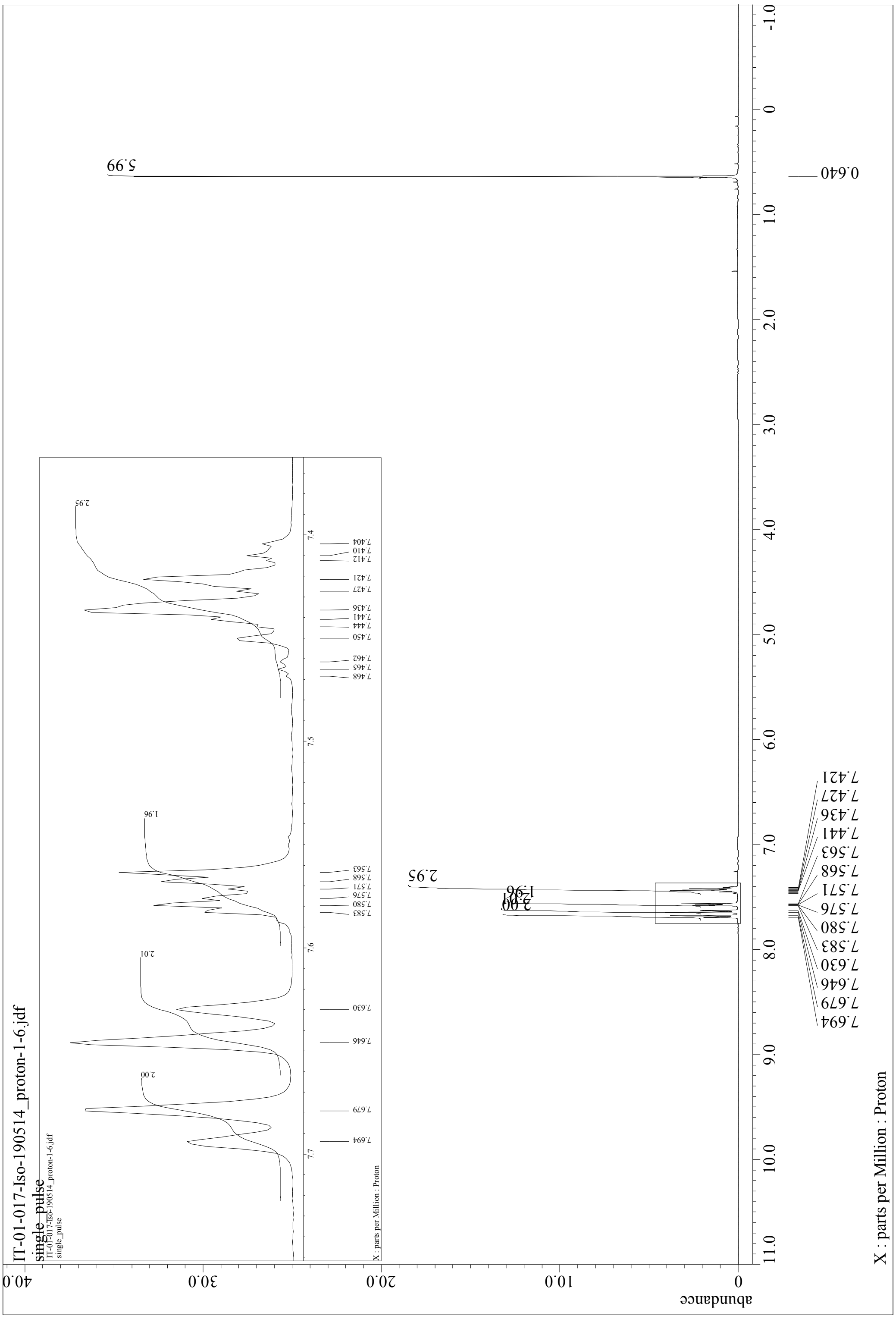

Supplementary Figure 27. ^{1}H NMR spectrum of **2i**

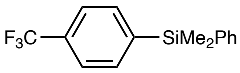

2i (^{13}C NMR, 126 MHz, $CDCl_3$)

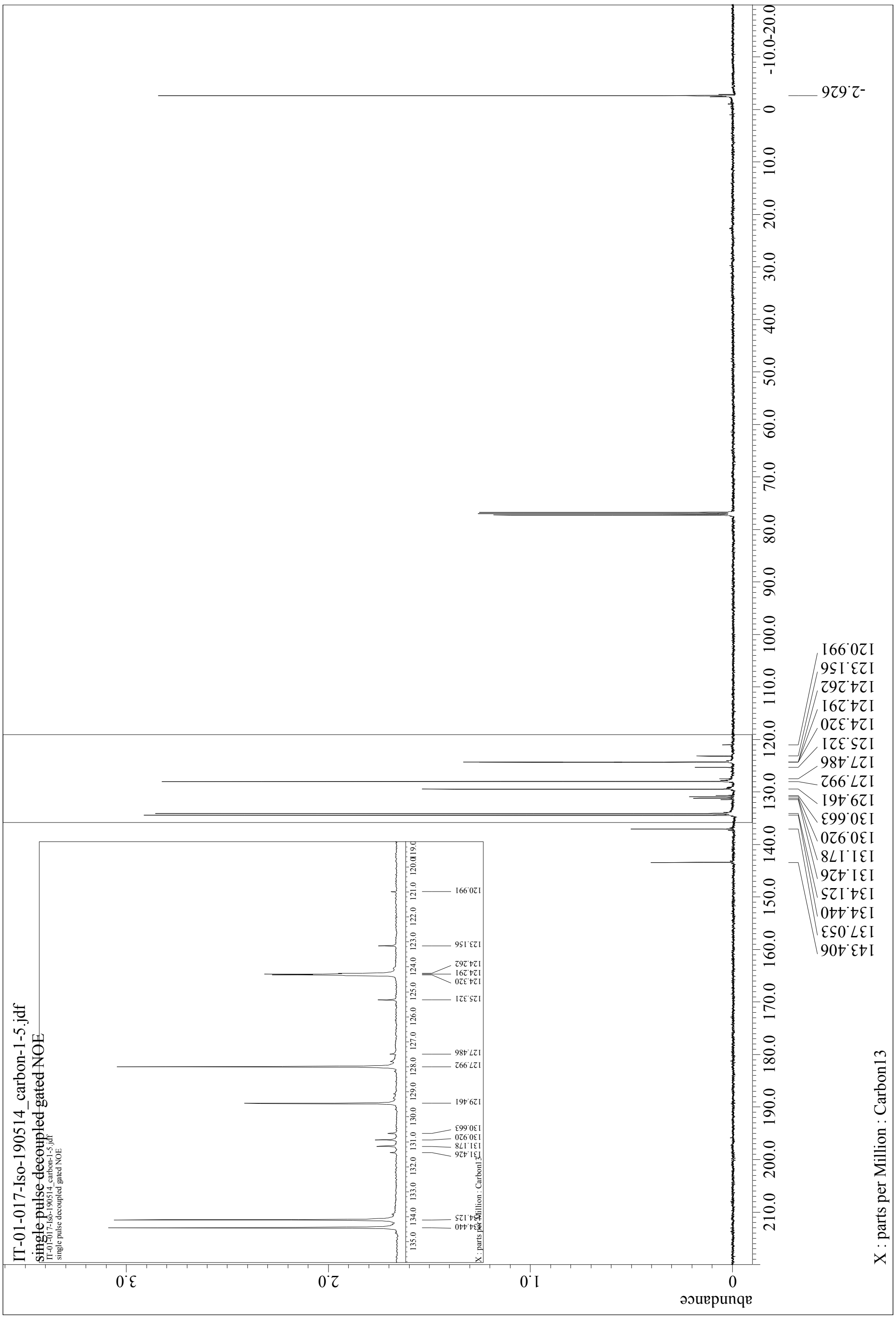

Supplementary Figure 28. ^{13}C NMR spectrum of **2i**

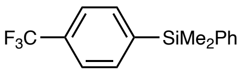

2i (^{19}F NMR, 470 MHz, $CDCl_3$)

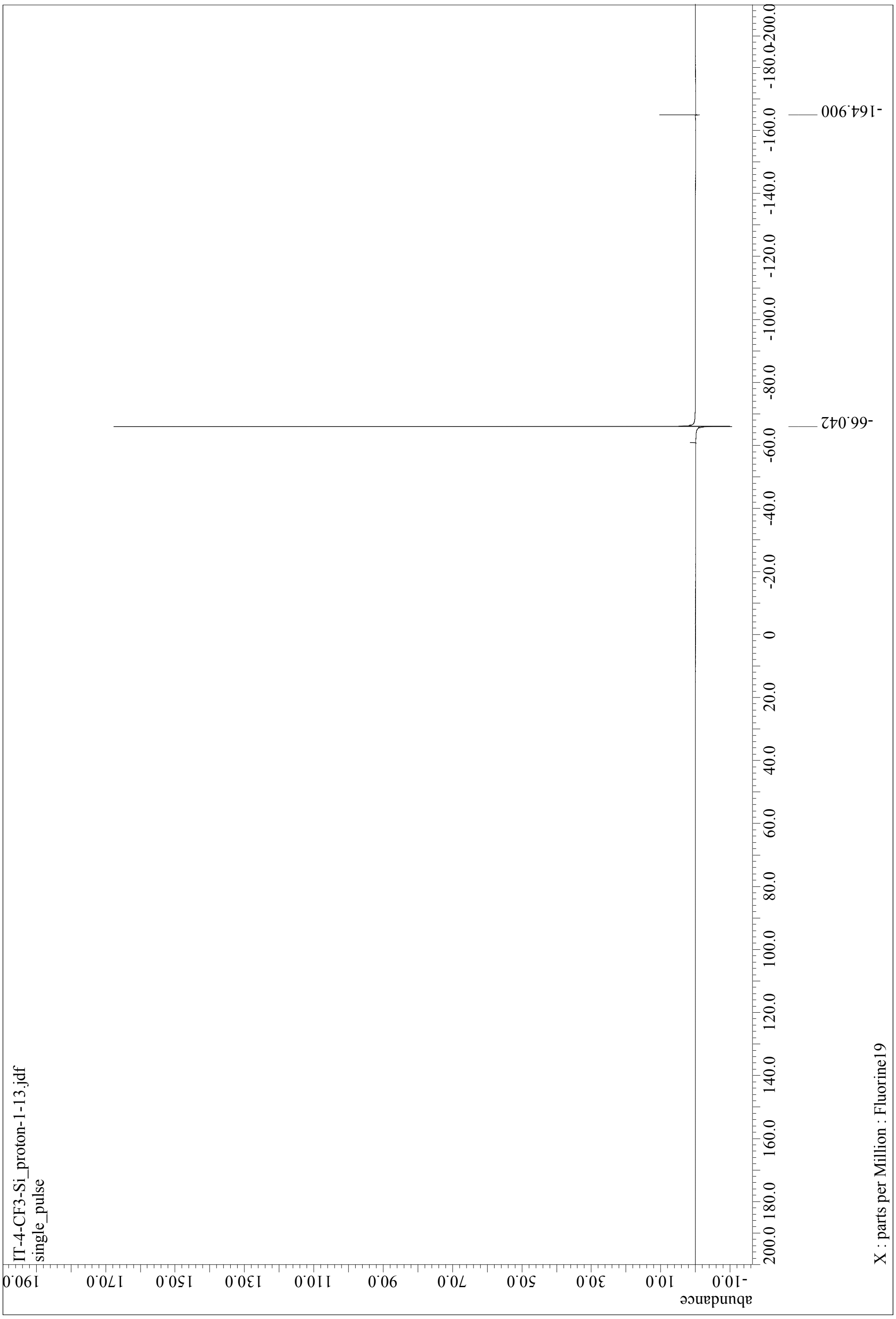

Supplementary Figure 29. ^{19}F NMR spectrum of **2i**

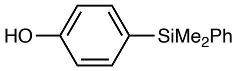

2j (^{1}H NMR, 500 MHz, $CDCl_3$)

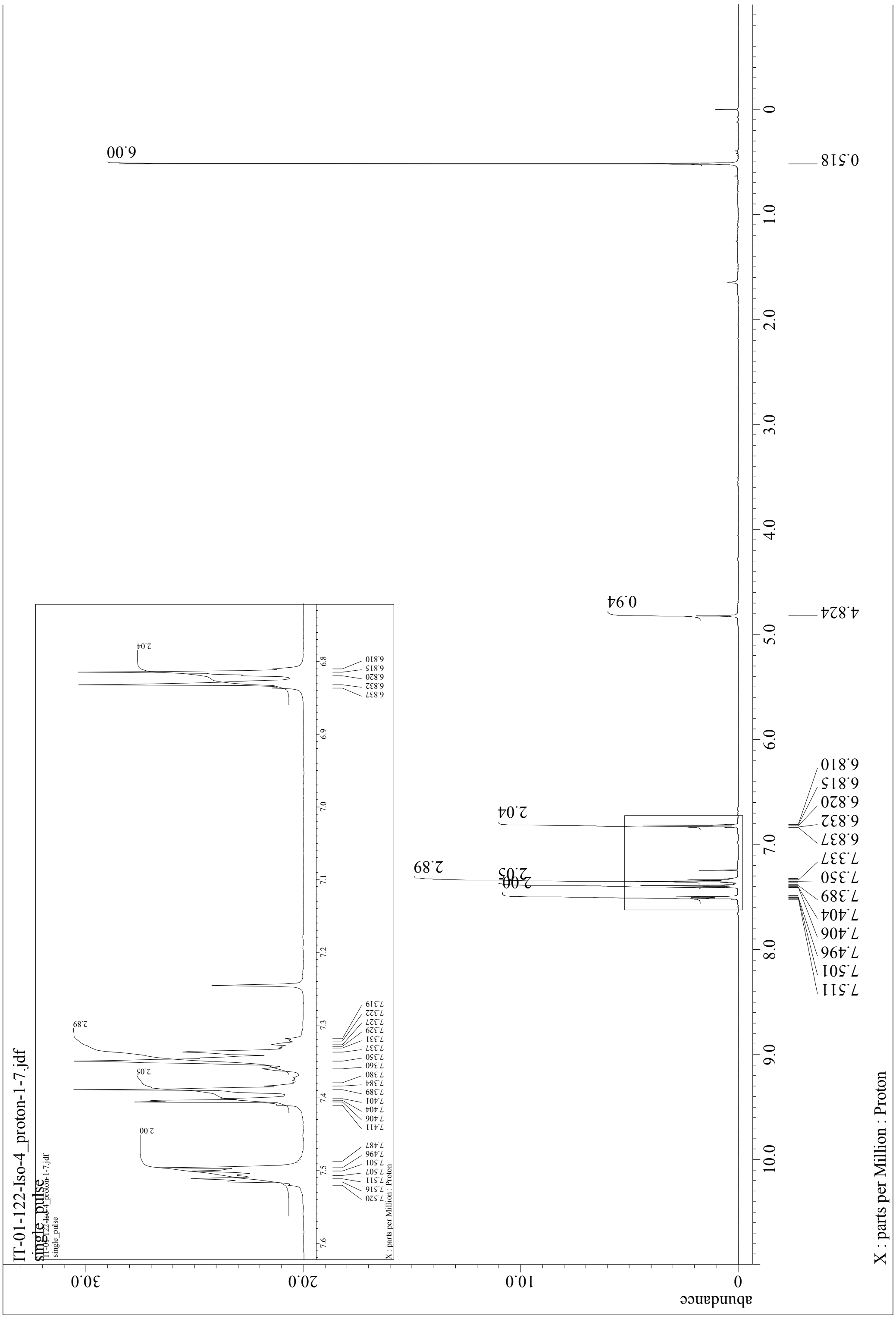

Supplementary Figure 30. ^{1}H NMR spectrum of **2j**

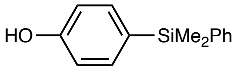

2j (^{13}C NMR, 126 MHz, $CDCl_3$)

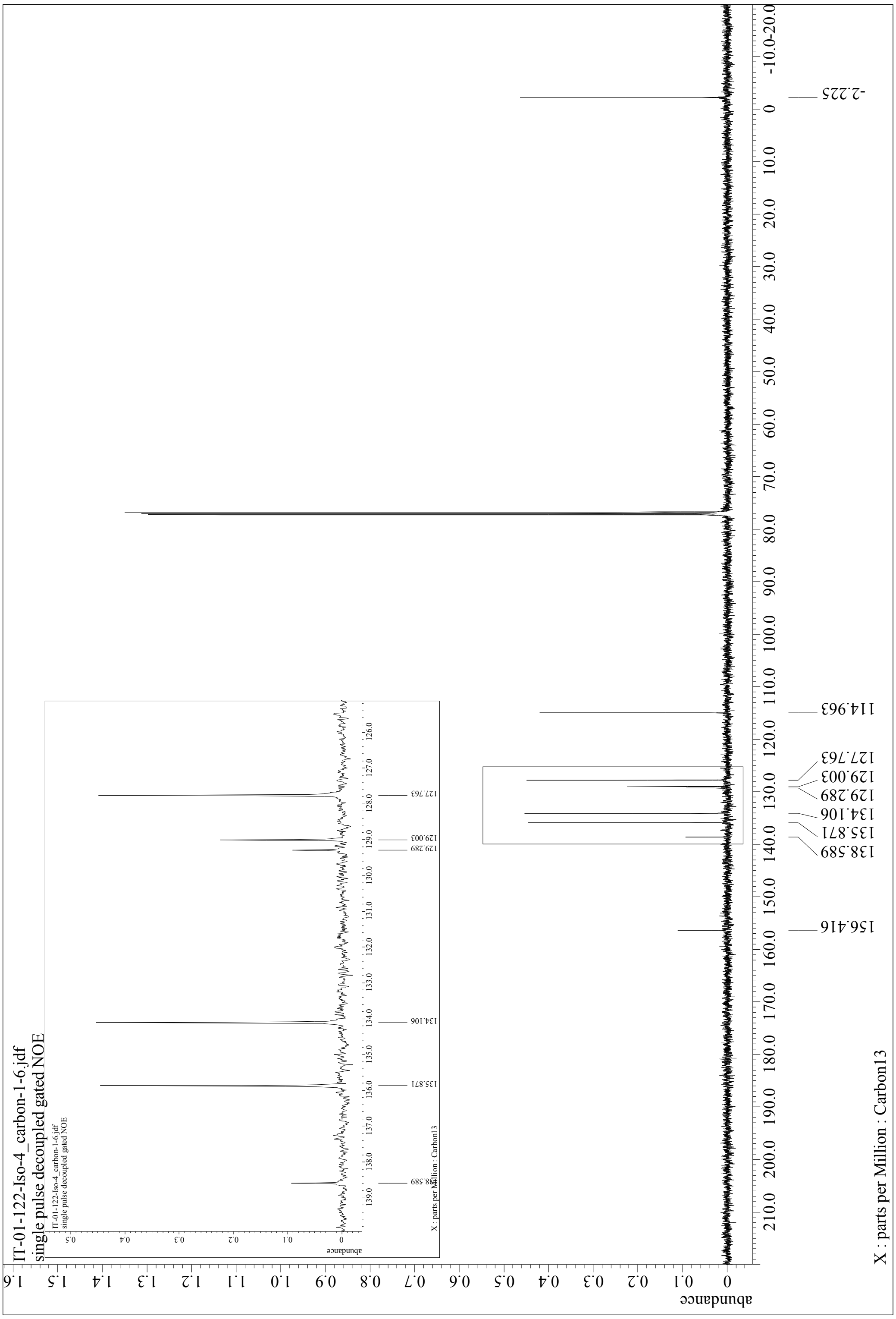

Supplementary Figure 31. ^{13}C NMR spectrum of **2j**

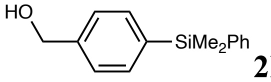

2k (^{1}H NMR, 500 MHz, $CDCl_3$)

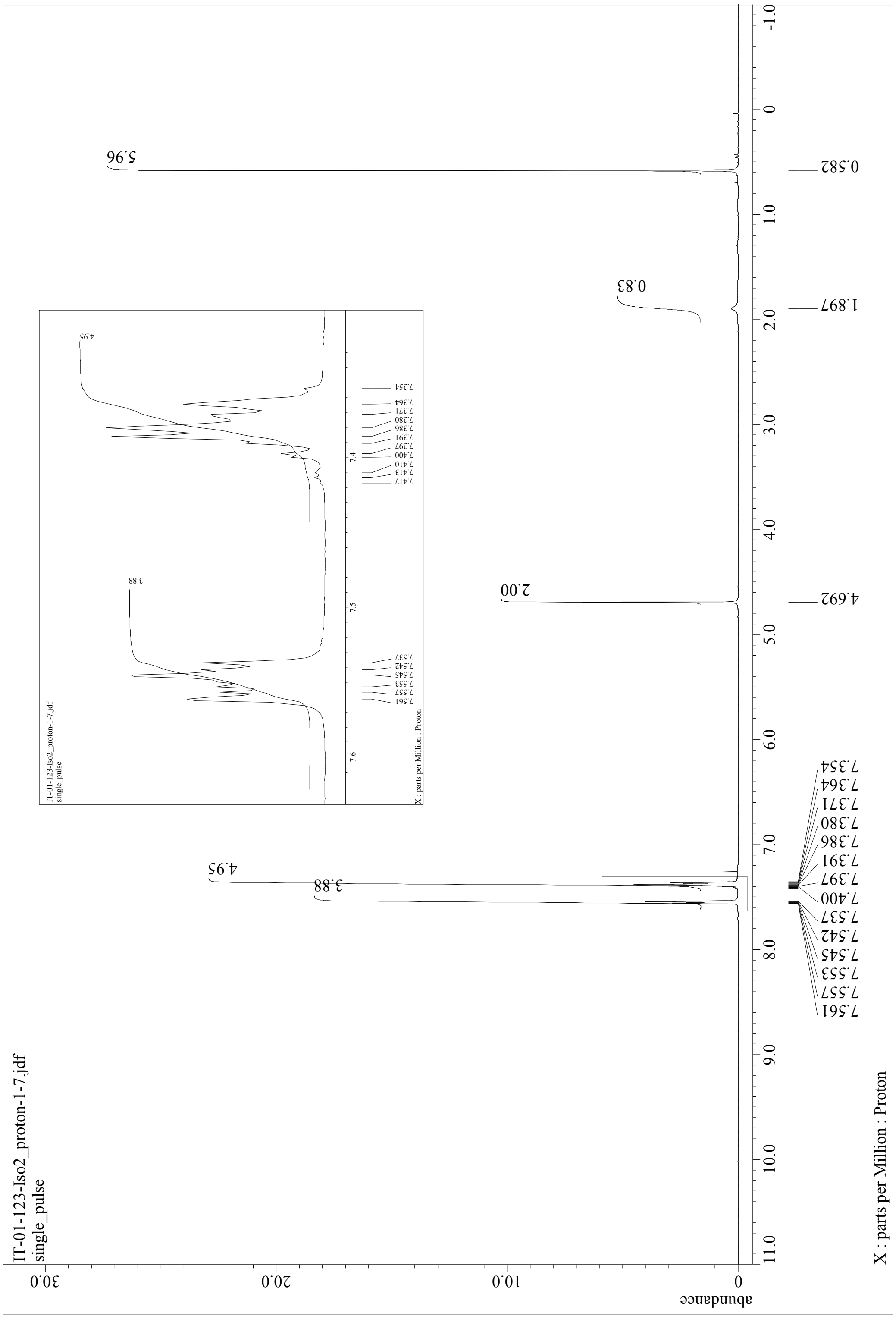

Supplementary Figure 32. ^{1}H NMR spectrum of **2k**

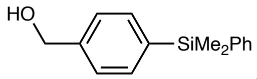

2k (^{13}C NMR, 126 MHz, $CDCl_3$)

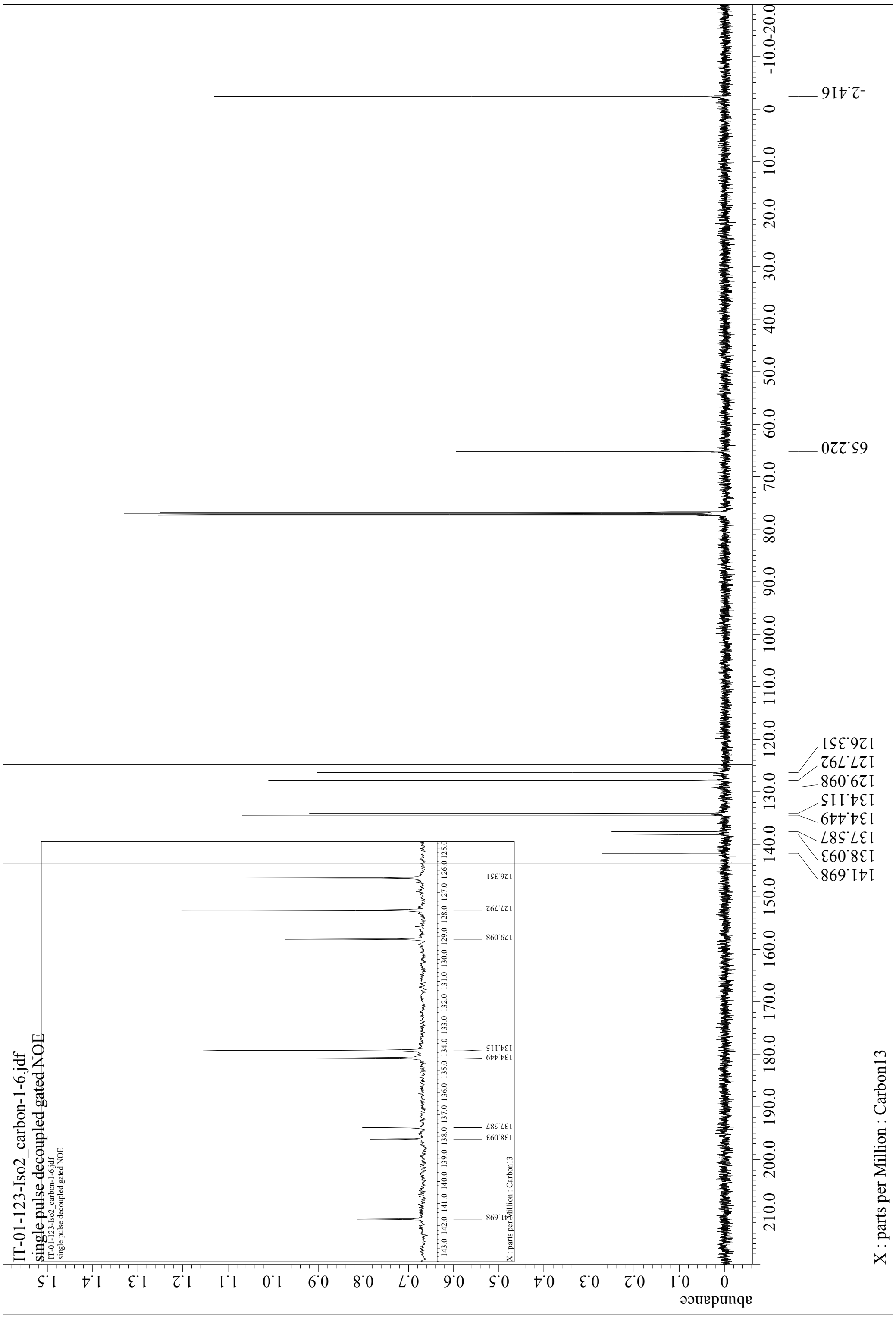

Supplementary Figure 33. ^{13}C NMR spectrum of **2k**

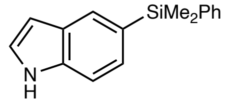

2l (^{1}H NMR, 500 MHz, $CDCl_3$)

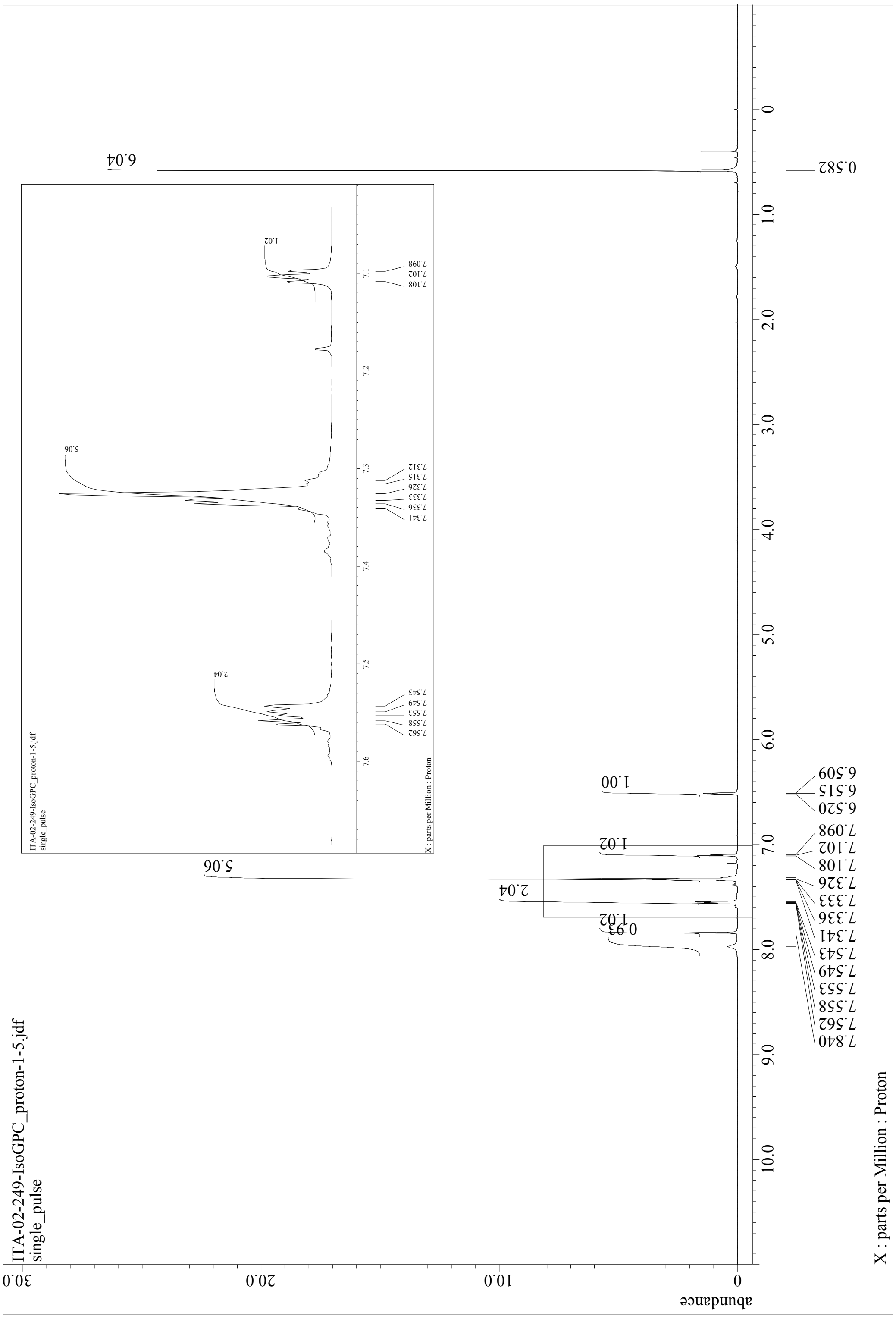

Supplementary Figure 34. ^{1}H NMR spectrum of **2l**

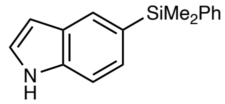

2l (^{13}C NMR, 126 MHz, $CDCl_3$)

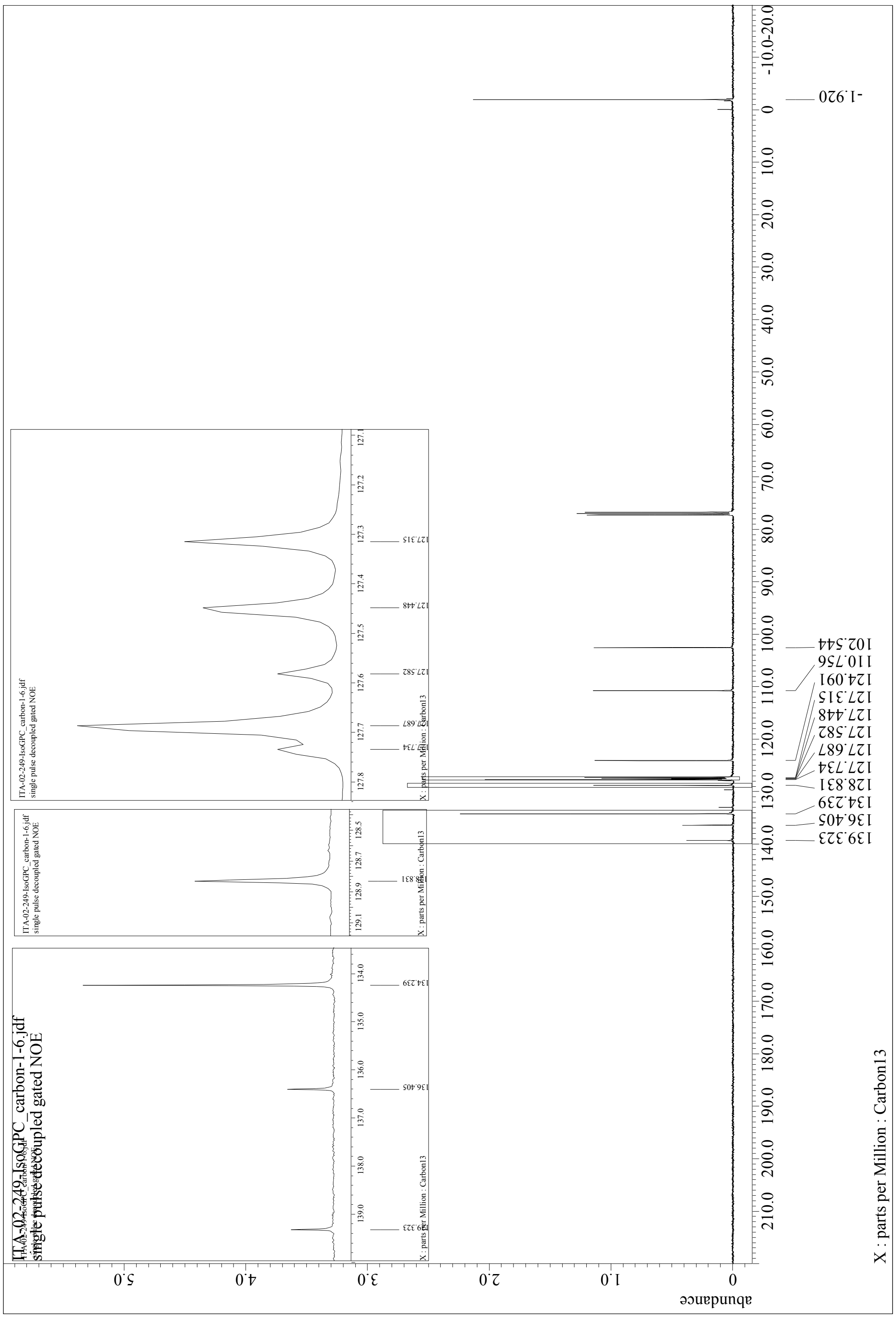

Supplementary Figure 35. ^{13}C NMR spectrum of **2l**

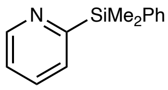

2m (^{1}H NMR, 500 MHz, $CDCl_3$)

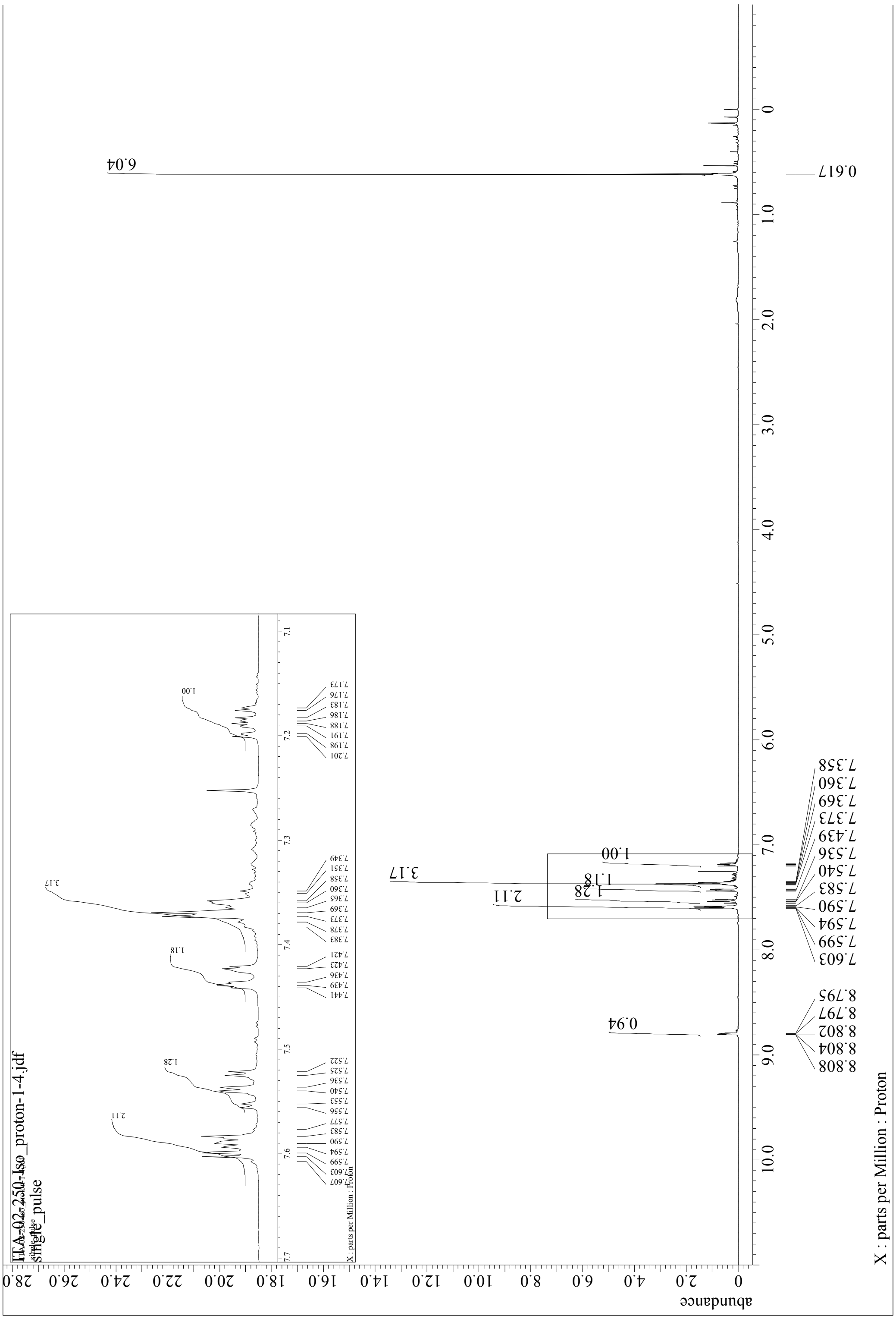

Supplementary Figure 36. ^{1}H NMR spectrum of **2m**

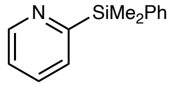

2m (^{13}C NMR, 126 MHz, $CDCl_3$)

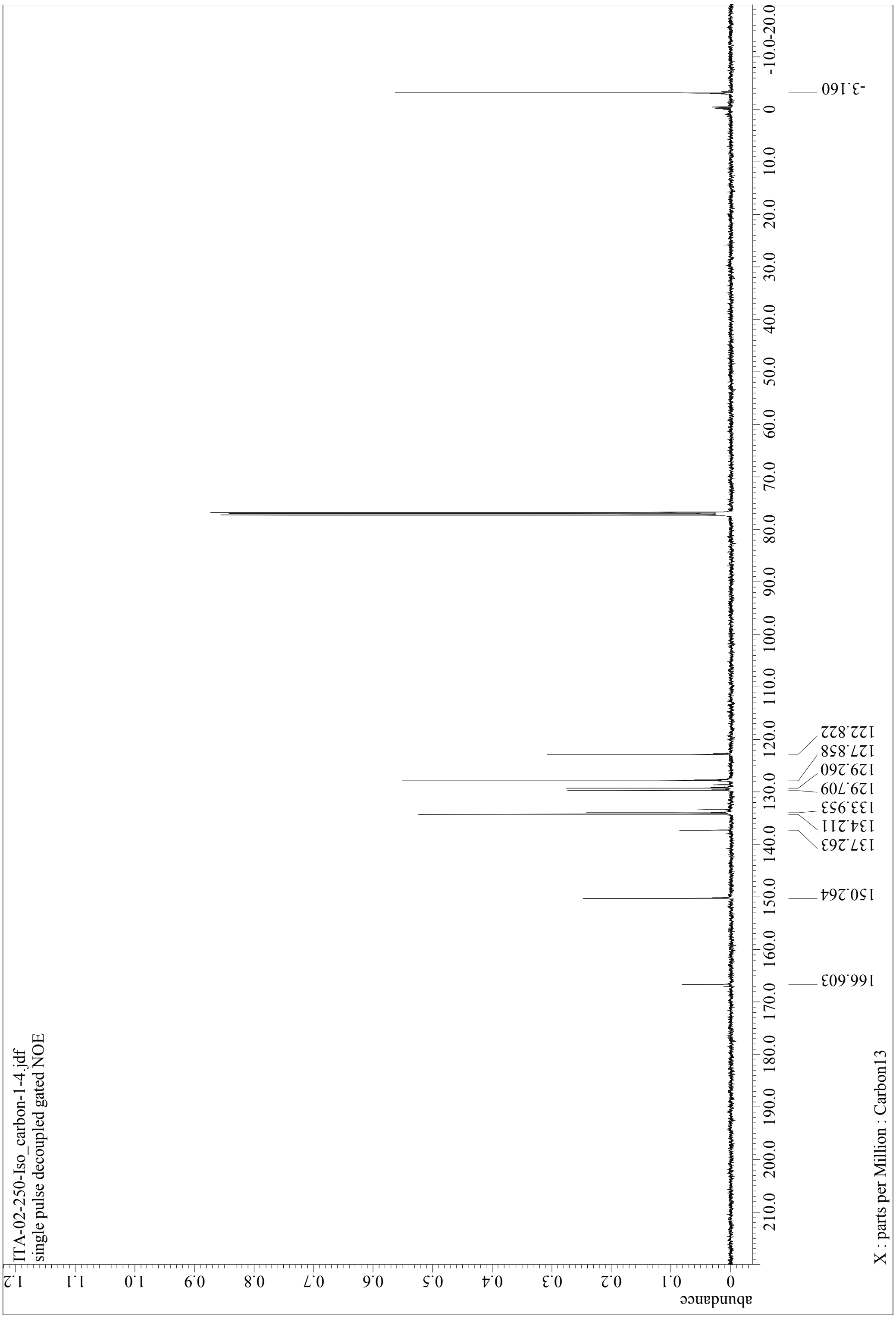

Supplementary Figure 37. ^{13}C NMR spectrum of **2m**

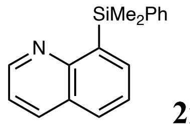

2n (^{1}H NMR, 500 MHz, $CDCl_3$)

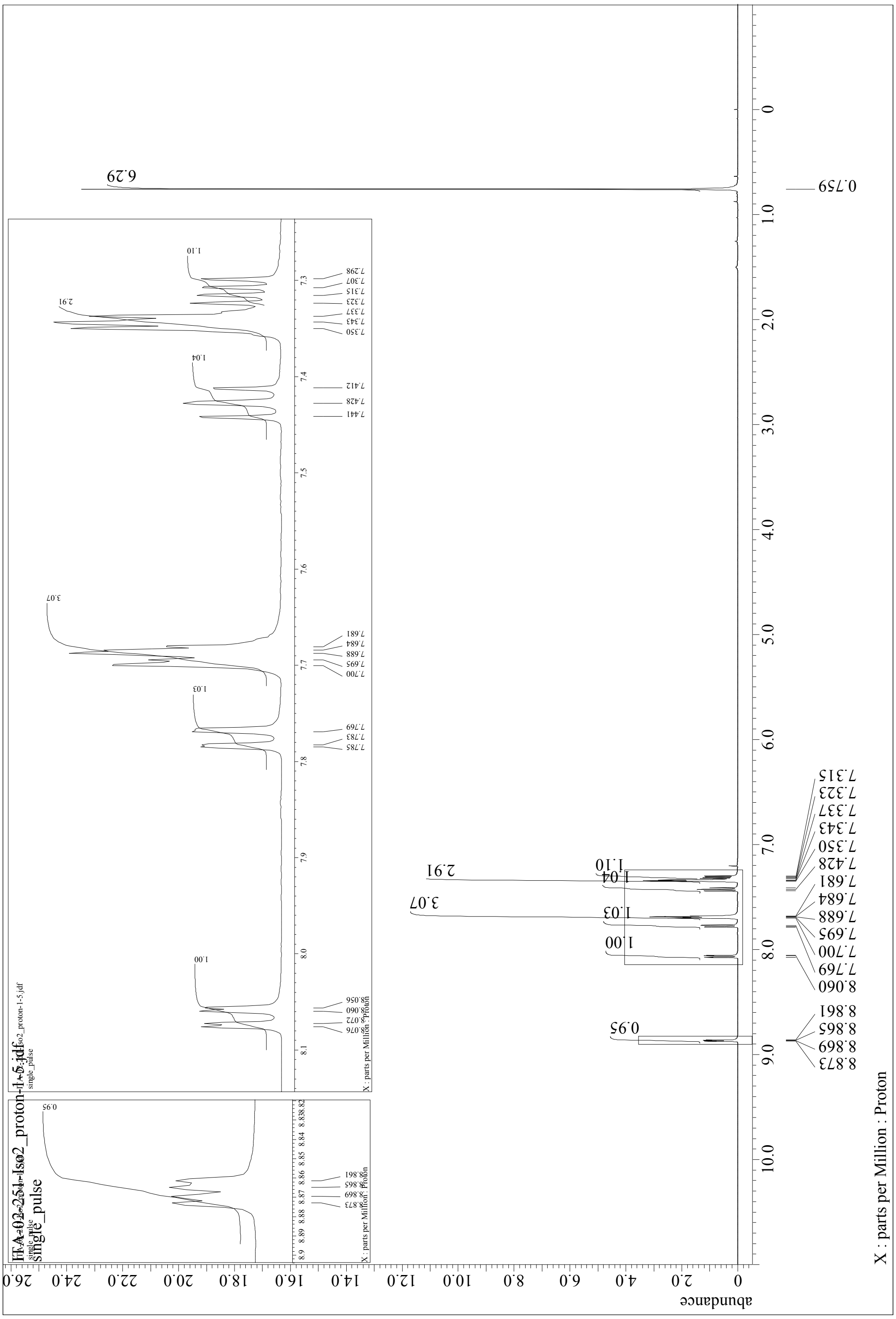

Supplementary Figure 38. ^{1}H NMR spectrum of **2n**

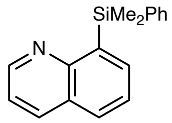

2n (^{13}C NMR, 126 MHz, $CDCl_3$)

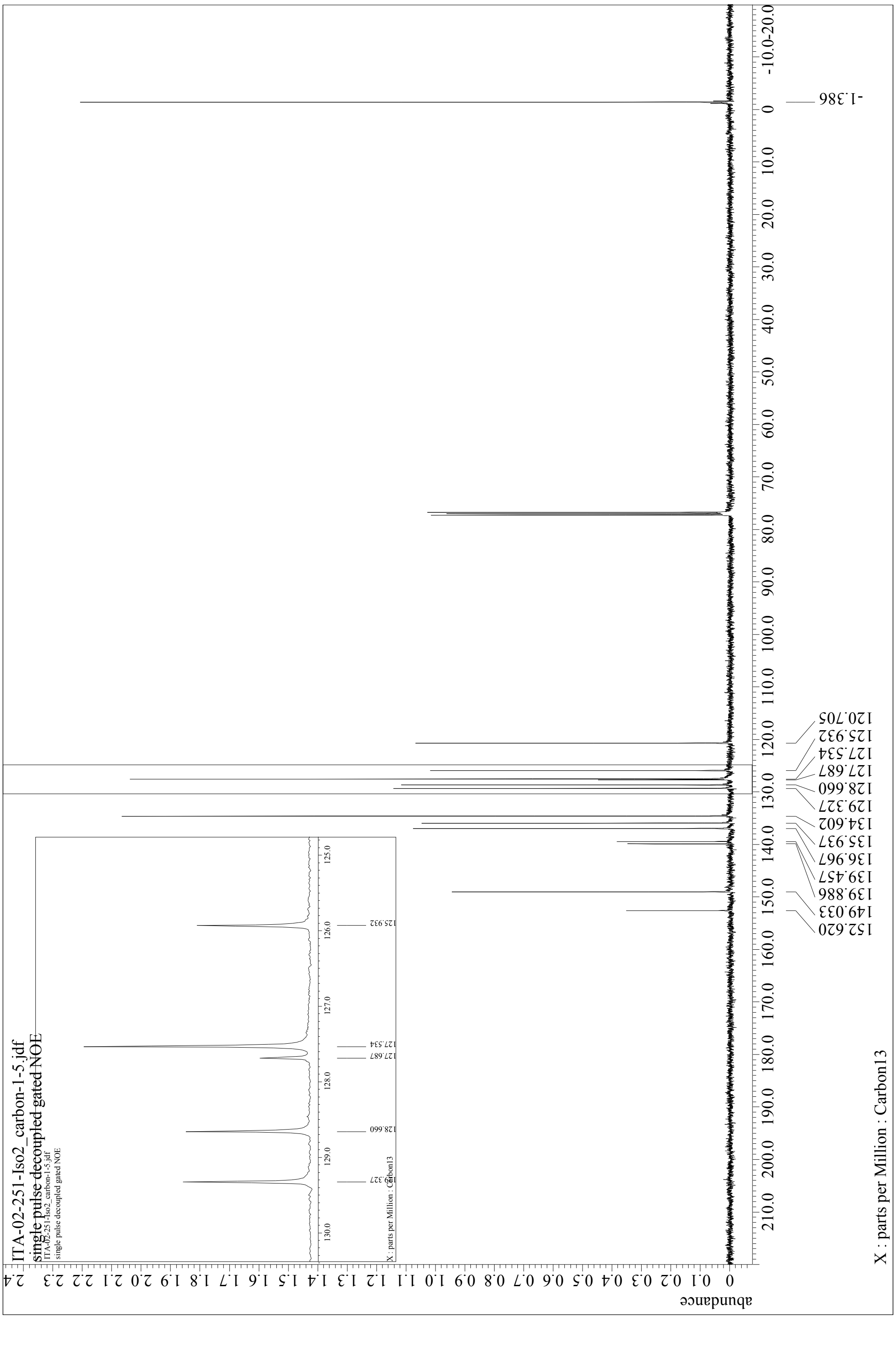

Supplementary Figure 39. ^{13}C NMR spectrum of **2n**

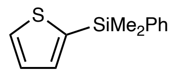

2o (^{1}H NMR, 500 MHz, $CDCl_3$)

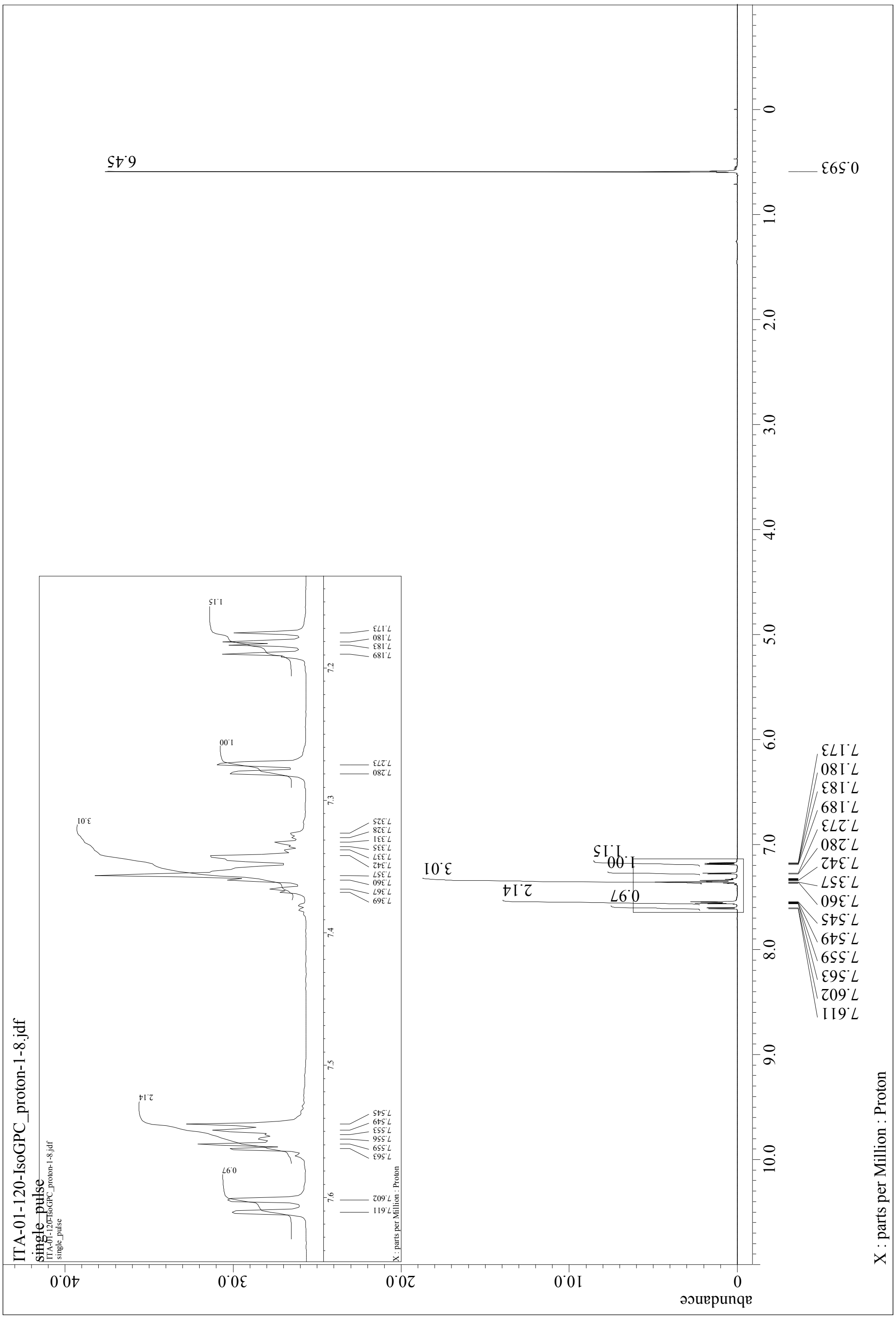

Supplementary Figure 40. ^{1}H NMR spectrum of **2o**

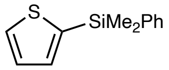

2o (^{13}C NMR, 126 MHz, $CDCl_3$)

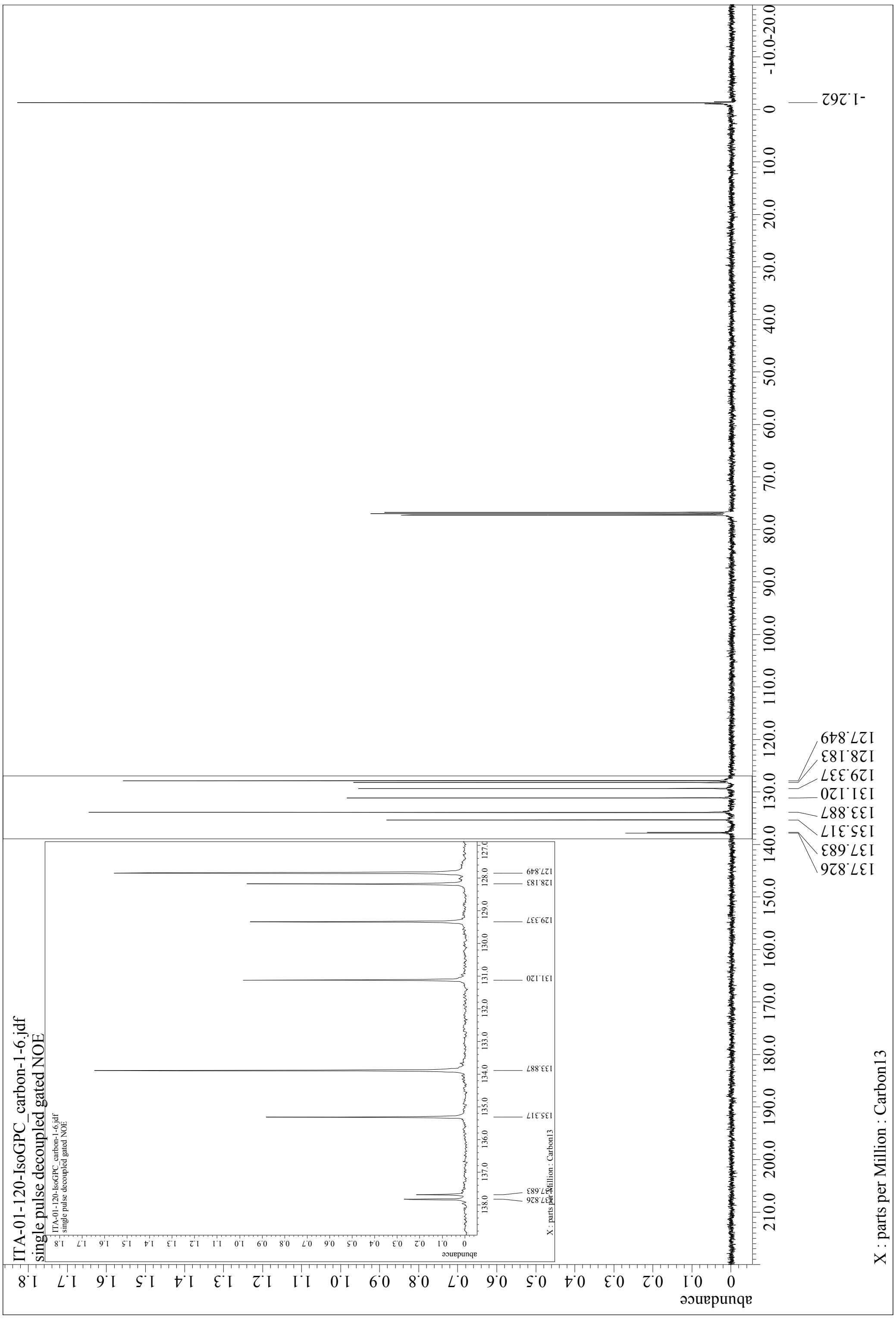

Supplementary Figure 41. ^{13}C NMR spectrum of **2o**

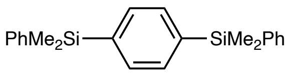

2p (^{1}H NMR, 500 MHz, $CDCl_3$)

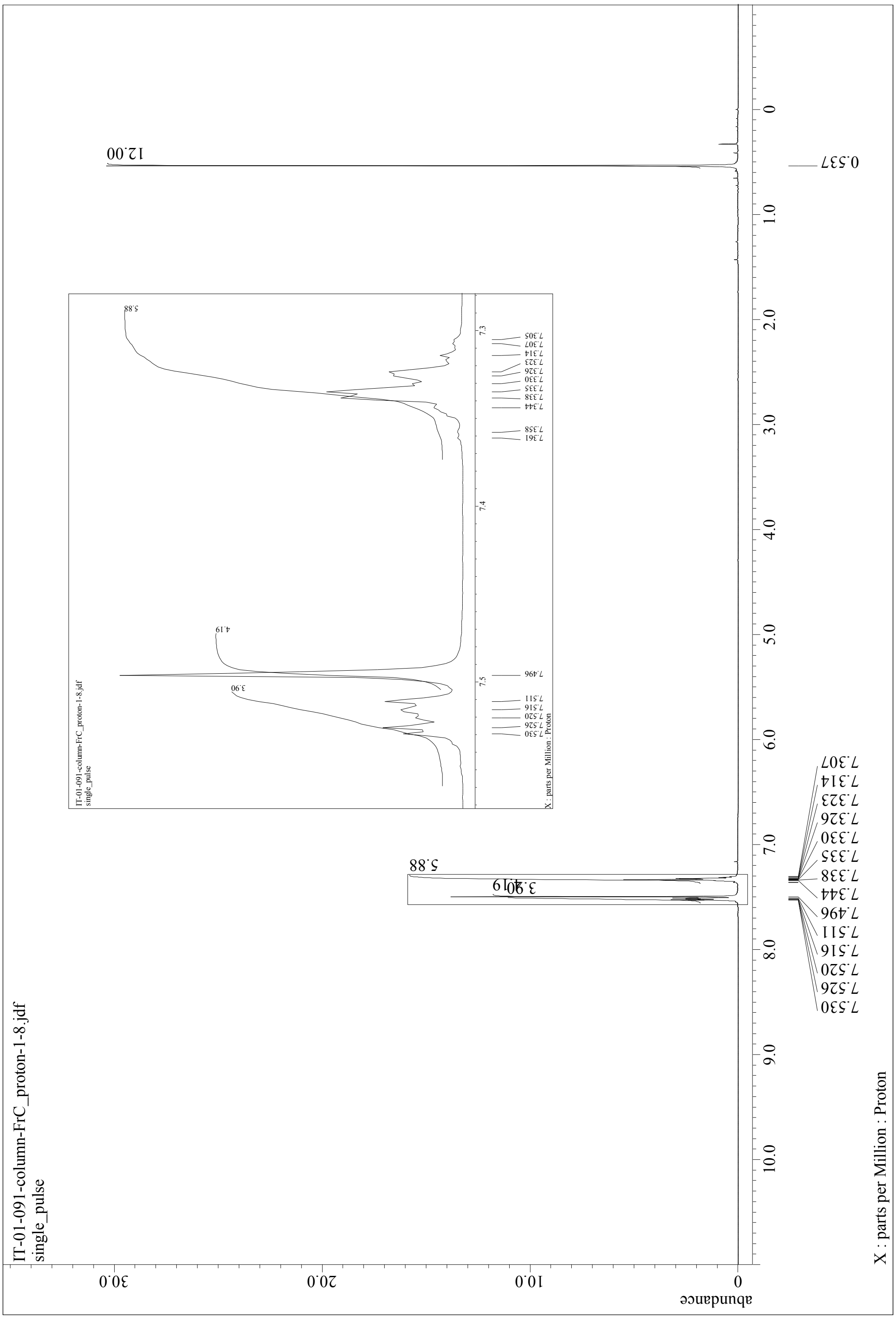

Supplementary Figure 42. ^{1}H NMR spectrum of **2p**

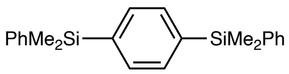

2p (^{13}C NMR, 126 MHz, $CDCl_3$)

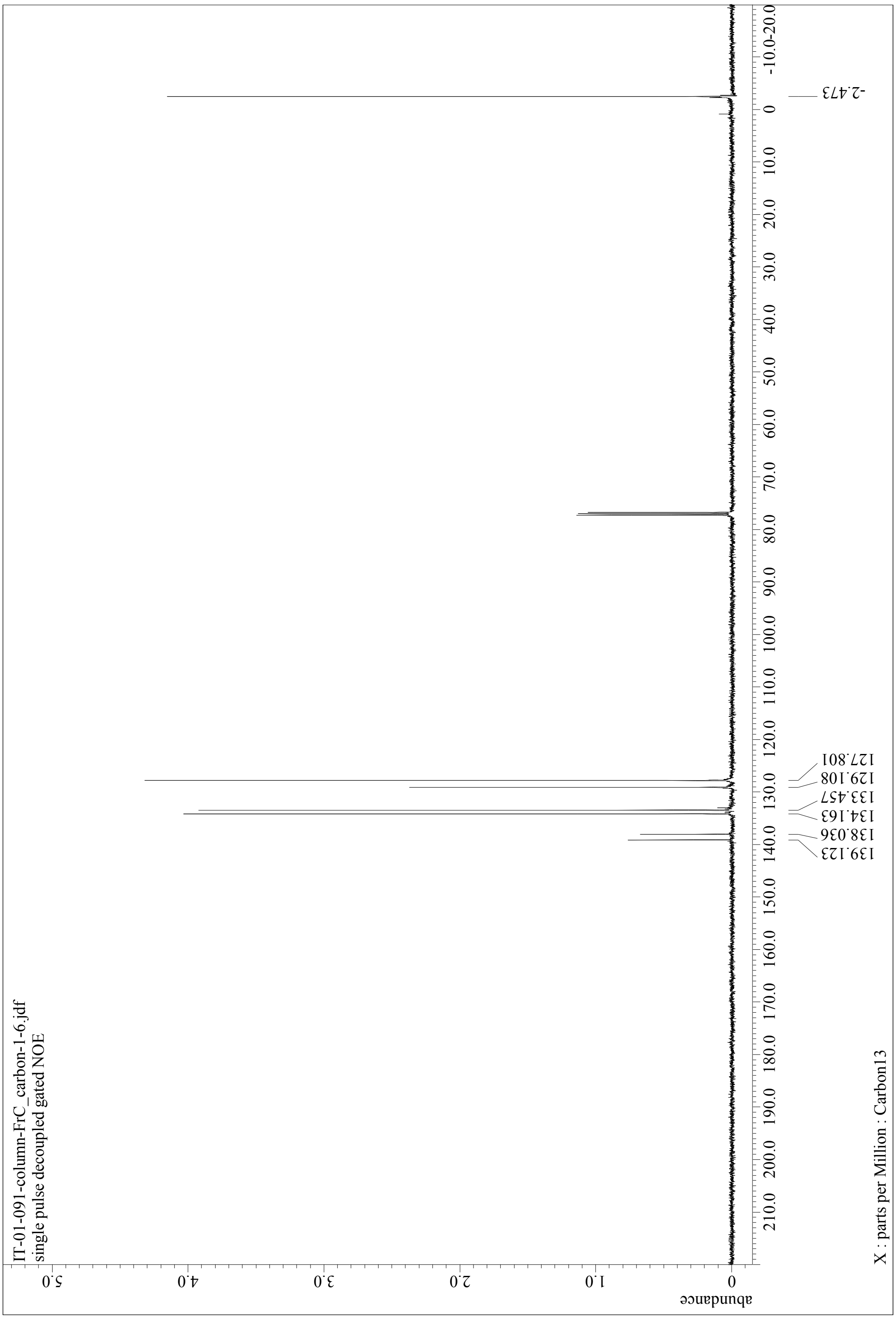

Supplementary Figure 43. ^{13}C NMR spectrum of **2p**

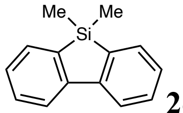

2q (^{1}H NMR, 500 MHz, $CDCl_3$)

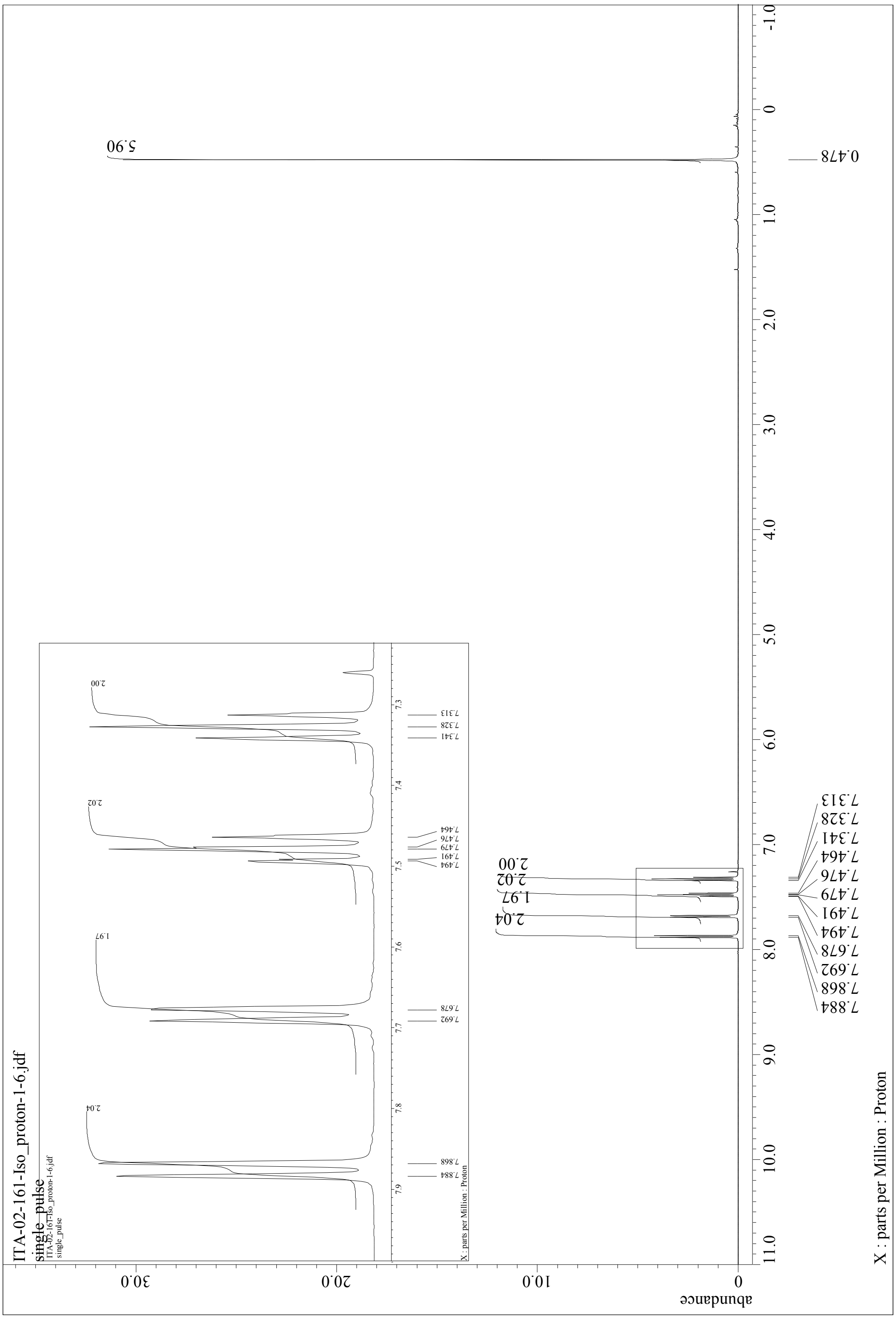

Supplementary Figure 44. ^{1}H NMR spectrum of **2q**

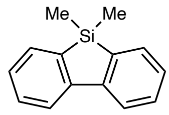

2q (^{13}C NMR, 126 MHz, $CDCl_3$)

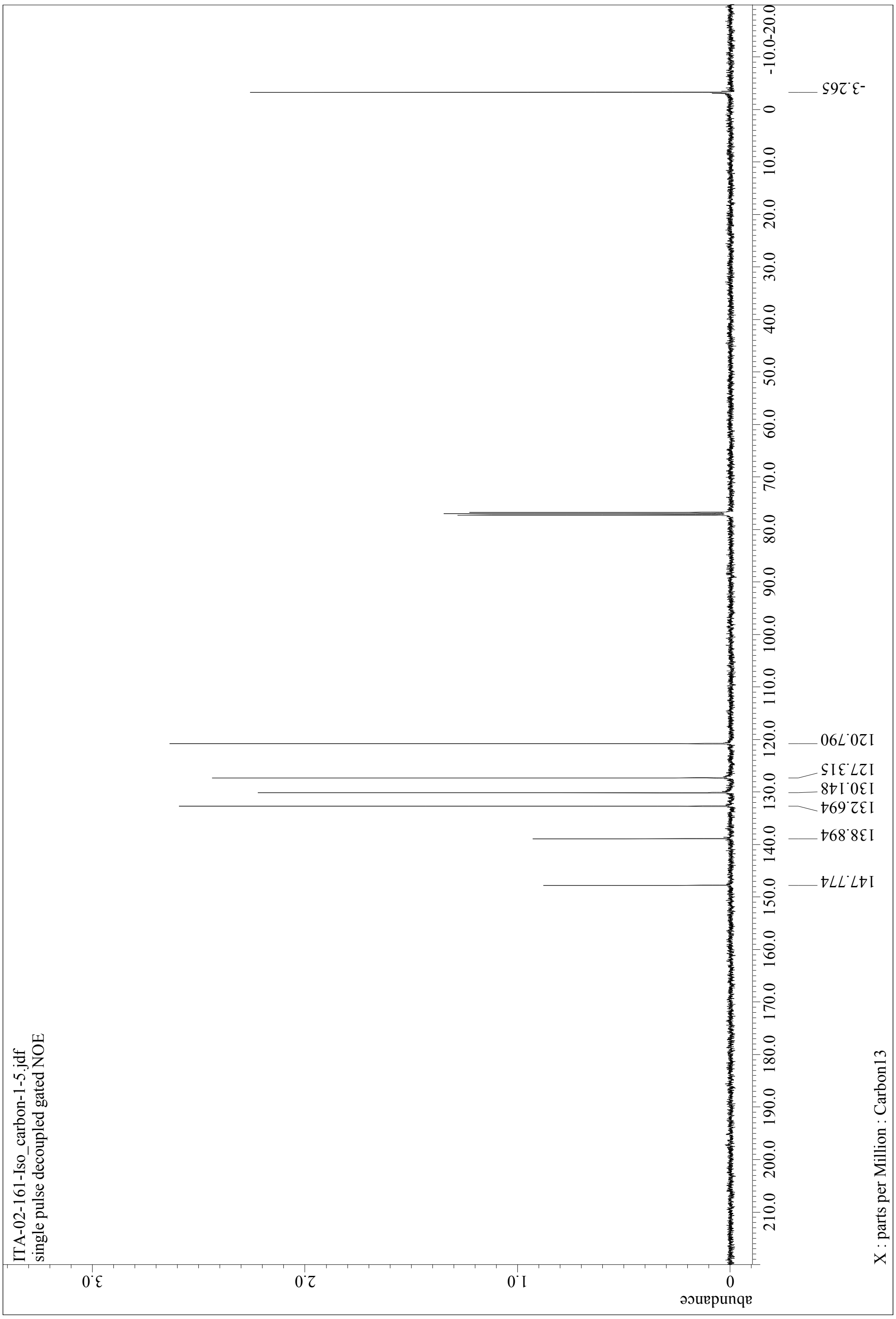

Supplementary Figure 45. ^{13}C NMR spectrum of **2q**

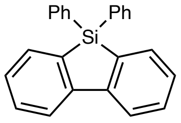

2r (^{1}H NMR, 500 MHz, $CDCl_3$)

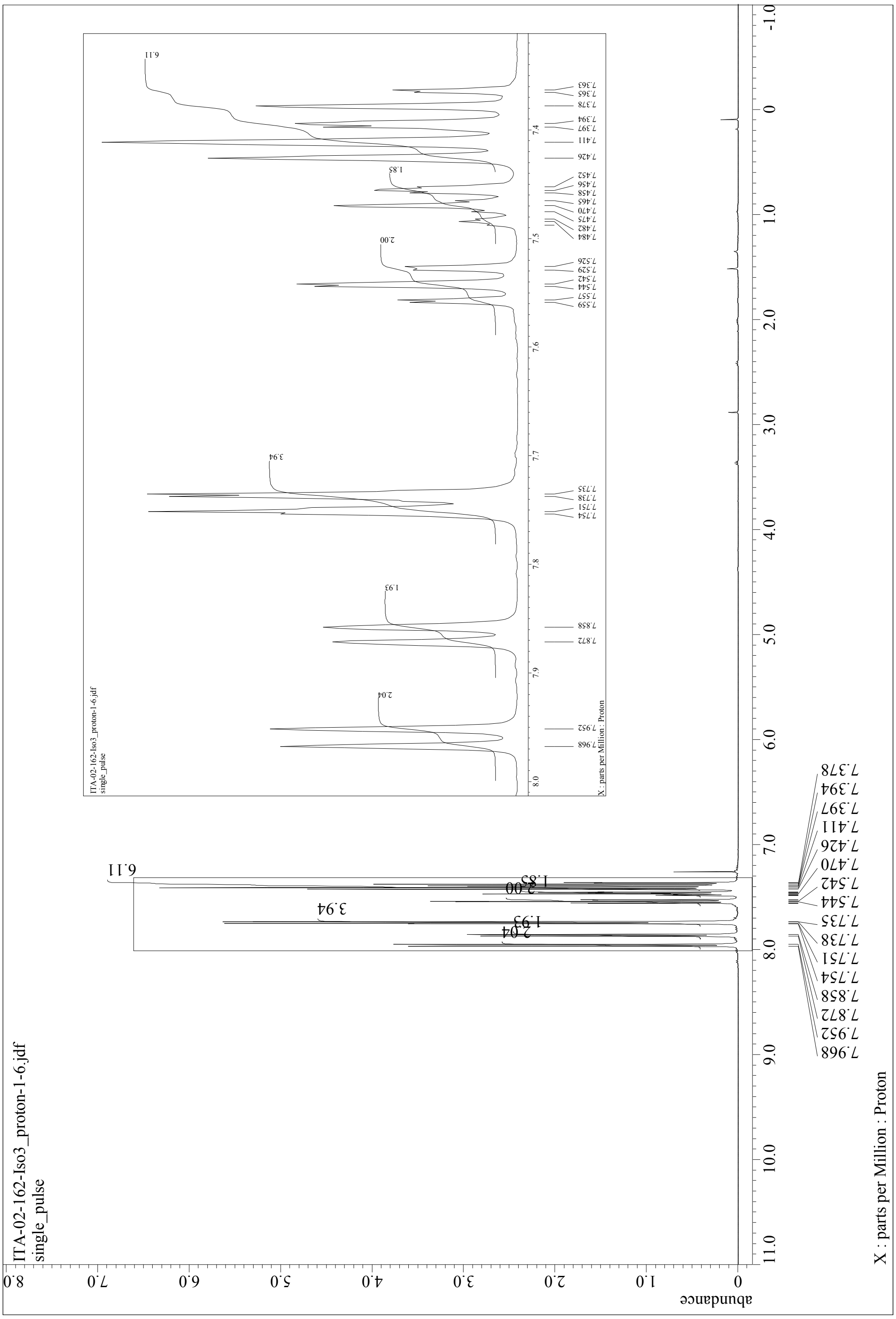

Supplementary Figure 46. ^{1}H NMR spectrum of **2r**

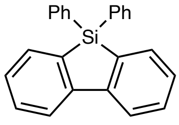

2r (^{13}C NMR, 126 MHz, $CDCl_3$)

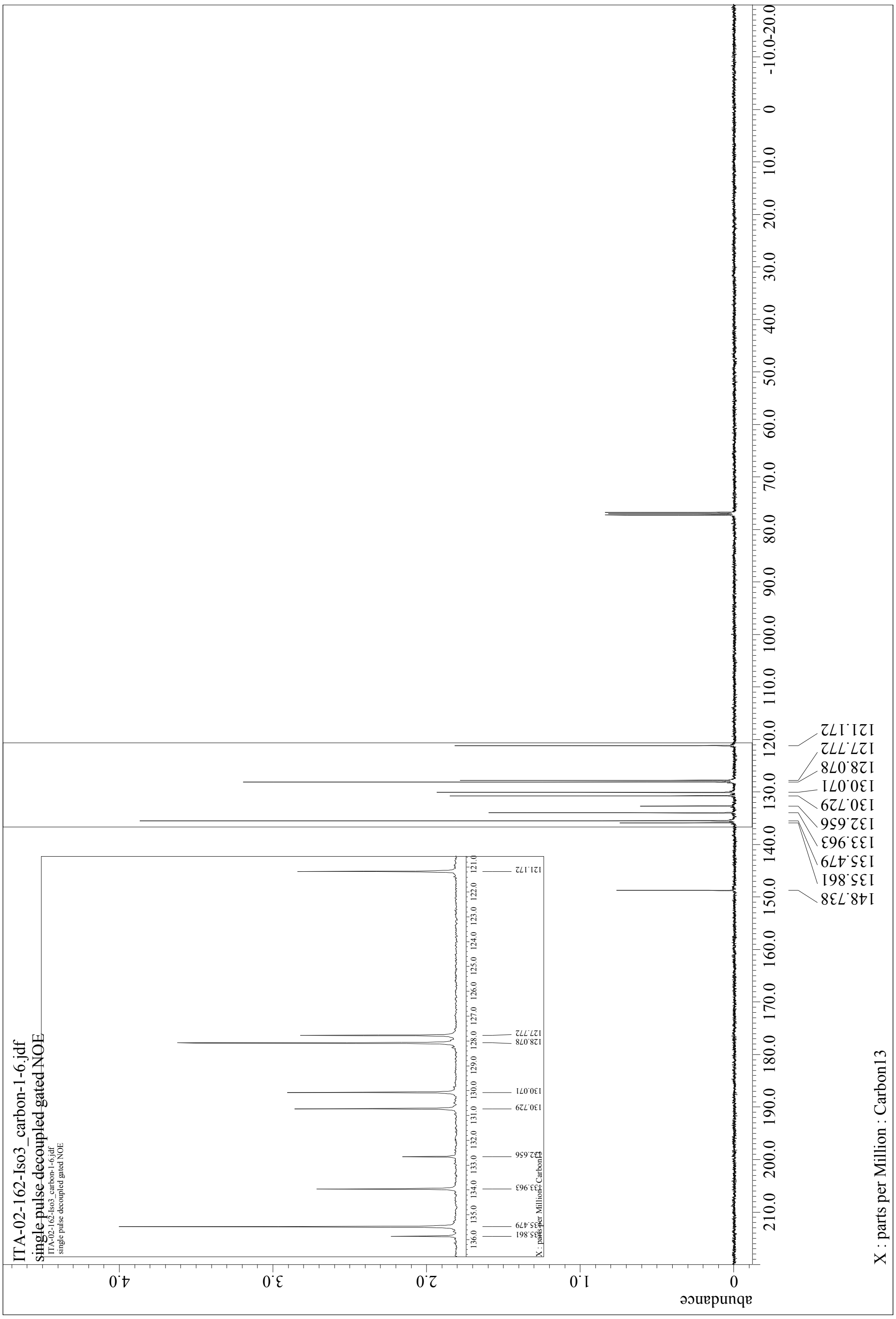

Supplementary Figure 47. ^{13}C NMR spectrum of **2r**

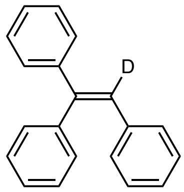

2s (^{1}H NMR, 500 MHz, $CDCl_3$)

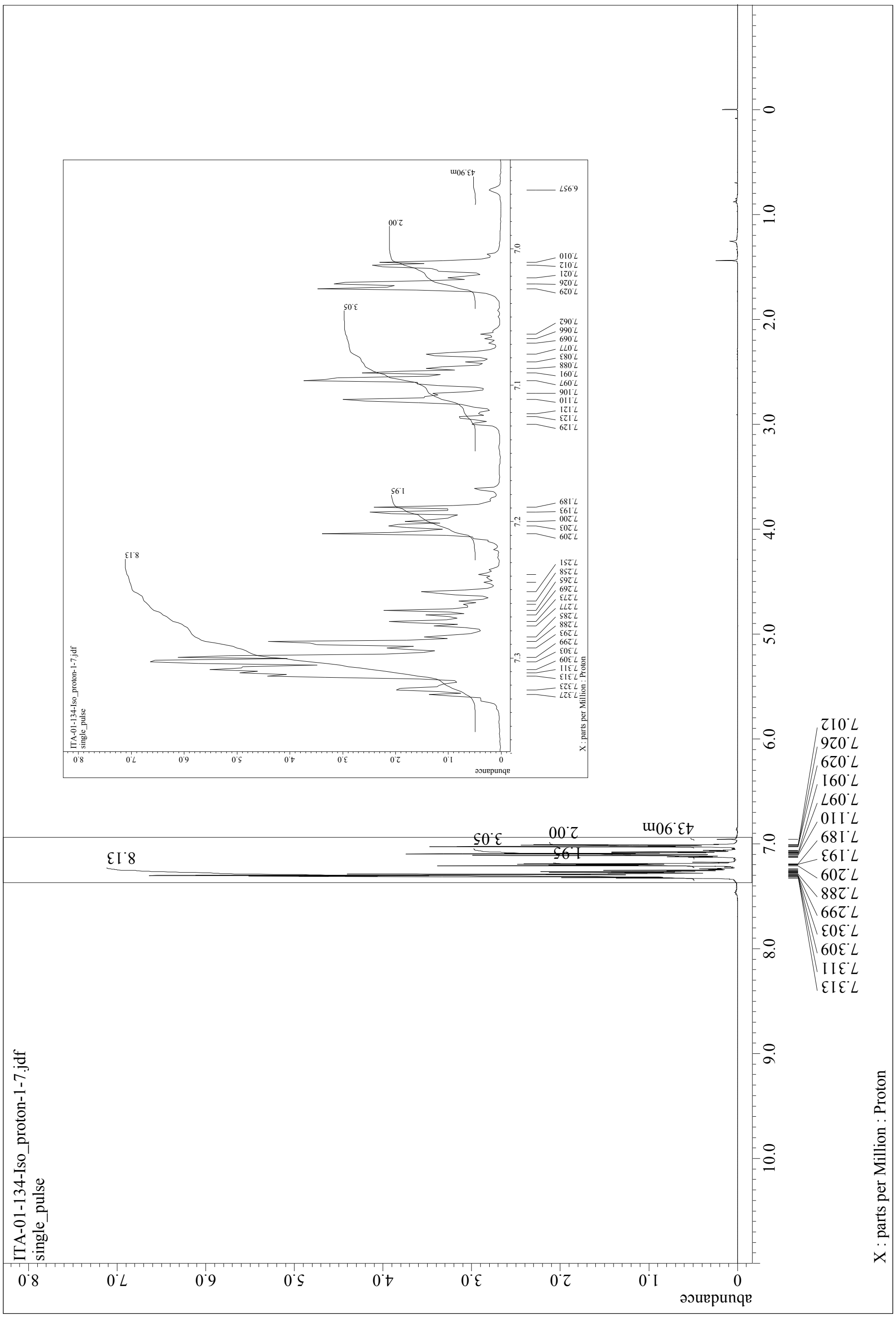

Supplementary Figure 48. ^{1}H NMR spectrum of **2s**

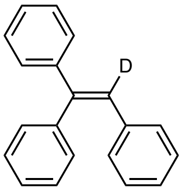

2s (^{13}C NMR, 126 MHz, $CDCl_3$)

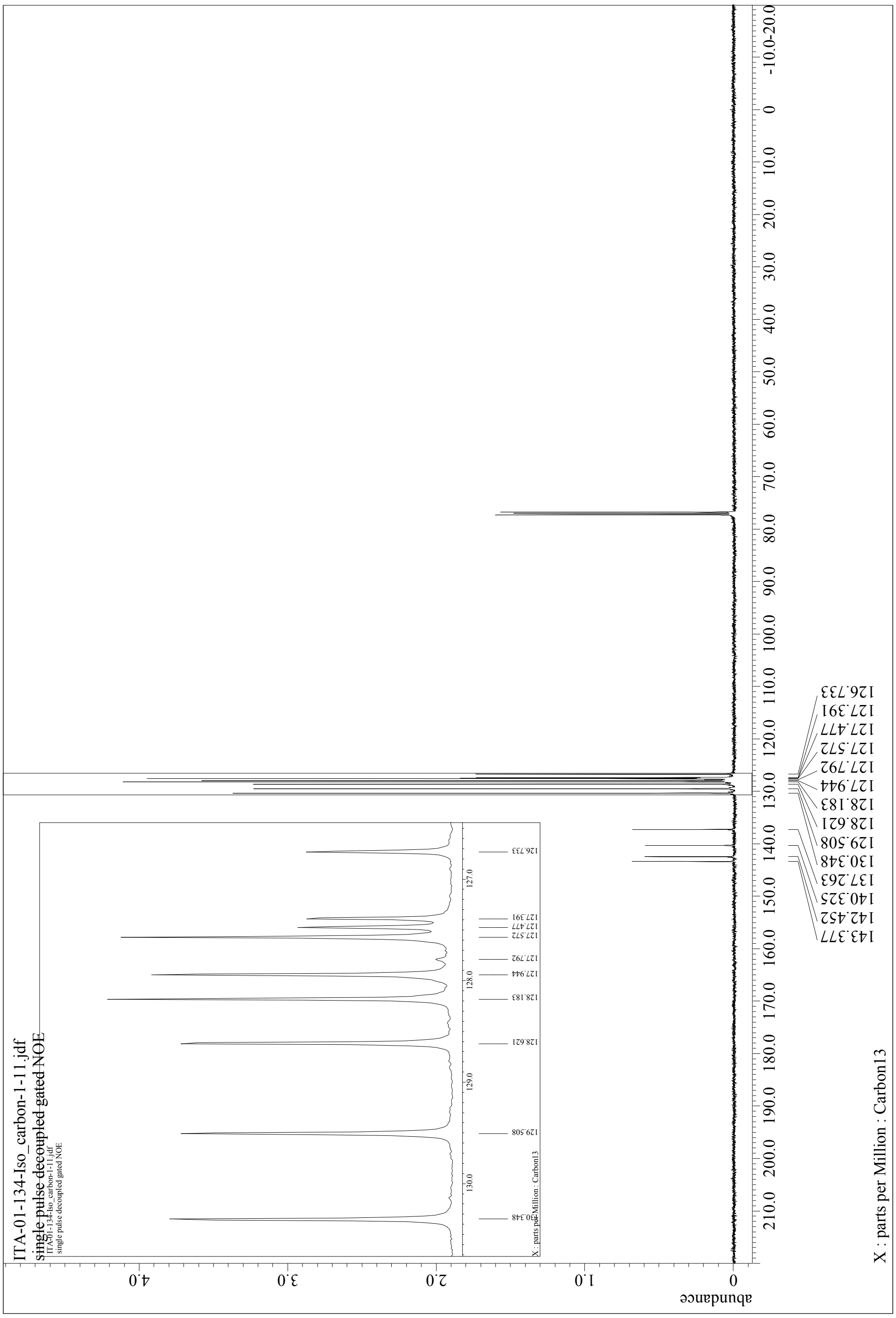

Supplementary Figure 49. ^{13}C NMR spectrum of **2s**

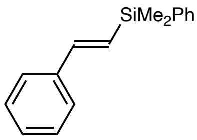

2t (*E*/*Z* = >99:1) (^{1}H NMR, 500 MHz, $CDCl_3$)

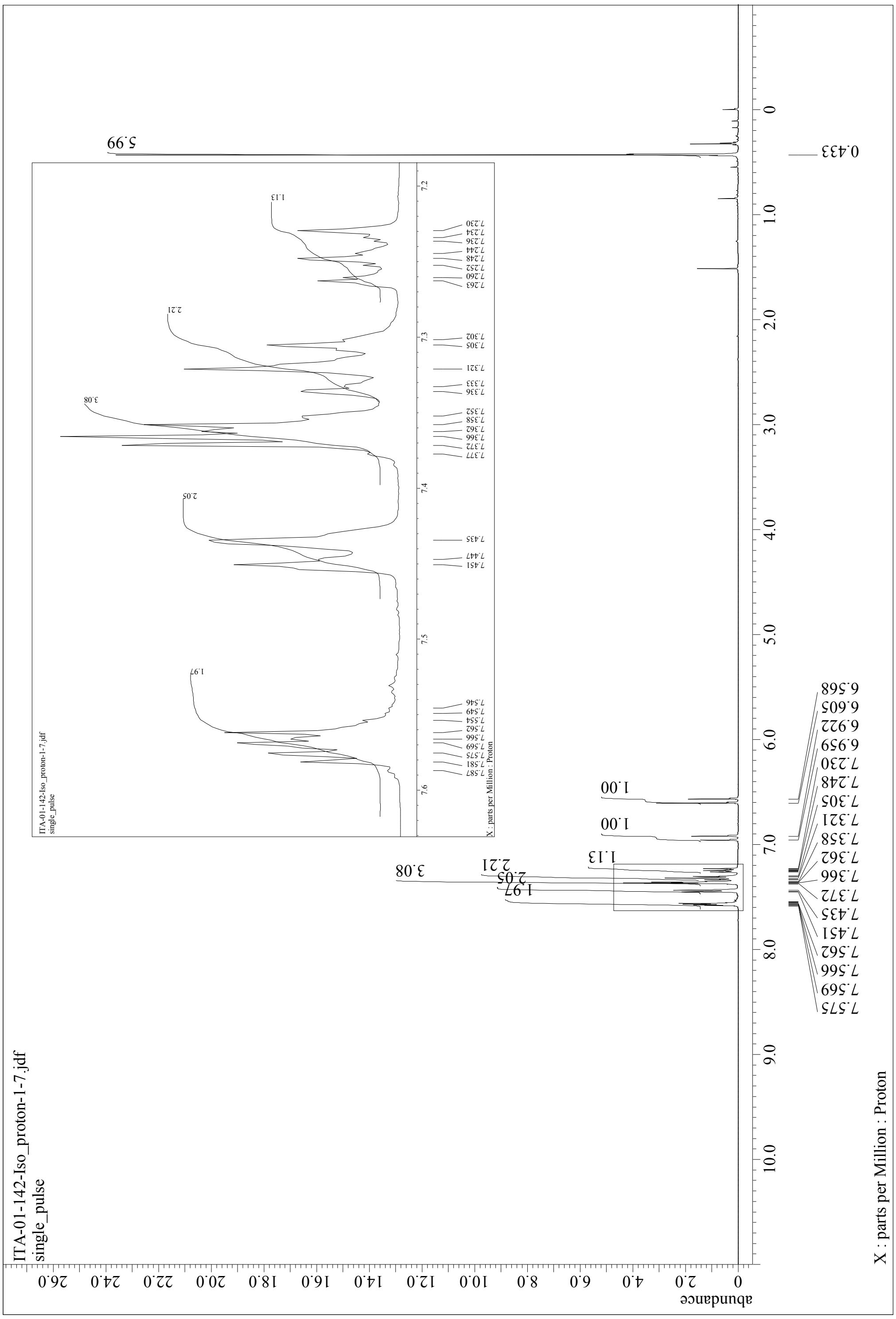

Supplementary Figure 50. ^{1}H NMR spectrum of **2t** (*E*/*Z* = >99:1)

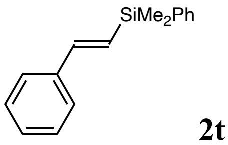

2t (*E*/*Z* = >99:1) (^{13}C NMR, 126 MHz, $CDCl_3$)

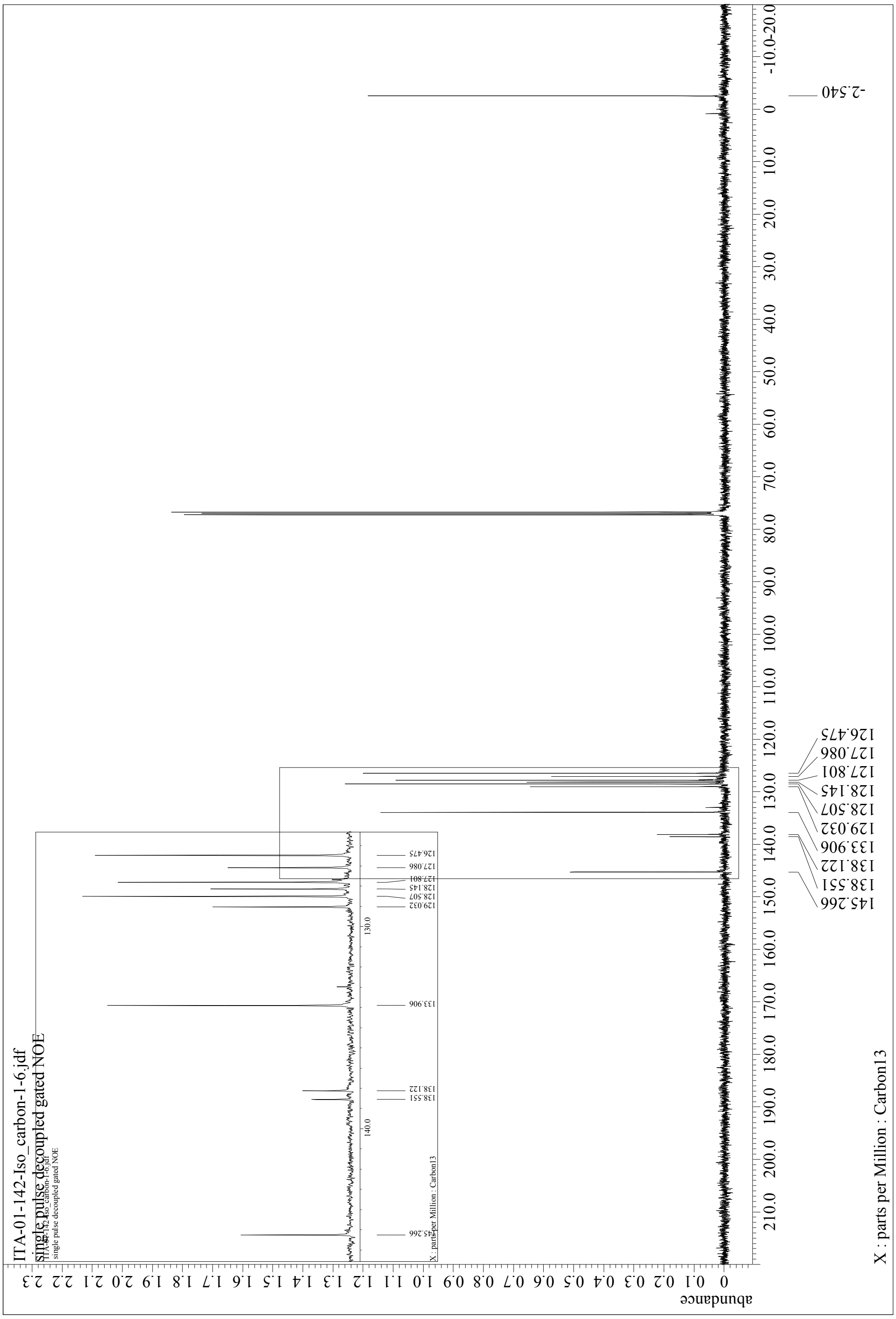

Supplementary Figure 51. ^{13}C NMR spectrum of **2t** (*E*/*Z* = >99:1)

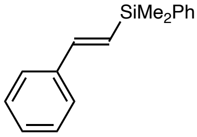

2t (E/Z = 86:14) (^{1}H NMR, 500 MHz, $CDCl_3$)

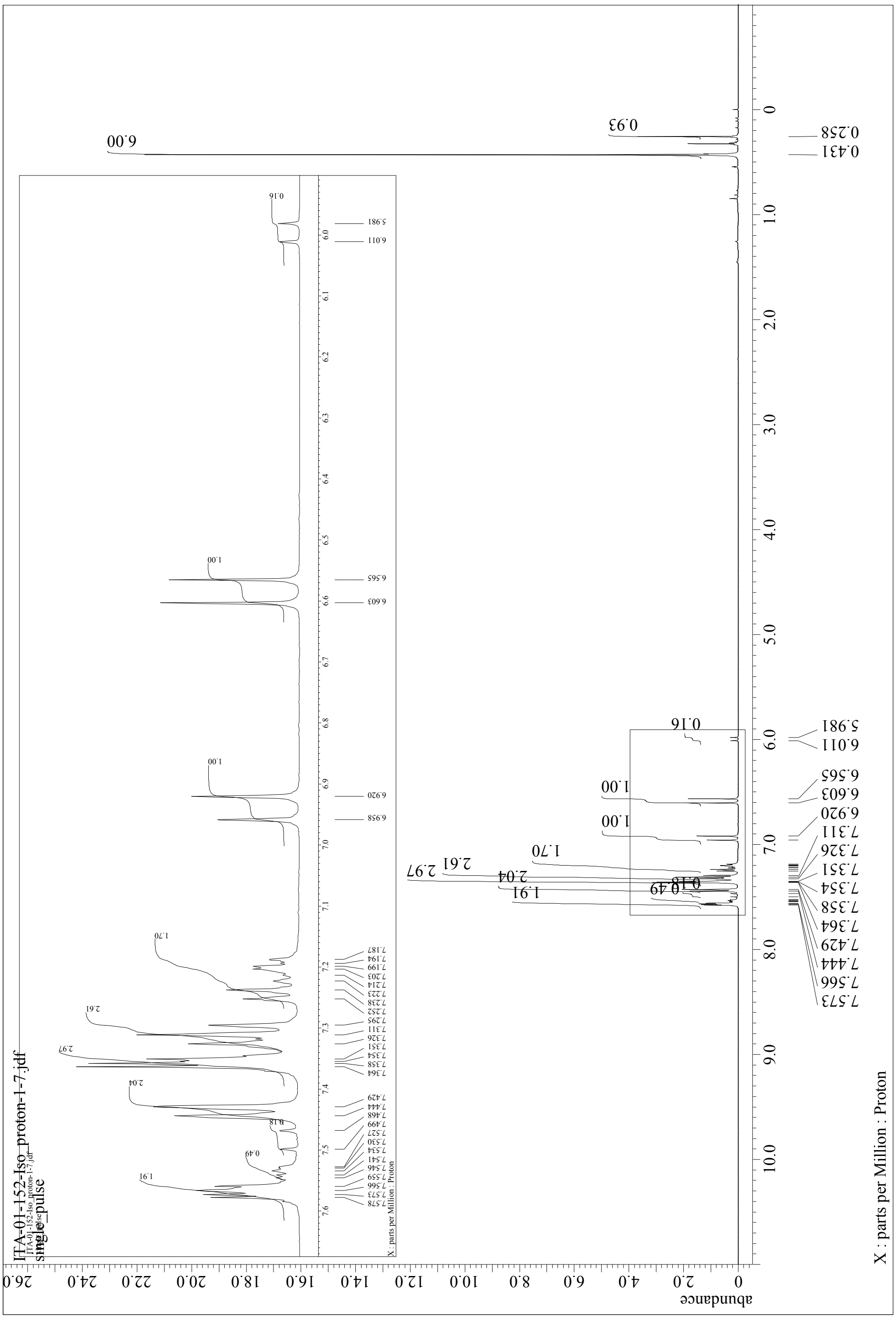

Supplementary Figure 52. ^{1}H NMR spectrum of **2t** (E/Z = 86:14)

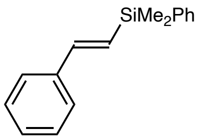

2t (E/Z = 86:14) (^{13}C NMR, 126 MHz, $CDCl_3$)

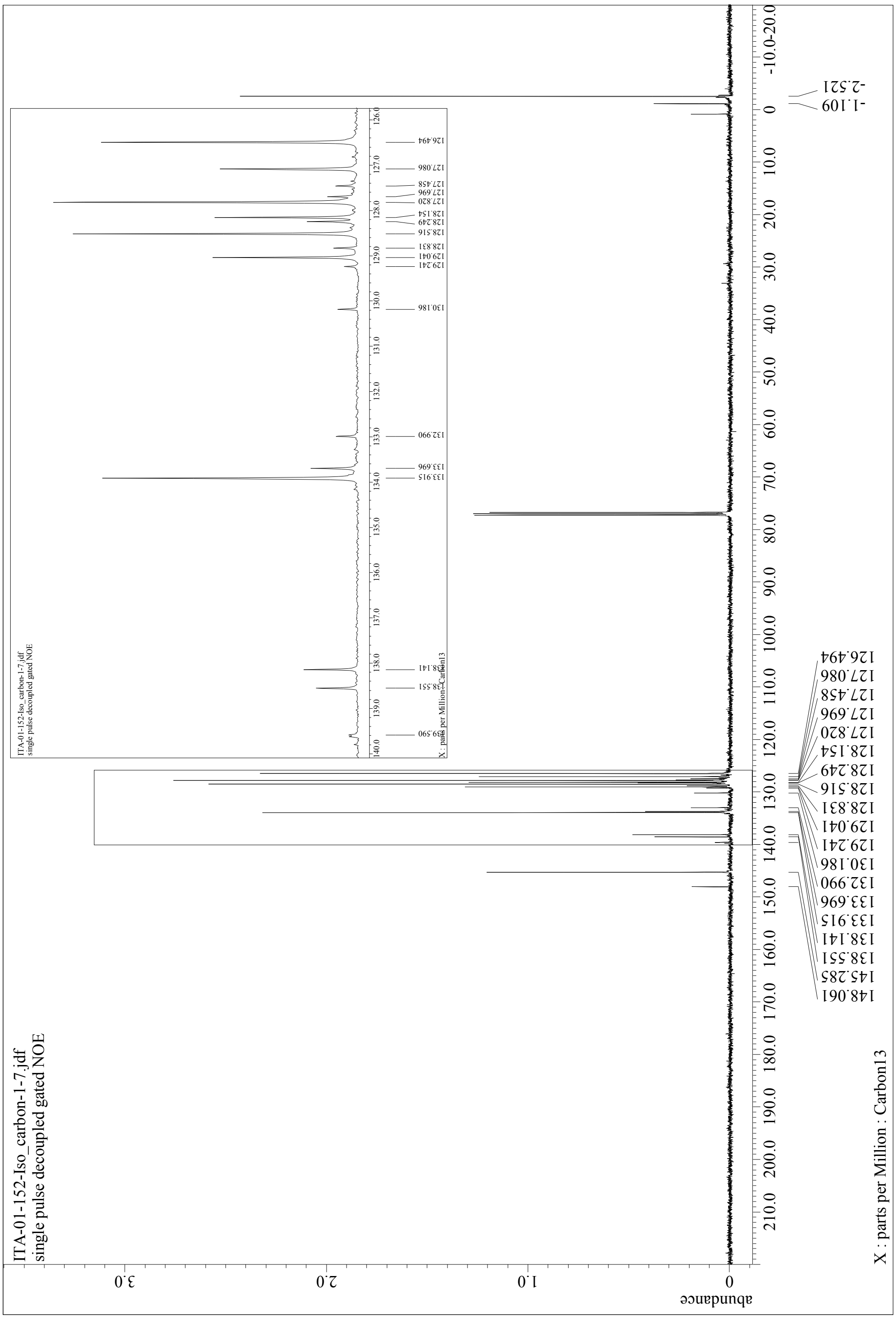

Supplementary Figure 53. ^{13}C NMR spectrum of **2t** (E/Z = 86:14)

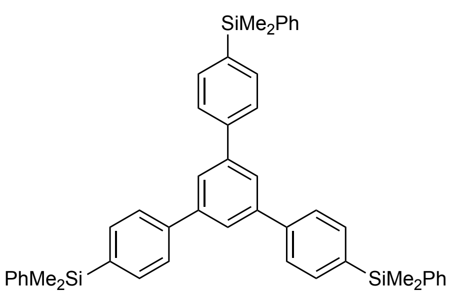

2u (^{1}H NMR, 500 MHz, $CDCl_3$)

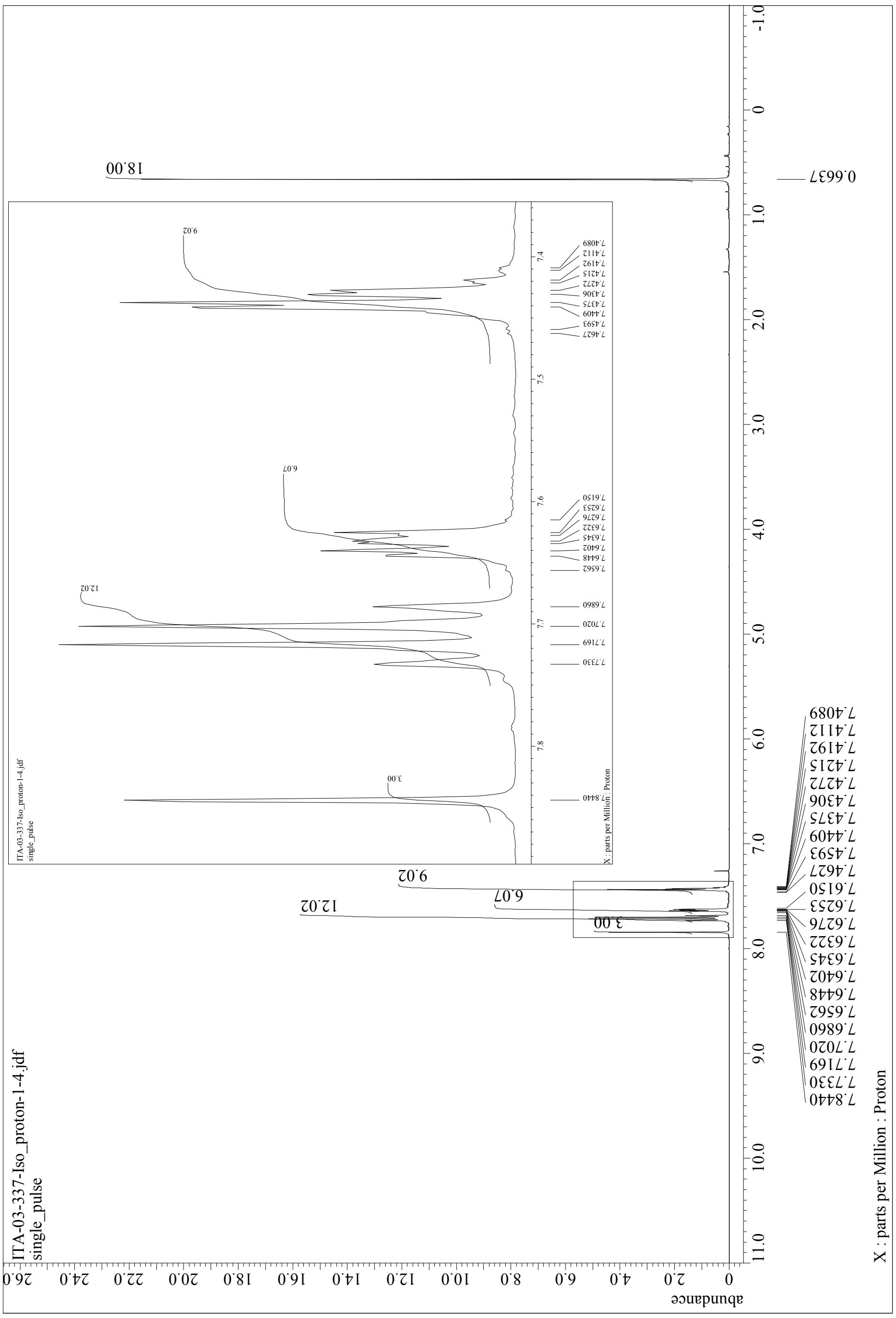

Supplementary Figure 54. ^{1}H NMR spectrum of **2u**

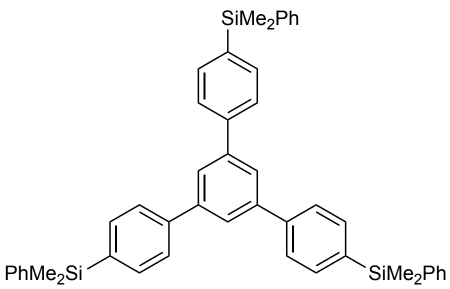

2u (^{13}C NMR, 126 MHz, $CDCl_3$)

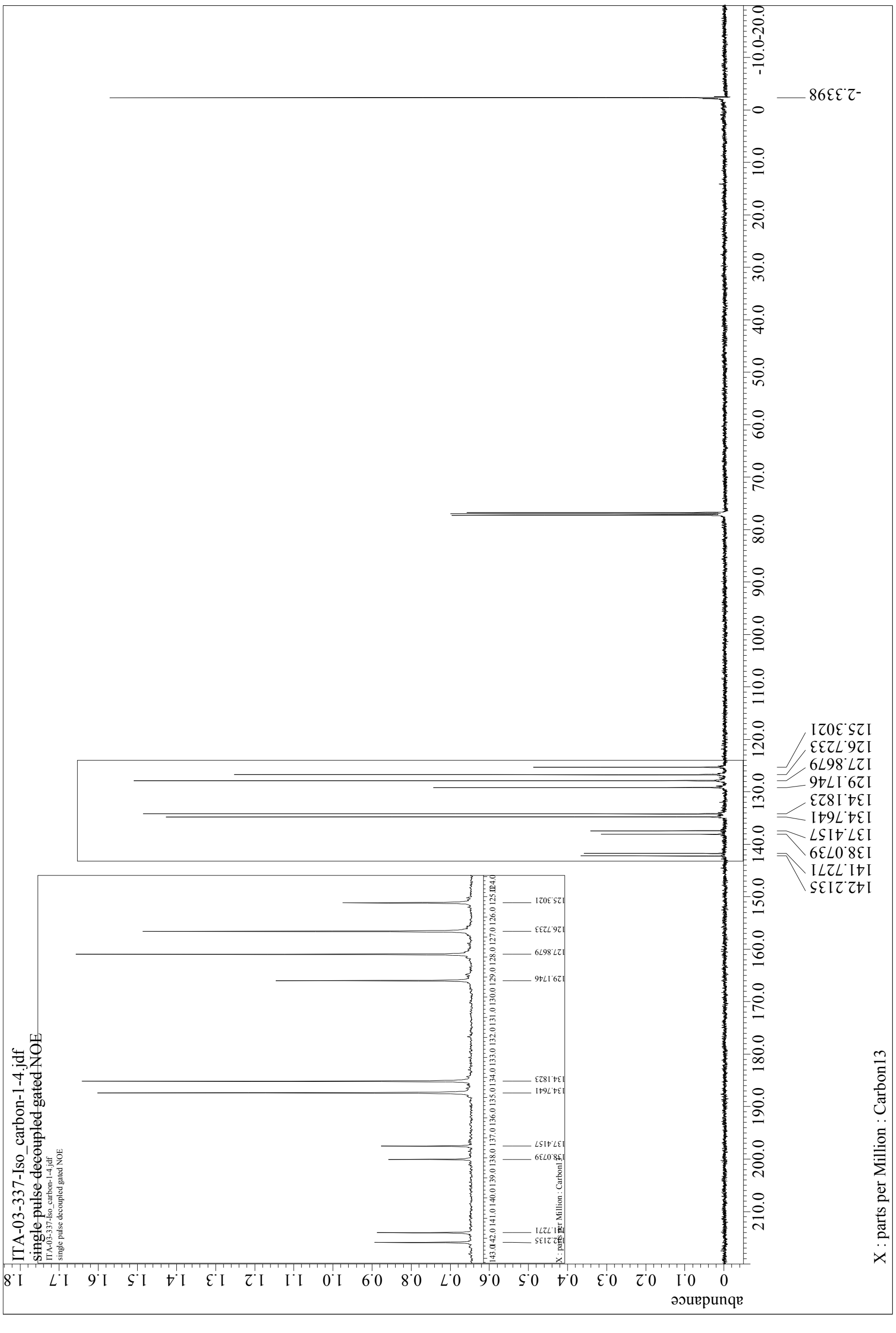

Supplementary Figure 55. ^{13}C NMR spectrum of **2u**

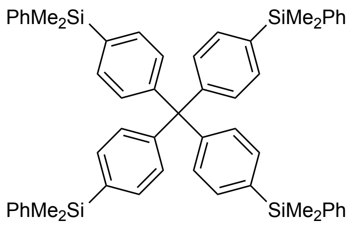

2v (^{1}H NMR, 500 MHz, $CDCl_3$)

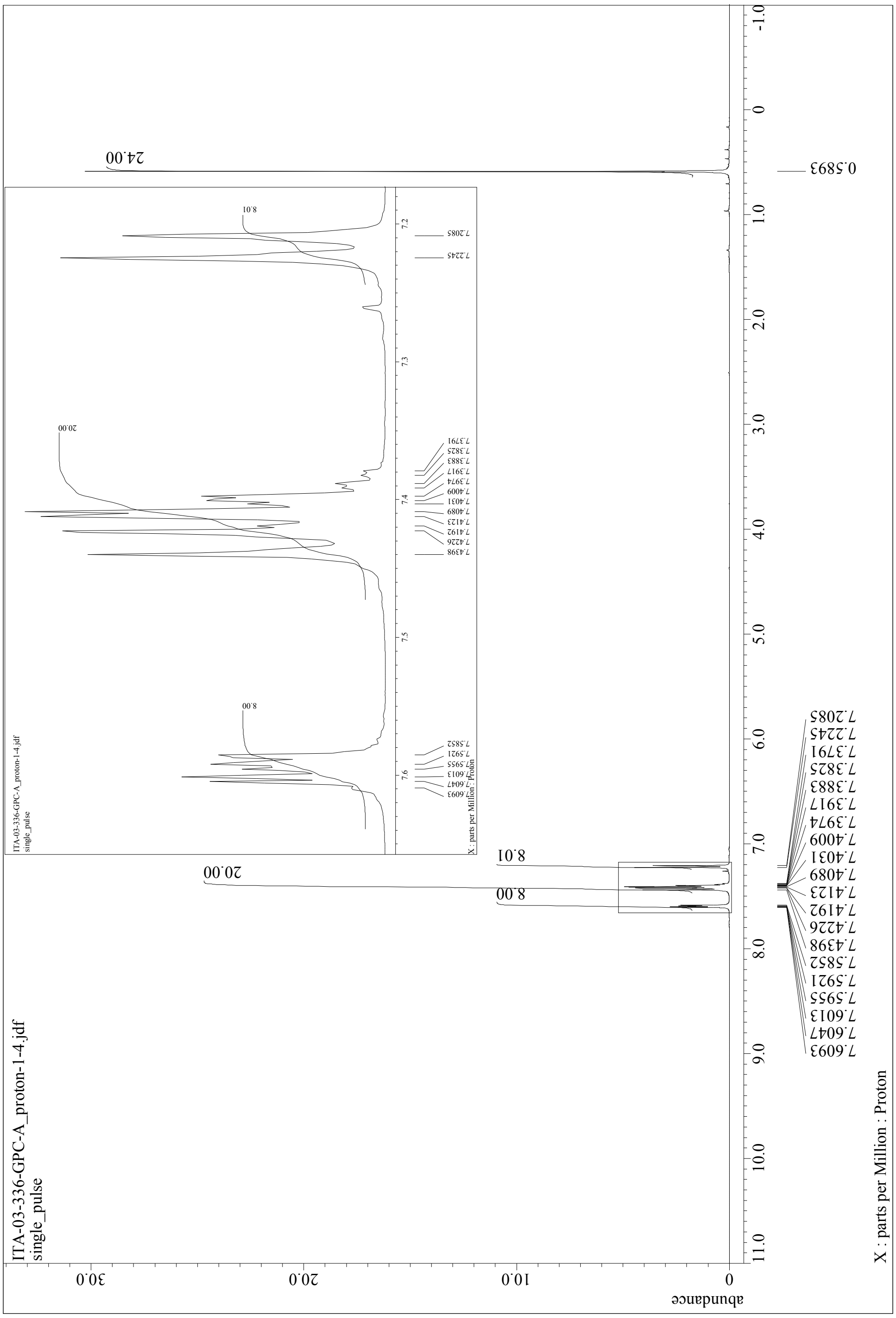

Supplementary Figure 56. ^{1}H NMR spectrum of **2v**

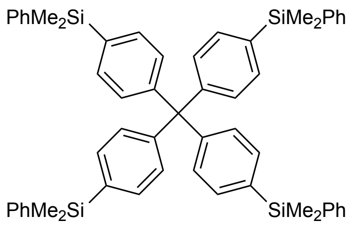

2v (^{13}C NMR, 126 MHz, $CDCl_3$)

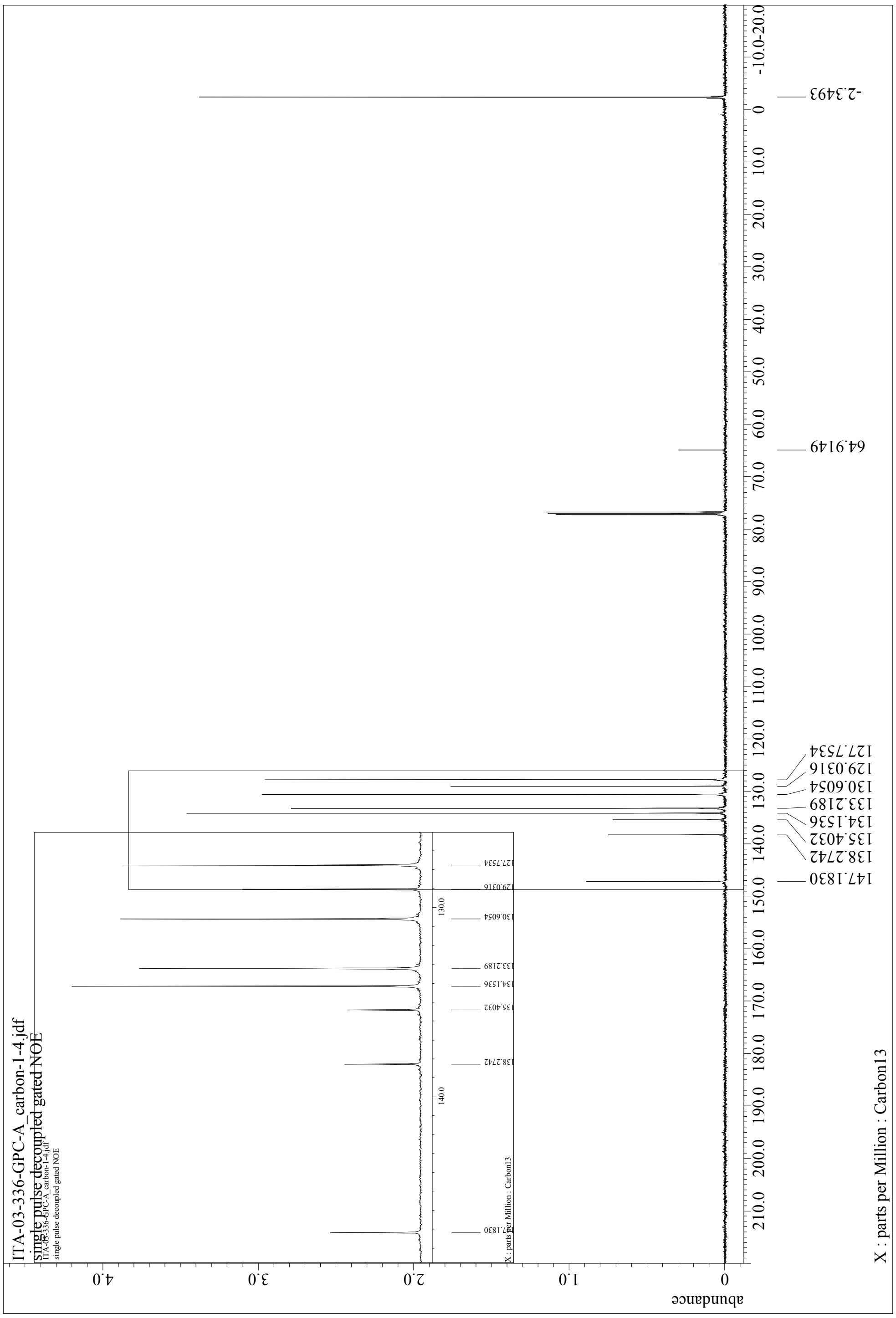

Supplementary Figure 57. ^{13}C NMR spectrum of **2v**

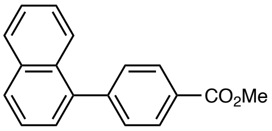

4a (^{1}H NMR, 500 MHz, $CDCl_3$)

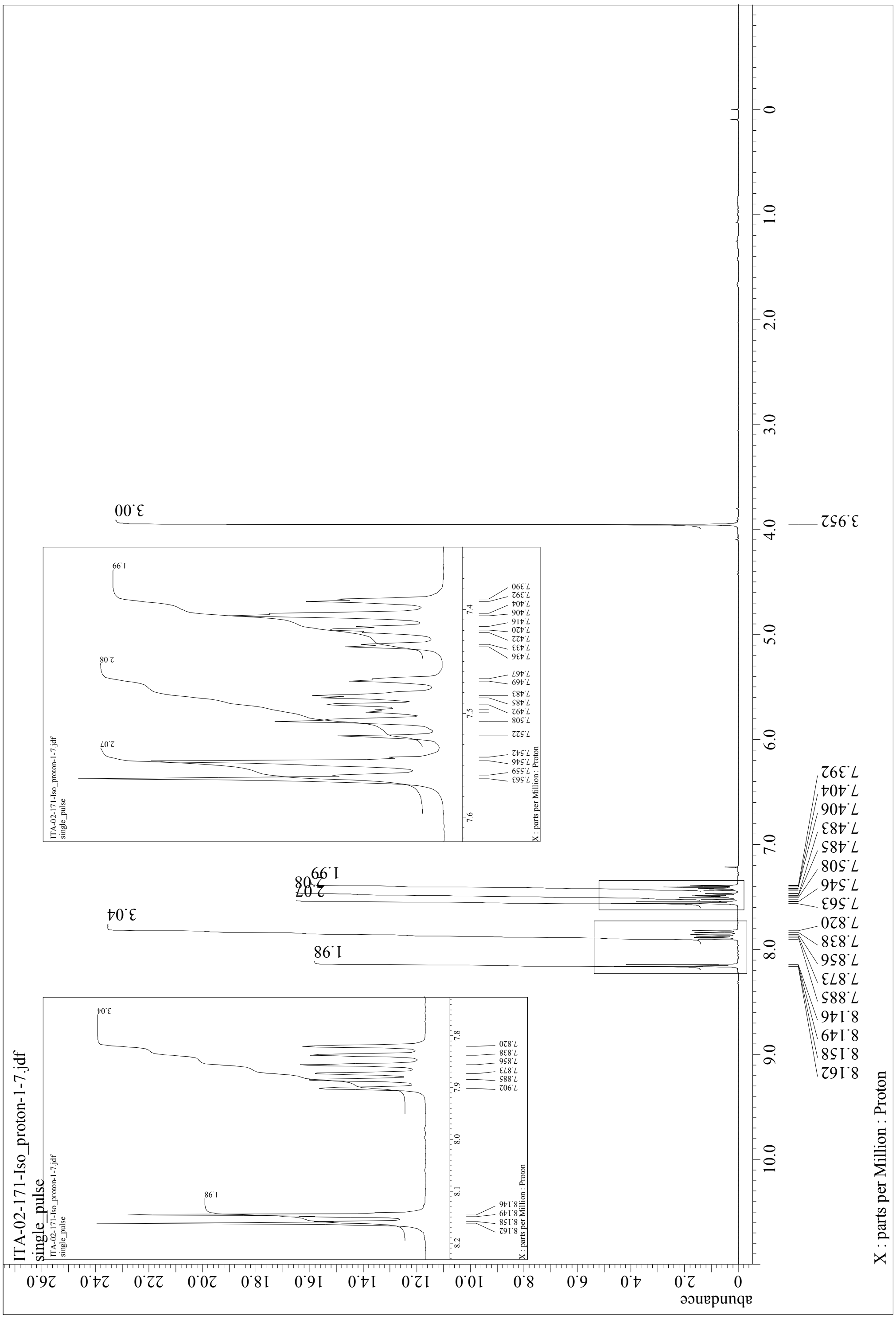

Supplementary Figure 58. ^{1}H NMR spectrum of **4a**

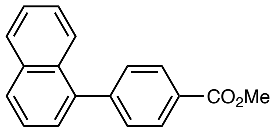

4a (^{13}C NMR, 126 MHz, $CDCl_3$)

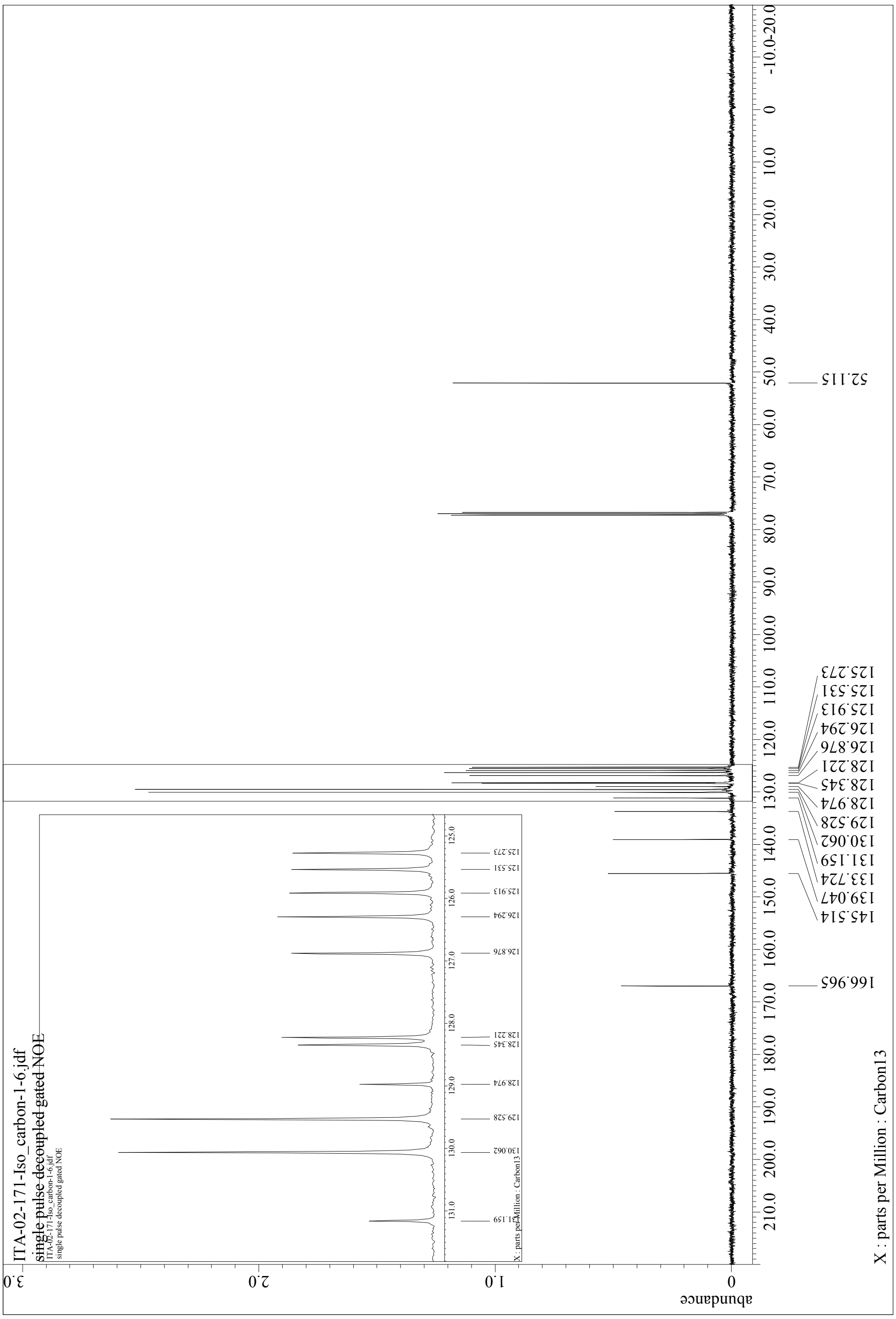

Supplementary Figure 59. ^{13}C NMR spectrum of **4a**

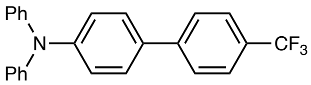

4b (^{1}H NMR, 500 MHz, $CDCl_3$)

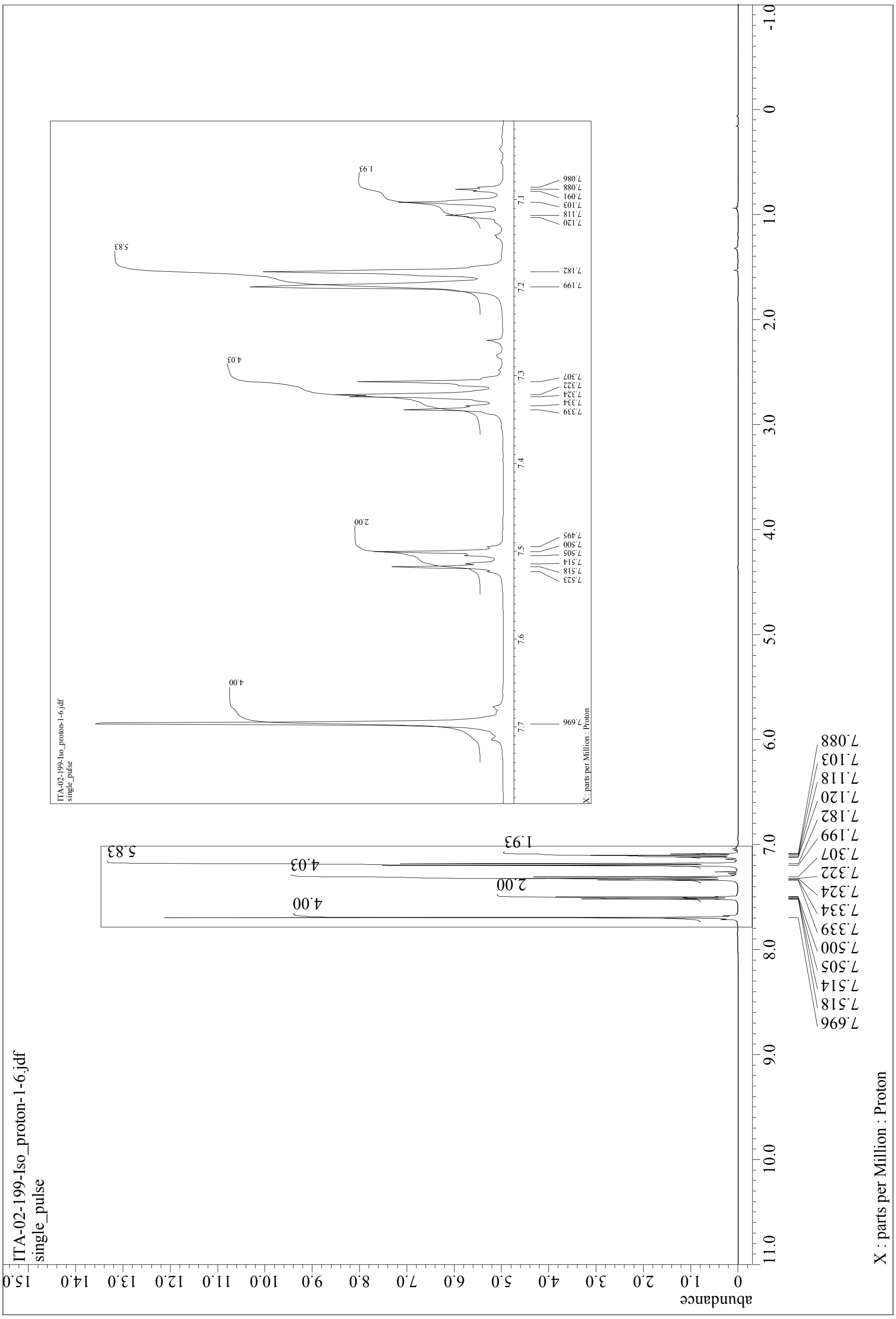

Supplementary Figure 60. ^{1}H NMR spectrum of **4b**

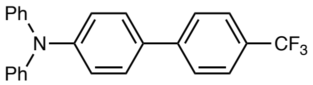

4b (^{13}C NMR, 126 MHz, $CDCl_3$)

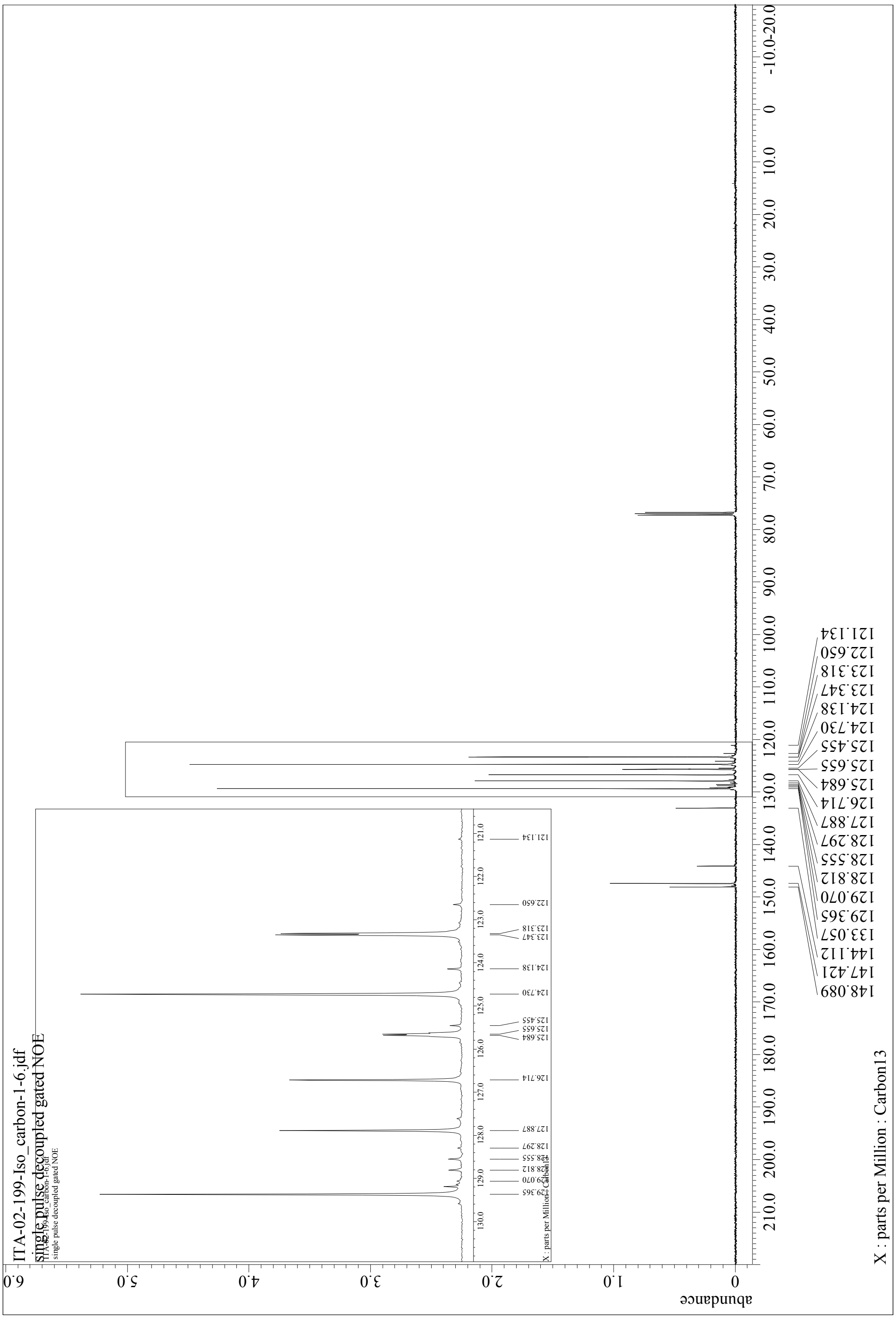

Supplementary Figure 61. ^{13}C NMR spectrum of **4b**

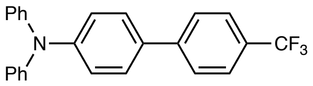 **4b** (^{19}F NMR, 470 MHz, $CDCl_3$)

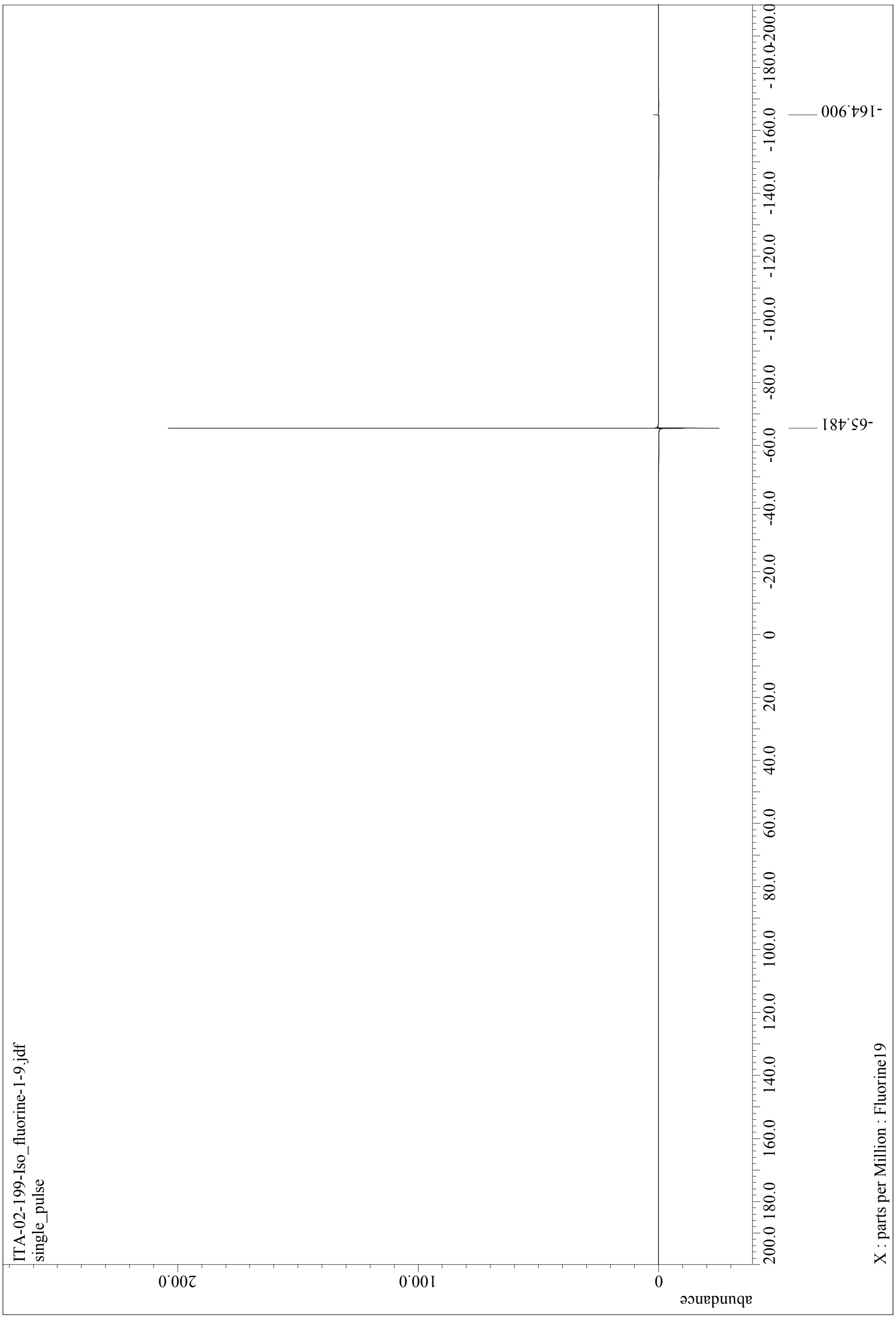

Supplementary Figure 62. ^{19}F NMR spectrum of **4b**

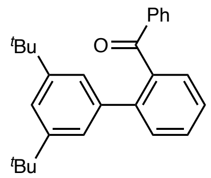

4c (^{1}H NMR, 500 MHz, $CDCl_3$)

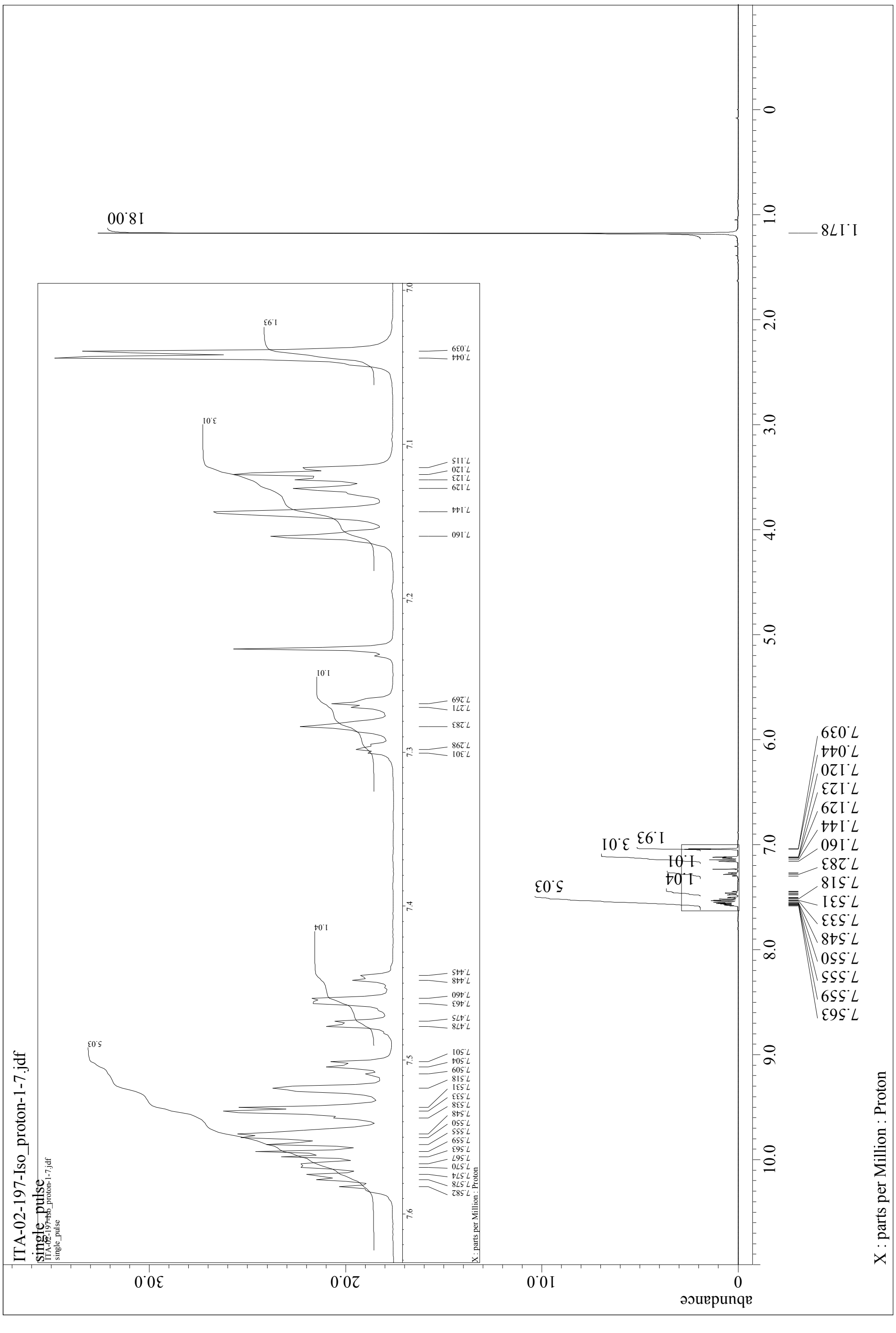

Supplementary Figure 63. ^{1}H NMR spectrum of **4c**

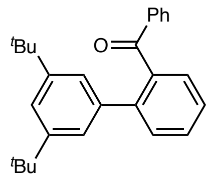

4c (^{13}C NMR, 126 MHz, $CDCl_3$)

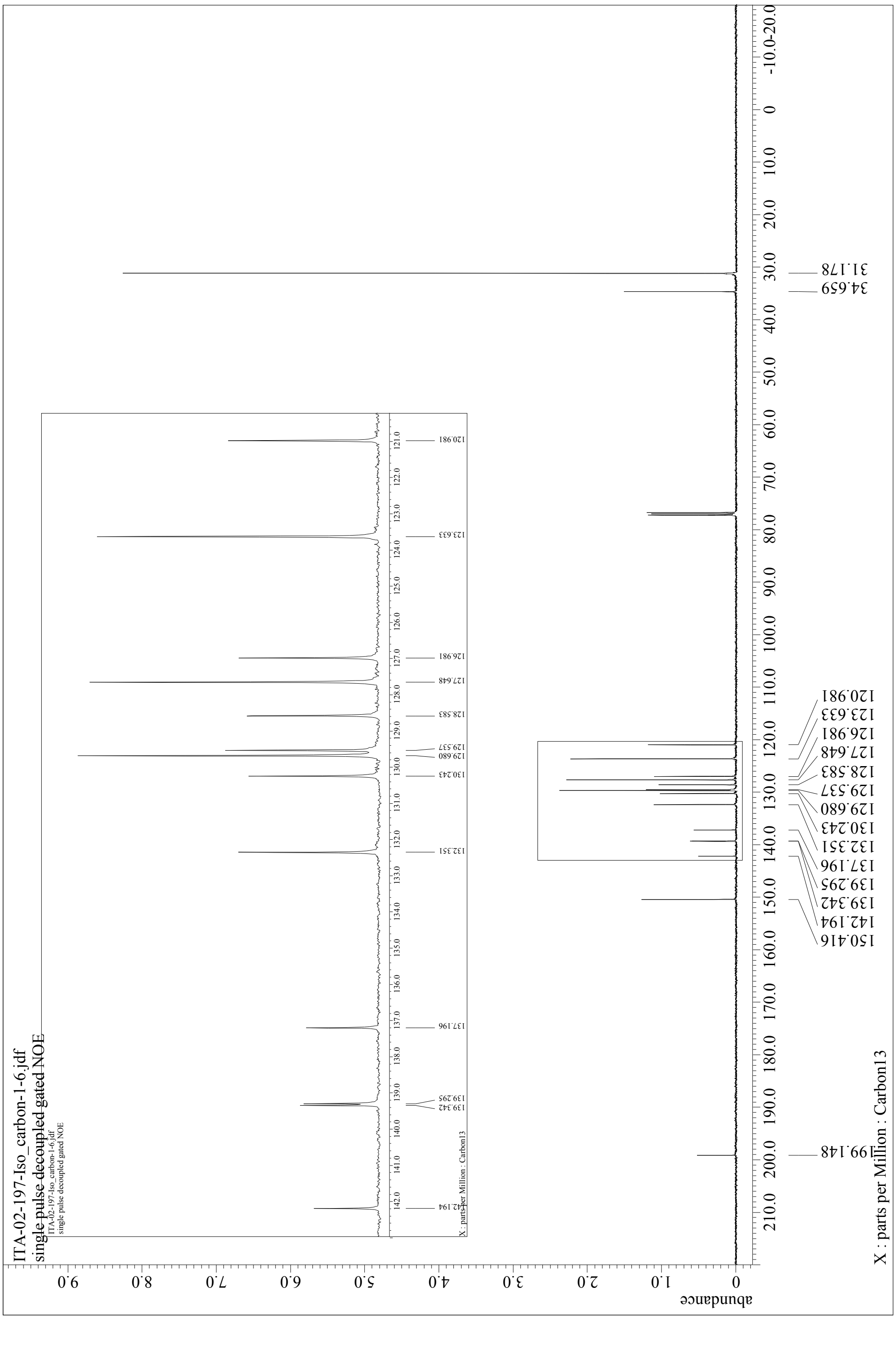

Supplementary Figure 64. ^{13}C NMR spectrum of **4c**

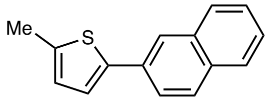

4d (^{1}H NMR, 500 MHz, $CDCl_3$)

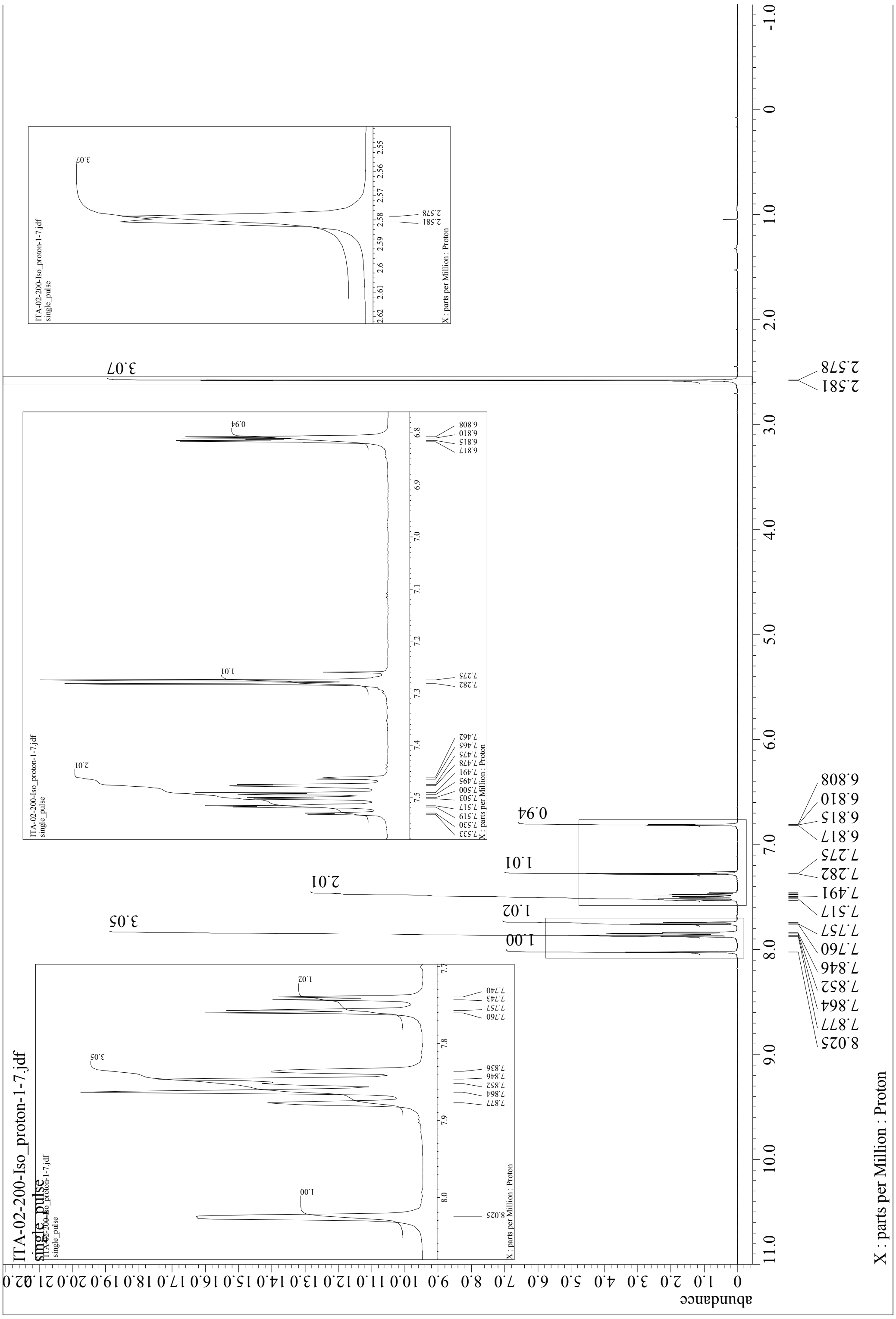

Supplementary Figure 65. ^{1}H NMR spectrum of **4d**

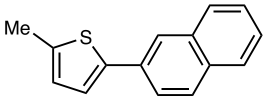

4d (^{13}C NMR, 126 MHz, $CDCl_3$)

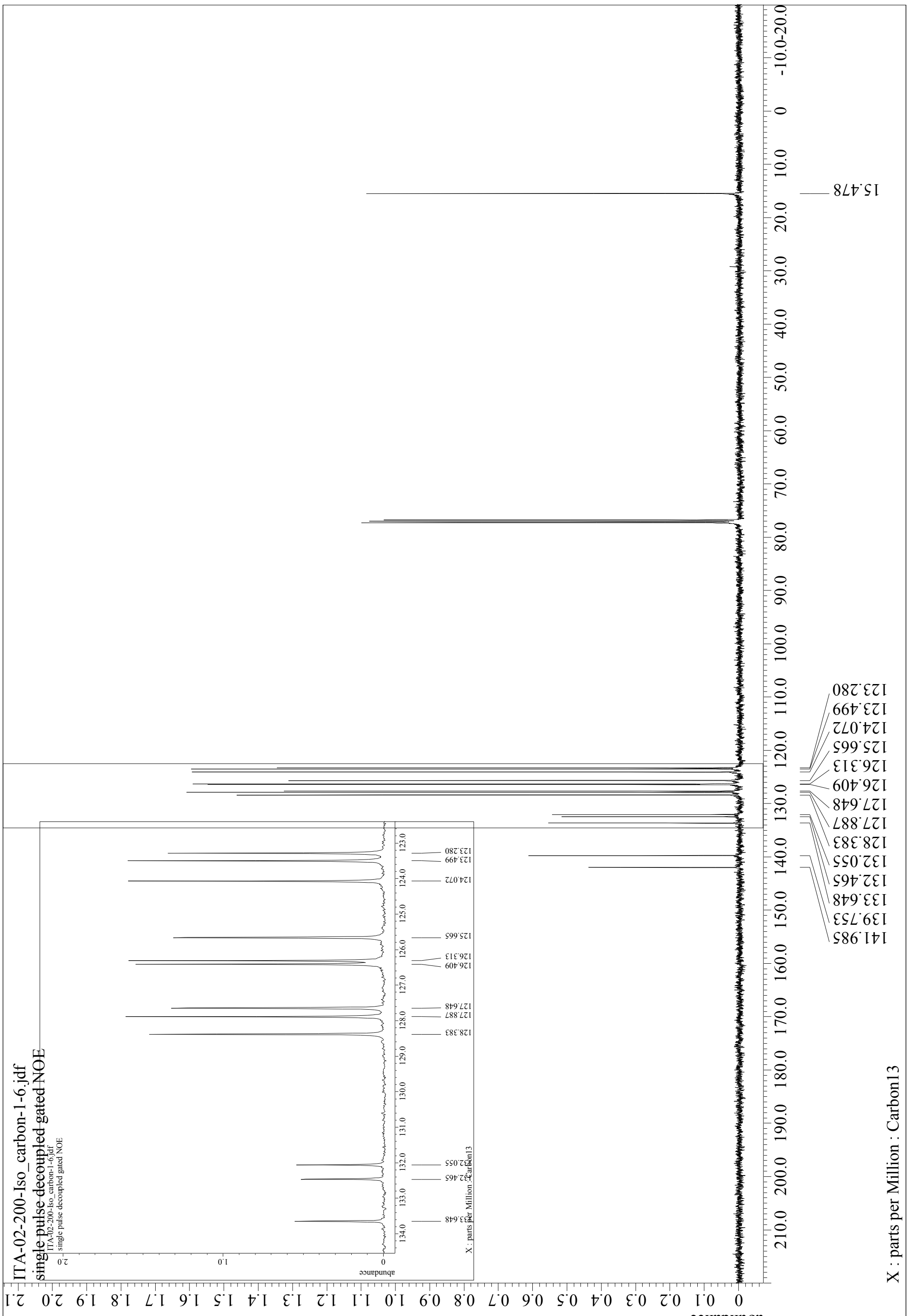

Supplementary Figure 66. ^{13}C NMR spectrum of **4d**

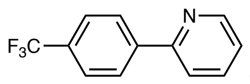

4e (^{1}H NMR, 500 MHz, $CDCl_3$)

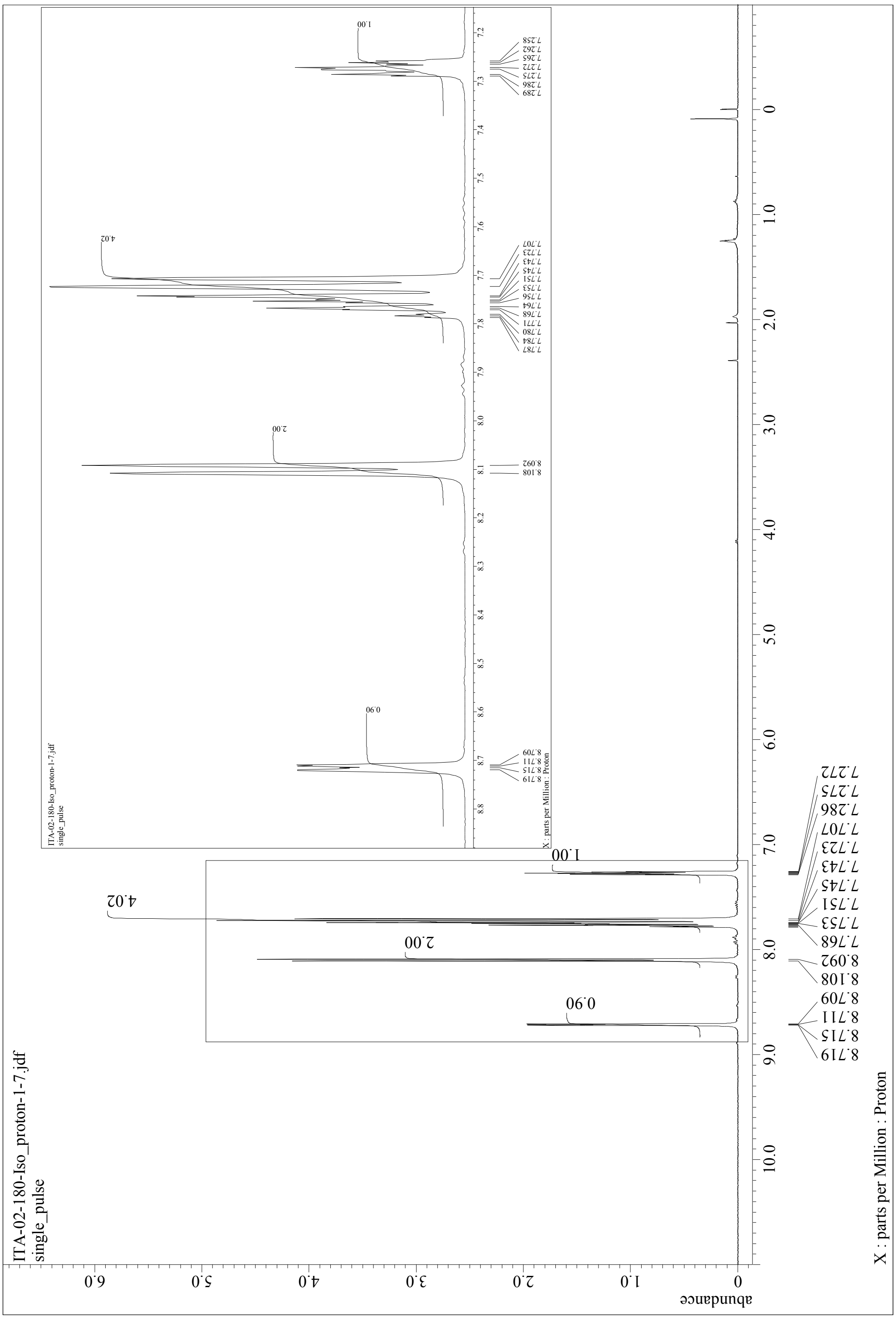

Supplementary Figure 67. ^{1}H NMR spectrum of **4e**

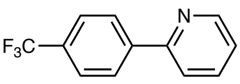

4e (^{13}C NMR, 126 MHz, $CDCl_3$)

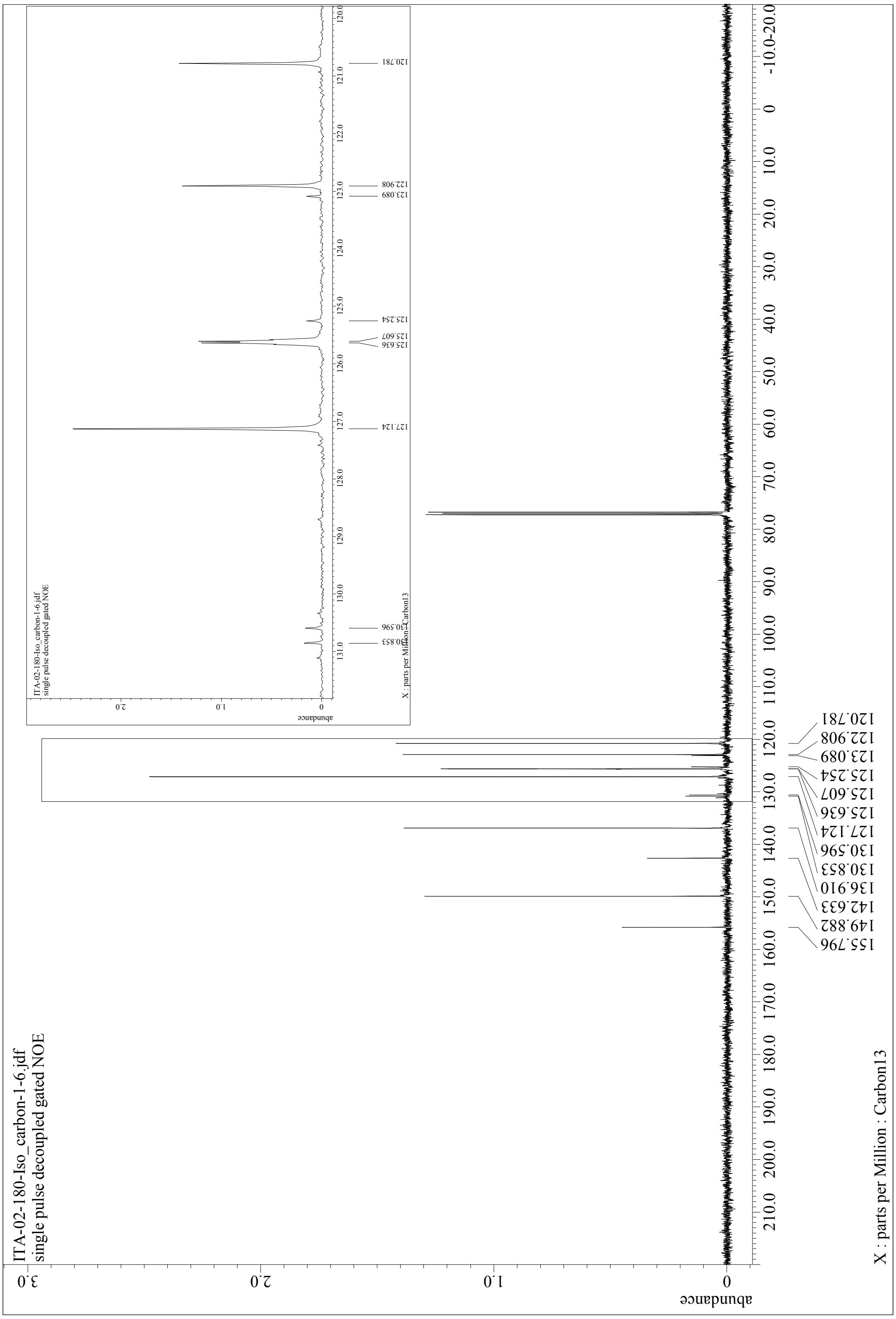

Supplementary Figure 68. ^{13}C NMR spectrum of **4e**

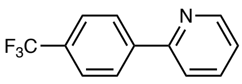

4e (^{19}F NMR, 470 MHz, $CDCl_3$)

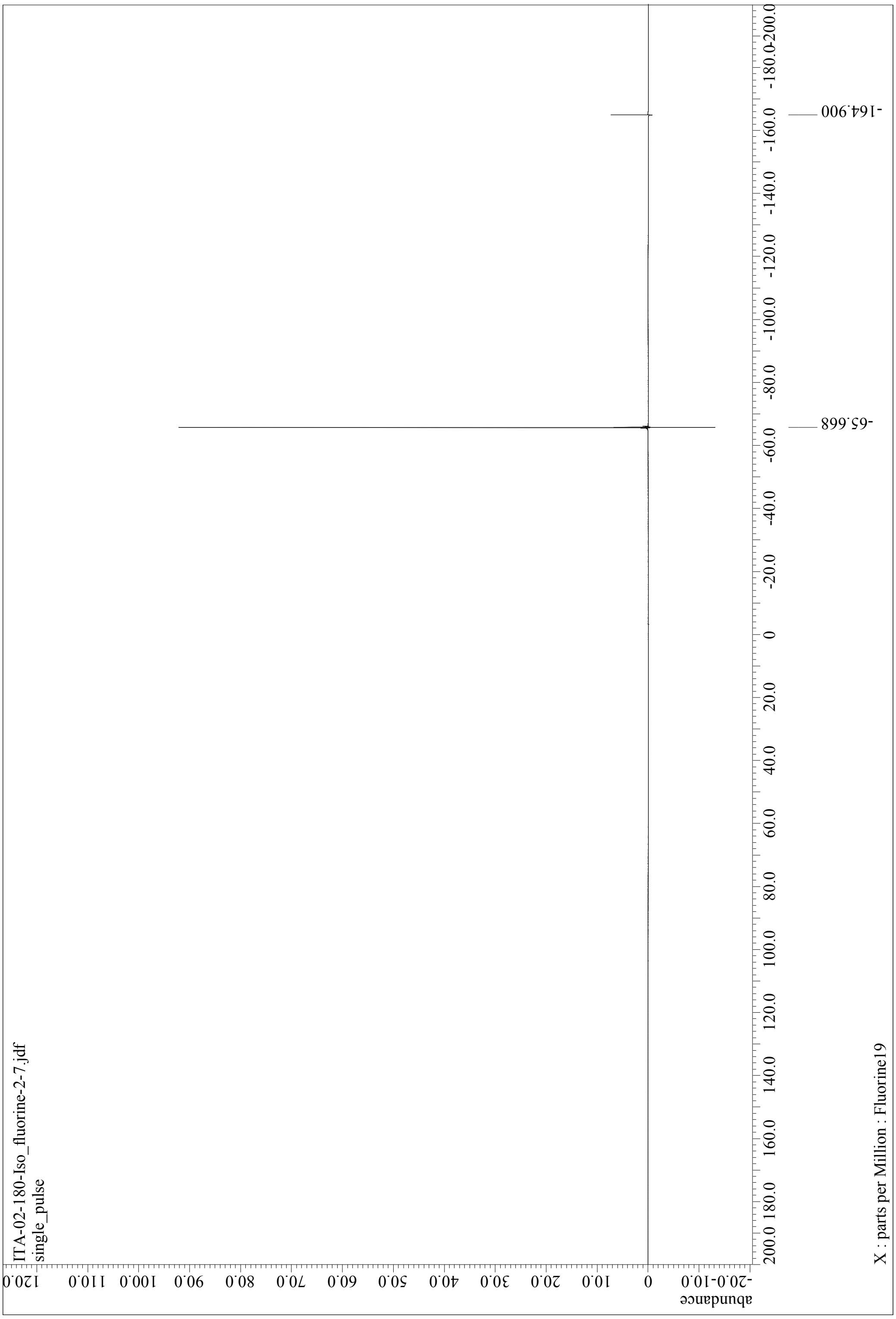

Supplementary Figure 69. ^{19}F NMR spectrum of **4e**

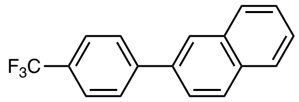

4f (^{1}H NMR, 500 MHz, $CDCl_3$)

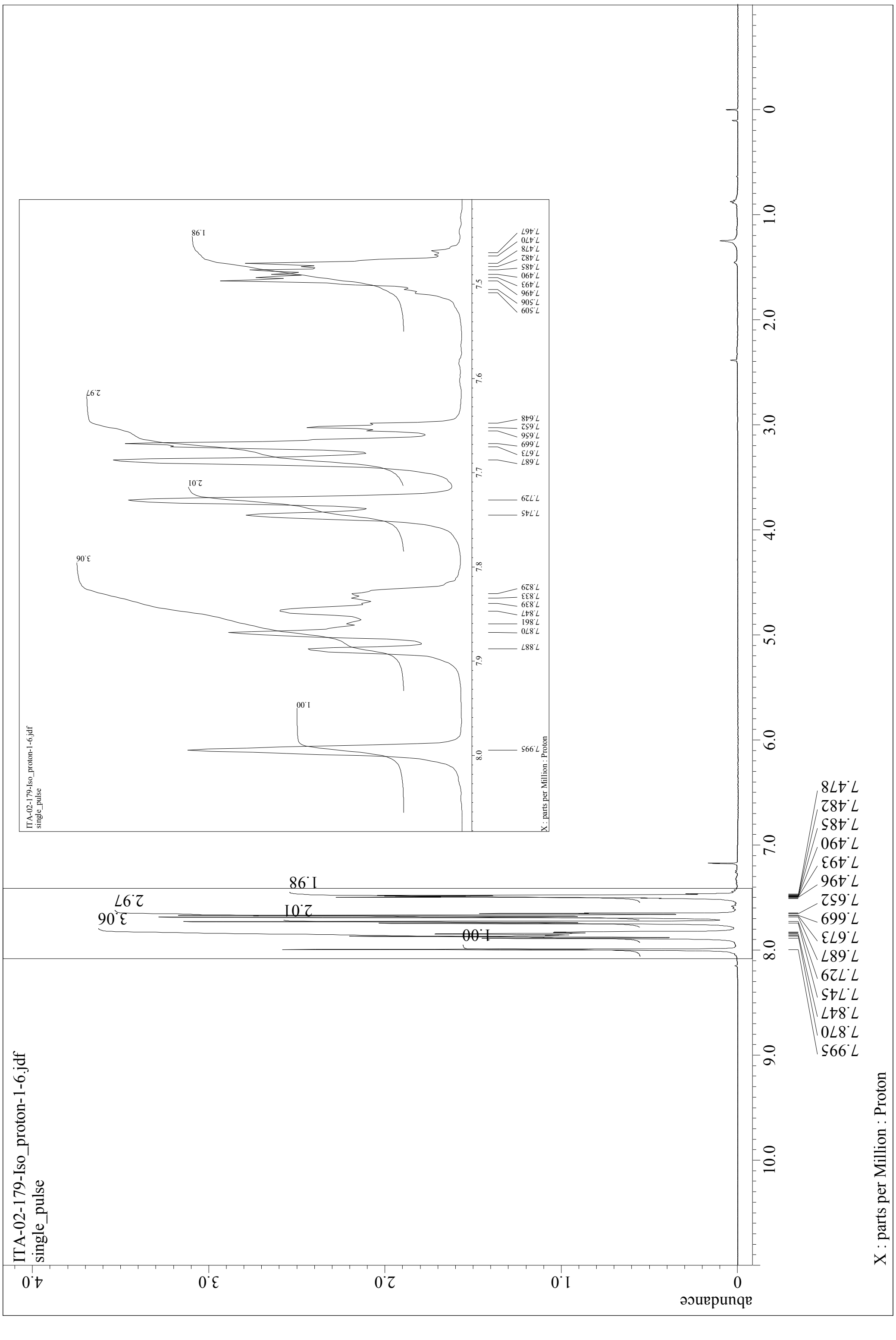

Supplementary Figure 70. ^{1}H NMR spectrum of **4f**

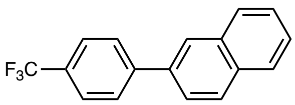

4f (^{13}C NMR, 126 MHz, $CDCl_3$)

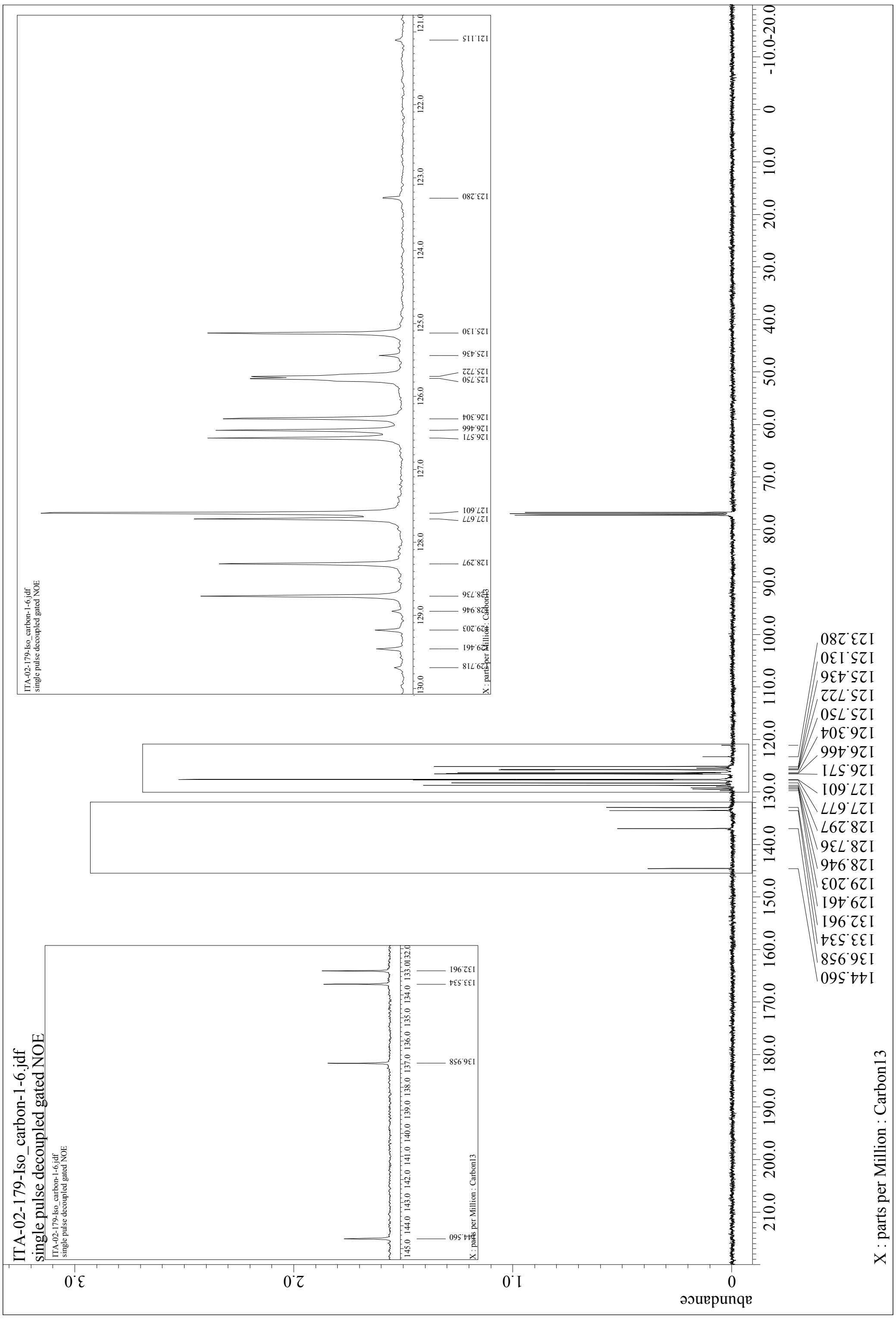

Supplementary Figure 71. ^{13}C NMR spectrum of **4f**

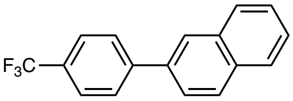

4f (^{19}F NMR, 470 MHz, $CDCl_3$)

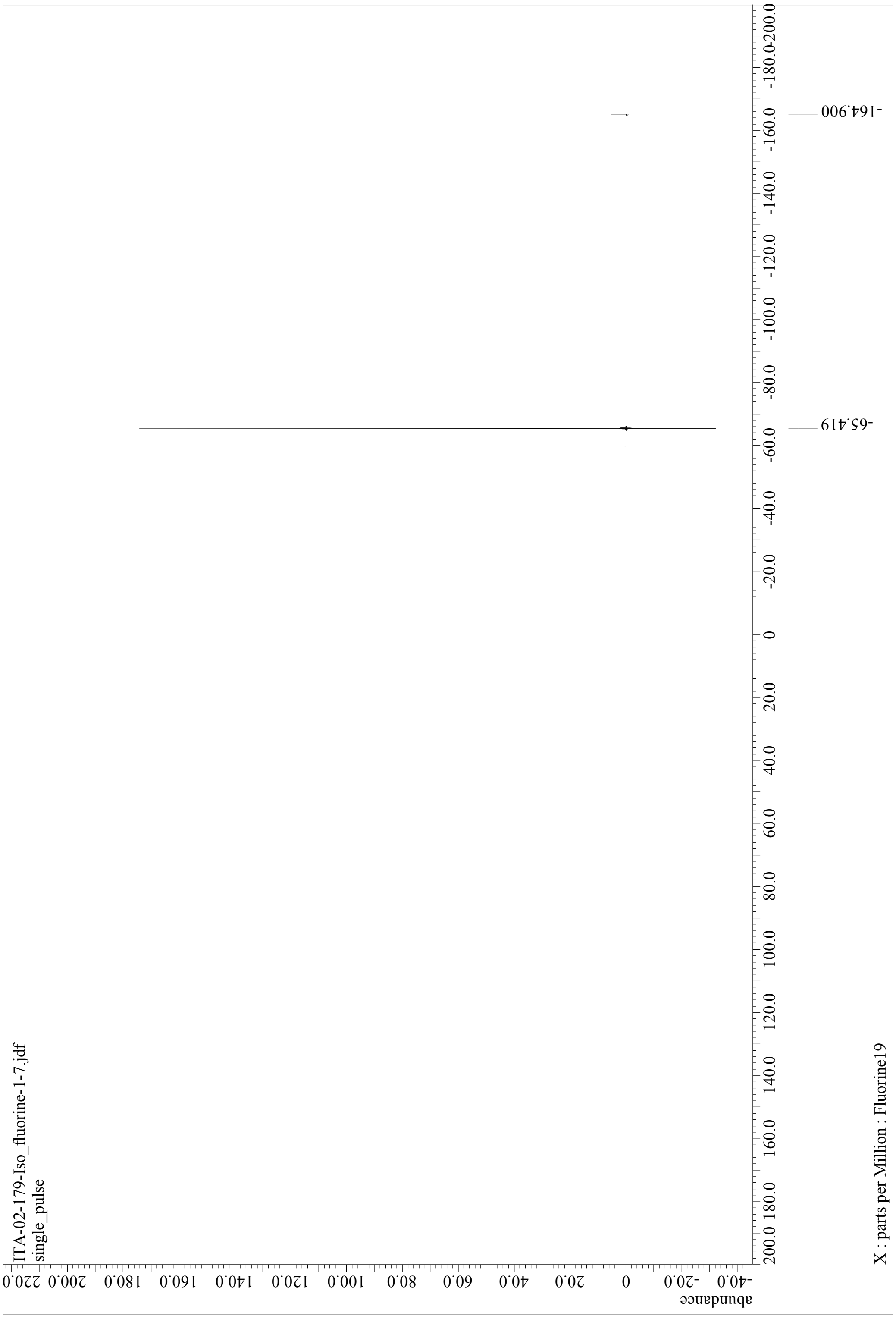

Supplementary Figure 72. ^{19}F NMR spectrum of **4f**

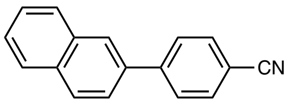

5a (^{1}H NMR, 500 MHz, $CDCl_3$)

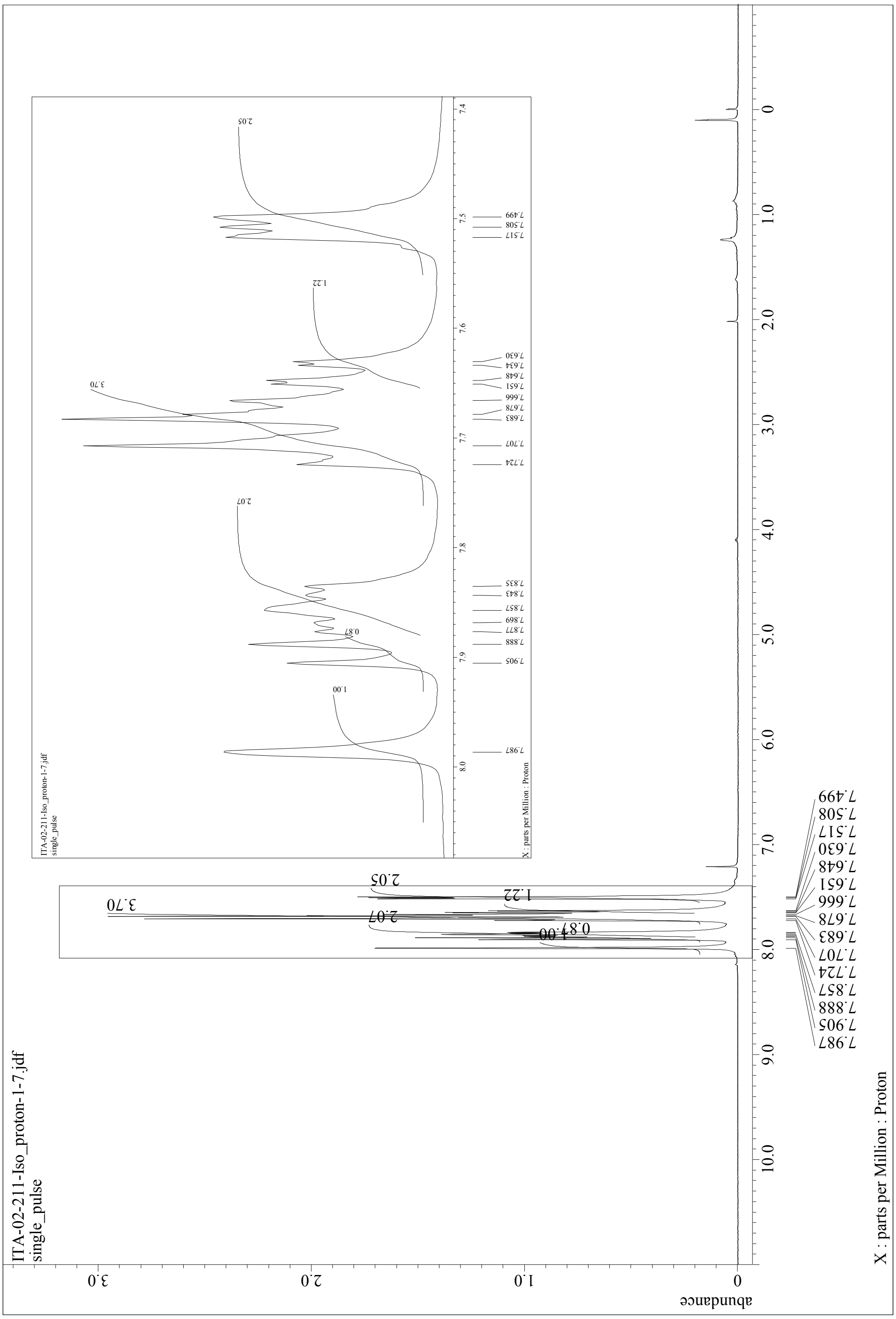

Supplementary Figure 73. ^{1}H NMR spectrum of **5a**

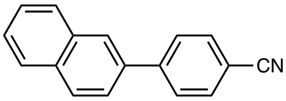

5a (^{13}C NMR, 126 MHz, $CDCl_3$)

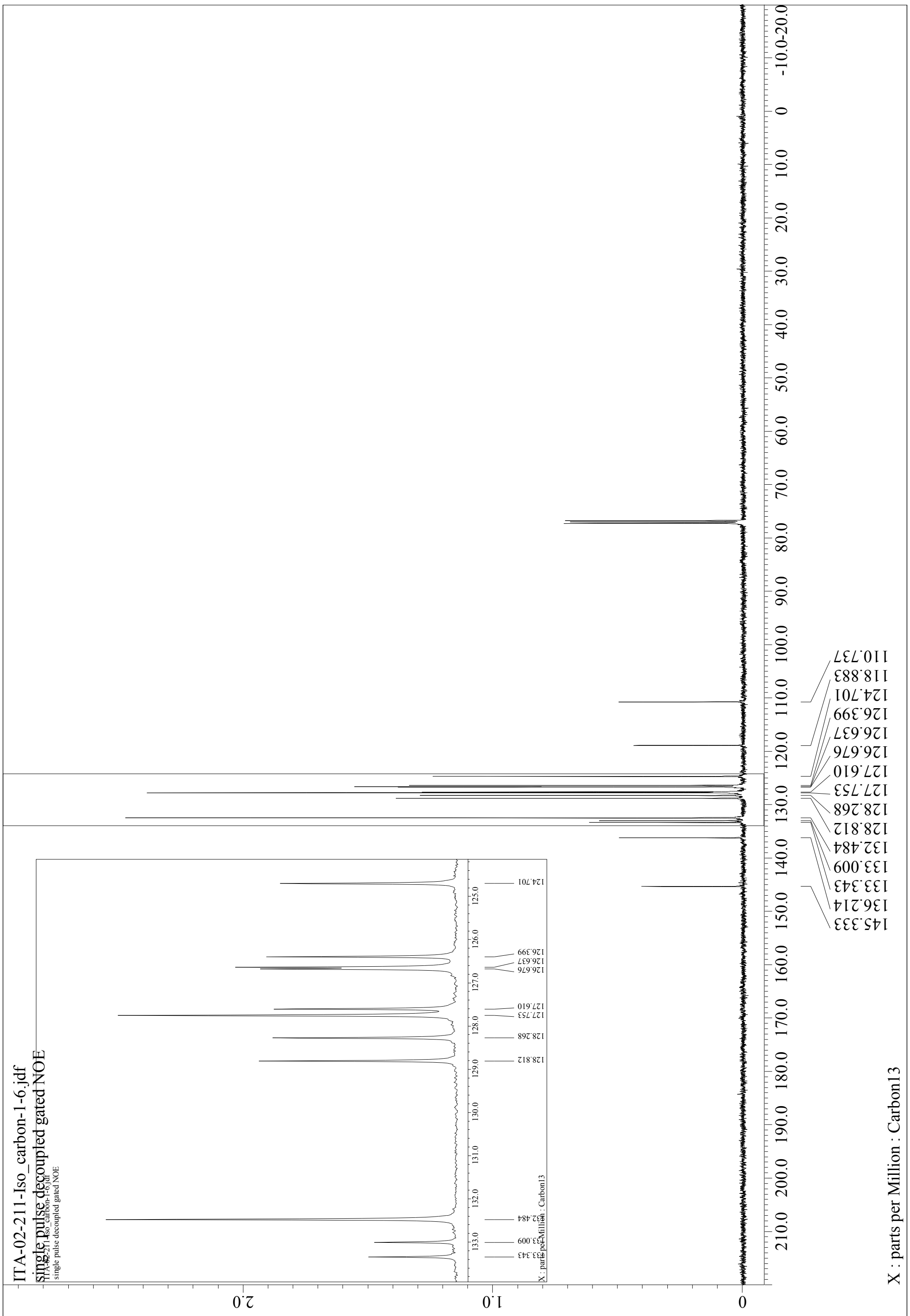

Supplementary Figure 74. ^{13}C NMR spectrum of **5a**

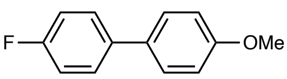

5b (^{1}H NMR, 500 MHz, $CDCl_3$)

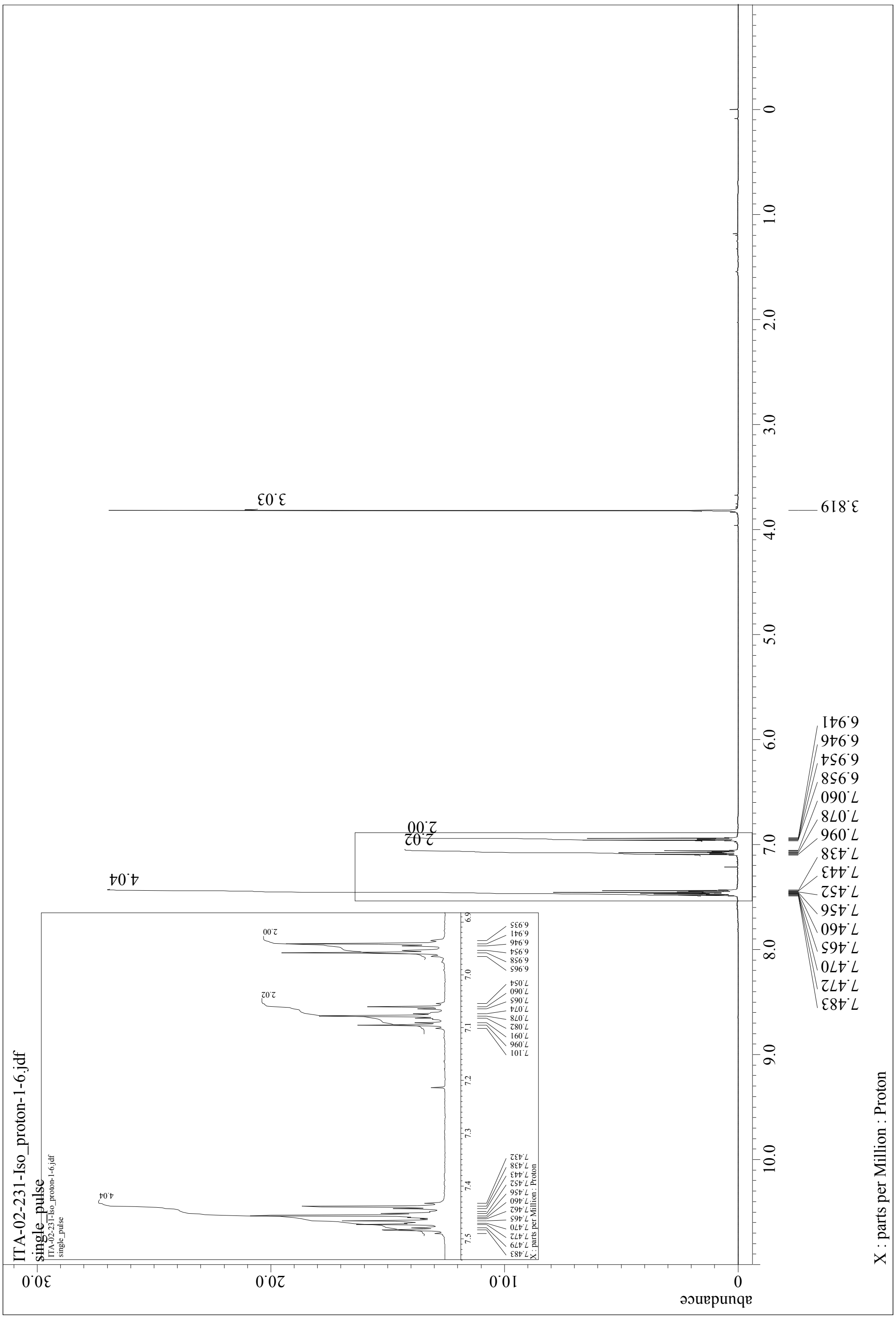

Supplementary Figure 75. ^{1}H NMR spectrum of **5b**

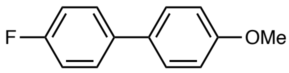

5b (^{13}C NMR, 126 MHz, $CDCl_3$)

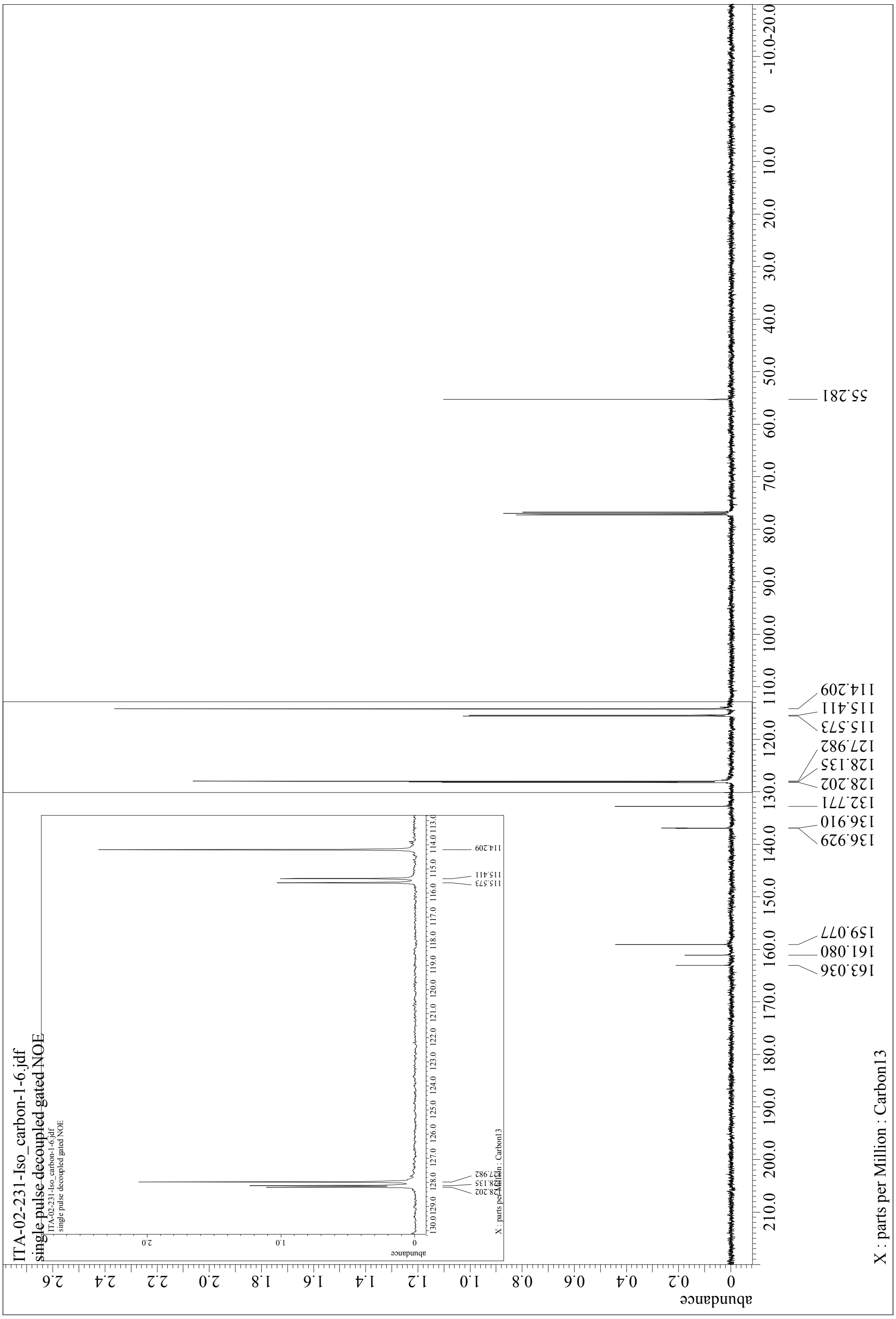

Supplementary Figure 76. ^{13}C NMR spectrum of **5b**

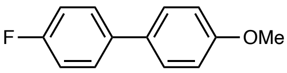

5b (^{19}F NMR, 470 MHz, $CDCl_3$)

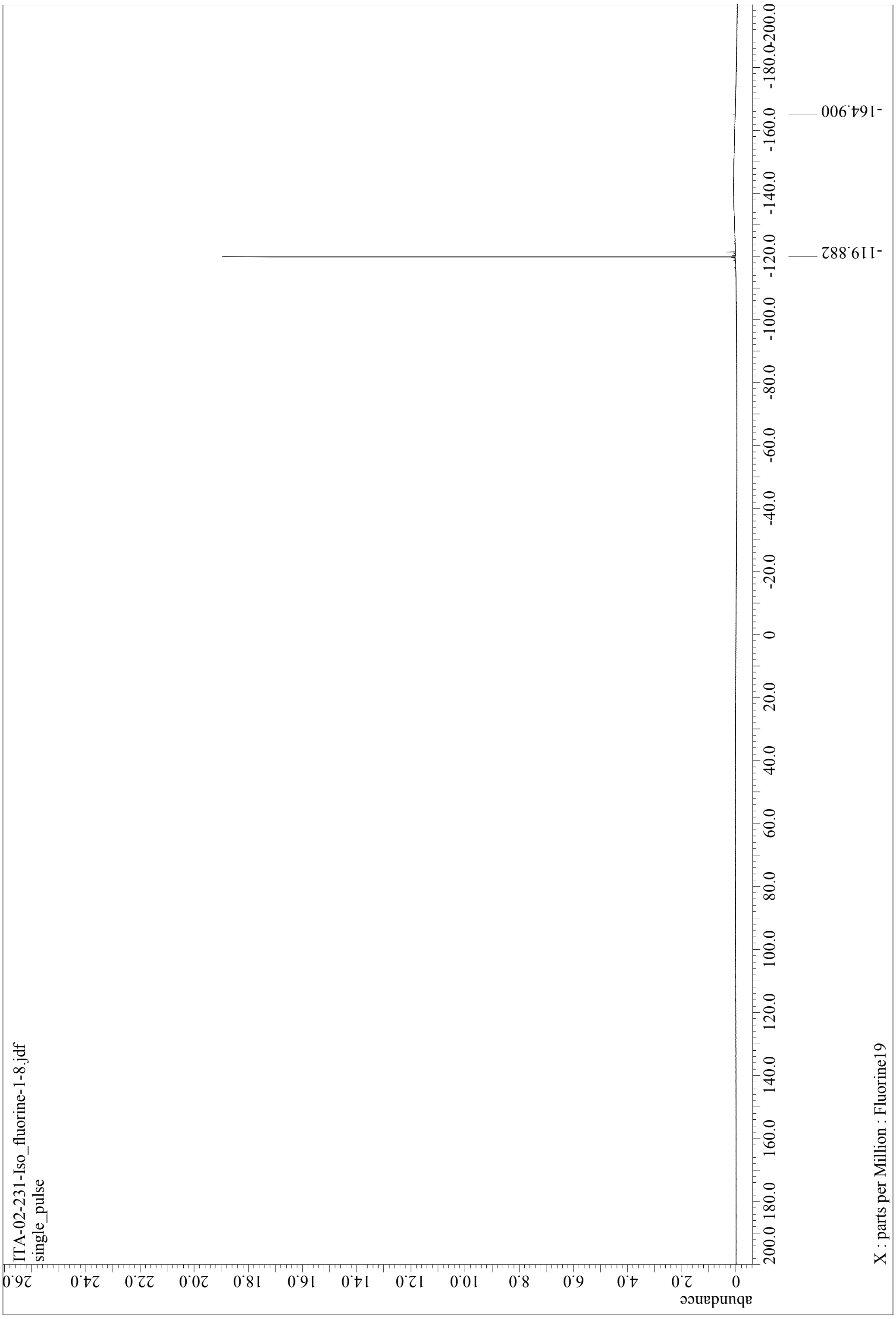

Supplementary Figure 77. ^{19}F NMR spectrum of **5b**

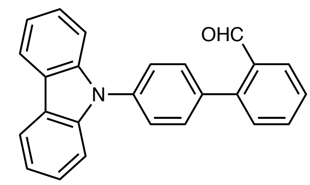

5c (^{1}H NMR, 500 MHz, $CDCl_3$)

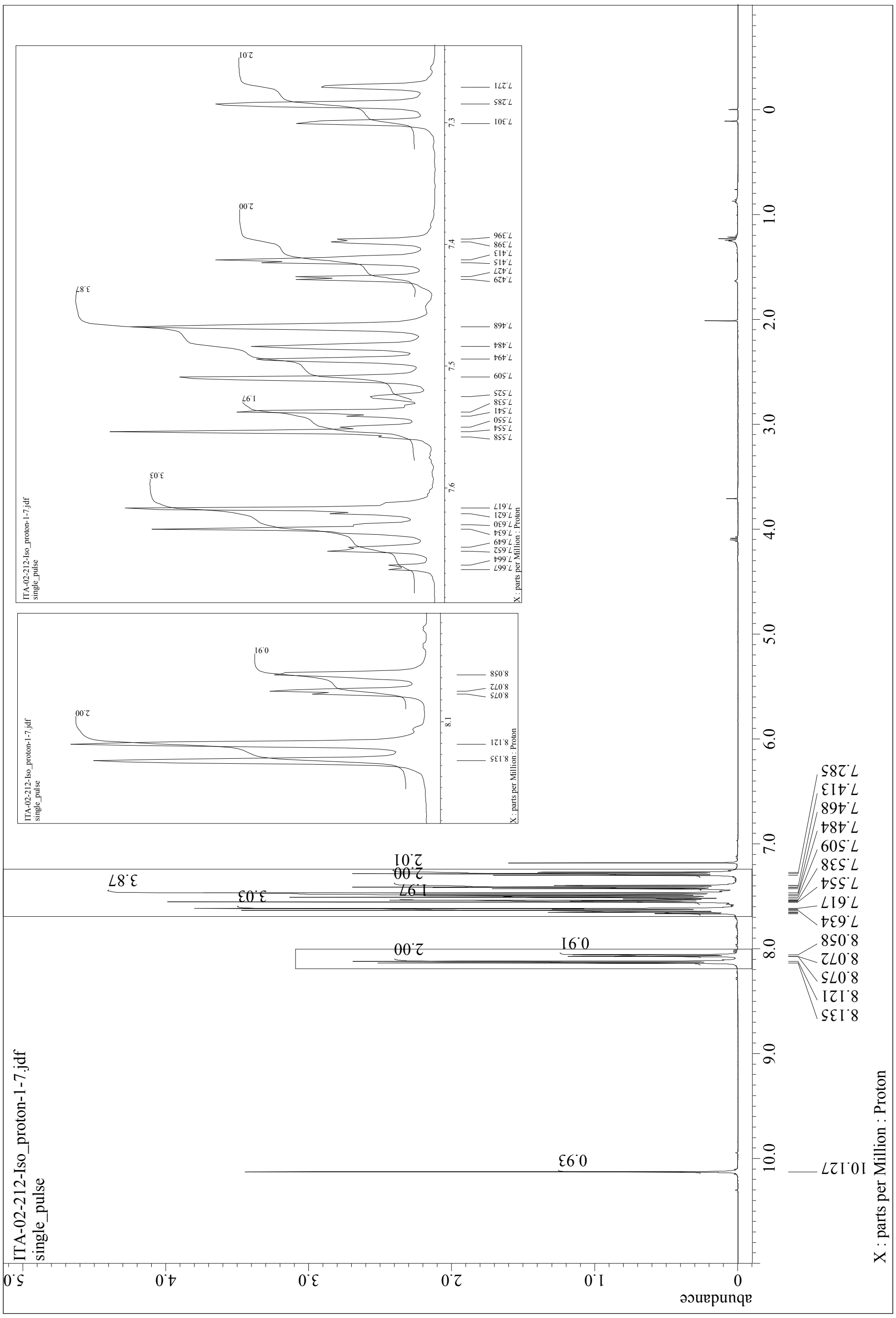

Supplementary Figure 78. ^{1}H NMR spectrum of **5c**

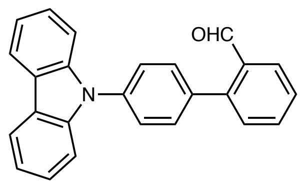

5c (^{13}C NMR, 126 MHz, $CDCl_3$)

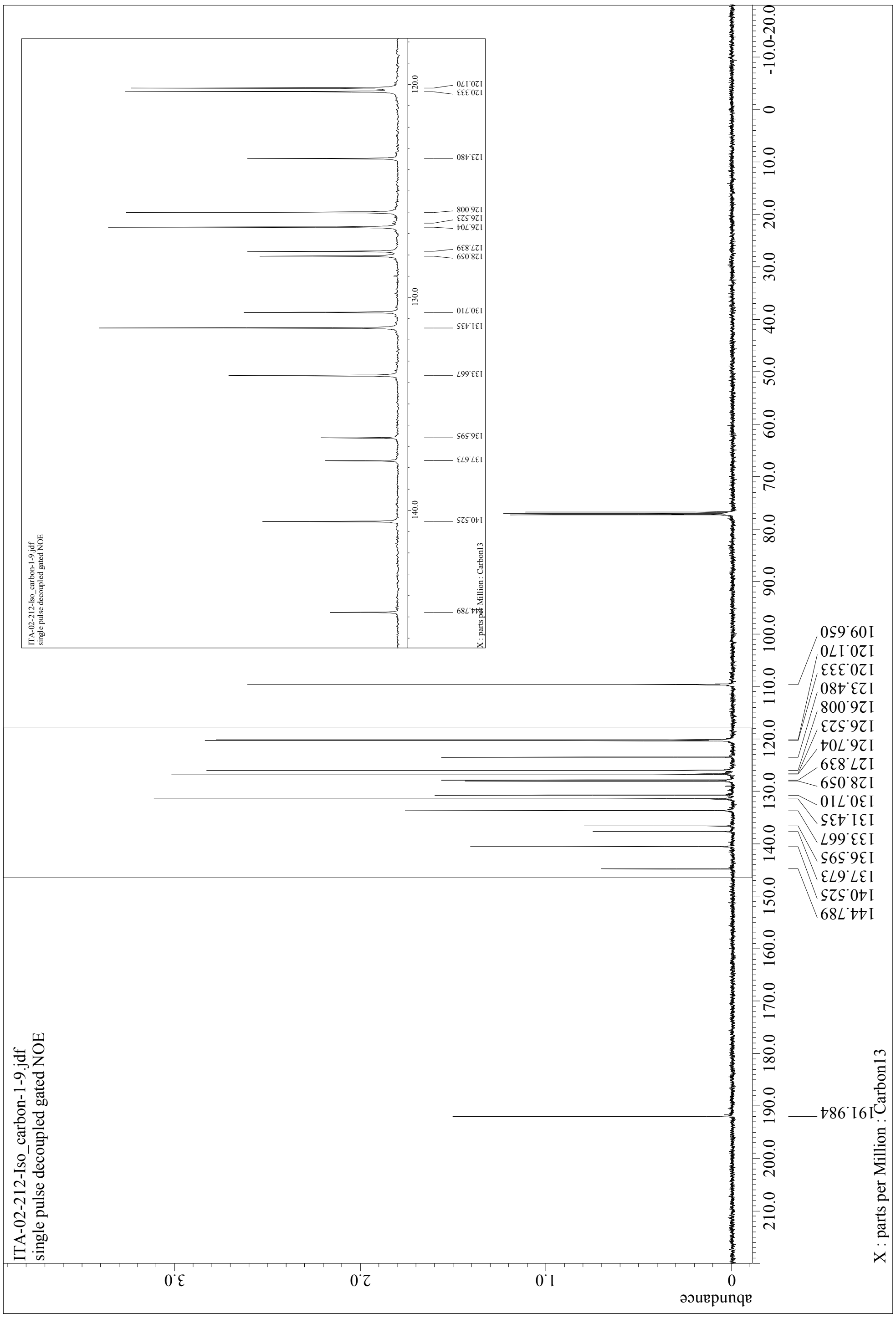

Supplementary Figure 79. ^{13}C NMR spectrum of **5c**

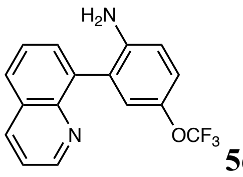

5d (^{1}H NMR, 500 MHz, $CDCl_3$)

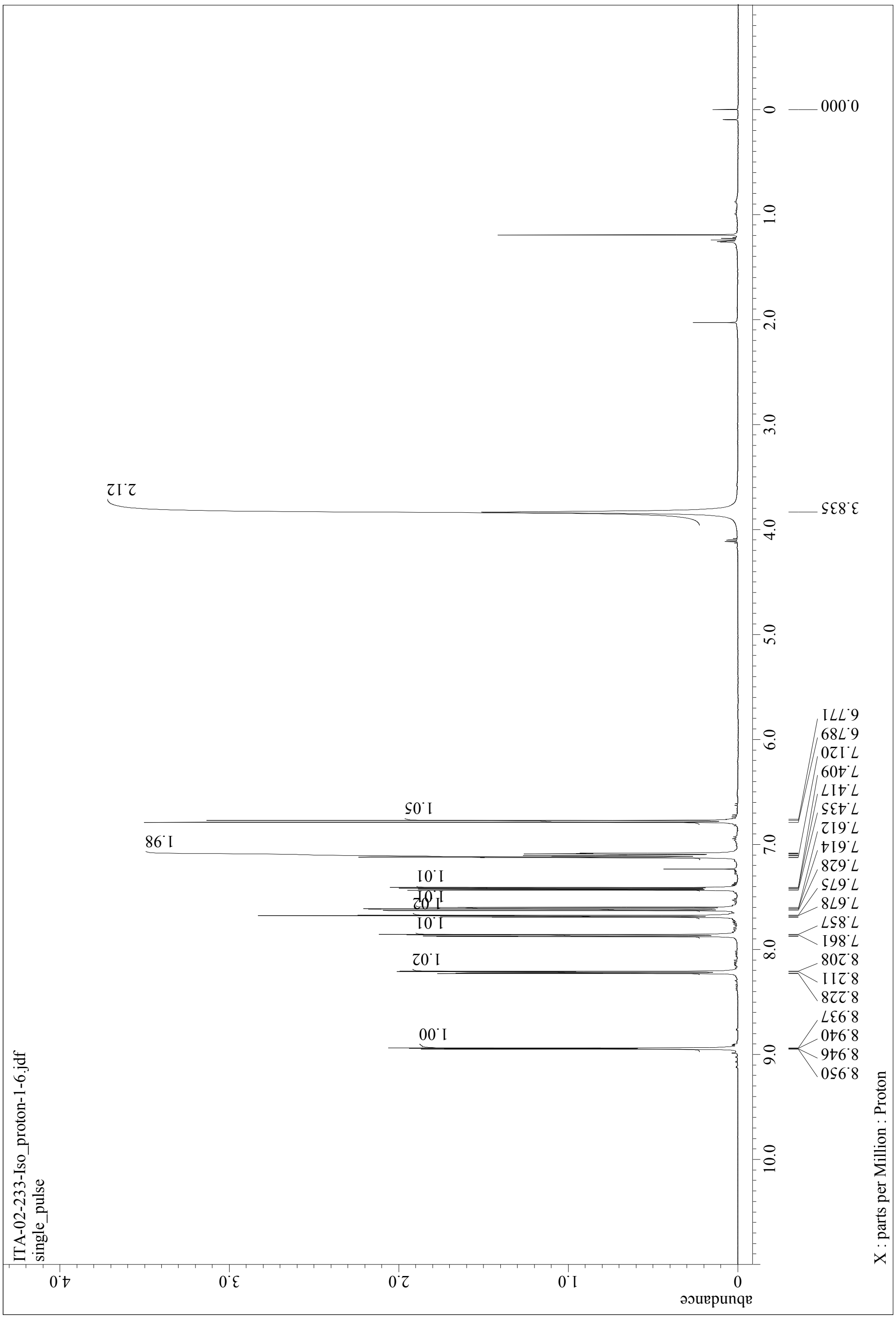

Supplementary Figure 80. ^{1}H NMR spectrum of **5d**

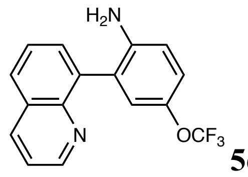

5d (^{13}C NMR, 126 MHz, $CDCl_3$)

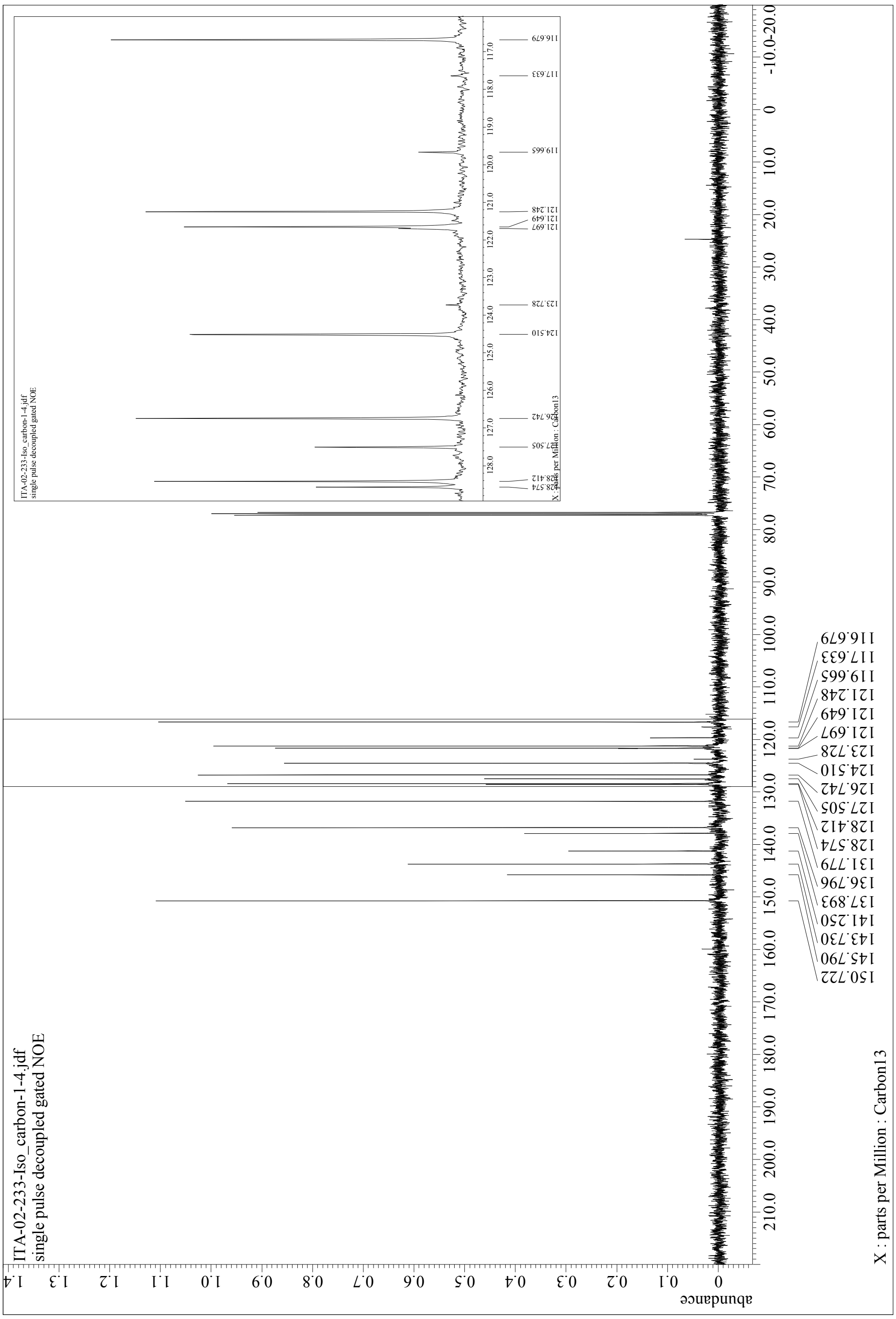

Supplementary Figure 81. ^{13}C NMR spectrum of **5d**

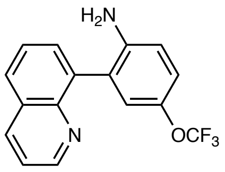

5d (^{19}F NMR, 470 MHz, $CDCl_3$)

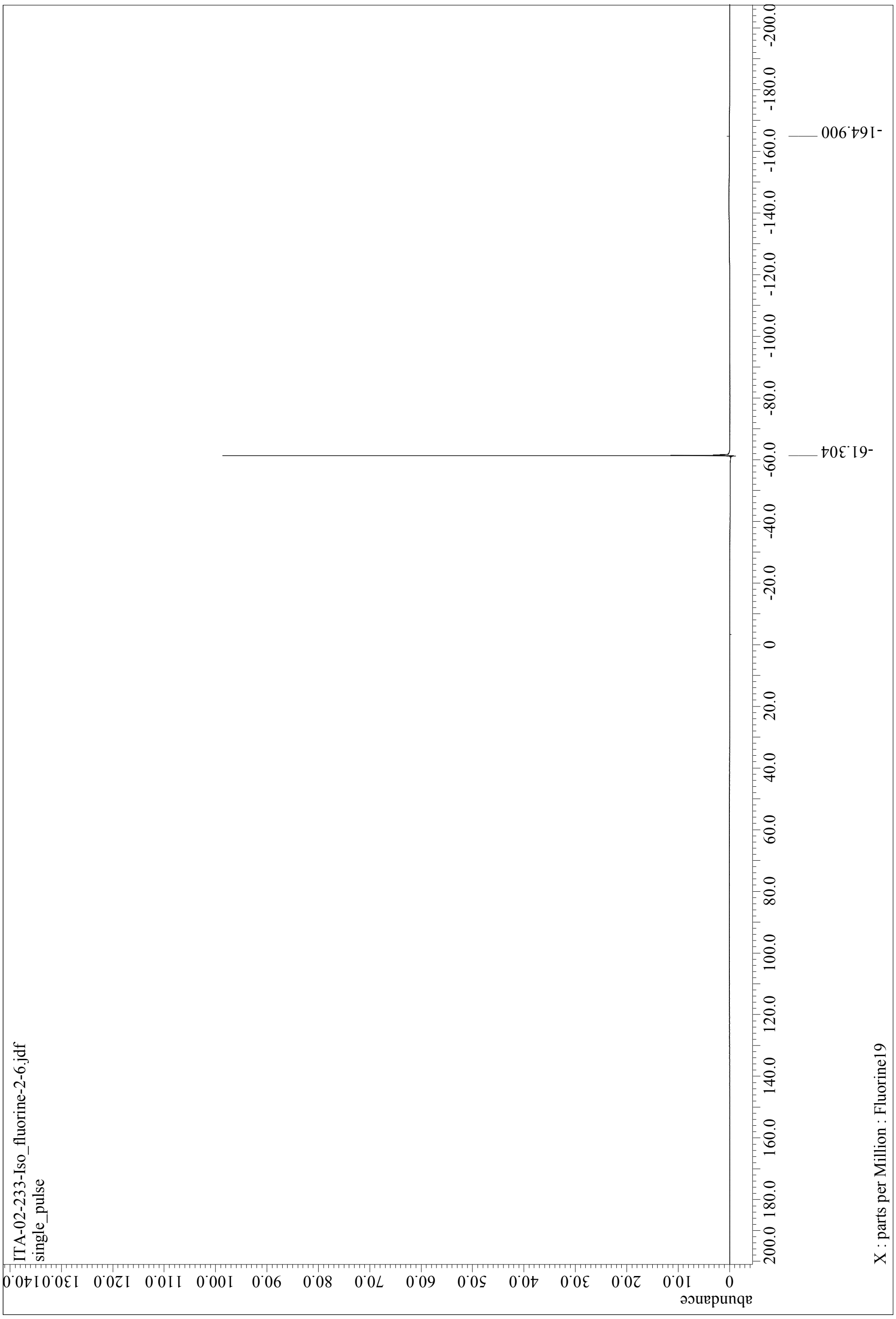

Supplementary Figure 82. ^{19}F NMR spectrum of **5d**

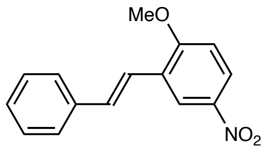

5e (^{1}H NMR, 500 MHz, $CDCl_3$)

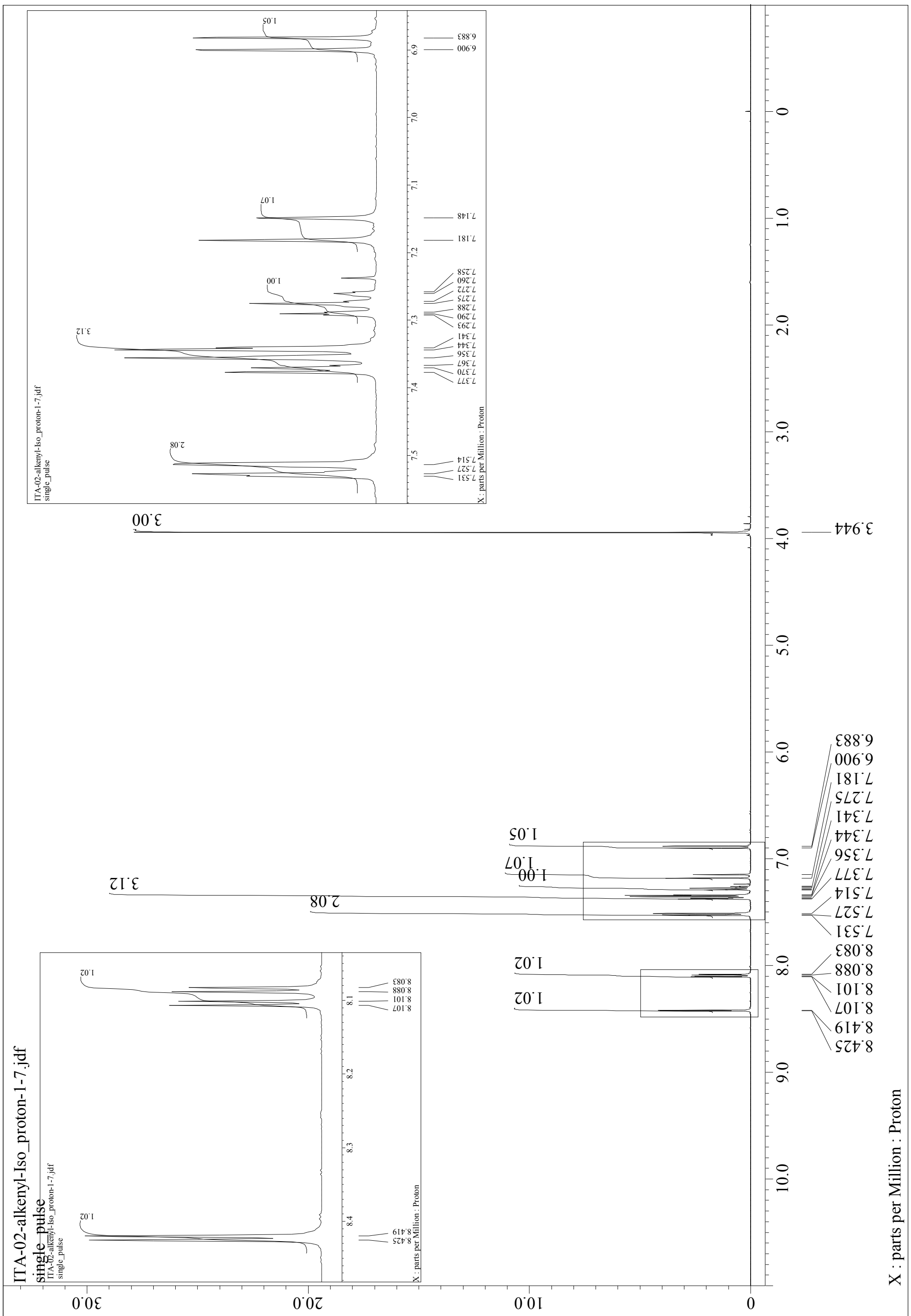

Supplementary Figure 83. ^{1}H NMR spectrum of **5e**

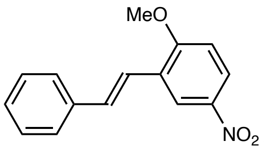

5e (^{13}C NMR, 126 MHz, $CDCl_3$)

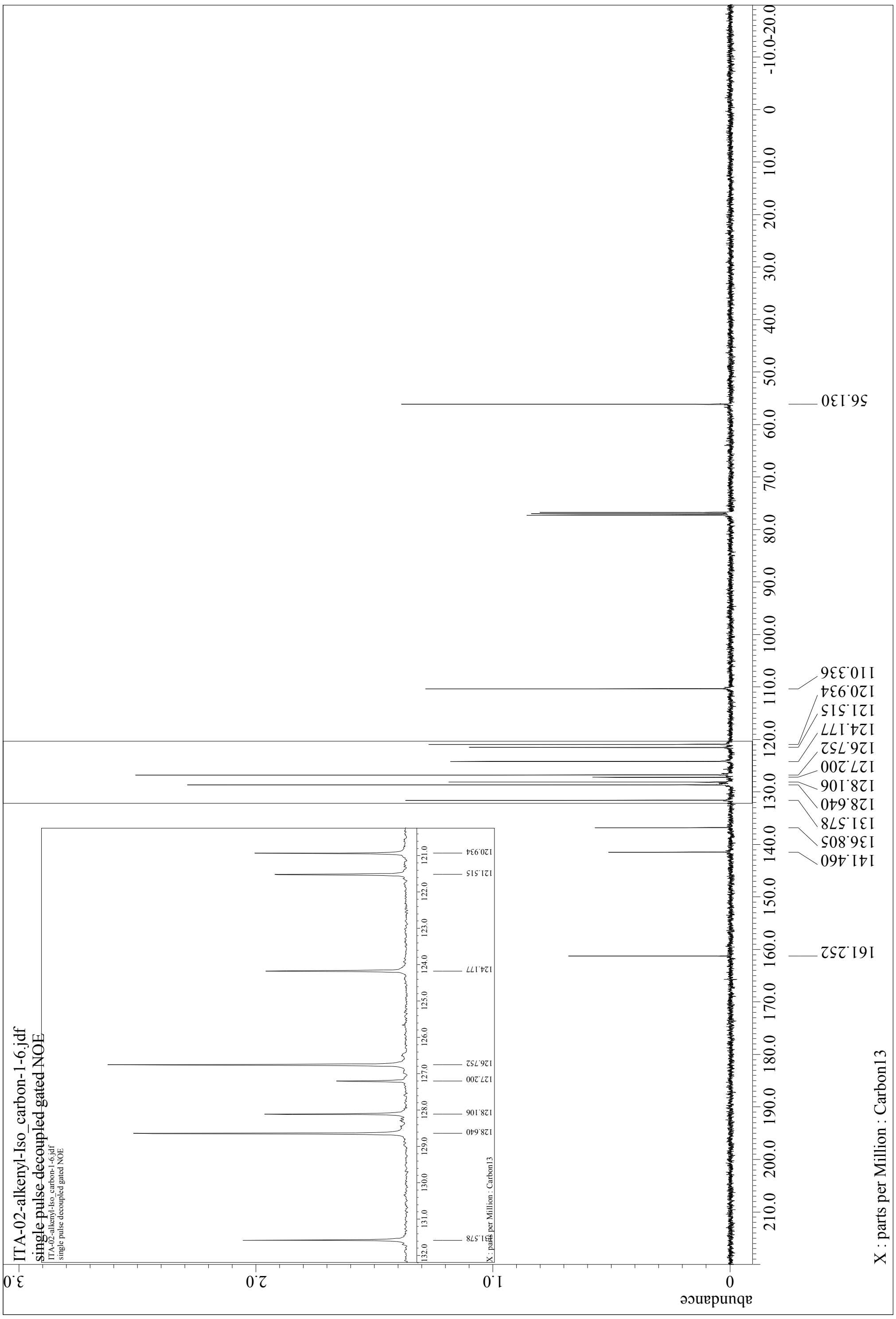

Supplementary Figure 84. ^{13}C NMR spectrum of **5e**

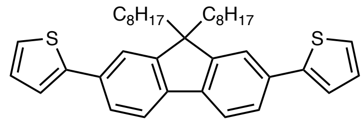

5f (^{1}H NMR, 500 MHz, $CDCl_3$)

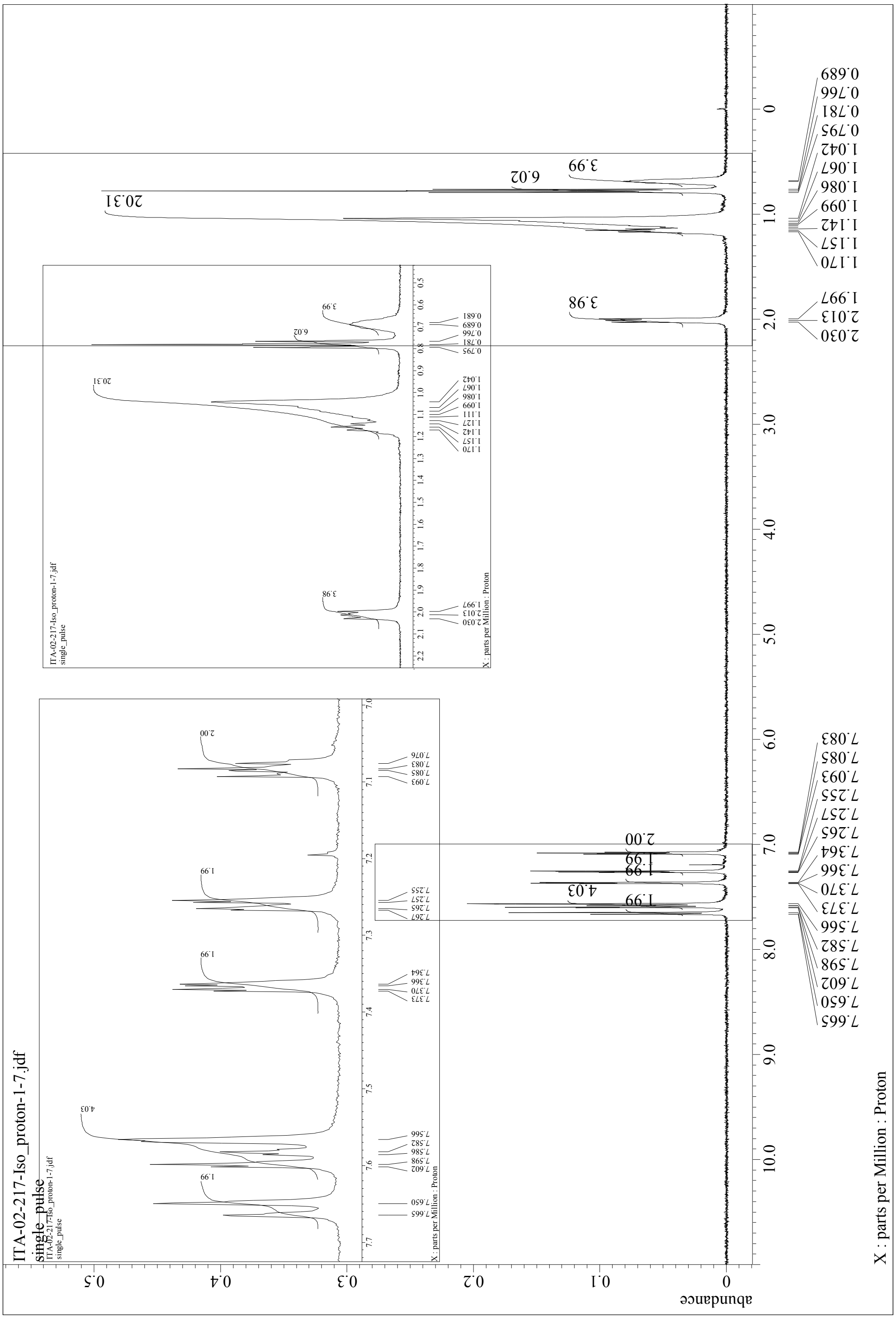

Supplementary Figure 85. ^{1}H NMR spectrum of **5f**

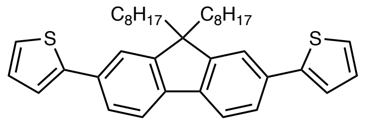

5f (^{13}C NMR, 100 MHz, $CDCl_3$)

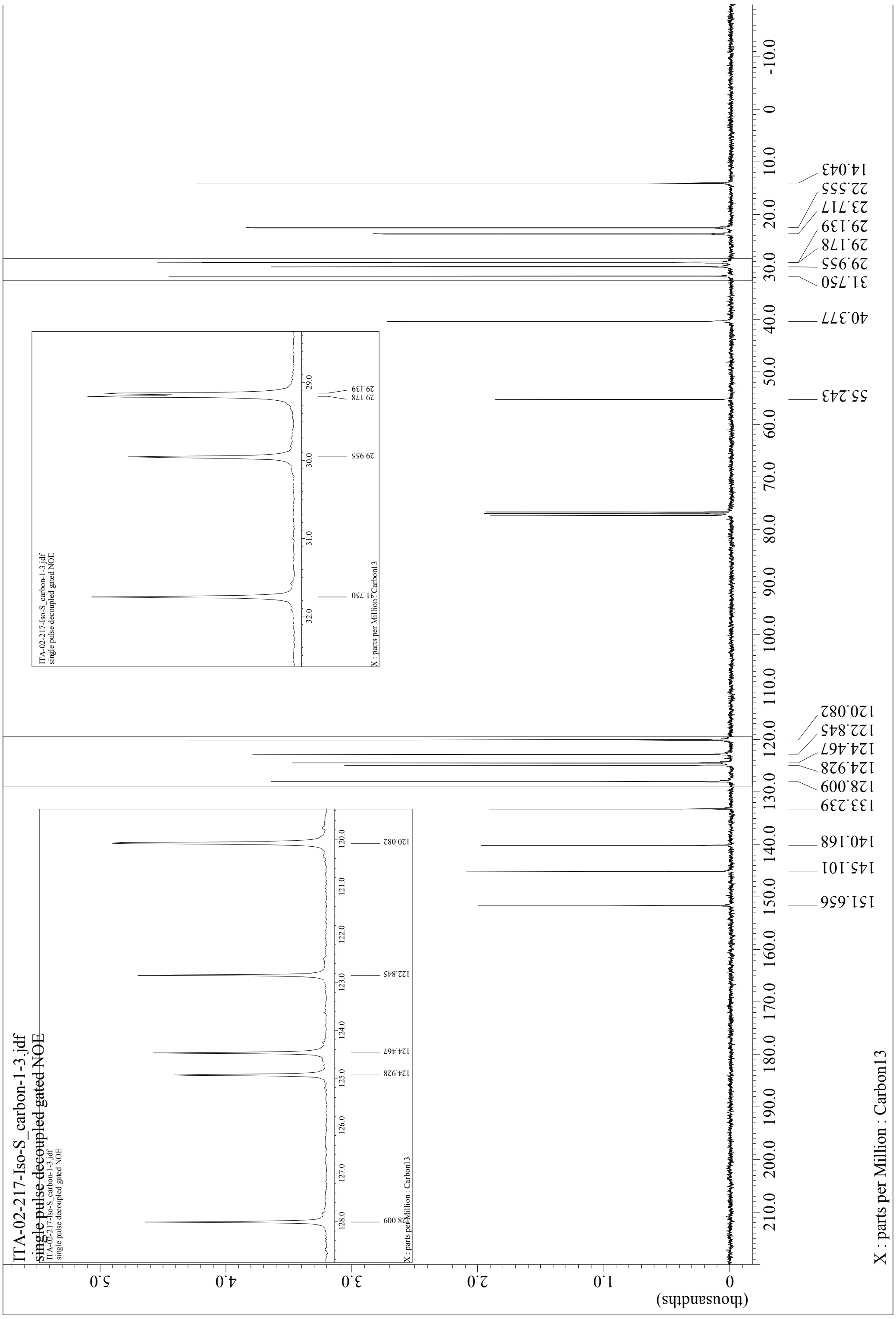

Supplementary Figure 86. ^{13}C NMR spectrum of **5f**

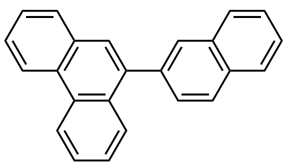

6a (^{1}H NMR, 500 MHz, $CDCl_3$)

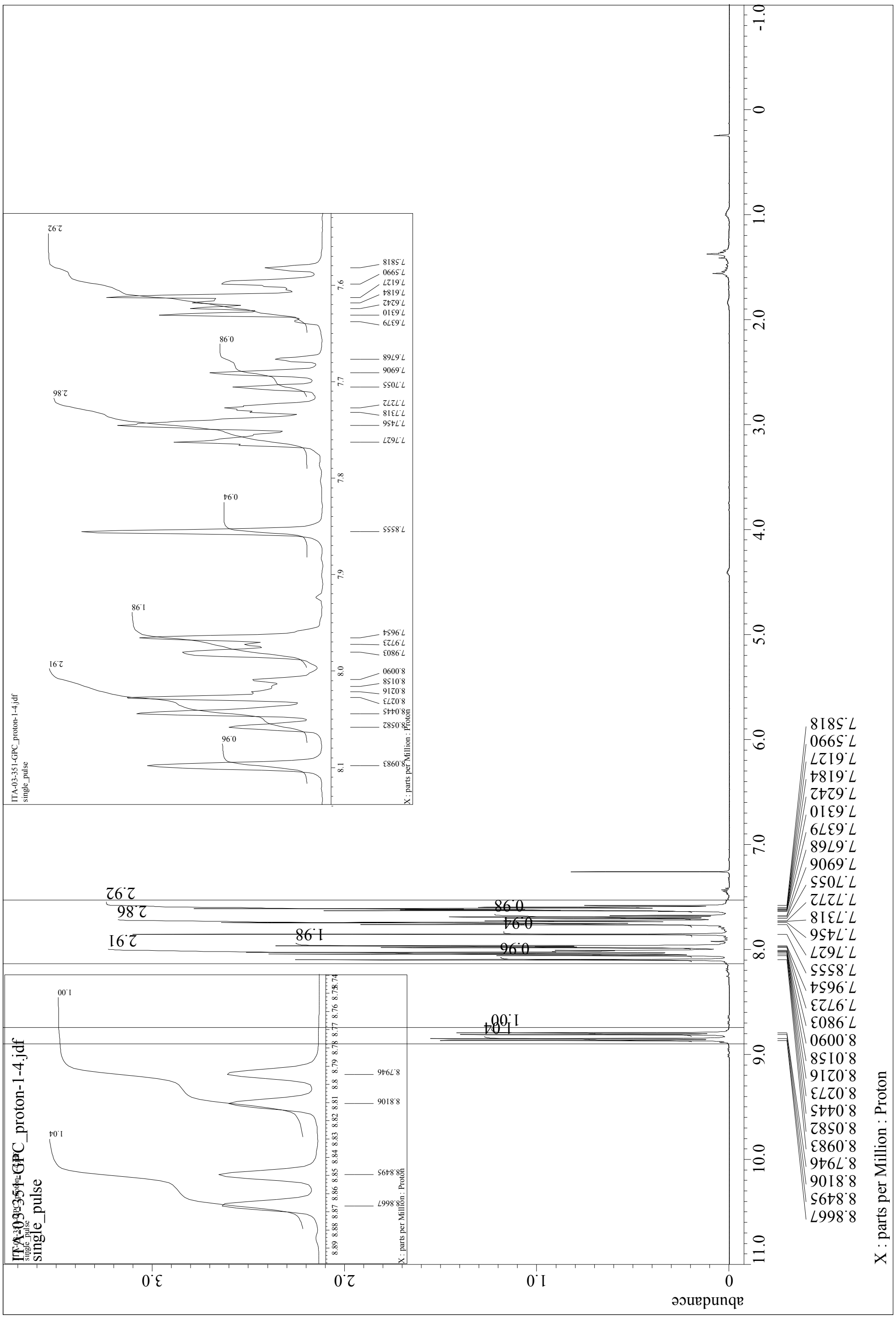

Supplementary Figure 87. ^{1}H NMR spectrum of **6a**

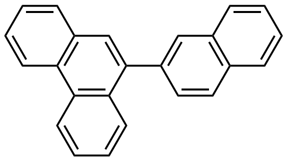

6a (^{13}C NMR, 126 MHz, $CDCl_3$)

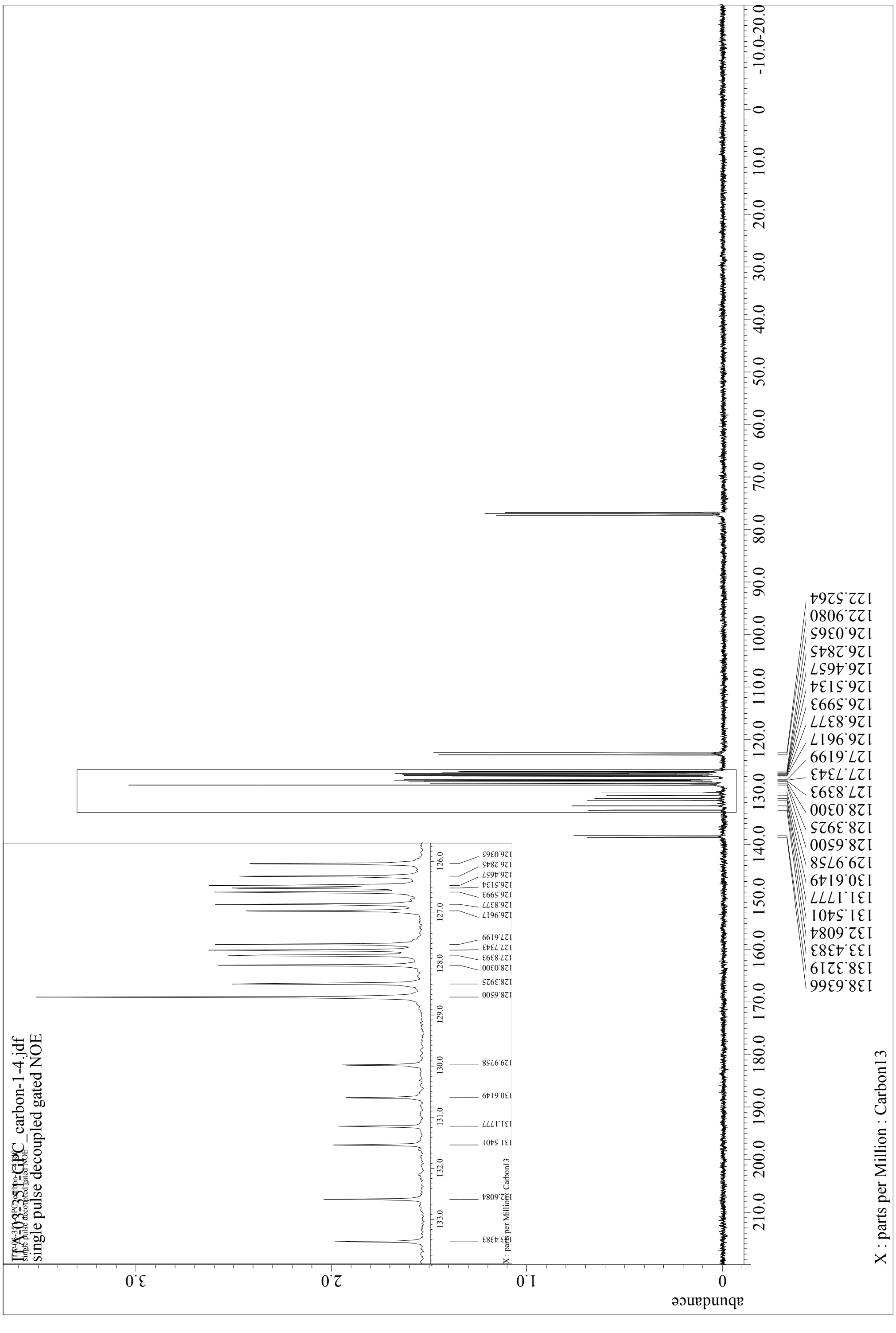

Supplementary Figure 88. ^{13}C NMR spectrum of **6a**

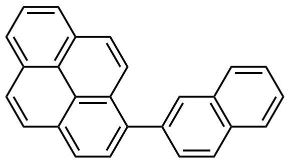

6b (^{1}H NMR, 500 MHz, $CDCl_3$)

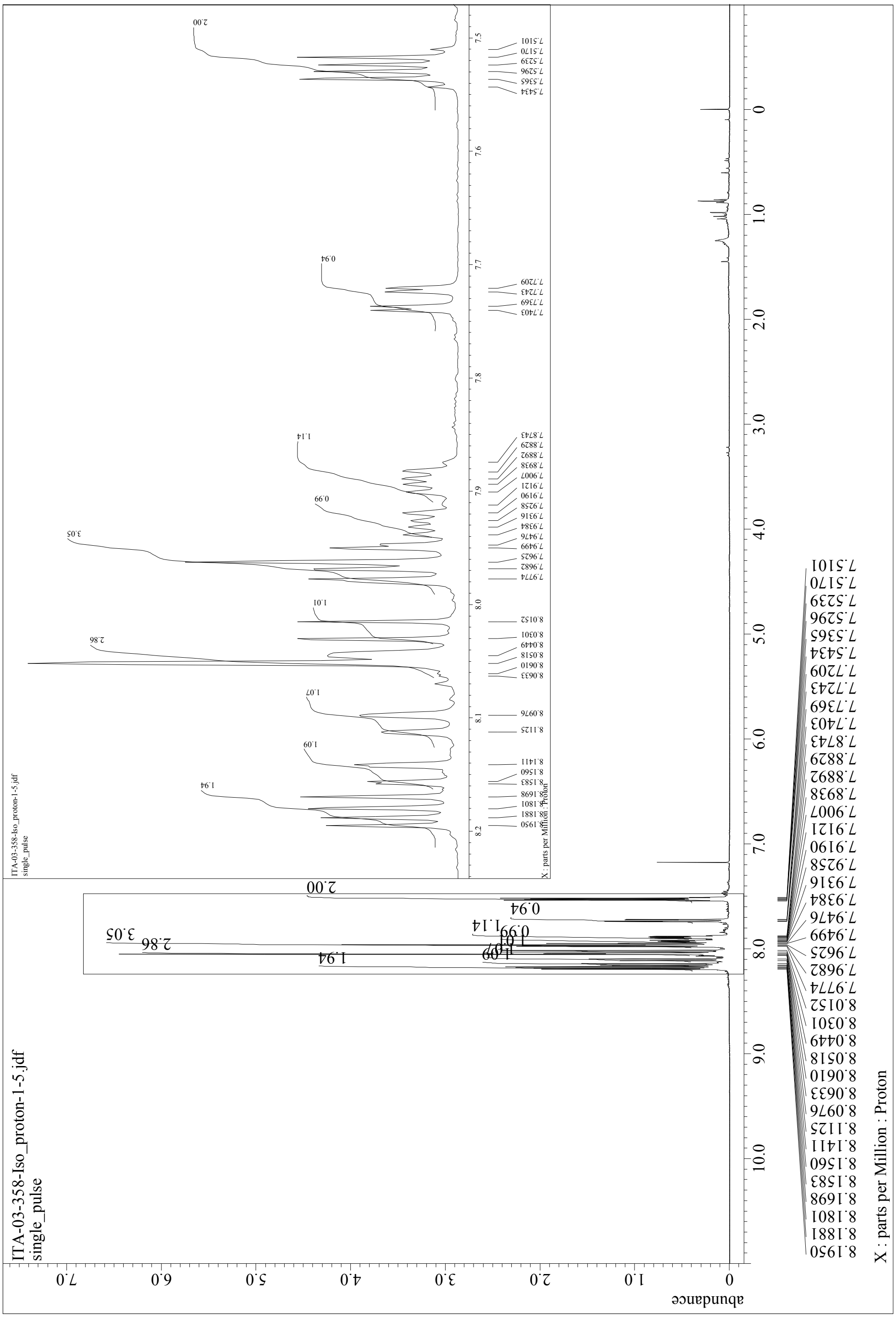

Supplementary Figure 89. ^{1}H NMR spectrum of **6b**

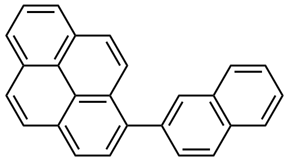

6b (^{13}C NMR, 126 MHz, $CDCl_3$)

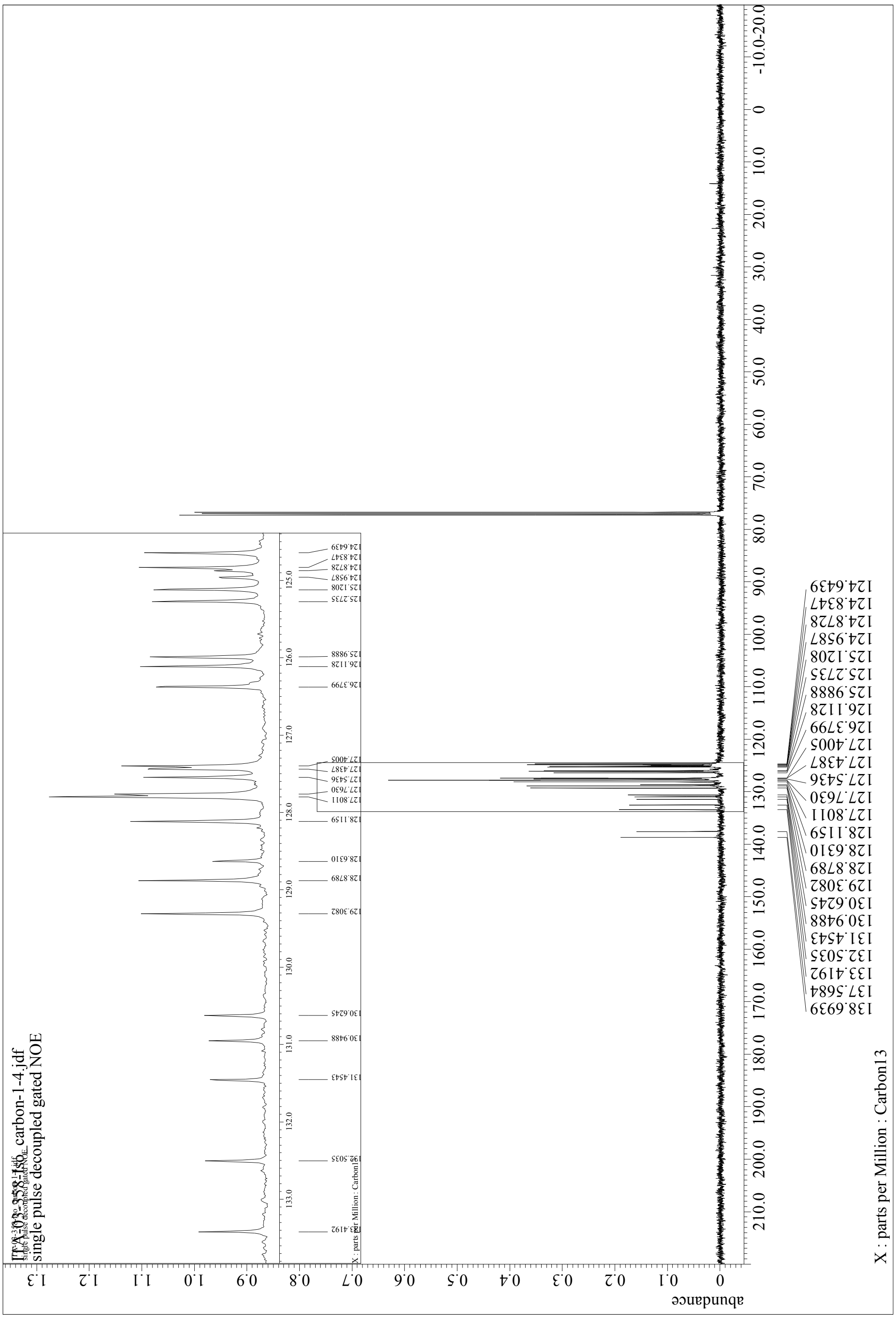

Supplementary Figure 90. ^{13}C NMR spectrum of **6b**

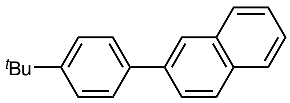

6c (^{1}H NMR, 500 MHz, $CDCl_3$)

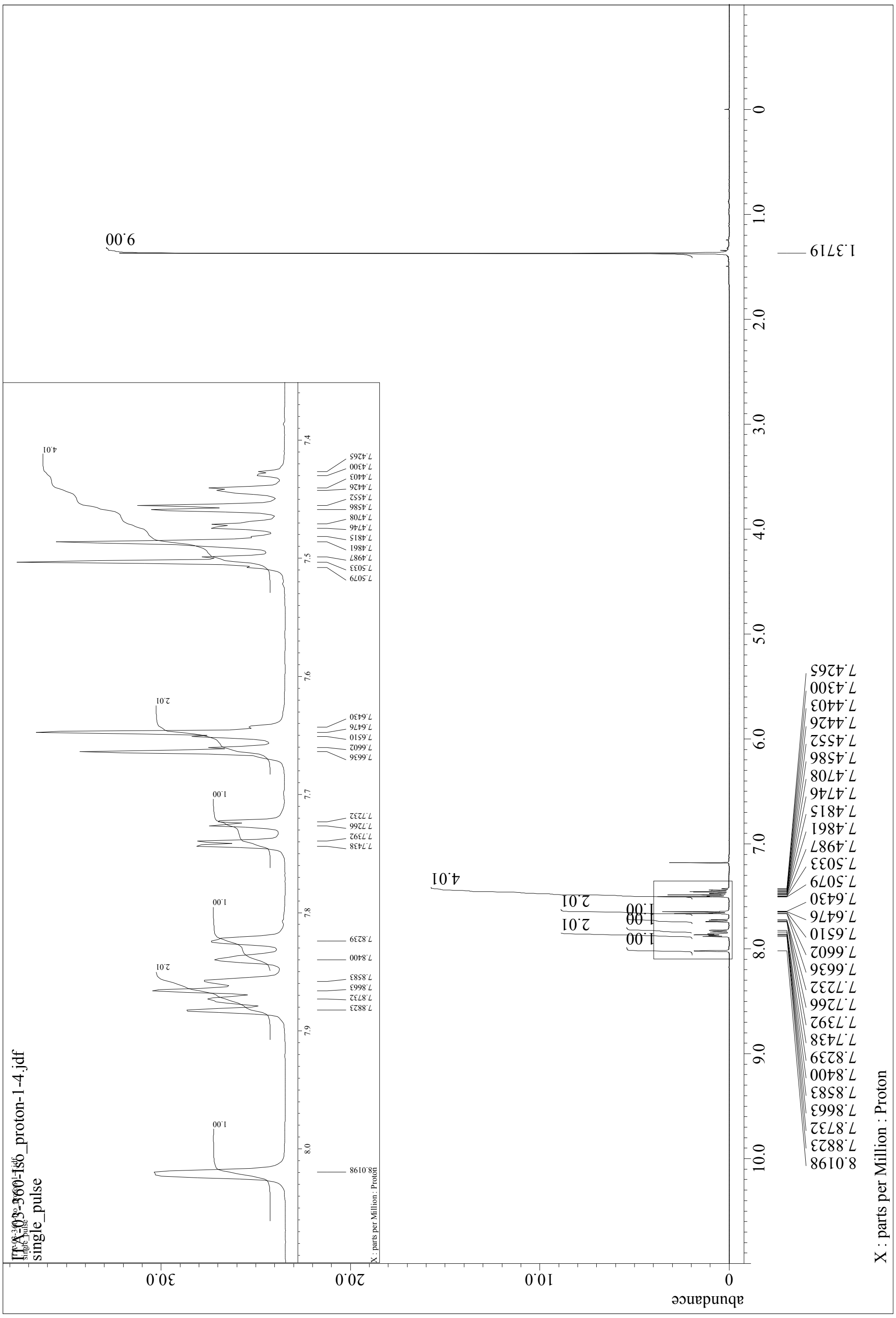

Supplementary Figure 91. ^{1}H NMR spectrum of **6c**

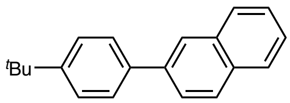

6c (^{13}C NMR, 126 MHz, $CDCl_3$)

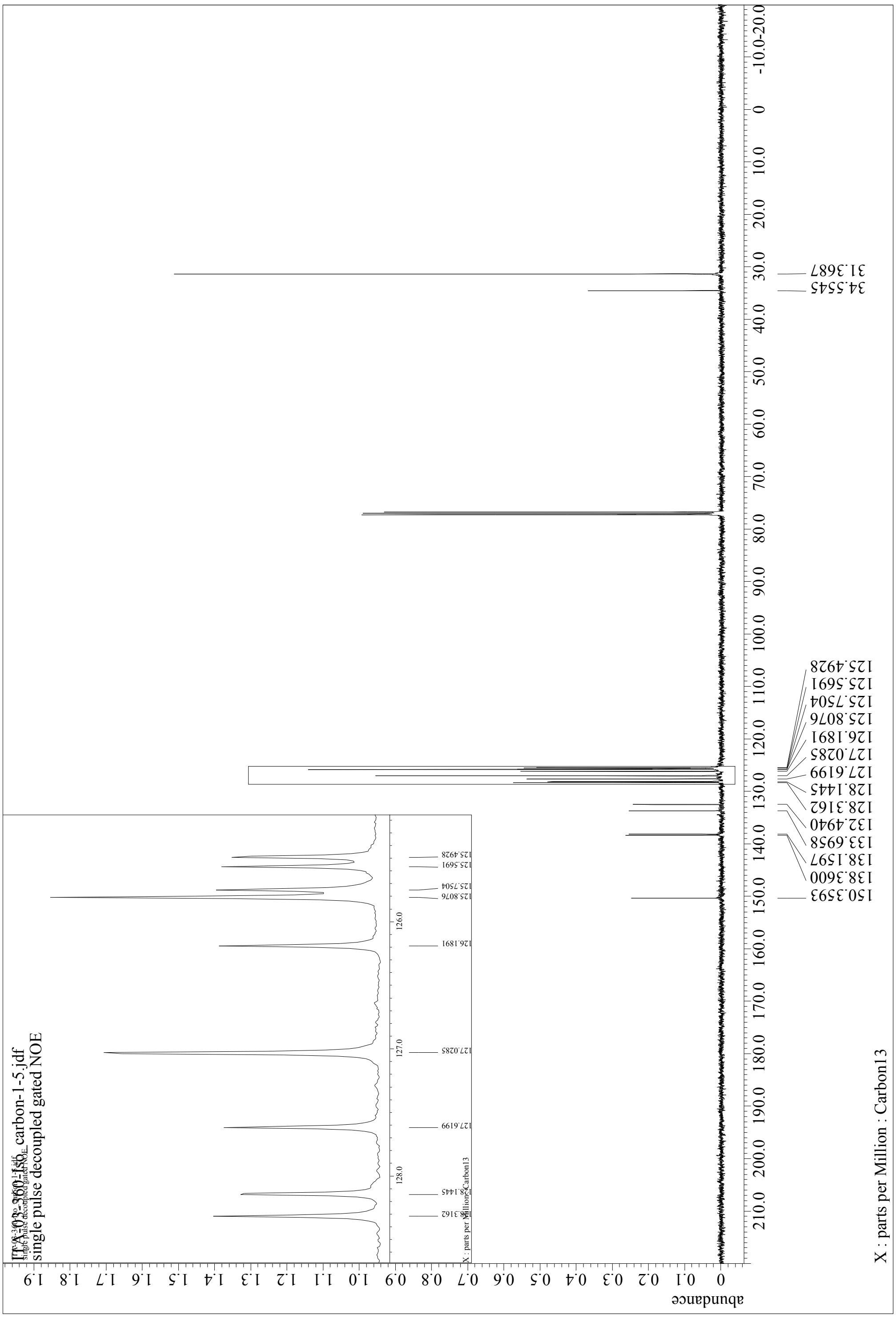

Supplementary Figure 92. ^{13}C NMR spectrum of **6c**

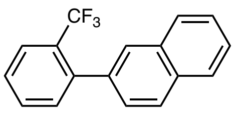

6d (^{1}H NMR, 500 MHz, $CDCl_3$)

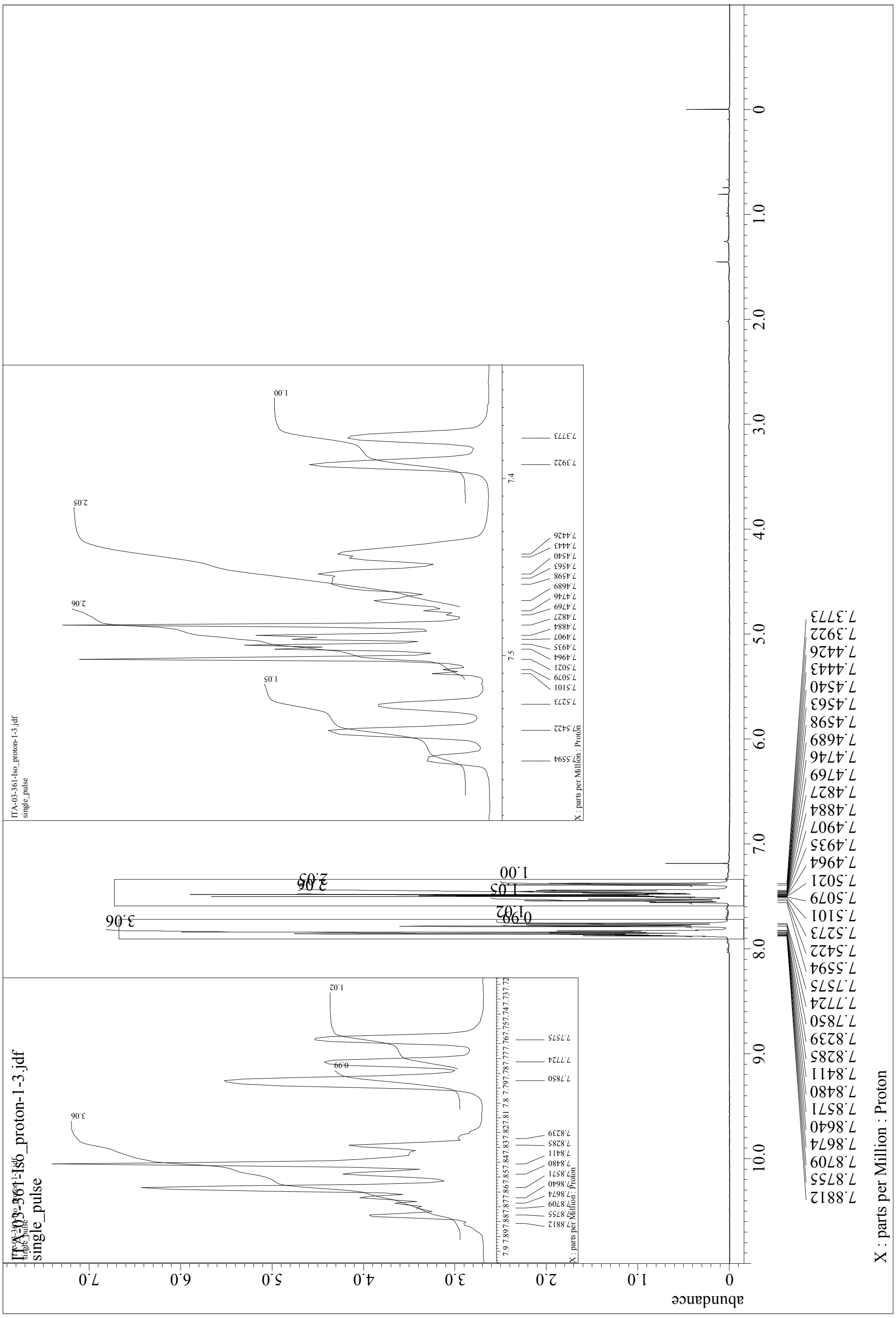

Supplementary Figure 93. ^{1}H NMR spectrum of **6d**

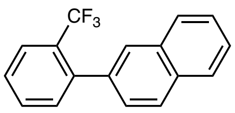

6d (^{13}C NMR, 126 MHz, $CDCl_3$)

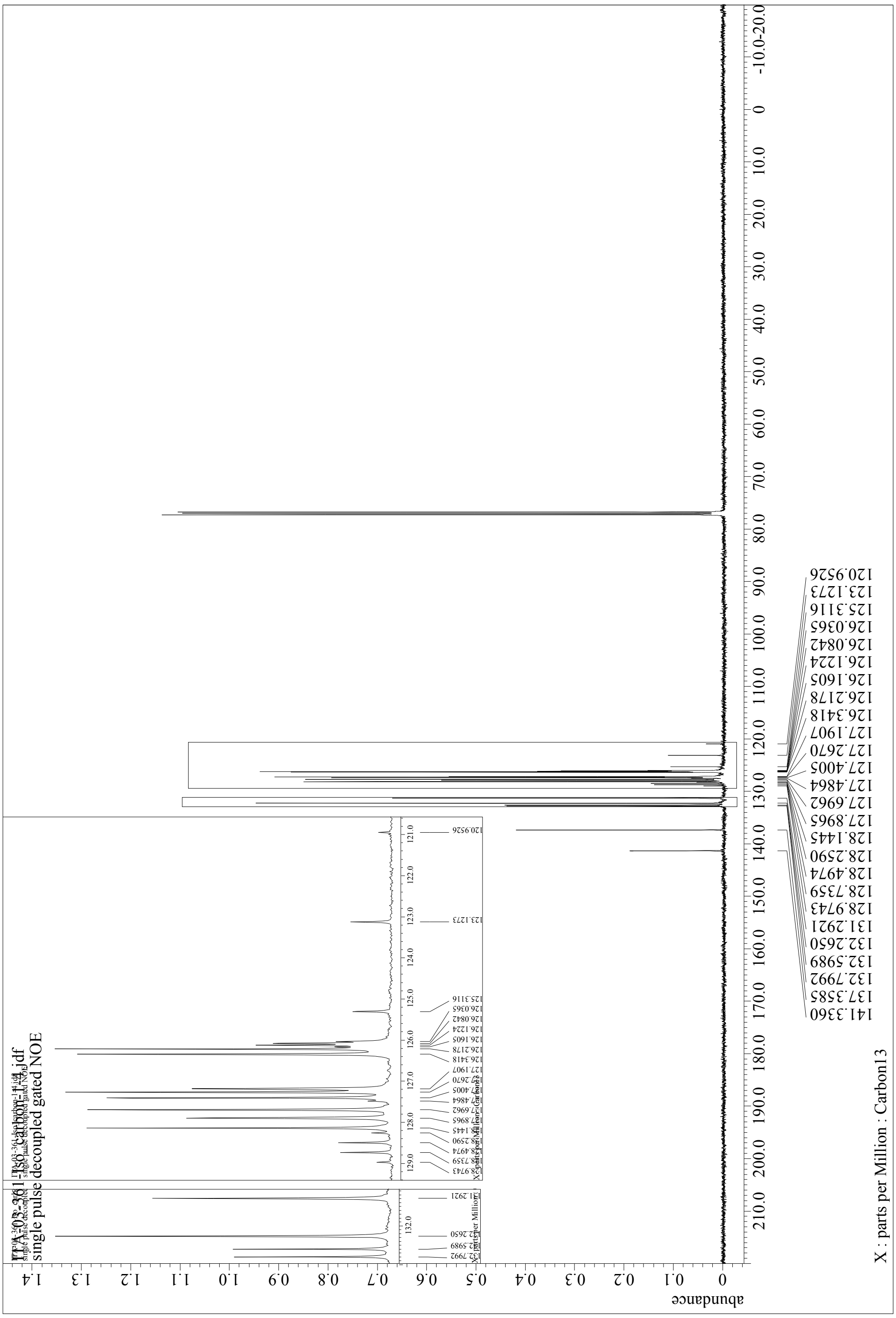

Supplementary Figure 94. ^{13}C NMR spectrum of **6d**

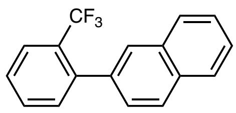

6d (^{19}F NMR, 470 MHz, $CDCl_3$)

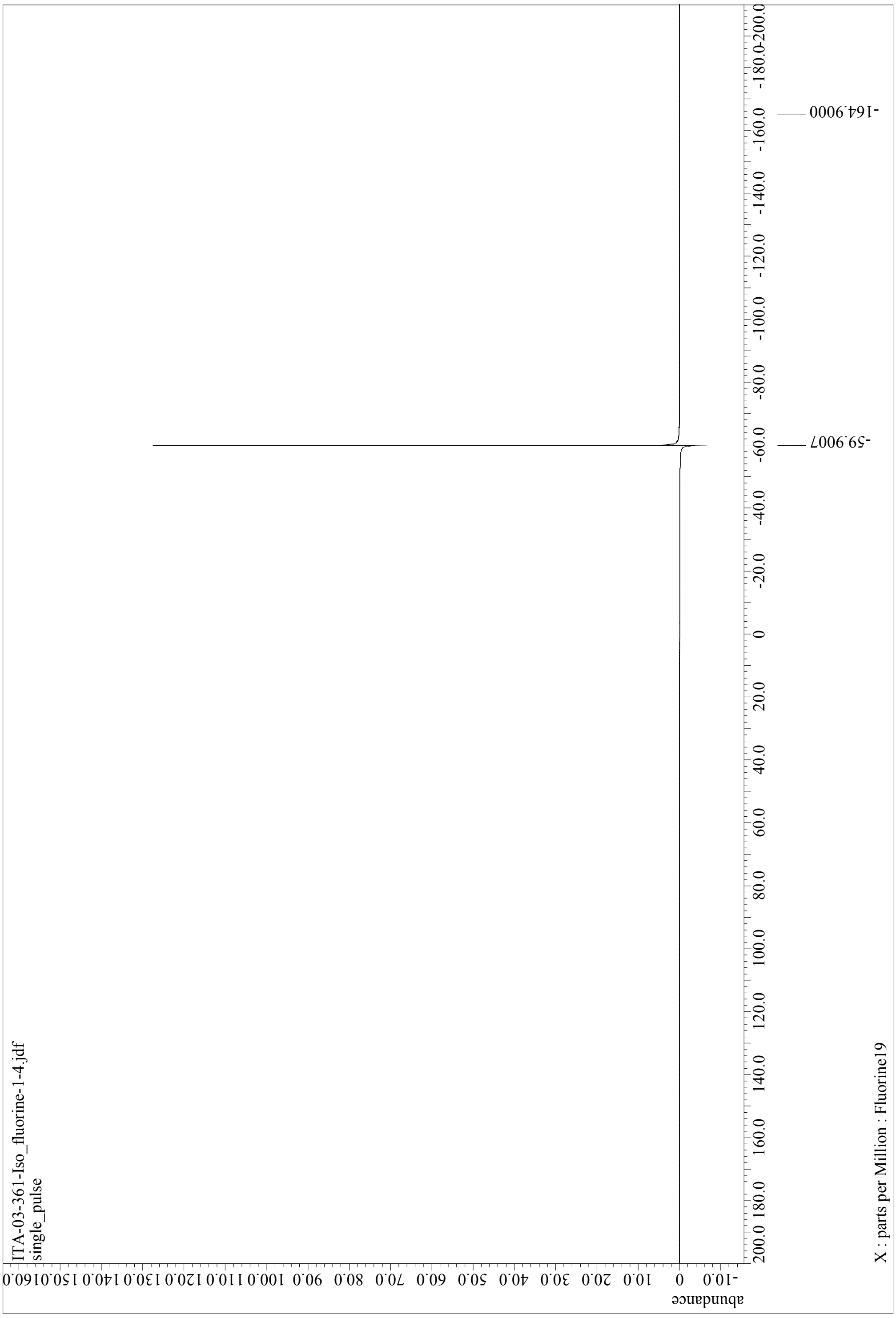

Supplementary Figure 95. ^{19}F NMR spectrum of **6d**

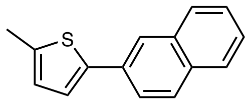

6e (^{1}H NMR, 500 MHz, $CDCl_3$)

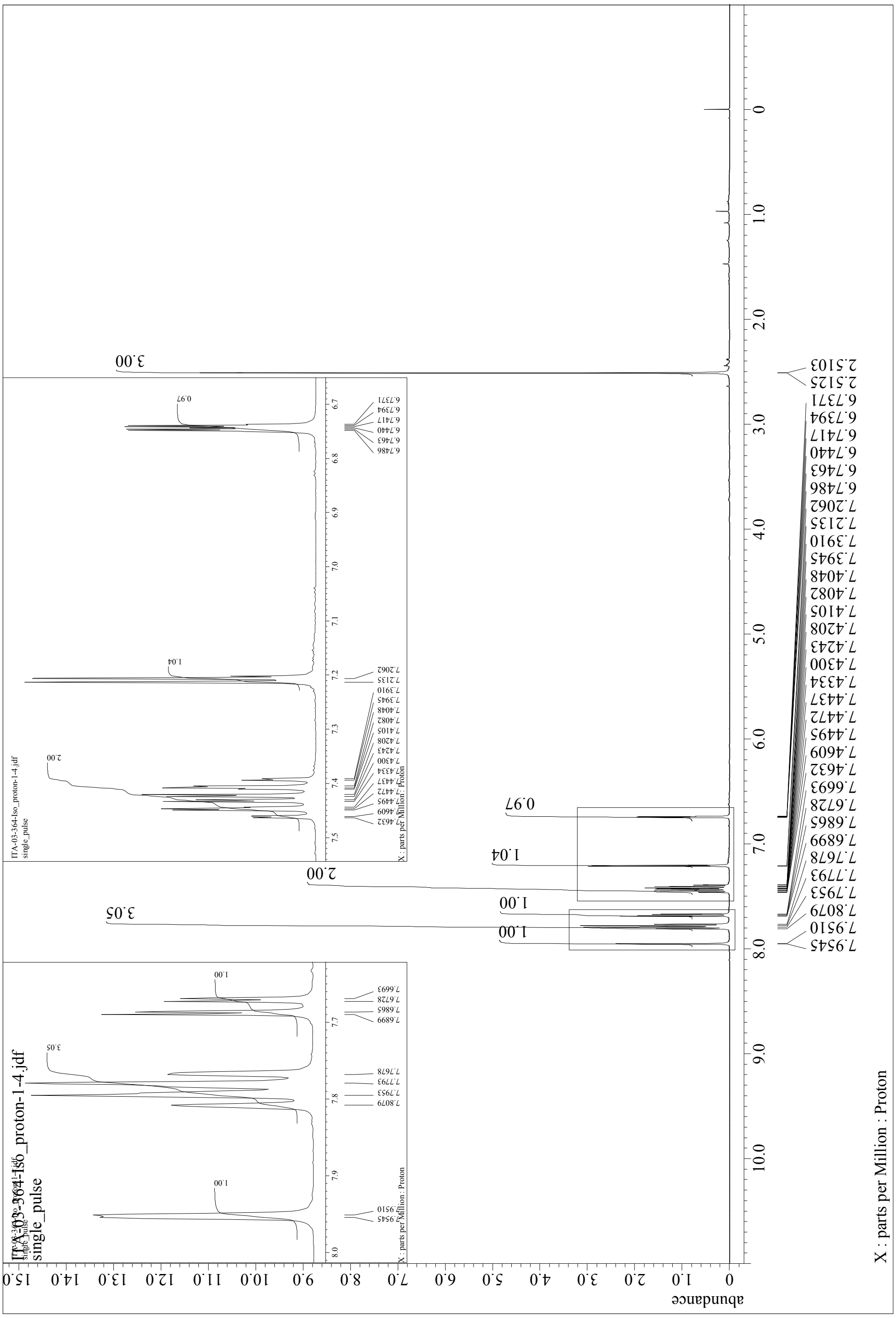

Supplementary Figure 96. ^{1}H NMR spectrum of **6e**

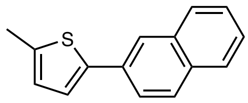

6e (^{13}C NMR, 126 MHz, $CDCl_3$)

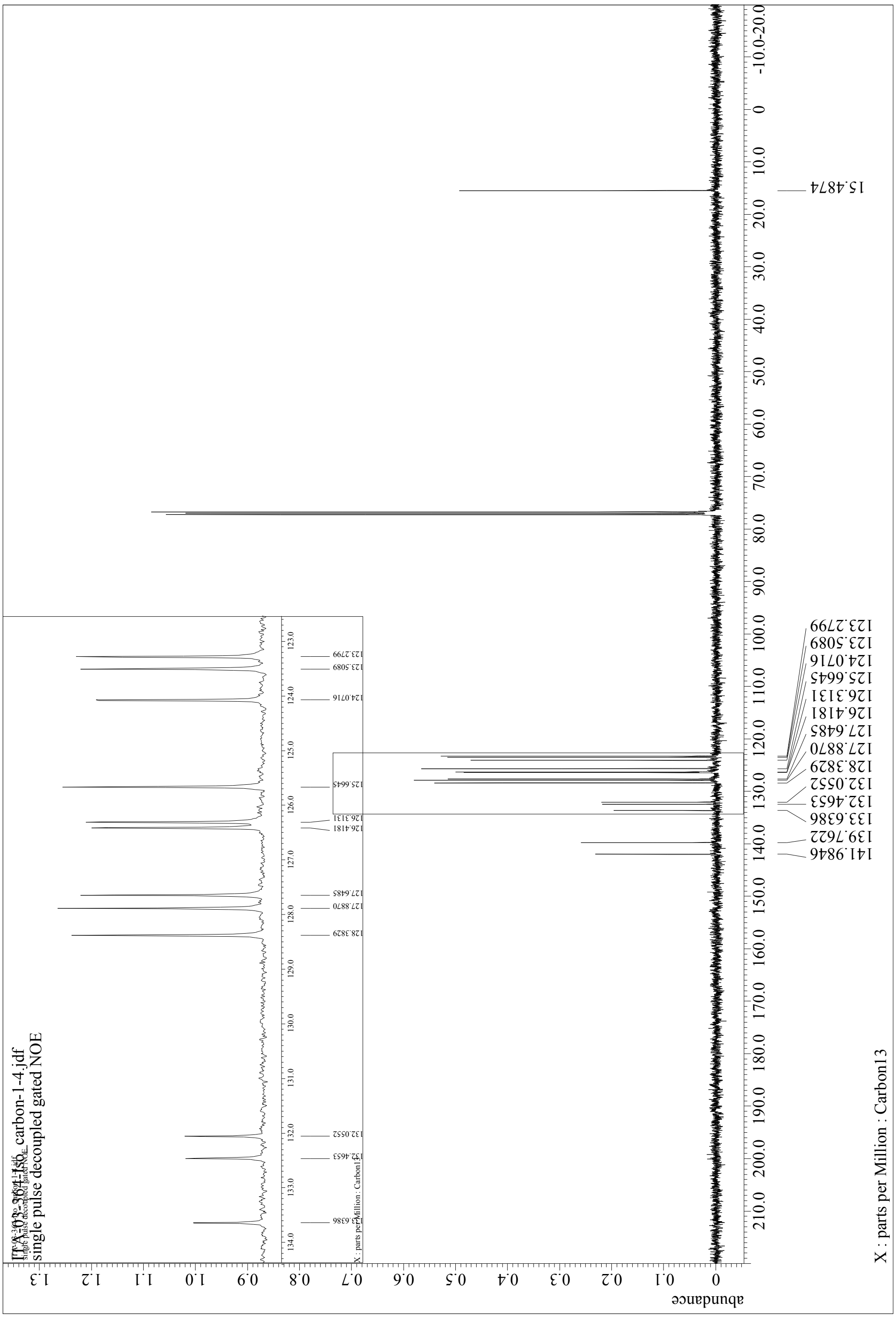

Supplementary Figure 97. ^{13}C NMR spectrum of **6e**
